# The *bZIP* gene family in watermelon: genome-wide identification and expression analysis under cold stress and root-knot nematode infection

**DOI:** 10.7717/peerj.7878

**Published:** 2019-10-16

**Authors:** Youxin Yang, Jingwen Li, Hao Li, Yingui Yang, Yelan Guang, Yong Zhou

**Affiliations:** 1 Key Laboratory of Crop Physiology, Ecology and Genetic Breeding, Ministry of Education, Jiangxi Agricultural University, Nanchang, Jiangxi, China; 2 Jiangxi Key Laboratory for Postharvest Technology and Nondestructive Testing of Fruits & Vegetables, Collaborative Innovation Center of Post-Harvest Key Technology and Quality Safety of Fruits and Vegetables, College of Agronomy, Jiangxi Agricultural University, Nanchang, Jiangxi, China; 3 State Key Laboratory of Crop Stress Biology for Arid Areas, College of Horticulture, Northwest A & F University, Yangling, Shaanxi, China; 4 Department of Biochemistry and Molecular Biology, College of Bioscience and Bioengineering, Jiangxi Agricultural University, Nanchang, Jiangxi, China

**Keywords:** Watermelon (*Citrullus lanatus*), bZIP transcription factor, Cold stress, Genome-wide analysis, Root-knot nematode, Expression pattern

## Abstract

The basic leucine zipper (bZIP) family transcription factors play crucial roles in regulating plant development and stress response. In this study, we identified 62 *ClabZIP* genes from watermelon genome, which were unevenly distributed across the 11 chromosomes. These ClabZIP proteins could be classified into 13 groups based on the phylogenetic relationships, and members in the same group showed similar compositions of conserved motifs and gene structures. Transcriptome analysis revealed that a number of *ClabZIP* genes have important roles in the melatonin (MT) induction of cold tolerance. In addition, some *ClabZIP* genes were induced or repressed under red light (RL) or root-knot nematode infection according to the transcriptome data, and the expression patterns of several *ClabZIP* genes were further verified by quantitative real-time PCR, revealing their possible roles in RL induction of watermelon defense against nematode infection. Our results provide new insights into the functions of different *ClabZIP* genes in watermelon and their roles in response to cold stress and nematode infection.

## Introduction

Plants have developed complex signaling transduction pathways to protect themselves against a variety of biotic and abiotic environmental stimuli. Various transcription factors (TFs) can bind to the *cis*-acting elements in the promoters of stress-responsive genes for regulating their expression to control the signaling networks of plant development and stress responses (Jin et al., 2017). Notably, the basic leucine zipper (bZIP) family is one of the largest TF families named after a shared highly conserved bZIP domain. The bZIP domain is composed of 60–80 amino acids in length and possesses two functionally distinct parts: a highly conserved basic region and a less conserved leucine zipper, which are linked by a hinge region (Correa et al., 2008; Dröge-Laser et al., 2018; Wang et al., 2018b). The basic region contains a characteristic motif (N-X7-R/K-X9) responsible for DNA-binding and nuclear localization, while the leucine zipper forms an amphipathic surface that mediates specific recognition and dimerization (Hu et al., 2016c; Li et al., 2016b).

In plants, members of bZIPs have been reported to take part in various developmental processes, such as pollen development (Gibalova et al., 2017; Iven et al., 2010; Li et al., 2015b), seed maturation (Jain et al., 2017; Zinsmeister et al., 2016), floral transition and initiation (Abe et al., 2005; Wang et al., 2013), and root development (Kim, Yamaguchi-Shinozaki & Shinozaki, 2018; Ma et al., 2018). Besides, accumulating evidence has suggested that plant *bZIP* genes act as key components that regulate responses to various abiotic stresses, and the functions of *bZIP* genes in stress tolerance are usually realized via abscisic acid (ABA)-dependent pathway. For example, a grapevine bZIP TF, VlbZIP30, serves as a positive regulator of dehydration stress through ABA signaling pathway (Tu et al., 2018). In rice, many *OsbZIP* genes also contribute to stress resistance by mediating ABA signaling, such as *OsbZIP42* (Joo, Lee & Song, 2019), *OsbZIP46/OsABF2* (Ma et al., 2019; Tang et al., 2012, 2016), *OsbZIP66* (Yoon et al., 2017), *OsbZIP71* (Liu et al., 2014), and *OsbZIP72* (Lu et al., 2009). In addition, a positive role of *bZIPs* in defense against bacterial pathogens was also observed in some plants (Li et al., 2017c; Lim et al., 2015), suggesting the importance of their immune functions. Abiotic/biotic stress can increase the endogenous level of melatonin (MT), which may serve as a secondary messenger for protecting plants against multiple abiotic and biotic stresses by increasing the expression and activities of antioxidant enzymes, improving photosynthesis and redox homeostasis, and regulating the expression of stress-responsive genes (Li et al., 2017a, 2018; Sharif et al., 2018; Shi et al., 2015b; Zhang et al., 2015). Some *bZIP* genes are significantly regulated by exogenous MT treatment, suggesting that bZIP TFs may also be involved in stress response through MT-mediated signaling pathway (Li et al., 2017b; Liang et al., 2015; Shi et al., 2015a). Moreover, the studies on plant *bZIP* genes have revealed their roles in the regulation of light response in recent years. For example, *Arabidopsis* ELONGATED HYPOCOTYL5 (HY5) acts downstream of multiple photoreceptors including phytochromes, cryptochromes and UV-B photoreceptor UV RESISTANCE LOCUS8 (UVR8) and regulates photomorphogenesis, chloroplast development, pigment accumulation, and defense response (Binkert et al., 2014; Gangappa & Botto, 2016; Yang et al., 2018a). Low red/far-red ratio and cold stress can induce the expression of *SlHY5* in a PHYTOCHROME A-dependent manner, and *SlHY5* can inhibit the growth and induce cold tolerance through integrating the temperature, light, and hormone signaling pathways in tomato (Wang et al., 2018a, 2019). These findings demonstrate that plant *bZIPs* play vital roles in regulating numerous developmental processes and responses to various abiotic/biotic stresses.

As one of many economically important crops consumed worldwide, watermelon is particularly susceptible to different biotic and abiotic stresses during developmental processes. Plant-parasitic nematodes can attack numerous economically important crops and cause a global yield loss of up to 12.3% on average (Holbein, Grundler & Siddique, 2016). Root-knot nematodes (RKNs), *Meloidogyne* spp., are sedentary endoparasitic nematodes that parasitize many agricultural crop plants including watermelon (Bebber, Holmes & Gurr, 2014; Yang et al., 2018b). *Meloidogyne incognita* is considered as the most devastating plant disease-causing agent, which may increase susceptibility to other pathogenic diseases and finally significantly undermine agricultural productivity (Bebber, Holmes & Gurr, 2014; Yang et al., 2015). Our previous studies have shown that light (especially red light, RL) plays vital roles in the defense response of plants to the RKN *M. incognita* (Yang et al., 2015, 2018b), which may contribute to environment-friendly strategies to control RKNs in plants. A recent report has identified 59 *bZIP* genes in watermelon, and found that some of them may be involved in drought stress response (Unel et al., 2019). Although the chromosomal distributions, phylogenetic relationships, conserved motifs, and gene structures of the 59 *ClabZIPs* have been analyzed in a previous study (Unel et al., 2019), these analyses were insufficient to comprehensively reveal the information of the *bZIP* family genes in watermelon. Moreover, there is still limited information on the functional properties of *ClabZIP* genes during the growth and development of watermelon, as well as in plant defense against different biotic and abiotic stresses. In the present study, we performed comprehensive analyses of the *bZIP* family genes in watermelon and a total of 62 *ClabZIP* genes were identified. These ClabZIPs could be precisely classified into 13 groups based on the evolutionary relationships, and members in the same group showed similar compositions of conserved motifs and gene structures. To explore the functions of watermelon *bZIP* genes, we determined the tissue-specific expression of selected *ClabZIP* genes and the global expression profiles of *ClabZIP* genes in response to RL and nematode treatments and MT induction of cold tolerance. Our results are expected to lay a foundation for functional analysis of watermelon *bZIP* genes in the future, and provide clues for revealing their possible roles in nematode infection and cold stress in watermelon.

## Materials and Methods

### Identification and protein properties of ClabZIPs

Watermelon *Citrullus lanatus* subsp. *vulgaris* cv. 97103 genome and protein sequences were downloaded from the cucurbit genomics database (CuGenDB; http://cucurbitgenomics.org). To identify the watermelon *bZIP* family genes, the bZIP domains (PF00170, PF07716, and PF03131) downloaded from Pfam (http://pfam.sanger.ac.uk/) were used to search the watermelon protein sequences by HMMER software with an *e*-value cutoff of 1*e*^−5^. BlastP search was also performed against watermelon protein sequences by using the *Arabidopsis* and rice bZIP protein sequences as queries with the cutoff *e*-value set at 1*e*^−5^. The AtbZIP protein sequences of *Arabidopsis thaliana* were downloaded from the *Arabidopsis* Information Resource database (http://www.arabidopsis.org/) according to the protein IDs in a previous report (Dröge-Laser et al., 2018). After removal of redundant sequences, these potential bZIP proteins were further checked for the presence of a bZIP domain by the simple modular architecture research tool (SMART) server (http://smart.embl-heidelberg.de/), and the proteins without the bZIP domain were deleted. The amino acid sequences of the watermelon bZIP proteins are listed in Table S1. The Protparam program (http://web.expasy.org/protparam/) was employed to examine the theoretical protein properties of ClabZIPs, including molecular weight (MW) and isoelectric point (pI). The gene ontology (GO) annotations of watermelon bZIP family members were obtained from the watermelon genome database (http://cucurbitgenomics.org/organism/1) and visualized by using WEGO software (http://wego.genomics.org.cn/).

### Multi-sequence alignment, phylogenesis, protein motif, and gene structure analysis

The full-length sequences of bZIP proteins were used to analyze their phylogenetic relationships. The amino acid sequences of ClabZIPs and AtbZIPs (Tables S1 and S2) were aligned using MAFFT (https://www.ebi.ac.uk/Tools/msa/mafft/) with default parameters. Then, an unrooted neighbour-joining (NJ) phylogenetic tree was constructed with MEGA 7.0 based on the alignment results using bootstrap replications of 1,000. To identify the conserved motifs in the ClabZIP proteins, motif search was performed by MEME online software (http://meme-suite.org/tools/meme) and the results were visualized with TBtools (Chen et al., 2018). The numbers of motifs were set at ten, and the motif widths were set at 6 and 50. Other parameters were set as default parameters. To identify the gene structure of the *ClabZIP* genes, their CDS sequences and corresponding genomic DNA (gDNA) sequences (Tables S3 and S4) were aligned by the GSDS online software (http://gsds.cbi.pku.edu.cn).

### Chromosomal location and duplication analysis of *ClabZIP* genes

To determine the chromosomal locations of *bZIP* genes in watermelon genome, the information of locus coordinates was downloaded from the watermelon genome database (http://cucurbitgenomics.org/organism/1), and the distributions of *ClabZIP* genes on the chromosomes were visualized using Map Chart 3.2. Before determining the chromosomal locations of the *ClabZIP* genes, the alternative splicing forms from the same gene locus were examined by using the watermelon genome annotation, and no alternative splicing events in these genes were identified. Gene duplication analysis was performed based on a previous study (Wang et al., 2018b).

### In silico expression analysis of *ClabZIP* genes

The details of RKN infection experiment have been illustrated in our previous report (Yang et al., 2018b). A total of 24 samples of leaves and roots from four different treatments, including control (mock, white light, and water solution), RL (red light treatment and water solution), RKN (white light and RKN *M. incognita* infection), and RL+RKN (red light treatment and root-knot nematode *M. incognita* infection), were sequenced on the Illumina HiSeq X Ten platform and paired-end reads were generated for transcriptome sequencing. The sequencing raw sequence data were deposited in the genome sequence Archive in the BIG Data Center GSA database, Beijing institute of Genomics (BIG), Chinese Academy of Sciences, under the accession numbers of CRA001311 and CRA001312. The genome-wide transcriptome data of watermelon (*Citrullus lanatus* L., cv. Y134) treated with MT and cold were obtained under the accession numbers of SRP078211 and SRA438977 (Li et al., 2017b). The gene expression levels were estimated with fragments per kilobase of exon per million fragments mapped (FPKM) values extracted from the above mentioned transcriptome data using the Top Hat/Cufflinks pipeline according to previous reports (Li et al., 2017b; Yang et al., 2015), and presented in Table S5. The log2-transformed FPKM values were used to create a heatmap to depict the expression of each *ClabZIP* gene by using the OmicShare Tools (http://www.omicshare.com/tools/Home/Index/index.html).

### Plant materials and treatments

Watermelon (*C. lanatus* L. cv. Xinong 8) seeds were sown in trays filled with nutritional soil and placed in the greenhouse of the practice base of Jiangxi Agriculture University, Nanchang, China. For tissue-specific analysis, the roots, stems, expanding leaves, mature leaves, stem apexes, fruits, and flowers were separately sampled from 2-month-old watermelon plants. For cold treatment, watermelon seedlings were grown in Hoagland solution under a photoperiod of 25 °C/19 °C (12 h/12 h), a photosynthetic photon flux density of 200 µmol · m^−2^ · s^−1^ supplied from fluorescent tubes, and a relative humidity of 70% in growth chambers. At four-leaf stage of watermelon plants, low-temperature treatment was carried out at 4 °C under the same photoperiod and light conditions. The leaves were sampled at 0 (as the control), 1, 3, 9, and 24 h after treatments for analysis.

### RNA extraction and quantitative real-time PCR

Total RNA was extracted from the above samples using the total RNA Miniprep Kit (Axygen Biosciences, Union City, CA, USA) according to the manufacturer’s protocol, and approximately one μg of purified total RNA was reverse-transcribed for the synthesis of cDNA using the ReverTra Ace qPCR-RT Kit (Toyobo, Osaka, Japan) according to the manufacturer’s instruction. To analyze the relative transcript levels of selected genes, quantitative real-time PCR (qRT-PCR) was performed using the iCycler iQTM Real-time PCR Detection System (Bio-Rad, Hercules, CA, USA). The PCR conditions were as follows: denaturation at 95 °C for 3 min, followed by 40 cycles of denaturation at 95 °C for 30 s, annealing at 58 °C for 30 s, and extension at 72 °C for 30 s. The software provided with the PCR system was used to calculate the threshold cycle values and to quantify the mRNA expression levels based on the 2^−ΔΔCT^ method (Livak & Schmittgen, 2001). Watermelon β-*actin* gene was selected as the internal control. The primers used for qRT-PCR are listed in Table S6. The statistically significant differences of expression data were determined when *P*-values were < 0.05 using one-way analysis of variance with Tukey’s test.

## Results

### Genome-wide identification of *bZIP* family genes in watermelon

The watermelon genome database was used to perform genome-wide identification of *bZIP* family genes by using HMMER and BlastP. A total of 62 genes were identified and named as *ClabZIP1* to *ClabZIP62* (Table 1) according to their chromosomal distributions and the nomenclature of a previous study (Unel et al., 2019). The identified *ClabZIP* genes included the previously reported 59 *bZIP* genes in watermelon (Unel et al., 2019), along with three new *bZIP* genes (*ClabZIP60–ClabZIP62*). The predicted ClabZIP proteins ranged from 85 (ClabZIP44) to 936 (ClabZIP42) amino acids in length, and their calculated MW ranged from 10.01 to 82.8 kDa and theoretical pI was from 4.49 to 11.1 (Table 1). SMART analysis showed that most of the ClabZIP proteins contained only one bZIP domain, but there were 15 ClabZIP proteins possessing additional domains, such as multifunctional mosaic region (MFMR) and DELAY OF GERMINATION (DOG) (Table 1). The GO annotation results indicated that ClabZIP proteins may participate in various biological processes (Table S5; Fig. S1).

**Table 1:** The information of *bZIP* family members identified from *Citrullus lanatus* genome.

Nomenclature	CGD	Protein length (aa)	Chain	Chromosome	Group	Domain	Start	End	Molecular weight (kDa)	Theoretical pI
ClabZIP1	Cla005880	162	+	1	S	bZIP (PF00170)	54	112	18.79	5.76
ClabZIP2	Cla000383	253	+	0	A	bZIP (PF00170)	193	250	28.09	5.02
ClabZIP3	Cla014048	217	+	1	S	bZIP (PF00170)	95	140	25.28.	7.07
ClabZIP4	Cla014195	367	+	1	G	MFMR (PF07777) bZIP (PF00170)	267	329	38.65	6.4
ClabZIP5	Cla014247	159	+	1	S	bZIP (PF00170)	29	87	18.41	7.96
ClabZIP6	Cla015627	377	−	2	E	bZIP_2 (PF07716)	188	236	36.92	5.67
ClabZIP7	Cla015828	267	−	2	F	bZIP_2 (PF07716)	86	142	28.73	5.7
ClabZIP8	Cla015873	576	−	2	I	bZIP_2 (PF07716)	419	471	61.71	5.89
ClabZIP9	Cla015874	513	−	2	I	bZIP_2 (PF07716)	418	470	54.88	6.06
ClabZIP10	Cla016019	408	−	2	A	bZIP (PF00170)	330	380	44.83	9.59
ClabZIP11	Cla020278	273	+	2	F	bZIP_2 (PF07716)	87	140	29.48	5.93
ClabZIP12	Cla019809	428	+	2	C	bZIP (PF00170) bZIP_C (PF12498)	227 296	285 421	46.01	6.34
ClabZIP13	Cla013418	377	+	2	I	bZIP (PF00170)	178	226	40.77	6.51
ClabZIP14	Cla008649	441	+	2	D	DOG (PF14144) bZIP (PF00170)	207 288	248 366	49.47	6.5
ClabZIP15	Cla008141	240	−	3	M	bZIP (PF00170)	110	167	27.29	8.81
ClabZIP16	Cla011083	247	+	3	A	bZIP (PF00170)	203	246	27.76	9.26
ClabZIP17	Cla011295	379	−	3	C	bZIP (PF00170) bZIP_C (PF12498)	200 269	255 370	41.42	8.92
ClabZIP18	Cla021184	417	+	5	G	MFMR (PF07777) bZIP (PF00170)	1 281	185 343	44.24	9.05
ClabZIP19	Cla021868	334	−	5	I	bZIP (PF00170)	231	279	36.33	5.62
ClabZIP20	Cla021871	319	−	5	I	bZIP (PF00170)	222	270	34.57	5.63
ClabZIP21	Cla004308	321	−	5	E	bZIP (PF00170)	243	281	36.12	7.17
ClabZIP22	Cla020959	210	−	5	H	bZIP (PF00170)	77	136	23.42	9.77
ClabZIP23	Cla020795	165	−	5	S	bZIP (PF00170)	37	95	19.28	6.29
ClabZIP24	Cla020334	200	+	5	S	bZIP (PF00170)	83	139	22.95	6.25
ClabZIP25	Cla009958	155	−	5	S	bZIP (PF00170)	57	115	18.41	9.75
ClabZIP26	Cla007293	349	−	7	I	bZIP_2 (PF07716)	140	191	38.05	5.97
ClabZIP27	Cla014572	151	+	7	S	bZIP (PF00170)	25	83	17.24	5.41
ClabZIP28	Cla014501	362	−	7	D	DOG (PF14144) bZIP (PF00170)	77 163	118 241	41.26	7.07
ClabZIP29	Cla010797	109	−	7	S	bZIP (PF00170)	26	75	13	6.42
ClabZIP30	Cla007950	146	+	8	S	bZIP (PF00170)	62	120	17.59	7.82
ClabZIP31	Cla007982	467	+	8	D	DOG (PF14144) bZIP (PF00170)	180 264	221 342	51.57	5.83
ClabZIP32	Cla013824	352	−	8	I	bZIP_2 (PF07716)	187	238	38.11	9.07
ClabZIP33	Cla013813	236	+	8	A	bZIP (PF00170)	176	221	25.92	5.1
ClabZIP34	Cla013666	300	+	8	A	bZIP (PF00170)	246	289	33.43	7.82
ClabZIP35	Cla022056	333	−	8	D	DOG (PF14144) bZIP (PF00170)	47 129	88 207	37.29	9.18
ClabZIP36	Cla022235	327	−	8	C	bZIP (PF00170) bZIP_C (PF12498)	161 230	214 273	35.94	5.52
ClabZIP37	Cla022315	158	+	8	H	bZIP (PF00170)	85	146	17.53	9.83
ClabZIP38	Cla022469	162	−	8	S	bZIP (PF00170)	30	87	18.3	5.21
ClabZIP39	Cla022580	448	+	8	A	bZIP (PF00170)	333	385	48.9	9.74
ClabZIP40	Cla022644	151	+	8	S	bZIP_2 (PF07716)	62	111	17.32	6.44
ClabZIP41	Cla015138	127	−	9	S	bZIP (PF00170)	1	42	15.01	11.1
ClabZIP42	Cla014803	936	+	9	C	bZIP (PF00170) bZIP_C (PF12498)	316 247	417 301	47.9	6.06
ClabZIP43	Cla015019	360	+	9	I	bZIP_2 (PF07716)	185	236	38.92	7.19
ClabZIP44	Cla016247	85	+	9	G	bZIP (PF00170)	14	76	10.01	9.79
ClabZIP45	Cla008839	405	−	10	G	MFMR (PF07777) bZIP (PF00170)	1 299	196 361	43.19	6.43
ClabZIP46	Cla008917	208	−	10	A	bZIP (PF00170)	138	188	22.86	9.88
ClabZIP47	Cla017361	144	+	10	S	bZIP_2 (PF07716)	21	73	15.89	9.42
ClabZIP48	Cla002873	467	+	10	D	DOG (PF14144) bZIP (PF00170)	180 263	222 341	51.57	8.41
ClabZIP49	Cla017444	767	+	10	B	bZIP (PF00170)	272	332	82.8	6.64
ClabZIP50	Cla017522	393	−	10	D	DOG (PF14144) bZIP (PF00170)	223 313	260 389	44.26	6.96
ClabZIP51	Cla017696	464	−	10	A	bZIP (PF00170)	358	410	50.01	8.98
ClabZIP52	Cla017709	525	−	10	J	bZIP (PF00170)	171	233	58.43	7.51
ClabZIP53	Cla011901	356	+	11	D	DOG (PF14144) bZIP (PF00170)	73 159	104 237	40.98	7.23
ClabZIP54	Cla022943	367	−	11	E	bZIP (PF00170)	238	284	41.29	8.77
ClabZIP55	Cla023140	156	−	11	S	bZIP (PF00170)	133	155	17.49	6.92
ClabZIP56	Cla023348	305	−	11	E	bZIP_2 (PF07716)	164	211	33.19	5.99
ClabZIP57	Cla023484	358	−	11	K	bZIP (PF00170)	188	232	39.13	4.49
ClabZIP58	Cla016491	184	−	11	S	bZIP (PF00170)	86	144	21.61	6.16
ClabZIP59	Cla016581	152	+	11	S	bZIP (PF00170)	26	84	16.76	5.79
ClabZIP60	Cla013375	236	+	2	E	bZIP_Maf (PF03131)	83	147	26.64	9.32
ClabZIP61	Cla018535	151	+	4	S	bZIP (PF00170)	113	141	17.6	7.09
ClabZIP62	Cla009927	385	+	5	D	DOG (PF14144) bZIP (PF00170)	98 181	140 259	43.54	8.97

### Phylogenetic characterization of watermelon *bZIP* gene family

**Figure 1:**
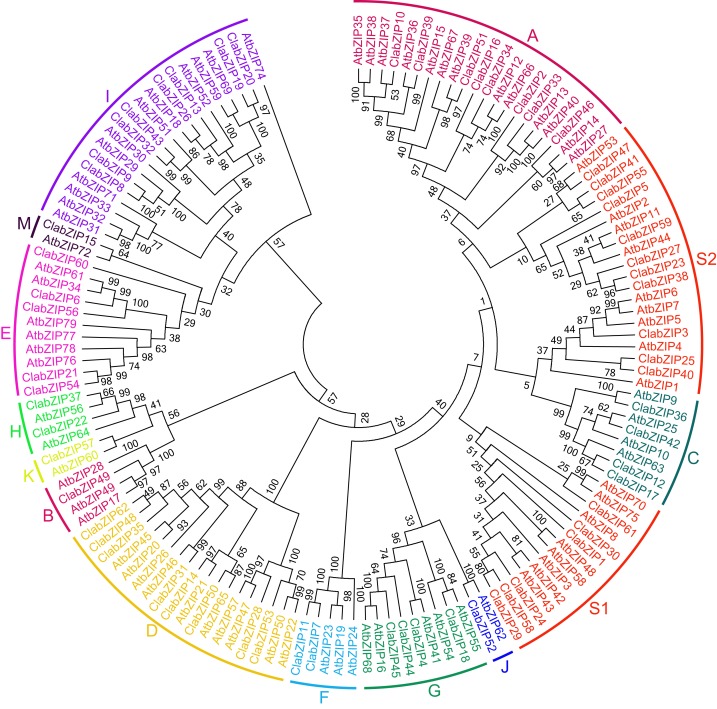
Phylogenetic relationships of watermelon and *Arabidopsis* bZIP proteins. The protein sequences of 62 watermelon ClabZIPs and 78 *Arabidopsis* AtbZIPs were aligned by MAFFT, and the phylogenetic tree was constructed by MEGA 7.0 using the NJ method with 1,000 bootstrap replicates.

A previous study has shown that the ClabZIP proteins can be phylogenetically divided into seven clusters (Unel et al., 2019). To further reveal the evolutionary relationships among the *ClabZIP* genes, a NJ phylogenetic tree was generated with the amino acid sequences of bZIP family proteins from watermelon and *Arabidopsis*. According to same classification criteria as in *Arabidopsis* (Dröge-Laser et al., 2018), the 62 ClabZIP proteins were classified into 13 different groups, namely A, B, C, D, E, F, G, H, I, J, K, M, and S (Fig. 1; Table 1). It should be noted that the groups comprising ClabZIPs with high sequence identity to AtbZIP60, AtbZIP62, and AtbZIP72 were named as U, V, and W in a previous study (Zhou et al., 2017), whereas these groups were named as K, J, and M in this study, respectively. These three groups in watermelon and *Arabidopsis* were the smallest groups, and each group only contained one member, while group S was the largest group with the maximum number of 17 ClabZIP members (Fig. 1; Table 1). In addition, two members in group J (AtbZIP62 and ClabZIP52) were clustered with AtbZIP1 and other group I members, and group S was separated by group F into S1 and S2 (Fig. 1). According to the phylogenetic results, four ClabZIP proteins containing bZIP and bZIP_C domains (ClabZIP12, ClabZIP17, ClabZIP36, and ClabZIP42) were clustered in group C. Meanwhile, eight ClabZIP proteins sharing bZIP and DOG domains fell into group D, and three ClabZIP proteins containing bZIP and MFMR domains (ClabZIP4, ClabZIP18, and ClabZIP45) were clustered together with ClabZIP44 in group G (Fig. 1). Interestingly, 11 ClabZIP proteins possessing bZIP_2 domain were scattered in groups of E, F, S, and I. In addition, five ClabZIPs were categorized into group E, including ClabZIP60, which contained the bZIP_Maf domain (Fig. 1).

### Conserved domain analysis of ClabZIP proteins

**Figure 2:**
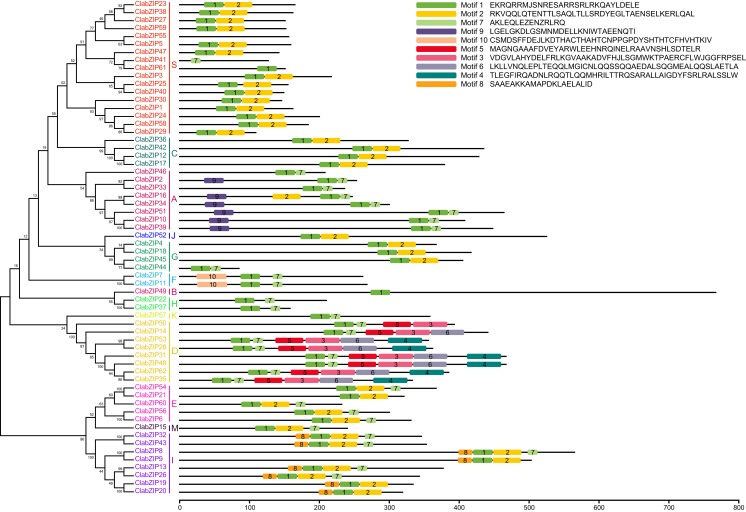
Conserved motif patterns of ClabZIP proteins based on their phylogenetic relationships. The NJ tree was constructed from the amino acid sequences of ClabZIPs using MAFFT and MEGA 7.0 with 1,000 bootstrap replications. The conserved motifs in the ClabZIP proteins were identified by MEME.

Identification of the conserved motifs of proteins could help to elucidate the protein functions, and plant bZIP proteins usually possess additional conserved motifs that might be involved in activating the functions of bZIP proteins (Jin, Xu & Liu, 2014). MEME online software was used to analyze the conserved motifs of ClabZIP proteins. As a result, 10 conserved motifs were identified (Fig. 2; Fig. S2). Amongst them, motifs 1, 2, and 7 were annotated as the bZIP domain, which was widely present in nearly all ClabZIP proteins, except for ClabZIP55, which had no motif. The bZIPs in group D, including ClabZIP28, -31, -35, -48, -53, and -62, which contained the bZIP and DOG domains, possessed six conserved motifs (motifs 1, 7, 5, 3, 6, and 4), while ClabZIP14 and ClabZIP50 also possessed the bZIP and DOG domains, but ClabZIP14 was lack of motif 4, and ClabZIP50 did not contain motif 6 and motif 4 (Fig. 2). Moreover, we found that though bZIP proteins within the same group generally shared similar motif compositions, those from different groups might possess specific conserved motifs, such as motif 9 in group A, motif 10 in group F, motifs 3, 4, 5, and 6 in group D, and motif 8 in group I (Fig. 2).

### Gene structure analysis of *ClabZIP* genes

**Figure 3:**
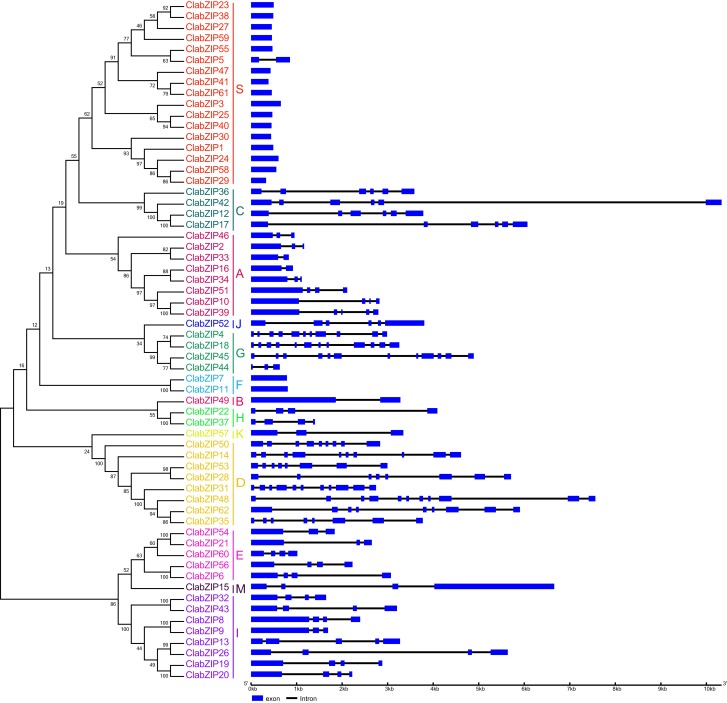
Exon-intron structures of *ClabZIP* genes based on their phylogenetic relationships. The exon-intron arrangement of *ClabZIP* genes was analyzed by GSDS. The exons and introns are presented by blue boxes and black lines, respectively.

The exon-intron profiles of 59 watermelon *bZIP* genes have been determined in a previous study (Unel et al., 2019). To gain further insights into the possible structural evolution of *ClabZIP* genes, the intron-exon structural patterns were investigated according to their phylogenetic relationships. The intron numbers of *ClabZIP* genes varied from 0 to 11, and the largest number of introns was found in *ClabZIP18* and *ClabZIP45* (Fig. 3). Most genes in the same group had conserved exon-intron structures. For example, with the exception of *ClabZIP5*, members of group S and group F had no intron, and all members of group C and group H contained 5 and 3 introns, respectively (Fig. 3). In addition, the intron number of *ClabZIP* genes varied greatly among different groups. For example, *ClabZIP* genes in groups A, B, E, K, M, H, and I contained 1–4 introns, whereas the members in groups C, J, D, and G possessed 5, 5, 7–10, and 10–11 introns (with the exception of *ClabZIP44*), respectively (Fig. 3), indicating that watermelon genome has undergone significant divergence during the long evolutionary history.

### Chromosomal locations and gene duplication of *ClabZIP* genes

**Figure 4:**
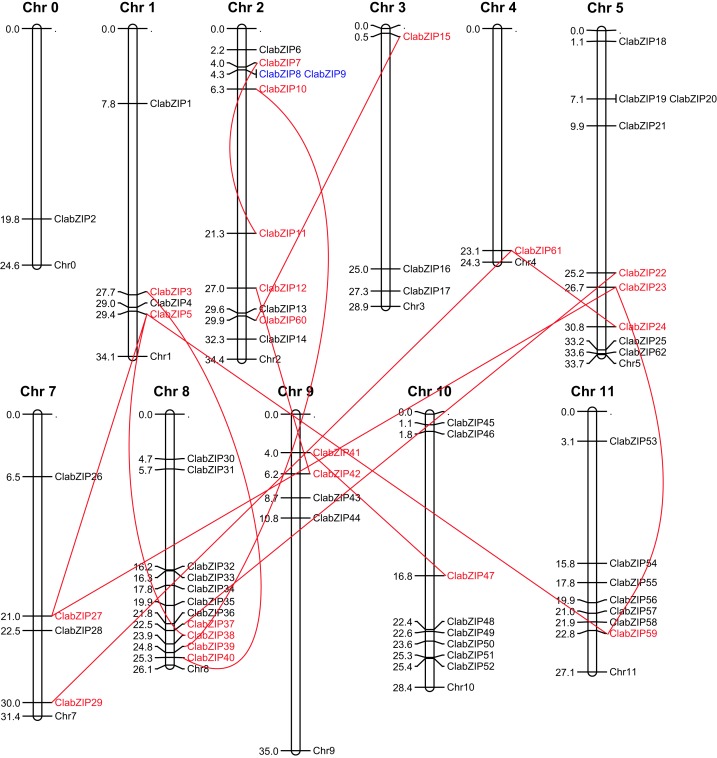
Chromosomal locations of watermelon *bZIP* genes. The vertical columns represent chromosomes with the gene names shown on the right. Genes located on the duplicated segmental regions have been joined by red lines. The segmental and tandemly duplicated genes are colored with red and blue, respectively.

The 62 *ClabZIP* genes were successfully mapped to 11 out of the 12 chromosomes in watermelon genome, with the exception of *ClabZIP2*, which was located in chromosome 0 (Fig. 4). For example, there were 11 genes on chromosome 8, followed by 10 on chromosome 2, 9 on chromosome 5, 8 on chromosome 10, 7 on chromosome 11, 4 on chromosomes 1, 7 and 9, and only 1 on chromosomes 0 and 4.

To further examine the evolution of *ClabZIP* genes, we investigated their genome duplication events, including tandem and segmental duplications, which contribute to the expansion of gene families throughout plant evolution (Cannon et al., 2004; Zhou et al., 2018b). As a result, one pair of tandem duplication (*ClabZIP8*/*ClabZIP9*) was identified on chromosome 2 (Fig. 4). In addition, 22 *ClabZIP* genes located on the duplicated segmental regions of watermelon chromosomes made up to 14 segmental duplication events (Fig. 4).

### Tissue-specific expression of selected *ClabZIP* genes in watermelon

**Figure 5:**
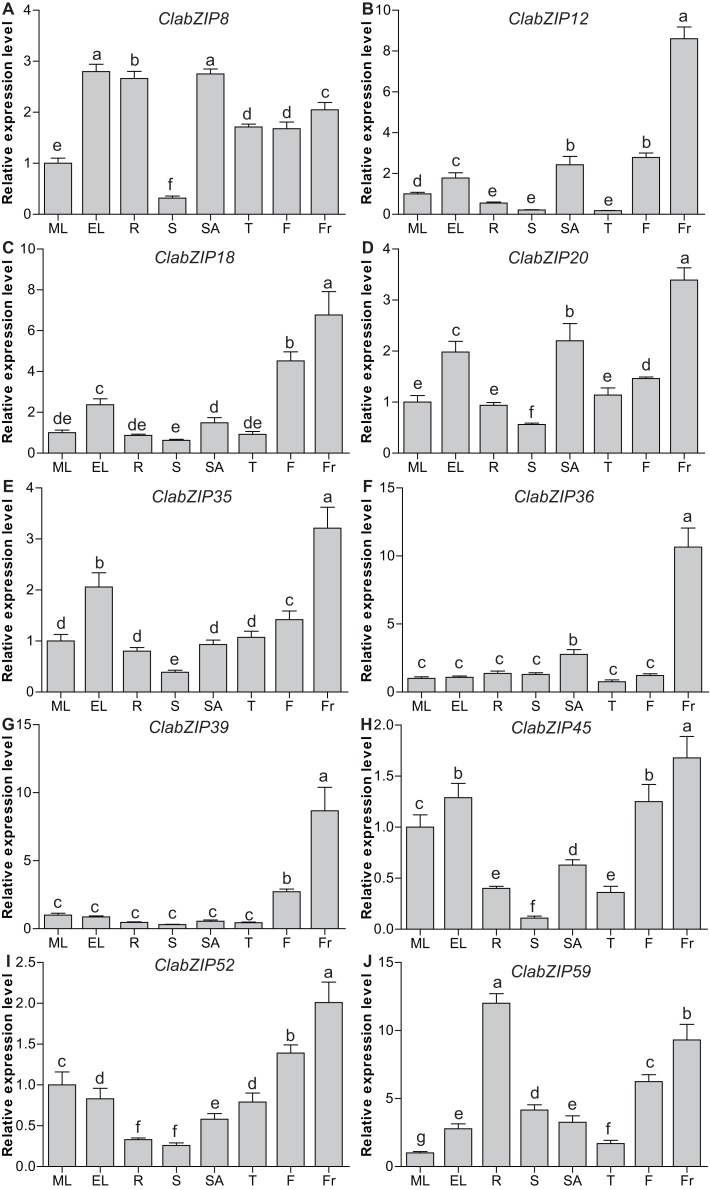
Tissue-specific expression patterns of 10 selected *ClabZIP* genes (A–J) in watermelon. ML, mature leaves; EL, expanding leaves; R, roots; S, stems; SA, stem apexes; T, tendrils; F, flowers; Fr, fruits. Three independent replicates were used, and error bars indicate standard deviation (SD). Different letters represent statistically significant differences (*P* < 0.05) based on Tukey’s test.

To further understand the tissue-specific expression of *ClabZIP* genes in watermelon, qRT-PCR analyses were carried out to examine the expression of 10 selected *ClabZIP* genes from seven different groups (one from each of groups S, A, J, and D, and two from each of groups C, G, and I) in various tissues, including mature leaves, expanding leaves, roots, stems, stem apexes, tendrils, flowers, and fruits. As a result, *ClabZIP* genes showed a broad spectrum of expression in the eight tested tissues. Among them, eight *ClabZIP* genes (*ClabZIP12*, *ClabZIP18*, *ClabZIP20*, *ClabZIP35*, *ClabZIP36*, *ClabZIP39*, *ClabZIP45*, and *ClabZIP52*) exhibited the highest expression in fruits, and *ClabZIP59* was found to be highly and preferentially expressed in roots (Fig. 5). Besides fruits, *ClabZIP35* had relatively high expression in expanding leaves; *ClabZIP18*, *ClabZIP39*, and *ClabZIP52* displayed higher expression in flowers; while *ClabZIP20* and *ClabZIP36* exhibited relatively higher expression in stem apexes than in other tissues. Notably, *ClabZIP8* had high transcript abundance in expanding leaves, roots, and stem apexes, moderate transcription in mature leaves, and the lowest expression in stems (Fig. 5). These results suggested that *ClabZIP* genes may be involved in diverse growth and development processes of watermelon.

### Roles of *ClabZIP* genes in melatonin induction of cold tolerance

**Figure 6:**
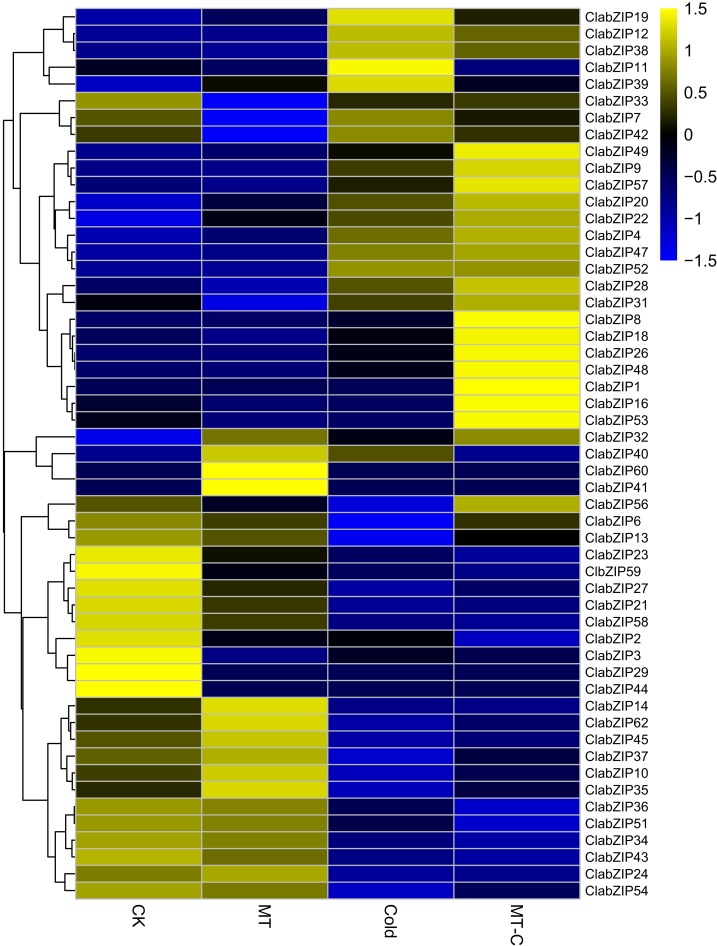
Cluster analysis of DEGs identified by transcriptome comparisons of melatonin (MT), cold, melatonin-cold (MT-C) treatments and control (CK). The log2-transformed FPKM values were used to create a heatmap depicting the expression of each *ClabZIP* gene.

To examine the effects of cold stress on the expression of *ClabZIP* genes, we determined the differentially expressed genes of *ClabZIP* genes under MT, cold, and melatonin-cold (MT-C) treatments based on the transcriptome data from a previous study (Li et al., 2016a), and the FPKM values of *ClabZIP* genes are presented in Table S5. As shown in Fig. 6, a total of 50 *ClabZIP* genes (23 up-regulated, 27 down-regulated) were differentially expressed in response to cold stress compared with the control (CK), suggesting that these genes might be involved in regulating the response of watermelon to cold stress. In addition, compared with cold treatment, MT-C treatment induced the expression levels of 31 *ClabZIP* genes, while significantly repressed the expression of 17 *ClabZIP* genes (Fig. 6), suggesting that MT could influence the expression of *ClabZIP* genes to regulate the cold response of watermelon.

**Figure 7:**
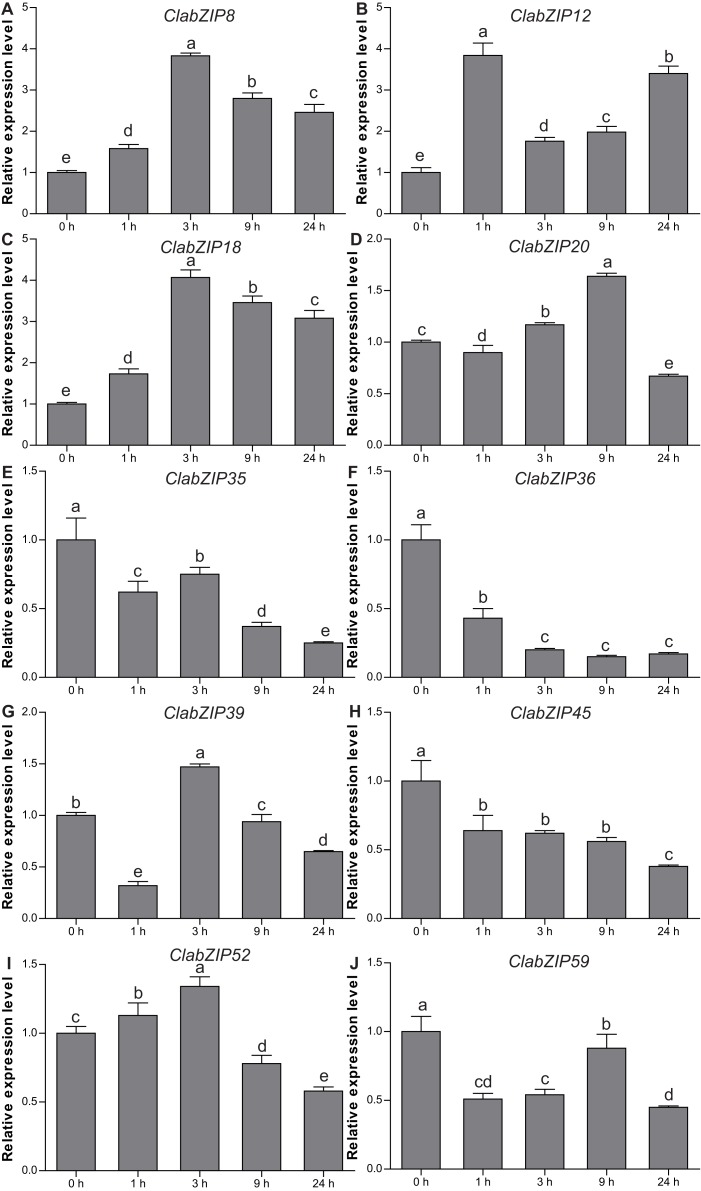
Relative transcript levels of 10 selected *ClabZIP* genes (A–J) in the leaves of watermelon under cold stress by qRT-PCR. Error bars were SD of three biological replicates, and different letters represent statistically significant differences (*P* < 0.05, Tukey’s test).

To further study the roles of *ClabZIP* genes in response to cold stress, 10 selected *ClabZIP* genes were examined by qRT-PCR to test the accuracy of the gene expression determined from transcriptome data. As shown in Fig. 7, the expression of *ClabZIP8*, *ClabZIP12*, and *ClabZIP18* sharply increased at certain time points, and reached the highest level at 3, 1, and 3 h, respectively. However, the transcript levels of *ClabZIP35*, *ClabZIP36*, *ClabZIP45*, and *ClabZIP59* were found to decrease at all-time points. Additionally, the expression of *ClabZIP39* was dramatically reduced at the early time point (1 h), and sharply up-regulated at 3 h, followed by gradual decreases at 9 and 24 h (Fig. 7). The changes in the expression of these genes were consistent with the transcriptome results.

### Roles of *ClabZIP* genes in red light-induced resistance against root-knot nematodes

**Figure 8:**
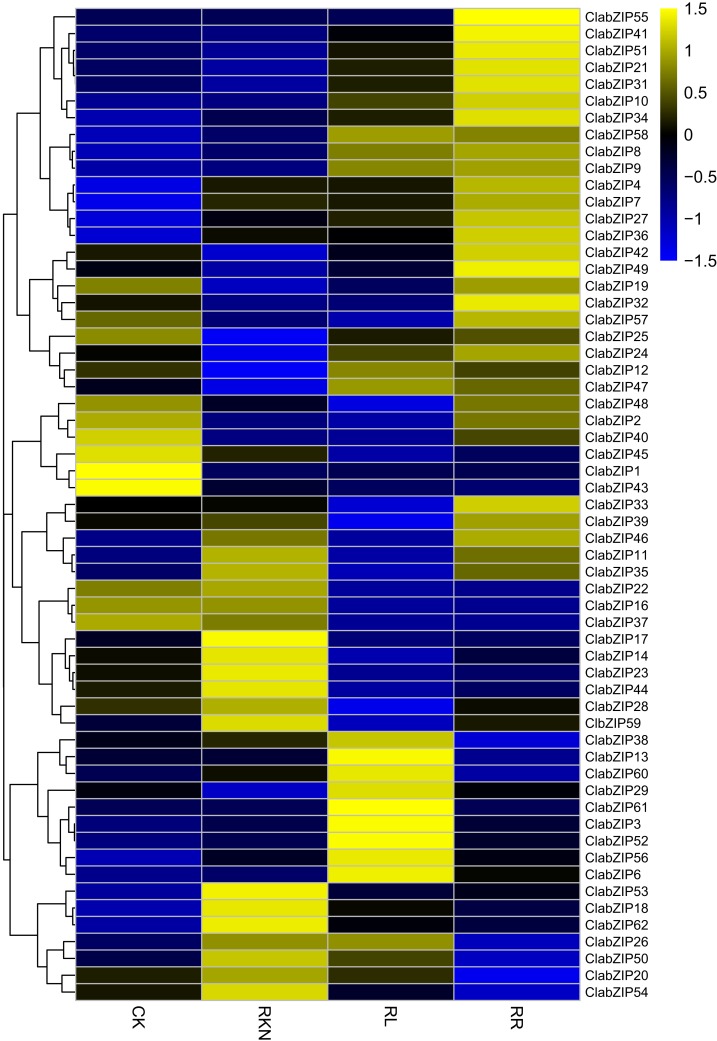
Cluster analysis of DEGs identified by transcriptome comparisons with inoculation of *M. incognita* under white light (RKN), red light and water control (RL), inoculation of *M. incognita* under red light (RR) and white light and clean water (CK) treatments in the leaves. The log2-transformed FPKM values were used to create a heatmap depicting the expression of each *ClabZIP* gene.

We also examined the expression of *ClabZIP* genes in the leaves and roots under the treatments of CK, RKN, RL, and RR, and the FPKM values of *ClabZIP* genes are presented in Table S5. In leaves, the expression of *ClabZIP* genes was significantly affected by RL, *M. incognita* infection and their interaction (Fig. 8). A total of 34 *ClabZIP* genes (such as *ClabZIP6* and *ClabZIP56*) showed up-regulated expression, while 23 *ClabZIP* genes (such as two *HY5*-like genes, *ClabZIP22* and *ClabZIP37*) were down-regulated under RL treatment compared with the control (CK) (Fig. 8). Compared with CK, a total of 34 and 23 *ClabZIP* genes were found to be up-regulated and down-regulated under RKN treatment, respectively. In addition, we also found that a total of 31 and 27 *ClabZIP* genes were respectively up-regulated and down-regulated by RR treatment compared with RKN treatment (Fig. 8).

**Figure 9:**
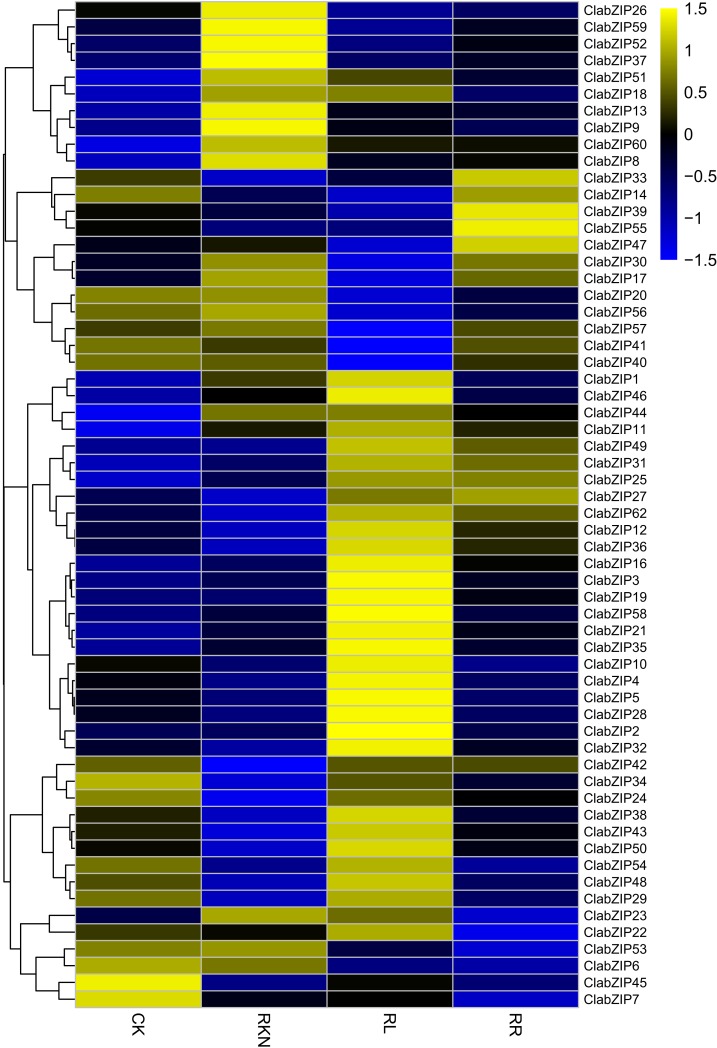
Cluster analysis of DEGs identified by transcriptome comparisons with inoculation of *M. incognita* under white light (RKN), red light and water control (RL), inoculation of *M. incognita* under red light (RR) and white light and clean water (CK) in the roots. The log2-transformed FPKM values were used to create a heatmap depicting the expression of each *ClabZIP* gene.

We also determined the expression levels of the *ClabZIP* genes in roots under the treatments of CK, RKN, RL, and RR (Table S5). As shown in Fig. 9, the expression of 60 *ClabZIP* genes (38 up-regulated and 22 down-regulated) was significantly altered by RL treatment compared with CK. Compared with CK, a total of 31 and 29 *ClabZIP* genes were respectively up-regulated and down-regulated by RKN treatment, respectively (Fig. 9). In addition, compared with RKN treatment, a total of 33 and 27 *ClabZIP* genes were found to be up-regulated and down-regulated under RR treatment, respectively.

**Figure 10:**
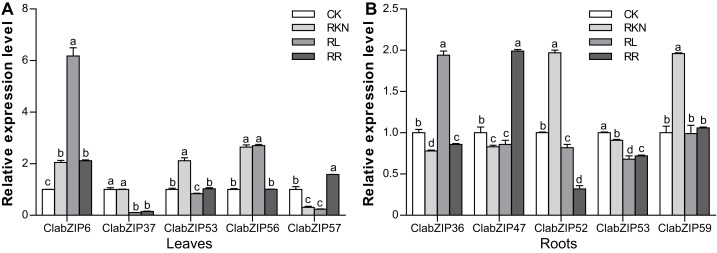
qRT-PCR analysis of the expression of selected *ClabZIP* genes under the treatments with inoculation of *M. incognita* under white light (RKN), red light and water control (RL), inoculation of *M. incognita* under red light (RR) and white light and clean water (CK) in the leaves (A) and roots (B) of watermelon plants. Error bars represent SD of three biological replicates, and different letters indicate statistically significant differences (*P* < 0.05, Tukey’s test).

We also randomly selected five each *ClabZIP* genes to examine their expression levels in the leaves and roots under the treatments of CK, RKN, RL, and RR using qRT-PCR. In leaves, *ClabZIP6* and *ClabZIP56* were strongly induced, while *ClabZIP37* and *ClabZIP57* were significantly repressed by RL treatment when compared with CK. In addition, *ClabZIP37*, *ClabZIP53*, and *ClabZIP56* were observably down-regulated by RR treatment compared with RKN treatment in leaves, while *ClabZIP57* was up-regulated (Fig. 10A). In roots, *ClabZIP36* was up-regulated by RL treatment, while the expression of other four selected *ClabZIP* genes was decreased (Fig. 10B). Additionally, *ClabZIP36* and *ClabZIP47* were up-regulated after RR treatment compared with RKN treatment in roots, while *ClabZIP52*, *ClabZIP53*, and *ClabZIP59* were suppressed. Furthermore, the expression of nine selected *ClabZIP* genes was significantly altered by RKN treatment (Fig. 10). In general, the qRT-PCR results were consistent with the transcriptome results.

## Discussion

In this study, a total of 62 *bZIP* genes were obtained from watermelon genome, among which three genes were novel (Table 1). The number is comparable to that of some dicot plants, such as cucumber (64 members) (Baloglu et al., 2014), tomato (69 members) (Li et al., 2015a), *Arabidopsis* (78 members) (Dröge-Laser et al., 2018), and cassava (77 members) (Hu et al., 2016c), but smaller than that of monocot plants, such as rice (89 members) (Nijhawan et al., 2008), barley (89 members) (Pourabed et al., 2015), and *Brachypodium distachyon* (96 members) (Liu & Chu, 2015). Previous reports have also shown that *bZIP* genes are associated with the evolution of plants, and eudicot *bZIP* genes have a lower frequency of evolution than those of monocots after divergence (Li et al., 2015b; Wang et al., 2011, 2017). In addition, 14 segmental duplication events were detected in watermelon genome (Fig. 4), indicating that the expansion of *bZIP* family in watermelon is mainly due to segmental duplication. Similar results have been reported in various plant species, including rice (Nijhawan et al., 2008), sorghum (Wang et al., 2011), maize (Wei et al., 2012), grape (Gao et al., 2014), tomato (Li et al., 2015a), and sesame (Wang et al., 2018b). Moreover, most *ClabZIP* genes are located on the upper and lower parts of watermelon chromosomes (Fig. 4), and similar results were also obtained in *Brassica oleracea* (Hwang et al., 2016) and apple (Li et al., 2016b), implying conversed locations of *bZIP* genes during the evolution of these plants.

The phylogenetic analysis results showed that the 62 ClabZIP proteins were clustered into 13 groups, including A, B, C, D, E, F, G, H, I, J, K, M, and S, which comprised 8, 1, 4, 8, 5, 2, 4, 2, 8, 1, 1, 1, and 17 ClabZIPs, respectively (Fig. 1; Table 1). Each group included at least one AtbZIP and one ClabZIP (Fig. 1), indicating that there is a similar evolutionary trajectory of *bZIP* genes in *Arabidopsis* and watermelon. Plant bZIP proteins usually possess additional conserved motifs that may be involved in activating their functions (Jin, Xu & Liu, 2014). In the present study, two types of additional domains, namely DOG and MFMR domains, were present in eight and three ClabZIP proteins (Table 1), which respectively fell into two groups (D and G) according to the phylogenetic analysis (Fig. 1), and some groups possessed specific sequence motifs corresponding to different protein domains (Fig. 2). These findings imply that different motifs outside the bZIP domain region might play different roles in determining the functions of bZIP proteins (Jin, Xu & Liu, 2014; Wang et al., 2018b). In addition, the *bZIP* members from the same group often exhibit similar exon-intron compositions (Fig. 3). This phenomenon is considered as an imprint of evolution in some gene families, resulting in the generation of functionally distinct paralogs (Li et al., 2015b; Liu et al., 2017). It is noteworthy that some *ClabZIP* genes, especially those in groups S and F, were prevalently lack of introns (Fig. 3), which could shorten the posttranscriptional process for immediate response to abiotic stresses (Zhou et al., 2018b). Similarly, a total of 25 *GmbZIP* genes belonging to group S were found to be intronless in soybean (Zhang et al., 2018). It is known that genes lacking introns would evolve faster than the rate of intron gain after gene duplication, and most members in groups D and G possessed more introns than those in other groups (Fig. 3). Therefore, it can be speculated that groups D and G might contain the original genes compared with other groups (Hu et al., 2016c). Moreover, some pairs of *ClabZIP* genes, such as *ClabZIP8*/*ClabZIP9* and *ClabZIP10*/*ClabZIP39*, which were distributed closely to each other based on the phylogenetic analysis results, shared similar exon-intron arrangements but different intron numbers (Fig. 3), suggesting that gain or loss of introns may occur in *ClabZIP* genes during the evolution of watermelon genome.

The bZIP TFs were shown to play important roles in various developmental processes throughout the plant life cycle (Wang et al., 2018b; Zhou et al., 2017). For example, there is evidence showing that *Arabidopsis* seed maturation can be regulated by multiple bZIP TFs including bZIP53, bZIP10, and bZIP25 (Alonso et al., 2009; Jain et al., 2017). In this study, all of the 10 selected *ClabZIP* genes were found to be highly and preferentially expressed in fruits (Fig. 5), indicating that they may play vital roles in fruit development. Similar findings were also obtained in other plant species, such as apple (Li et al., 2016b) and banana (Hu et al., 2016b). In addition, *ClabZIP8* and *ClabZIP59* were highly expressed in roots (Fig. 5), and *ClabZIP8*, *ClabZIP20*, *ClabZIP35*, and *ClabZIP45* also had relatively higher transcript abundance in expanding leaves, indicating their roles in leaf and/or root development. In *Arabidopsis*, *AtbZIP29* was found to participate in leaf and root development through regulating the genes involved in cell cycle and cell wall organization (Van Leene et al., 2016).

Accumulating evidence shows that many bZIP TFs are also involved in response to cold stress. For example, a large number of rice *bZIP* genes are regulated by cold stress, and several genes including *OsbZIP73* (Liu et al., 2018), *OsbZIP52/RISBZ5* (Liu, Wu & Wang, 2012), *OsbZIP38/LIP19* and *OsbZIP87/OBF1* (Shimizu et al., 2005) were identified as positive or negative regulators of response to cold stress. A previous study has shown that four *ClabZIP* genes (*ClabZIP3*, *ClabZIP6*, *ClabZIP23*, and *ClabZIP57*) can be regulated by cold stress (Li et al., 2017b). In this study, most of the *ClabZIP* genes were differentially expressed (21 up-regulated, 27 down-regulated) under cold stress based on the transcriptome data (Fig. 6), and qRT-PCR results revealed that the 10 selected *ClabZIP* genes were highly responsive to cold stress, which is in accordance with the results from the transcriptome data (Fig. 7). Similar results were also obtained in other plants. In Chinese cabbage, 36 and 17 of 136 *bZIP* genes were up-regulated and down-regulated after cold treatment, respectively (Hwang et al., 2014). Correspondingly, 12 and 28 of the 96 *Brachypodium distachyon bZIP* genes were found to be induced and suppressed under cold stress, respectively (Liu & Chu, 2015). A number of studies have indicated that cold stress can induce the endogenous MT level, and exogenous MT can enhance cold tolerance of various plant species, including *Arabidopsis* (Bajwa et al., 2014; Shi et al., 2015b), bermudagrass (Hu et al., 2016a; Shi et al., 2015a), rice (Han et al., 2017), melon (Zhang et al., 2017), watermelon (Li et al., 2016a, 2017b), and tea plant (Li et al., 2018). Besides, MT-induced enhancement of cold tolerance in plants is closely related to the up-regulated transcripts of numerous stress-responsive genes (Li et al., 2017b; Shi & Chan, 2014; Shi et al., 2015a, 2015b). In this study, among the cold-responsive *ClabZIP* genes, 31 genes had higher expression levels under MT-C treatment compared with under cold treatment (Fig. 6), implying the roles of them in the response of MT-pretreated plants to cold stress. It should be noted that the transcript levels of *ClabZIP6*, *ClabZIP13*, and *ClabZIP56* were significantly decreased by cold stress in control plants, while their expression was found to sharply increase in MT-pretreated plants under cold stress (Fig. 6), revealing that they might play essential roles in response to MT induction of cold tolerance of watermelon.

There has been increasing evidence suggesting that *bZIP* genes play important roles in controlling photomorphogenesis and light-regulated gene expression (Abbas et al., 2014; Banerjee & Roychoudhury, 2017; Nawkar et al., 2017). In this study, a number of *ClabZIP* genes were regulated by RL in leaves (34 up-regulated, 23 down-regulated) and roots (38 up-regulated, 22 down-regulated), with 23 and four genes being both up-regulated and down-regulated in leaves and roots, respectively (Figs. 8 and 9), revealing that RL could regulate the expression of these *ClabZIP* genes in watermelon. It is noteworthy that *ClabZIP6* and *ClabZIP56* were significantly up-regulated in leaves but down-regulated in roots by RL treatment (Figs. 8 and 9), revealing that *bZIP* genes are likely to participate in certain light-dependent biological processes in different tissues. In *Arabidopsis*, AtbZIP56/AtHY5 acts as an evolutionarily conserved regulator that participates in the concordance of light, environmental, hormonal, and developmental signaling pathways (Dröge-Laser et al., 2018; Gangappa & Botto, 2016). In addition, compared with RL, blue light stimulates much higher accumulations of AtHY5 and its closest homolog AtbZIP64/AtHYH (HY5-HOMOLOG) at both transcriptional and post-transcriptional levels, and thus regulates the pace of *Arabidopsis* circadian clock (Hajdu et al., 2018). AtHY5 could be regulated by FR light transmission from the shoot into the root and thus mediate the lateral root development (Van Gelderen et al., 2018). In apple, the expression of *MdHY5* was increased in response to light, and *MdHY5* could promote anthocyanin accumulation in response to light by regulating a number of TFs (An et al., 2017a; Liu et al., 2019). In this study, a *HY5*-like gene *ClabZIP37* was down-regulated by RL compared with the control in both leaves and roots (Figs. 8 and 9), suggesting that it may negatively affect the response to RL in watermelon. However, another *HY5*-like gene *ClabZIP22* was down-regulated in leaves but up-regulated in roots by RL compared with the control (Figs. 8 and 9), suggesting that the two *HY5*-like genes play different roles in response to RL in watermelon. Moreover, HY5 also plays important roles in regulating cold stress response. For example, *MdHY5* was shown to positively modulate plant cold tolerance through CBF-dependent and -independent pathways (An et al., 2017b). Tomato SlHY5 can improve cold tolerance by integrating temperature, photoperiod and light quality signals, as well as activate ABA biosynthesis and gibberellin (GA) deactivation (Wang et al., 2019). In this study, *ClabZIP22* was up-regulated but *ClabZIP37* was down-regulated by cold stress compared with CK (Fig. 6), implying their different roles in the crosstalk of cold stress and light signal transductions.

It is known that *bZIP* genes can regulate plant defense against pathogen infection (Noman et al., 2017). For example, a previous study has revealed that in potato, StbZIP61 functions together with StNPR3L to mediate the temporal activation of salicylic acid (SA) biosynthesis, contributing to SA-mediated immunity against *Phytophthora infestans* infection (Zhou et al., 2018a). Pepper CabZIP63 acts as a positive regulator of the defense responses to *Ralstonia solanacearum* by making a positive feedback loop with CaWRKY40 (Noman et al., 2019; Shen et al., 2016). Our previous reports also revealed that RL could influence the light-activated down-stream genes including SA and jasmonic acid (JA) pathway genes to induce watermelon resistance against nematode infection (Yang et al., 2015, 2018b). In the present study, some *ClabZIP* genes (such as *ClabZIP4* and *ClabZIP31*) were up-regulated, whereas *ClabZIP8*, *ClabZIP20*, and *ClabZIP50* were down-regulated under RR treatment compared with under RKN treatment in both leaves and roots, revealing their important roles in watermelon resistance against nematode infection. In the near future, it should be interesting to further clarify the functions of these *bZIP* genes in RL induction of plant defense against nematode infection.

## Conclusions

In conclusion, we performed a genome-wide identification of putative *ClabZIP* genes in watermelon, including their basic classification, phylogenetic relationship, conserved motifs, gene structures, and tissue-specific expression. In addition, transcriptome analysis revealed that some *ClabZIP* genes (such as *ClabZIP4* and *ClabZIP31*) may play crucial roles in protecting plants from nematode infection and cold stress. This comprehensive study could lay a solid foundation for revealing the roles of *bZIP* family genes in watermelon growth and stress response, which may contribute to the breeding of stress tolerant cultivars.

## Supplemental Information

10.7717/peerj.7878/supp-1 Supplemental Information 1 Gene ontology analysis of the *ClabZIP* genes.

10.7717/peerj.7878/supp-2 Supplemental Information 2 Distribution of amino acid positions of conserved motifs for watermelon ClabZIPs.

10.7717/peerj.7878/supp-3 Supplemental Information 3 The amino acid sequences of ClabZIP proteins.

10.7717/peerj.7878/supp-4 Supplemental Information 4 The amino acid sequences of AtbZIP proteins.

10.7717/peerj.7878/supp-5 Supplemental Information 5 The ****CDS sequences of *ClabZIP* genes.

10.7717/peerj.7878/supp-6 Supplemental Information 6 The gDNA sequences of *ClabZIP* genes.

10.7717/peerj.7878/supp-7 Supplemental Information 7 The FPKM values of *ClabZIP* genes extracted from the transcriptome data.

10.7717/peerj.7878/supp-8 Supplemental Information 8 Primers used for qRT-PCR in this study.

## Additional Information and Declarations

### Data Availability

The following information was supplied regarding data availability: The sequencing raw sequence data are available in the BIG Data Center GSA database, Beijing institute of Genomics (BIG): CRA001311 and CRA001312.
