# Supplementary figures and images for: The bZIP gene family in watermelon: genome-wide identification and expression analysis under cold stress and root-knot nematode infection
Source: PeerJ. 2019 Oct 16;7:e7878. doi: 10.7717/peerj.7878 (PMC6800529; doi:10.7717/peerj.7878)

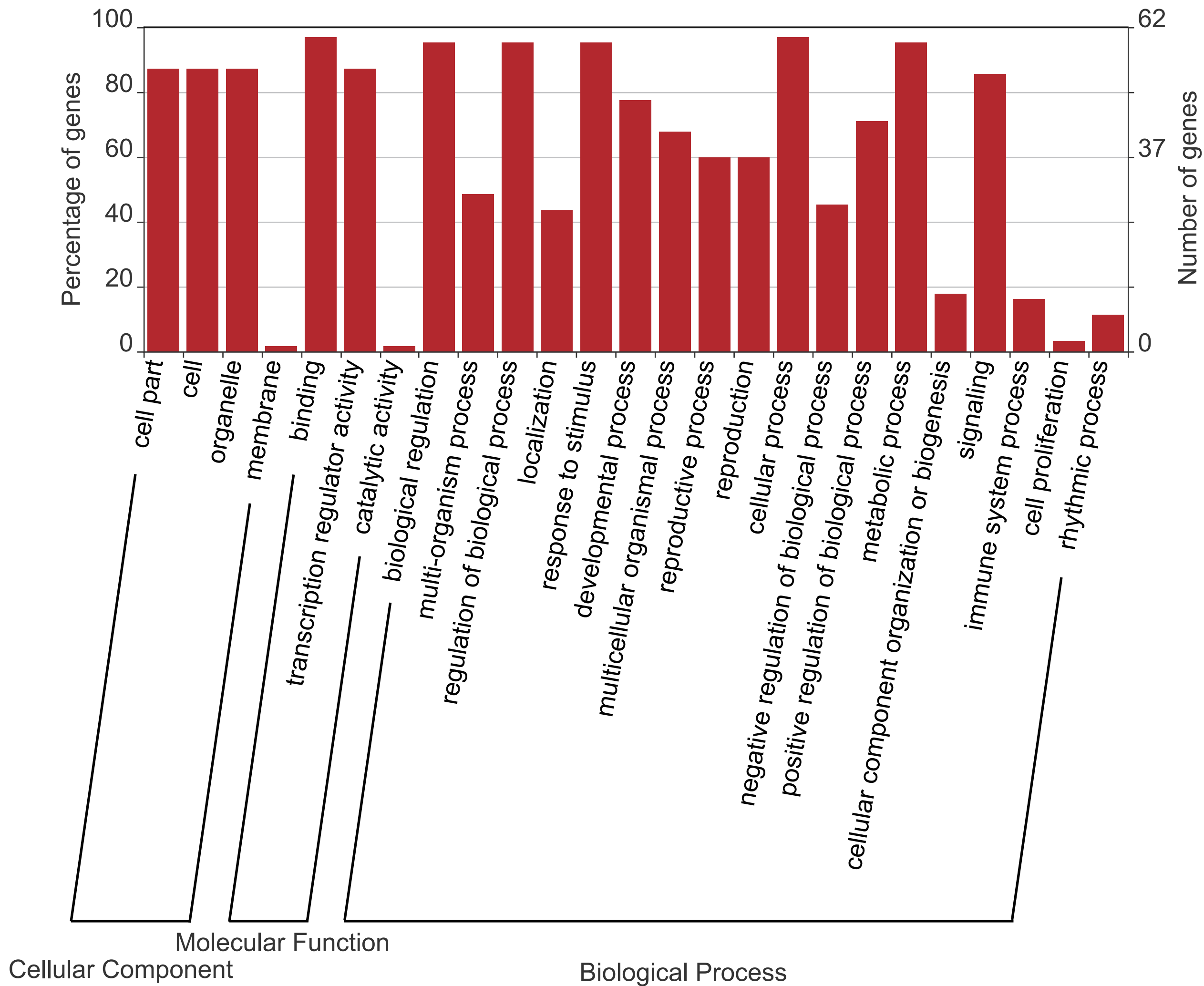

Supplement: Supplemental Information 1 [file peerj-07-7878-s001.pdf]

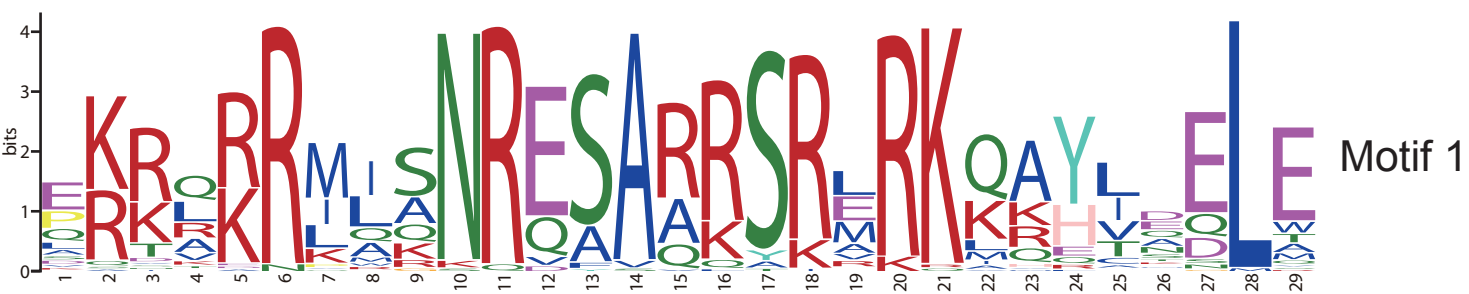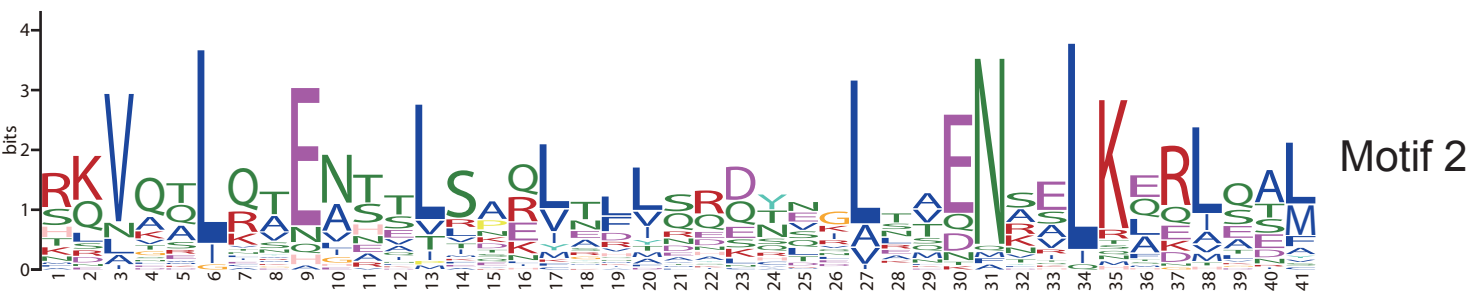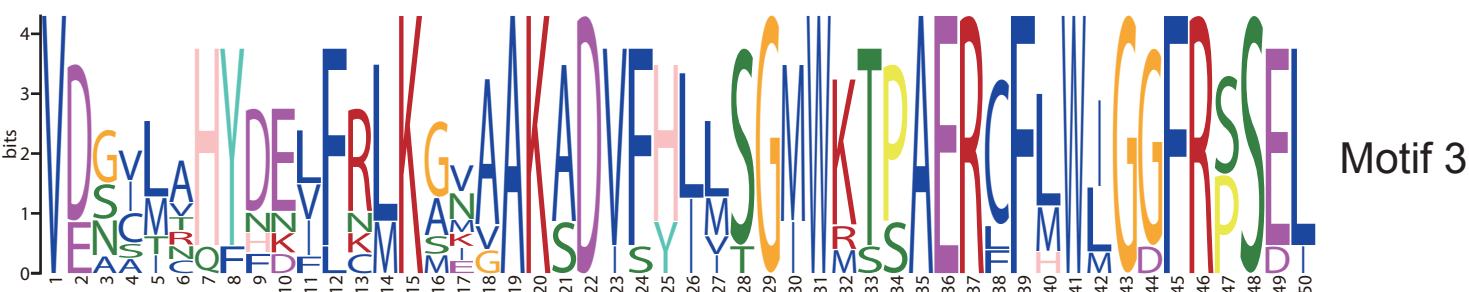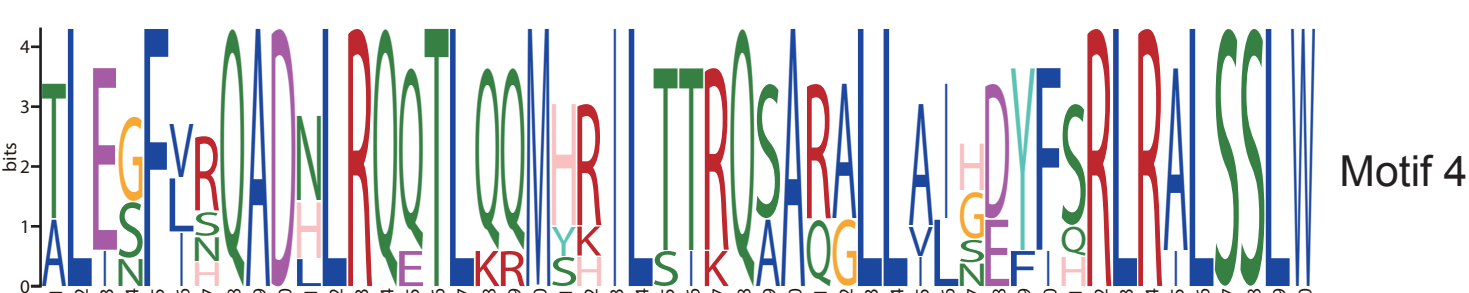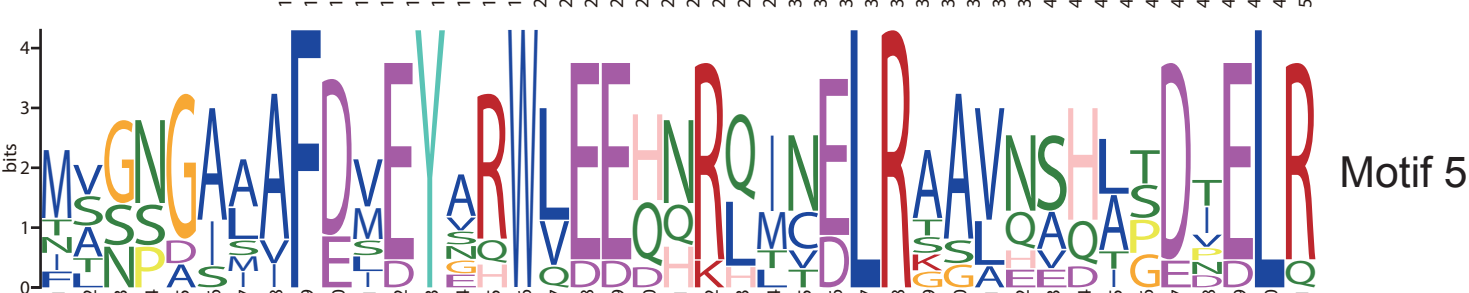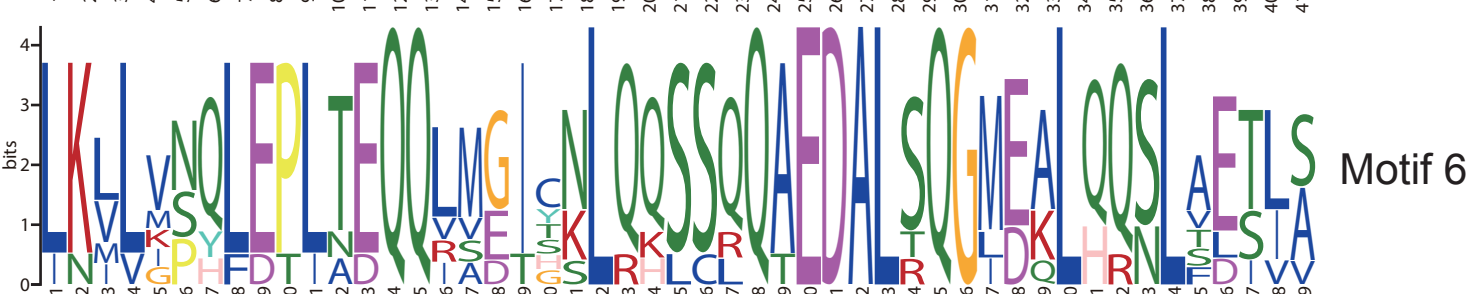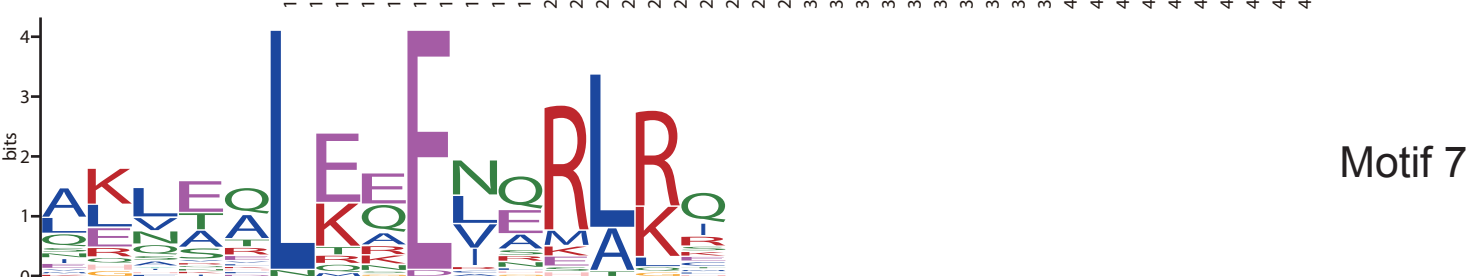

Supplement: Supplemental Information 2 [file peerj-07-7878-s002.pdf]
