# Supplementary material for: The bZIP gene family in watermelon: genome-wide identification and expression analysis under cold stress and root-knot nematode infection
Source: PeerJ. 2019 Oct 16;7:e7878. doi: 10.7717/peerj.7878 (PMC6800529; doi:10.7717/peerj.7878)
Supplement: Supplemental Information 3 [file peerj-07-7878-s003.doc]

**Table S1.** The amino acid sequences of ClabZIP proteins.

>ClabZIP2

MASSNVEMASTSKTNPDLPRQSSVCSISTIIADLHHTDPSRNLASMSMDDLLKNIYSDAQTHNQVQDENPIIGSSISPQDTFPLPKELSTRTVEEVWKEIVAGGDQQRRESATDQEITLEDFLSKAGAVCDDDVRVPVISEPGGFAVDSTLNNQLQIPSQQLEGPMVGGYASGIDGRIVGVGRGKRRAVEEPVDKATQQKQRRMIKNRESAARSRERKQAYTLELESLVTQLEQENARLLREEYWPLKEMSLK

>ClabZIP1

MEISGTHFAAPENYGMIQNLNINELLSYIPGNSTSTSDDGDDLNHKLGVVIDERKRRRMISNRESARRSRMRKQKHLDELWSLVVRLRMENHSLMEKLSKLTDSQQQLLQENVKLKEEASDFRRMITDIQMGSPYITHLRELEEAPCNTFDLMAESSSQSNE

>ClabZIP3

MLSTFPTASAYESMLENPFPAVNGTSTPWECHDHDHDPEFLPVVSQMTEPTGLFDVFHSPNPVISSSSSENRDEPEPMDAGPCAPDRKVEVIDERKRRRMESNRESARRSRMRKQKHLENLRNLVNKLKVENRELSNRLRFTVYEVNRVRTENDHLQTEHTILRQKLSYSRQVLIFRQFQRQLAFASPYSALEQPPTISQSQSQYILNNNNNNLINC

>ClabZIP4

MGTGEEGTPSKTSKPPSSSQEIAPTPSYPDWSSSMQAYYGAGATPPPFFASTVASPTPHPYLWGGQHPLMPPYGTPVPYPAIYPPGGVYAHPNITVTPGSVPINAEYEGKSPDGKERASKKSKGMSGNTASGGGRTGESGKVASSSGNDGASQSAESGTEGSSEGSDENANQQEFAANKKGSFNQMLADGANAQNNTGGPNGKSSVTGKPITSIPGTNLNMGMDLWNTTTAAPGAGKVRGNAVSSAIVTVPMVGRDGVMPEQWVQDERELKRQKRKQSNRESARRSRLRKQAECEELQARVQTLNNENRTLRDELQRLSEECEKLTSENTSIKEELTRFCGPEALANFEKGTTAPPAQSRGSGEGKD

>ClabZIP5

MASSNGASSGSANGSIRTSGSEDDREVERKRRRMQSNRESARRSRLRKQKHLHDLTHQVSQLKKHNNEMVTNMNVMANLCVNLEAENSILRAQMAELTHRLRSLNDVVMFINSMQVFETFLVDEVGDLNNGFEEEYHYNPWRYPFANYNSNTQIIAHKD

>ClabZIP48

MGSRTVKIGADDLNKTVNGMPSFVSSMPTANNSMGAEGSSIPSSRISDFRTLEQTLGFHIEDVDLTRNPLYNQIKSSSSTLNNIQFGSLIKPLASTDVNLPTAVMGSRTLPLQKESNPNLVSTSGGPRENWGESNMADTSTRTDTSTDDTDDKSQRLDKDQGNSLAAYDSSNKSKEKTADQKTLRRLAQNREAARKSRLRKKAYVQQLESSRLKLTQLEQELQRARQQGIFISNSGDQAHSMSGNGALAFDVEYSRWLEEHNRLMNELRAAVNSHAGDTELRTIVDNVTTQFDDIFRLKGIAAKADVFHILSGMWKTPAERCFLWIGGFRSSEILKLLVNQLEPLAEQQLMGICNLQQLSQQAEDALSQGMDALQQSLAETLASATPATSGSSGNVANYMGQMAMAMGKLGTLEGFLRQADNLRQQTLQQMHRILTTRQSARALLAINDYFSRLRALSSLWLARPRD

>ClabZIP45

MSGSEMEKPPKDKETKTPPPTTTTQEQTTTTSAGTVNPDWSGFQAYSPIPPHGFLASSPQAHPYMWGVQHIMPPYGTPPHPYVAMYPPGGIYAHPSIPPGSYPFSPFTMPSPNGITEASGNTAGSLEGDVKPPEVKEKLPIKRSKGSLGSLNMITGKNNELGKASGTSANGAYSKSAESASEGTSEGSDANSQNESQPKLGSRPDTLEVEVSQNGNSMHGTQNGGSNTQAMAAIPLATAGASGVVPGPTTNLNIGMDYWGASSTIPAIRGKVQSTPVAGGLVTAGSRDSIQSQLWLQDEREIKRQRRKQSNRESARRSRLRKQAECDELAHRAEALQEENASLRSEVNRIRSDYEQLLSENASLKERLGEVPGNEEVRAGRKGQRLSNETTQPTESEVVQVGNKN

>ClabZIP46

MEEVWKDINLSSLHSRSDHDFSAAAAAPATPIGLHHHSAAANFRHIILQDFLSSSTSKLDSSSAATAAAVAAPPPTVLSLNSTRELHFPDNAVAAAHFRHQDPASVSAAFHSPFDQVLGPPPFGKKRVPDSDSNNSGDRRQKRMIKNRESAARSRARKQAYTNELELEVAHLMEENARLRRQQEELRAAATAQVPRKHRLQRTSTAPF

>ClabZIP49

MADPIELVSPSDQNPNSTTYASEFDSLPIPPLDSLFFSDPNHDAPGDPFLYSTALDLGFEENDDFELTFDDLDDLYLPSEADDFLISDNLDQTTNSSNSPPDVPLLSDAGTRLSSPAGSPGSRSSSVSCEQSPGDCKSLNYQSSKLRTADSECFSTGSGGWDSKRSRMVNCPSPEDGAGGSDHEFSGGPASSQGSGSGVSEGTNYPSSNAECYDVISDQKIKSEEVGKNCITKRKKEQDEGNADSRSAKYRRSSVPAETTNPQLGSCSVNEDDEKRKARLMRNRESAQLSRQRKKHYVEELEDKVRNMHATIAELNSKVSYMMAENAGLRQQLSGSGMCQPPPPPPGMYPHPSMPPMPYTWLPCAPYVVKPQGSQVPLVPIPRLKPQQPAPVAKGKKNESKKAEGTTKKVASVSLLGLMLSFMLFGVLVPLANVRFENVGGGPGKLSFVGDRVYNSNRRRVLSVDGYSNLSDGENVGTPCGKSGTSNRLQCERNYRKGRDLKYNQRGKGSQRLNDSDESIKLGNASEALVASLYVPRNDKLVKIDGNLIIHSFLASEKAMASRRASDTNKARETGLAIPRDLSPALTVRNIRALTSGSEDHIKATAADGKLQQWFREGLAGPMLSSGLCTEVFQFDVSSTAPGAIIPASSIANTSRAHRKNGTHLNKAKNRRILGGLPVPLSGSNFNITEEPVRTPHKDNFPGNNKTASSMVVSVLIDPREAGDSEVDGVITQKSISRIFVVVLLDSVKYVTYSCVLPRSGPHLVST

>ClabZIP50

MASSNIKSTSIIQQANQQEEEKQQQQQLLLHHQQQQPQLLQTPSFVISSNSKNSSLFNFQNPNFPDHHHHHHQLPFSMNLPSIPSNFLSKDGGGDDLGELDQALFLYLDGQEPSTTTTQDQRQSLGMRPPTLNIFPSQPMHVDPLPIKGNTALINHSDSKIKASEPSRELANQRSSNGATASGPPPTQTTTTEPHPKPPKREPNKKGLTSSSEQEGPKTPDPKTLRRLAQNREAARKSRLRKKAYVQQLEMSRIKLTQLEQELQRARTQGMFLGGGAAILGPDQGLPSGFYNLSSDAAVFDIEYGRWQEEHHRLMCELRAAVQEQLPENELRLFVDSCLAHYDEVLNLKMMVAKSDVFHLVSGMWKTPAERCFMWMGDFRPSELIKVPFTIII

>ClabZIP51

MVVTESEMISHDEVESPLQSEQQLKHHGFSSLGRQSSIYSLTLDEFQHTLCESGKNFGSMNMDEFLTSIWTAEENQAINASQSGTSTVAAMAALSNAQAHLPVSGASMEKRNIEKQASLPRQGSLTLPAPLCRKTVDEVWSEIHKGQQGRNQNSNSGNANSQNPESATRQPTFGEMTLEDFLIKAGVVREPCAGAGVSQPLPPPQQYGMYQNSNHTIGAGYVSRPIMGLNTSAAGGGASNNPSAGGITTYQPVPQGGSTIGDTSGYVGNGKRNSVFSSQPPPAVCYGGRVVNGGGGGGGGGGYPPAQPMGLAAPVSPVSPEGMCTNQVDSSNQFGLDLGGLRGRKRIIDGPVEKVVERRQRRMIKNRESAARSRARKQAYTVELEAELNQLREENAHLKQALAELERKRKQQVPFPKQLARFCLIPNLIQFEAMYLEETKNVHTKAQRAKEKLRVMRRTLSCPL

>ClabZIP52

MASSSKCSDGTTCSGLSSSSSSSSSMSSSMAKAADQMVKVEIEAAEALAGLAVLAVRETGPQPSETKWGIKGKGKRARKEVKTESPTSAFADSLPSRADLNLRIEQEDRGVVRHQPLEKECTSQSHPEWETTGEMMKVDKEAESCKVSPACTTSYQLFGCRRSRRTLTEAEKEERRVRRILANRESARQTIRRRQALCEELTRKAADLAWENENLKREKELALKEYQSLETTNKELKEQLAEAVKPKVEEIPGNNRSSHVQMPPLPTNYPLFLFSRLPYFWPSVVQPTSPYHELPNVVVVPSSINLPANSNVSVSGSSHVQENFTNACGPRTPLCILPPCSWLLPHHDFRNQQSPQIWFPAGNNLEDIYSKSQNSANTSKVVCAESRQSSLPSAEEENDAPDLNEPPNLNEASNPKDHAQNSVGVSVDGFDTNARPQVREVLSPVRLECIESSSAVKQGNRSEDDHGLSSRTCDDLFDFAERRHKPEIAPCKKTIDAMAATEARRRRKELTKLKNLYARQCRMHS

>ClabZIP53

MSSSSTQLCASRMGIYEPFHQINSWANAFGSRLDTSISPIIKVDDCVDIKPEFVPFESMDHLESSQEMNKPIDDKVQRRLAQNREAARKSRMRKKVYVQQLETSRLKLRLLEQELERTKQQKGTSCLVDISHFGFSGFVNPGIAAFEMEYNHWVEEQQRQITELRKALQVHTTDIELQILVESSLNHYHNLFCMKANAAKADVFYLMSGIWRTSAERFFHWIGGFRPSELLNVLKPYFEPLNEQQRADIHKLQQSSRQAEDALTQGMEKLHQNLSLSIASDPIGSYISQMGDGMEKLEALESFVSQADHLRQQTLKRMSHILTTKQAAQGLLALGEYFHRLRILSSLWATRPREPA

>ClabZIP58

MQPGEVTSLQYLIPSNLSPYATHFPMAQNNLPTMQLNEFSNNEFSNPLYNFQGPSQVHDFNRHPCLSSNSTSDEADEQQQSLINERKQRRMISNRESARRSRMRKQKHLDELWSQVVWLRNENHQLIDKLNQVSDCHDKVVQENAQLKEQTSELRRMLTEVQVNSHYPNFRELEKIPPNTPSES

>ClabZIP59

MASSSGTSSTSSSMEEGELAALMEQRKRKRMISNRESARRSRMRKQKHLDDLMAMVGQLRKDNQQIVANLTVTTQHYAAVEAENSILKAQAAELSHRLQSLNEIVAFLNPSDGVLEDDTYGCDGAGGFFSPLQMAFYMSHPLTASADVFGEY

>ClabZIP54

MENSKVLSNMRNMIYSGKHALLPPKSPLPSGSSSYADYFPNPIIGSRAVQNPREGNVHHHRTSSESLVMEEQPSWLDDLLNEPETPVQRGGHRRSSSDSFAYLDAGNVSNENYTQDDSQCKNMYLPSWASQDFDSHQASFHMKASWIKQKNRTRELPPTTLTTNPGARSSAKSSILLESSRSLSTTPQEANGFSSTTEKQDSAETGLPDRKPSERMDSSHVKPGLADTDNKRAKQQFAQRSRVRKLQYIAELERNVQALQANGSEVSAELEFLSQQNLILGMENKALKQRLESLSQEQLIKYLEHEVLEREIGRLRMLYQQQQQPQPPPSSLKRTKSRDLETQFAKLSLRQKDARSGSESVAGPVQI

>ClabZIP55

MNLDELVFRNVMSVEEAELVHNPSSLSPAAATASLFLGKRNDDAEPQHDPMAEVSAPAEGMDWMHYQRAMLIDSKLPVSQAVYNHGSVPDIGVYNMQAMSITTSALSNSDFQEGNCGRKRRQLDDIKEKTIERRQRRMIKNRESAARSRARKQASS

>ClabZIP57

MEEDLHFSEYDLIGQIDWNDFFDEFPEVELPLAGDSTSPHGSPDSFSSWINHLENALLNDDQDKGVSLPTPTDDCCDSFLADVLVDTHGGASVIDLDSNASDCGNQFSSSQKEDGHKVSPAPTDDSCGSFMAEVLVDTHGSSSGVDAVVDVLSNASDCGDDSNNSQKEKIDATNIDDSVGEDVDDSLSKKRRRQLRNRDAAVRSRERKKMYVKDLEMKSKFLEGECRRLGRLLQCYCAENQALRFSLQMGGASGASLTKQESAVLLLESLLLGSLLWLVGTVCLFTLPQLPQSTLEPVPRVTMEDEGPGSAPLNENENKDSRYSYTSLQTRRCKAARTRMKPSMLDAMLGPSLALISV

>ClabZIP14

MGSQRVEETGPSSHYHHHLPYNSLLYGINNNNLSSTLINHDGQSFDFGELEQAIVLQGLGTKNKLDHHEPKQSFFSGKPAATLEMFPSWPIKHQQTSRGISLRRREESSDESGAAVNTHTINKSNNQEGHLELEIEKEESEMSKKGCCSSSSSGQDQSFIQKHLQQIQPEMILSDISTTALSSQHQSLQQKRKGCGSISTSQKQLDAKTLRRLAQNREAARKSRLRKKAYVQQLESSRIKLTQLEQDLQRARSQGLFLGAGGTGGGIVSPASAIFDMEYVRWLEEDHRHTVELRGGLEAHLPDVDLRVRVDACICHYNEIFRLKGEAAKSDIFHLITGIWMSPAERCFLWIGGFRPSDLIKMLMSQLDPITEQQVMEIYKLQHSSQQAEDALSQGLDQLHRSLTDIIAGGPIIDGINHMVLAMDKLSSLQGFLHQVIITFF

>ClabZIP13

MQDPAPSNPIHTPNSNQIPPLNVAAPPPKTHKPPPVANATASSSSMFANANTGNTSFMPRVGSHHRRAHSEVSFRLPEDMMDISALEEIGSEDDLFSTYIDVKKLGGNGGGNFVDHNGNGGSEGAGGSEGEKTSKPRHRHSVSVDGTTSSSSMFGEIMEAKKAMPPDKLAELWSSDPKRAKRILANRQSAARSKERKARYIQELERKVQTLQTEATTLSAQLTLFQRDTTGLSTENTELKLRLQAMEQQAQLRDALNEALKKEVERLKIATGEMMSPSESFNLGMHHMAYAPSSFIQLSQQQPGSVGLQNMQMPPYSHSPSNMTTHPLLPSESHSLSEVLQTDSLGRLQGLDISSKGSSLVKSEGPSLSASESSTTF

>ClabZIP10

MNFRNFEDMPPGEGTMAKAQGNFTLTRQPSIYSLTFDEFQNTWNGLGKDVGSMNMDELLKNIWTAEESQAITSTGAVAGGVGSTNAGNLQRQGSLTLPRTISQKTVDEVWKDLSKENTSVKEGNGIEAMPRRQPTLGEVTLEEFLARAGVVREEPPHIEERPFNCGFYGGLSREDNNAGLALGMFMGNQIAENKSMVSNQNQNSVFLGTGVVRSSQQQQQQQPLFPKPANVTFASSMNLVNNPQLTNGSGTNLVVAPKPPLHEALIQGSGIGAIGLGTRGVTVASRSPTSTISSDVITKSSIETSSFSPVPFSFGRGRRSSGALEKVVERRQRRMIKNRESAARSRARKQAYTLELEAEIAKLKEMNQELQKKQREIMETQKNQVLEKMKYQLGGKRLCLRRTLTGPW

>ClabZIP12

MDRVFPVGEISDHYWSSEPAAGPPPPPADEGSKMNRSASEWAFQRFLQEASETSPHSSAADHGEVVEIKDSAYNQLQKLNANQVGVSNCNTSSISSNAVPPNIPIDSEEYQAFLKSKLHLACAAVAMKRGSFRMTPASSTSADCGSQASNTLGIQAPKASNIGAGNNSLRSPDKDINGAAGVTSSSVVPKIPELRARPATSGSSRDLSDDEEIEGDTETNESKDPADVKRVRRMLSNRESARRSRRRKQAHLTELETQVAQLRVENSTLLKRLADISQKYNEANVDNRVLKANVETLRAKVKMAEETVKRVTGNPMFHAMSEISSIGISSFEGSPSDTSTDAAVPLQDDPCRHLYQSTSNNPMGPHDIVVNNGLANISQVGSGQQNSPSQVLPATSGNKTGRSESLQRVASLEHLQKRICGVKANAEQ

>ClabZIP15

MELCSVPVSTYPPITVGPDNRCLVNDSVRTTVSPDNRRLLKDSVTDWRRSFVVGCRREDEEGGRAVQLPPFSKIGGGEMNSGGDGAENVDGRNPEQILYGRNIEPNMDPRKLKRIMSNRVSAQKSRLKKVQYVADMERKLKALEAHIAVLSPQVELYRNQQQVLQMEQKRLNQKILNCSRNKLLRDAEIEENRAEVNRLRELHMKQQCEANGWDSNVFTMPPDLHASELSNIVSPQPTQT

>ClabZIP16

MGIQTMGSQLNGQQSHLQPAPLTRQNSWYGLTLDEVKNQLGEMGKPLGSMNLDELLHNIWTAEANQSMGMESESSSSVYSLKRQASFKLARALSGKTVDDVWREIQQGQKKKNHENLKSENSEIILGDMTLEDFLIQAGIYAEASPSPIMGLDAIDTMALAEKNFSQKMGLLSSSPSLGTLSDTTTPKRRRDPSDTLEKTMERRLKRKIKNRESAARSRARKQAYHNELVNKVSRLEEENLKLKKEK

>ClabZIP17

MIDRVFSVDGISDHFWTSPEESSKLNRSASEWSFRRFLQEAASVSDSSISPPPASPSAAEIRSNAVEIREKQSNQNVGVMKEREICSSSAREKSRAAAAADSDEYQAFLKSKLNLACAAVALCRGSFMKTQDSCASSTQAGASHLTSQSSSKGISCSPCVQKRAGILVSSANISSSREQTDEDDDVEGENNMNEQIDPASAKRVRRMLSNRESARRSRKRKQAHLTELETQVAELRVENSALLKRFGDISQKYNEAAVNNRVLKADLETLRAKVQMAEETVKRITGMKSMVHAMSEVSSISIHSFEGSPSEISTDAPNNHIADISSANIQKNSLEMATVSRNKMARTASMRRVASLEHLQKRIRGSSSSCHPSGKGDQQ

>ClabZIP61

MEMNEDLGFGENPFNGSLKRHCSSHLIMETNRMMRREGDDHEDESNGCCGFHREILFPTMITTTGQAPTTTNNNNCNVFSPNSASCYSNDNILDVVEVLDIHRHHHLTVMAERKLRRMISNRESARRSRMRKKKQIEELQYQARFSNIYLP

>ClabZIP21

MANSKGSSNVRSFMSSGKHALLPPKSPFPSVSPSYTEYVPNSVIGAKAVQRPRDGNSYHQRTSSESILIEEQPSWLDDLLNEPETPVRRVGHRRSSSDSFAYTDAANVNFDSIMQEEFRYANAIPGHSWLSQEFDHQRDARHVSFYTEANIAKQKNRVWESSLSTMNNPIALHSPRENIGIHTSGPLNTPQEADGLPSTASEKQDSTESGSHDPKVSSDRKDASHGKSSVSDTENKRAKQQFAQRSRVRKLQYIAELERKVQALQAEGSEVSAELEFLNQQNLILSMENKALKQRLENLAQEQLIKYCKLLFPRYLAFFVC

>ClabZIP62

MVTEGEWTRQVEKEMRRKWQTGCQVSLQRCPFVTNLMPLVSLPCSITENLKVYAMTDVSPRTDISTDVDTDEKNQRLDMLQRNVVASDSSDRTKDKSDQKTLRRLAQNREAARKSRLRKKAYVQQLESSRLKLTQLEQELQRARQQGIFISSSGDQAHSMAGNGAIAFDVEYARWLEEQNKQINELRAAVNSHASDTELRMIVDGILAHYDEVFRLKGVAAKADVFHLLSGMWKTPAERCFLWLGGFRSSELLKLLVNQLEPLTEQQLMGISNLQQSSQQTEDALSQGMEALQQSLAETLSSGSLGSSNMSGNVANYMGQMATAMGKLGTLEGFIRQADNLRQQTLQQMHRILTIRQSARALLAIHDYFSRLRALSSLWLARPKE

>ClabZIP25

MFYSEEEEVVGFPGPAQEPRFTPAKIQELWSLLEDPTRSSSGSQGSCQAVSLIDEERRRKRMISNRESARRSRLRKKRHLENLAVQTDRLKMKNQELKRQLNLVVNRCYMVRRQNERLWSEFVALHARLSDLYRISVPMQEKENSCMQISFNYFS

>ClabZIP24

MLPGEMTGIQFFPSENSFQIPSNIGMMQNSFQTLHHFNSFLGNLPMSHVPHPSHEFLAQSSSFSYNSTSDDAEEQQKSIIDERKQRRMISNRESARRSRMRKQKHLDELWSQVLRLRTENHKLIDKLNHVSDSHEKVLLENARLKEEASDLRQMLSDLQIGSPYTPCLSNLEDIPCNSAHLRAESSSCQSIANSIDNLLH

>ClabZIP23

MIMDCSSGNSSGSLSQIVLQNQSSGSEEELRQLMDQRKRKRMQSNRESARRSRMRKQQHLDGLMAQVSQLRDNKNQMISRINLTTQLFLNIEAENSVLRAQILELTHRLESLNQILSHINDNDDEEQHNFLQNFDDFDHNPLFINSFFITQQPIVASAHHHLLHY

>ClabZIP22

MQERAAAAAAGRLRSSSERSSSSAFQLDVKEGAESDEEEISREPQICGNSVSAVGISASGKAPASDSIRSRGRSAAEKESKRLKRLLRNRVSAQQARERKKAYLSELESRATNLEKRNSELEEKLSTLQNENQMLRHSRPTPLAVVRSEFQPLSSAGGRFFVHRRLSLSCWQLCLILVLPPPPLDDLLPWVWFLPCHPPLFTVARKGNLY

>ClabZIP18

MGTSEEAKSVKTEKPSSPTPDQNSVPNSASIHVFPDWAAMQAYYGPRVAVPPYYNSAVASGHAPHPYMWAPPQMIPPYGTPYAAIYSHGGVYAHPAVSMGPHSHGPGVPSSPGAAPPLSIETPSKVSGNSSQGLMKKLKGFDGLAMSIGNVSTETAEGGAEHGQSESMETEGSSDGSDGTTAGANQTKRKRSREGTPTTGKDAKIEPQASPVTAAEMNESSSKLLGTTKAANATGKLGSVISPGMSTALELRNPSSINAMTSPTTVPPCSVLPSEVWLQNEKELKRERRKQSNRESARRSRLRKQAETEELARKVDSLTAENVAIRSEISRLSENSEKLKKENSTLMEKLKSAQSGRTEALDMNEKRMQQPVSTETKGPVNKSINEESIICKKNSSSGAKLRQLLDTSPRADAVAAS

>ClabZIP19

MEKDKPQGYSGGFPTPSSRYSVLSPTGSIFNGKSEATSSSMLLPPMPSGALSDSGQFGRERPTDSNRFSHDISQMPDNPPRNIGHRRAHSEILTLPDDICFDNDLGVIGSADGPSFSDDTEEDLLSMYLDMDKFNSSTATSAIQVGESSSAVGDAISTPTPAIGAATSKDDTAVGLKERPRVRHQHSQSMDGLTTIKPEMLVSGSEEASAADSKKAMSATKLAELALIDPKRAKRIWANRQSAARSKERKMRYIAELERKVQTLQTEATSLSAQLTLLQRDTNGLSAENNELKLRLQTMEQQVHLQDGPFWKMILFAFLAFIDIVTLKVALVSN

>ClabZIP20

MDKEKPQCHGGGFLHQSSRYSGFSSAETSFNGKSEATSSSMSFPPLAPSTNSDWAQSGRGMSTDSARFSHDISRMPENPRRNVGHRRAHSEILTLPDDICFDSDLGIIGGADGPSLSDDTEEDLLSMYLDMDKFNSSTATSATQVGDSSSPLVEAAATSTDDIAVGLKERPRVRHQHSQSMDGLTNIKPEMLVSGSDEASAADTKKAMSAAKLAELALIDPKRAKRIWANRQSAARSKERKMRYIAELERKVQTLQTEATSLSTQLTLLQRDANGITAENSELKLRLQTMEQQVHLQDGAFTVSGPSGLGTGMIQLDMH

>ClabZIP29

MNPPSLSSNSTSDEAEDQQLSLINERKQRRMISNRESARRSRMRKQKHLDELWSQVLWLRNENHQLIDKLNQVSECHDRALQENAQLKEEASELRQMLTDFQLHNPYLP

>ClabZIP28

MNSSSTKFYNPGRMGLYEPLHHIGMWGETFRTSANLDAQSSFIIEADTKLETQSDDASLGSLGDPHVYDQDDTKRIDKIQRRLAQNREAARKSRLRKKAYIKQLETSRVKLIQLEQELEKARQQGLLAGSRFDNNQLGLSGTTNSGISAFESEYEQWVEEQNRQICDLRTAVHADITDIELRILVENAMRHYFKFFRMKAKAAKADVSYIMSGMWKTSAERLFLWIGGFRPSELLKVLIPQLETLTEQQISETGSLRKSCLQAEDALRQGMEKLQQNLFESVVAGQLGEGSHPLQMTAAMERLEALISFVNQADHLRQETLQQMYKILSTRQSAQGLLILGEFFQRLRALSSLWANRPCEPA

>ClabZIP27

MASPVGSSSGSPSSDEDLRQIVDQRKRKRMISNRESARRSRMRKQKQLDDLTSQVGQIRTENEQIAVNINFTNQLYVNLEAENSVLRAQMVELRHRLDSLNEIISFMNSSTRNLFDSEDHYEAPGIDGFVDSWGFPFLNQPIMAAGDLFMC

>ClabZIP30

MDLPSEIPEETQLLLGAEEKYFWTIERNDLQNMISSDQFCRPTSNYNNNNNRRSAEIMVDERKQRRMISNRESARRSRMRKQKHVKELWSQLAQLCTQNHDLEEKLRHLMESQQRLLQENASLKQQASAFRQILRDMELEQLVTQF

>ClabZIP31

MQSFENPEQFYAHSSSSSSIFLRGDDSGRFHTRFLPDIEELQQSAAAIAAFHQDDAVDLSPSSVFSLKSTHNTAFPIYLPYGNTDVGSIGRTGYLDTGQELMRLKRLAPPQSVAVTVAASSSLGNGSFENWGESAMADNSQQTDTSTDIDTDERNQFQGAAHGALMAVDSMDQSKPKSADQKTLRRLAQNREAARKSRLRKKAYVQQLENSRQRLAQLEQELHRARQQGIFVASGAGDHCASMAGNGALAFDLDYARWLDEHQRLINDLRASANSQLGDDELRFLVDGVMAHYDELFKLKSVGAKADVFHILSGMWKTPAERCFMWLGGFRSSELLKIVGSHLEPLTDQQLMGICNLQQSSQQAEDALSQGIEALQQSLVETLSTASLGPASSGNVADYMGQMAIAMSKLTTLENFLHQADLLRQQTLQQMHRILTTRQAARALLVISDYISRLRALSSLWLARPRD

>ClabZIP34

MGSQAGGDPNGKQSQFQPLVRQNSLYSLTLDEVQNQLGDLGKPLISMNLDELLKNVWTAEANQTVGKDNEDNNVLANQTSLQRQASLSLNGALSKKTVDEVWRDIQQSKDSEEKKSRERQPTLGEMTLEDFLVKAGVVAETSSNKKGAGPVVEIDANITPQFQQTQWMPYPQPQYQSQQAAMMGVYMSGQPIPQPLHVGGGAVMDVPYVDNQLALPTPLMGALSDTQTSGRKRGAPEDMIEKTVERRQKRMIKNRESAARSRARKQAYTNELENKVSRLEEENERLRKRKLNGLTFVTSH

>ClabZIP33

MTVDGLLRNGYNSNPTESSILLDAQITLVDSPNPSSLPMNTTTTTTTNSSAVIDSNHNSSSGAPPPKTVDDVWREIVSGERKELKEEVADEMITLEDFLLKSGAVPVEDVKLPQTERLSGGVFSFDPIPSTTFQALDKVEGSIIGFANGVDLIGSGGSGGRGKRGRAALEPLDKAAEQRQRRMIKNRESAARSRERKQAYQVELESLAVRLEEENERLLREKVFLFSFDIHMRTSF

>ClabZIP35

MATEGSPRTDISTDGDTDEKSRRPDRGQLTLTMASDSSDRSKDKTDQKTLRRLAQNREAARKSRLRKKAYVQQLESSRLKLTQLEQELQRARQQGIFISSSGDQTHSMSGNGAMAFDVEYARWLEDHNRQLNELRSAVNSHASDPELRIVVDGILVHYDELFRLKGNAAKADVFHLLSGMWKTPAERCFLWLGGFRSSELLKLLVNQLEPLTEQQLVGITNLQQSSQQAEDALSQGMEALQQSLAETLSSGALGSSGSSGNVANYMGQMAMAMGKLGTLEGFIRQADNLRQQTLQQMHRILTTRQSARALLAIHDYFSRLRALSSLWLARPRE

>ClabZIP36

MKRSASELALEEFLKKAAIISPDDDVIDPDDEDVFKIEQQQIIRSPKRGRNFQDSADATCFFGDIDFSCFLVKNNREIMDAIVNCGGGLAEAPLWSQNLTPKHSSFSATIDSQSSIVSSPTSASNLMGREHQRGNNSGSSEDQSDDEIEAGSCEQSTDPLALKRIRRMISNRDSARRSRRRKQAHLAELENQVKQLKGENETLFNQLLDASQQYRDANTNNRVLKSDVDALRAKVKLAEDTLARGSMTCSLNQLLQSHLSTPQPLTALRRMSNVAPPLGLPGDEVSYSCVTMSGQNPTVGLPNSDMHMKTGMGSEAVSCVSGIWPRN

>ClabZIP37

MQEQATSSAAASSLPSSSERSSSSALHLEVKEGMESDEEIRRVPEIGGESAGTSASGRDTGSVAGPDRVQVSREGQRKRGRSPADKESKRLKRLLRNRVSAQQARERKKAYLNDLEIRVKDLEKKNSELEERLSTLQNENQMLRQILKNTTASRRSGE

>ClabZIP38

MDSASGNSSGSTRLQNSGSEEDLQVLMDQRKRKRMQSNRESARRSRMRKQQHLDELMAQVTQLRKDNAQILSNINITSQLYMNIEADNSVLRAQMAELTQRLQSLEEIANCINTGSSNGGFGETEEEAFQIQTNVAADSFMNSMNFLYVNQPIMAAADIFHY

>ClabZIP39

MNFKDFGNDPSAGNGGGGGRPPANYPLARQSSIYSLTFDEFQSMGSIGKDFGSMNMDELLKNIWSAEEMQTMASSAAAVDKEGAGSVGRSGGYLQRQGSLTLPRTLSQKKVDEVWKDIINEHASAKDGATVASNLQQRQQTLGEMTLEEFLFRAGVVREDTQVTANPNNGGFFGNNTGFGIGFQRQAKVPENNNHIPIQSSNLSLNVNGVRTHQPQPIFPKQPAVTYGSQLALPSDGQLASPGIRGGIMGIGDQGLNTNLMQGSALQGGRMGVVNIAAAPLPIATESPRDQLSSDGIGKSNGDTSSVSPVPYALNGGIRGRRSNGIVDKVVERRQRRMIKNRESAARSRARKQAYTMELEAEVAKLKEENQELRQKQAEIMEMQKNRALEVMDKQQGIKKRCLRRTQTGPWCRVAYVSRRLACRVSYNVFASKWIIKSSVAFEWKQNT

>ClabZIP42

MHTFFSSEDLSDNSFWPPQPAASSSPPSHSPFRSPDPSLTMNRSASEWAFERFLEEVSALPVNSCPSTTSDRVPVSPVDVASPASQSSTSKRDEGDDEIVEIKKADCDHDRSQPIPSSDPSKMVRSSSDRYRVFLKNQLDMACAAVALSRAASLEPKGPVQPADHRGQSSNAFQFGMQAPGQGSDRGTSTKESEVSGSPLGIPSLPTMPKKLGVQPAQTTSGSSRDESDDDDLEGDIENIENMDPADARRARRMLSNRESARRSRRRKQAHLNELETQVGQLRVEHSTLLKRLTDVNQKYDDAAVDNRILKADIETLRAKVKMAEETVKRVTGVNPLLVAMSQTQMPFVNNQMPMQSNTQFFHQNMSAFANSPPHHHNLEPPPIPHVGRSQNDVATKISDMPSVHIDHVQKQAMHGPLSAWDAEPPHSTPNHKKN

>ClabZIP41

MQSNRESARRSRMKKQKQFQDLTSEVRRLQIVNSRIVESVNSREQARIEIETMNNLLRVEAMETTYRLKALDLVLQIVDKANALAVGVRDPLLEPWQLTSKGSCRRRRRITIRFFFNAVLFLTCRIF

>ClabZIP44

MAMISSAFSHTKDERELKRQRRKQSNRESARRSRLRKQAECDELSQRAEALKEENASLRSEVDRIRSEYEQLLSENASLKVMPKT

>ClabZIP47

MASIPRQTSSGSNGGSPSALPDERKRKRMQSNRESARRSRMRKQKQLEDL

AGEVSRLQIANNQLVQSIGAKEQAFVQVDNMNNVLRAQAMELTDRLRSLN

SVLHIVEEVSGLAMDIPEIPDPLLKPWELSRPVLPVADMFLC

>ClabZIP56

MPNLTPSPPPPPPPSATHHHNSSWVDDFLDFSTARRGLHRRSISDSIAFL

ETPFSDQCRNSALLHPPPPFDRLDDDQLMSMFTDDISIPIPASPSNPSTP

SDQNSNNDDKNPTEDAMFPPPPPPPLPPHQLKNEPGEVEISSSCQLQQQS

QPPPPSSAADNTIDPKRVKRILANRQSAQRSRVRKLQYISELERSVTTLQ

TEVSALSPRVAFLDHQRLILNVDNSALKQRIAALAQDKIFKDAHQEALKK

EIERLRQVYHQQSLKKMSGNQNGAAQPQEPPSGEGTPPQAAEMEKGQAVS

>ClabZIP6

MAQLPPKIPNMTPNWPDFSRQKIPSMETFAPPTTTPAVTQNNPSWVDEFL

DFSSARRGSHRRSVSDSITFLEMPMLEEDCRTAAAAPPPPPPGSGDRNEF

DRFDDEQFLSMFNDEISAAVAPTLSSSNPSTPSDHNSINDEKDAQNDGKQ

NQNKNESDEVQSQQQSENQTQSNSMATAATAASTDRITDPKRVKRILANR

QSAQRSRVRKLQYISELERSVTSLQAEVSVLSPRVAFLDHQRLLLNVDNS

ALKQRIAALAQDKIFKDAHQEALKREIERLRQVYHQQNIKKMENNAAPSP

AITPSRPVTAIATDAKLPNIDQNEQVPNIVV

>ClabZIP7

MDDGELDFSNQEVFSSPNMEIPSSCSMDSFFDELLKDTHTCTHTHTCNPP

GPDYSHTHTCFHVHTKIVPAPSEEDKVVTDDTAESTEKKSKKRPLGNREA

VRKYREKKKARAASLEDEVVRLRALNQHLMKRLQGQAALEAEIARLKCLL

VDIRGRIEGEIGSFPYQKAVNPNLSNPSIPGAFVMNPCNMQCEDQVYCLH

PGVDGNRSSEGAVINGQSFGACEFENPQCLANHDSGSKELPGCGVGNAVS

NDISSGKKKGEN

>ClabZIP8

MGDTEDANTENMRNLQCSYGVSSSSAGNLPFSMDQLKISQMNCSQIRPPH

FQSNFLADNSRRIGIPPSPNSPQIPPISPYSQIPISRPMNQQNYNPVPTH

SRSLSQPSFFSLDSLPPLSPSPFRESPTTSNSDQVSADTSMEDRDNSSHS

LLPPSPYMRANSSKMGDSLPPRKAHRRSSSDIPFGLSSMIQPSPLLPFNS

SGGLERSISSKENAGLLKPASQFVKREPSLEKSVDNNLEGMGERKSDGDS

VDDLFSAYMNLDNIDLFNSSGTNDKNGQENREDLDSRGSGTKTNGGESSD

NEAESSVNESGDSAQMPGLNSSAEKREGIKRTAGGDIAPTTRHYRSVSMD

SFMGKLQFGDESPKMPPTPPGVRPGQLSSNNLVDGNSAPFSLEFGNGEFS

GAELKKIMANDKLAEIALTDPKRAKRILANRQSAARSKERKMRYISELEH

KVQTLQTEATTLSAQLTLLQRDSVGLTNQNNELKFRLQAMEQQAQLRDAL

NEALTAEVQRLKLATTELNAQSHPSNGVMPQSSINHHGLQLQLQHQQQQQ

IQQNGNATTKPESNQ

>ClabZIP9

MGDTEDARTDNLRNLQCSFGTSSSSALKHHFSMDQLKISQMNCSQGRAQH

FQSNFLGDNSRRIGIPPCPNSPQIPPISPYSQIPVSRPMNQQSYNSVPTH

SRSLSQPSFFSLDSLPPLSPSPFRDSPSTSNSDQVSADTSMEDRDASSHS

LLPPSPYTRANSSKMGDALPPRKAHRRSNSDIPFGLSSMIQSSPLLPFSG

SGGLERSTSSKENAGIFKPASQFVKREPSLEKSIDNNLEGMGEKKSEGDT

VDDLFSAYMNLDNIDLFNSSGTNDKNGHENREDLDSRGSGTKTGGESSDN

EAESSVNESGDNSQVPGLNSSAEKREGIKRTAGGDIAPNNRHYRSISMDS

FMGKLQFGDESPKMPPTPPGIRPGQLSSNNLVDGNSTPFSLEFGNGEFSG

AELKKIMANDKLAEIALTDPKRAKRILANRQSAARSKERKMRYISELEHK

VQTLQTEATTLSAQLTLLQRDSVGLTNQNNELKFRLQAMEQQAQLRDGID

NFS

>ClabZIP11

MEDGELESSNPEVFSSSNAVELPSSCSMDSFFDEILKDTHACTHAHTCNP

PGPDYSHTHTCFHVHTKIVSSPTEEKVSTDDTAESVDKKNKKRPLGNREA

VRKYREKKKARAASLEDEVVRLRALNQQLLKRLQGQAALEAEISRLKCLL

VDIRGRIEGEIGTFPYRKPANSDLPNQNVPGSYMVNPCNVQCNDQAYCLH

PGDDGKSGESVSLNGQSFSACDFENLQCLANQNTGVKELPDCGLGNTISN

VNCSELNPKKGKTRALNF

>ClabZIP26

MELPNSTNQMASSSNPTTPFRGSFHRRAHSEVHFRIPDDLDLVSDPFDAP

SSGFGDLGFEDDLLCTFMDIEKIGSKIDNGSSSNPEVAGGGTAAENVEGE

KISRPRHRHSNSADGSSIMESIEAKKAMDPDKLAELWTIDPKRAKRILAN

RQSAARSKERKARYIMELERKVQSLQTEATTLSAQLTLYQRDTTGLSTEN

SELKLRLQAMEQQAHLRDALNEALKKEVERLKIATGEVMTATDSYNFGMS

QVSYPQSCFSHQPQPERHNPQRTTQGPQVHPFHSSLPNPHQSLFVASHQP

HALTEMFHQDPISRLQGLDISSRGTEIKPEGSSISVSESSSTF

>ClabZIP32

MMDLNFTSRKPPQTAPRMDIEQMPEAPHRGYHHRRSHSDTSFRFANFDDL

LLFDPPDIDLSSALPSPSPSPSPTPSGARMAVDSFNSRSPEDVSDTKPRA

GNGNSASFFSSHYRSLSMDSDFFEGLGMAGDGSDGEVLGGRVTAGEKKMS

RHRHSNSMDGSLTSSFEANSTKKAMAPDKLAELALMDPKRAKRILANRQS

AARSKERKIRYTNELEKKVQMLQSEATSLSAQVTVLQRDTTGLTTENREL

KLRLQAMEQQAHLRDALNETLKEEVQRLKIVAAQLPVANGNSFNIRGVLP

PQFPPLQTSSLLQFGNSQNHHQQPQLLHMSQPDARGGSPPSQPPGV

>ClabZIP40

MAMLNMAEDGVGFQCEVLENDHEFTATEIEELLSLFLANDGPPSPGSDSQ

GSMRTSATCSTNDDERKLRRMISNRESARRSRWRKKRHLEDLTNEVNRLM

IQNRELKERLGRVLNHRHVVLRENDWLWMESVGLRARLSDLCRILAVIQ

>ClabZIP43

MLMEPNFSSAKPPQPAAMDIEQMSENPHRGSHHRRSHSDTSFRFPNLDEL

LFFDPSELDLSMLSSPSSPPPGGTAMAVDSSNAKFSDDAVRPKPEPIASG

PFGGHLRSLSMDSDFFKNLDLGGDSGEIDSLGKKTPASEQRPVRHRHSLS

MDGSSSSFEADSTLVIDGVKKAMDPERLAELALIDPKRAKRILANRQSAA

RSKERKIRYTNELERKVQTLQSEATTLSAQVTILQRDTSGLTVENKELKL

RLQAMEQQAQLRDALSEALKEEVQRLRIAAGQVASINGNLFNRPPQYPSS

RPPVHHFSSSHAQQGQQQQPPSMLATNQQQQSDPKWTNSSQLLSRSPDGQ

AKP

>ClabZIP60

MEESYKMRMNQGGGIPTMAAALPPLPPSCLGKLTTSGEKKLPFFQSNMNL

SMYGNDKSILSQREATITPPPKQHQSQLLDSDKDLTVEAKRLRRVMQSRQ

YSQKYRLKQLHYITQLESELKALQAEVTITTPRIKFMDRQNSLLRAENYS

IKEKLSAYTGELLFKEAQYEELKRERNMLKEIYEAYQLKLLETLKSSNNN

NNTTAASGSTFQLVENYPQIATKSNPFTMLEN
