# Supplementary material for: The bZIP gene family in watermelon: genome-wide identification and expression analysis under cold stress and root-knot nematode infection
Source: PeerJ. 2019 Oct 16;7:e7878. doi: 10.7717/peerj.7878 (PMC6800529; doi:10.7717/peerj.7878)
Supplement: Supplemental Information 4 [file peerj-07-7878-s004.doc]

**Table S2.** The amino acid sequences of AtbZIP proteins.

>AtbZIP1

MANAEKTSSGSDIDEKKRKRKLSNRESARRSRLKKQKLMEDTIHEISSLERRIKENSERCRAVKQRLDSVETENAGLRSEKIWLSSYVSDLENMIATTSLTLTQSGGGDCVDDQNANAGIAVGDCRRTPWKLSCGSLQPMASFKT

>AtbZIP2

MASSSSTYRSSSSSDGGNNNPSDSVVTVDERKRKRMLSNRESARRSRMRKQKHVDDLTAQINQLSNDNRQILNSLTVTSQLYMKIQAENSVLTAQMEELSTRLQSLNEIVDLVQSNGAGFGVDQIDGCGFDDRTVGIDGYYDDMNMMSNVNHWGGSVYTNQPIMANDINMY

>AtbZIP3

MQPNYDSSSLNNMQQQDYFNLNNYYNNLNPSTNNNNLNILQYPQIQELNLQSPVSNNSTTSDDATEEIFVINERKQRRMVSNRESARRSRMRKQRHLDELLSQVAWLRSENHQLLDKLNQVSDNNDLVIQENSSLKEENLELRQVITSMKKLGGGIHDKYSSPSSMDELDQDFSSITDDPRTHHPS

>AtbZIP4

MFYTNMETVHNCFNDATITGDEIIDILAFLQSDESDNPSGINEVVPVDDKKRRRTISNRESAKRSRMKKKKRFEELTEEVNRLNIRNQELKNRLANVVSCGNFISRENNRLKTESVCLEIRLLELYRFLVAMQSPISTSVNYITTLEI

>AtbZIP5

MMSTISPVFSTEPGLLTSVLPAFETSFTPWDISHLFSVFDSLIDPKPVSTHDYGSVNQIGSDMSPTDNTDERKKKRKLSNRESAKRSREKKQKHLEEMSIQLNQLKIQNQELKNQLRYVLYHCQRTKMENDRLLMEHRILHDKLLNIRQVLMFRQT

>AtbZIP6

MMSTVPAFTFTEPGLVNQLSDFQTGFTPWELNCSDLFSTIHLEPVVPSPCSGESDAGSVKINTDFNGFDESCIGSIKTNSGSDDSNLFHGVPSPQSDELDSKNTKIRSNATNHNRNKLNRSVLQVTDDRKRKRMESNRESAKRSRMRKQRHIDNLKDEANRLGLENRELANRLRIVLYNIALMCTDNNQLLSEQEILRRRFLEMRQILIFRQLQLNPSLIINHHHMI

>AtbZIP7

MTQKLFIQYSLRSYIVFWKYDQITPWFVHKYQAPTMQFFPFPLLISLISLLEKLLAIMLSTAPAFSFSEPGLVNQFSGFQTGFTPWEWDCSDLFFVDQMSLEPAIPSPCYGESDTGSVKINSGSHDMKTGSDESCAGFVKINPRCDDADISNDLPCSQADEPDSDDTKQLTAITNFGSGENNHNRKKMIQPEMTDERKRKRMESNRESAKRSRMRKQSHIDNLREQVNRLDLENRELGNRLRLVLHQLQRVNSDNNRLVTEQEILRLRLSEMRRILIIRQLQQQQQWELHNRRMIMTEQNHPHLQ

>AtbZIP8

MAGSVYNLPSQNPNPQSLFQIFVDRVPLSNLPATSDDSSRTAEDNERKRRRKVSNRESARRSRMRKQRHMEELWSMLVQLINKNKSLVDELSQARECYEKVIEENMKLREENSKSRKMIGEIGLNRFLSVEADQIWTF

>AtbZIP9

MDNHTAKDIGMKRSASELALQEYLTTSPLDPCFDLMNRDYTCELRDSLLWSEGLFPAGPFRDAQSSICENLSADSPVSANKPEVRGGVRRTTSGSSHVNSDDEDAETEAGQSEMTNDPNDLKRIRRMNSNRESAKRSRRRKQEYLVDLETQVDSLKGDNSTLYKQLIDATQQFRSAGTNNRVLKSDVETLRVKVKLAEDLVARGSLTSSLNQLLQTHLSPPSHSISSLHYTGNTSPAITVHSDQSLFPGMTLSGQNSSPGLGNVSSEAVSCVSDIWP

>AtbZIP10

MNSIFSIDDFSDPFWETPPIPLNPDSSKPVTADEVSQSQPEWTFEMFLEEISSSAVSSEPLGNNNNAIVGVSSAQSLPSVSGQNDFEDDSRFRDRDSGNLDCAAPMTTKTVIVDSDDYRRVLKNKLETECATVVSLRVGSVKPEDSTSSPETQLQPVQSSPLTQGSLMTPGELGVTSSLPAEVKKTGVSMKQVTSGSSREYSDDEDLDEENETTGSLKPEDVKKSRRMLSNRESARRSRRRKQEQTSDLETQVNDLKGEHSSLLKQLSNMNHKYDEAAVGNRILKADIETLRAKVKMAEETVKRVTGMNPMLLGRSSGHNNNNRMPITGNNRMDSSSIIPAYQPHSNLNHMSNQNIGIPTILPPRLGNNFAAPPSQTSSPLQRIRNGQNHHVTPSANPYGWNTEPQNDSAWPKKCVD

>AtbZIP11

MESSSSGTTSSTIQTSSGSEESLMEQRKRKRMLSNRESARRSRMKKQKLLDDLTAQVNHLKKENTEIVTSVSITTQHYLTVEAENSVLRAQLDELNHRLQSLNDIIEFLDSSNNNNNNNMGMCSNPLVGLECDDFFVNQMNMSYIMNQPLMASSDALMY

>AtbZIP12

MGSIRGNIEEPISQSLTRQNSLYSLKLHEVQTHLGSSGKPLGSMNLDELLKTVLPPAEEGLVRQGSLTLPRDLSKKTVDEVWRDIQQDKNGNGTSTTTTHKQPTLGEITLEDLLLRAGVVTETVVPQENVVNIASNGQWVEYHHQPQQQQGFMTYPVCEMQDMVMMGGLSDTPQAPGRKRVAGEIVEKTVERRQKRMIKNRESAARSRARKQAYTHELEIKVSRLEEENEKLRRLKEVEKILPSEPPPDPKWKLRRTNSASL

>AtbZIP13

MTSFQVMRSSNSRNSDLSRRISSASTSSSSIRPQQQFRRDLTSVGYGGRNDGLYSSNSMTVEGILHDTFASDPPAPTESSLLDASINLMDASPAPMEITTTTASDVVDHGGGTETTRGGKSVDEIWREMVSGEGKGMKEETSEEIMTLEDFLAKAAVEDETAVTASAEDLDVKIPVTNYGFDHSAPPHNPFQMIDKVEGSIVAFGNGLDVYGGGARGKRARVMVEPLDKAAAQRQRRMIKNRESAARSRERKQAYQVELEALAAKLEEENELLSKEIEDKRKERYQKLMEFVIPVVEKPKQQPPRFLRRIRSLEW

>AtbZIP14

MLSSAKHQRNHRLSATNKNQTLTKVSSISSSSPSSSSSSSSTSSSSPLPSQDSQAQKRSLVTMEEVWNDINLASIHHLNRHSPHPQHNHEPRFRGQNHHNQNPNSIFQDFLKGSLNQEPAPTSQTTGSAPNGDSTTVTVLYSSPFPPPATVLSLNSGAGFEFLDNQDPLVTSNSNLHTHHHLSNAHAFNTSFEALVPSSSFGKKRGQDSNEGSGNRRHKRMIKNRESAARSRARKQECLYKRVRT

>AtbZIP15

MDSYWRLKNLVNDLPVSTSLSRQGSIYSWTVDQFQTSLGLDCGSMNMDELVKHISSAEETQEGSQRQGSTTLPPTLSKQNVGEVWKSITEEKHTNNNGGVTNITHLQGQQTLGEITLEEFFIRAGARGGNTNGGSIHDSSSSISGNPHTSLGVQIQPKAMVSDFMNNMVPRSHDSYLHQNVNGSMSTYQPQQSIMSMPNGYSYGKQIRFSNGSLGSGNQSLQDTKRSLVPSVATIPSEAITCSPVTPFPTLNGKQKINGESSLLSPSPYISNGSTSTRGGKINSEITAEKQFVDKKLRRKIKNRESAARSRARKQAQTMEVEVELENLKKDYEELLKQHVELRKRQMEPGMISLHERPERKLRRTKSDIK

>AtbZIP16

MASNEMEKSSKEKEPKTPPPSSTAPPSSQEPSSAVSAGMATPDWSGFQAYSPMPPPHGYVASSPQPHPYMWGVQHMMPPYGTPPHPYVAMYPPGGMYAHPSMPPGSYPYSPYAMPSPNGMTEVSGNTTGGTDGDAKQSEVKEKLPIKRSRGSLGSLNMITGKNNEPGKNSGASANGAYSKSGESASDGSSEGSDGNSQNDSGSGLDGKDAEAASENGGSANGPQNGSAGTPILPVSQTVPIMPMTAAGVPGPPTNLNIGMDYWGAPTSAGIPGMHGKVSTPVPGVVAPGSRDGGHSQPWLQDDRELKRQRRKQSNRESARRSRLRKQAECDELAQRAEVLNEENTNLRAEINKLKSQCEELTTENTSLKDQLSLFPPLEGISMDNDHQEPDTNQTGAAERKVDSYKDST

>AtbZIP17

MAEPITKEQPPPPAPDPNSTYPPPSDFDSISIPPLDDHFSDQTPIGELMSDLGFPDGEFELTFDGMDDLYFPAENESFLIPINTSNQEQFGDFTPESESSGISGDCIVPKDADKTITTSGCINRESPRDSDDRCSGADHNLDLPTPLSSQGSGNCGSDVSEATNESSPKSRNVAVDQKVKVEEAATTTTSITKRKKEIDEDLTDESRNSKYRRSGEDADASAVTGEEDEKKRARLMRNRESAQLSRQRKKHYVEELEEKVRNMHSTITDLNGKISYFMAENATLRQQLGGNGMCPPHLPPPPMGMYPPMAPMPYPWMPCPPYMVKQQGSQVPLIPIPRLKPQNTLGTSKAKKSESKKSEAKTKKVASISFLGLLFCLFLFGALAPIVNVNYGGISGAFYGNYRSNYITDQIYSQHRDRVLDTSRSGAGTGVSNSNGMHRGRDSDRGARKNISATESSVTPGNGSEPLVASLFVPRNDKLVKIDGNLIINSILASEKAVASRKASESKERKADLMISKDYTPALPLPDVGRTEELAKHLYRSKAEKQKALSSGSADTLKDQVKTKAANGEMQQWFREGVAGPMFSSGMCTEVFQFDVSSTSGAIIPAATNVSAEHGKNTTDTHKQQNRRILRGLPIPLPGSDFNLTKEHQRNSSSKEIKPASSMVVSVLVDPREGGDGDIDGMIGGPKSLSRVFVVVLLDSAKYVTYSCVLPRSGAPHLVTT

>AtbZIP18

MEDPSNPQPNQSNLSQCPPLATAPTPAPVRGPYHRRAHSEVQFRLPEDLDLSEPFGGFDELGSEDDLFCSYMDIEKLGSGSGSASDSAGPSAPRSDNPFSAENGGAEAGNSRPRHRHSLSVDGSSTLESIEAKKAMAPDKLAELWVVDPKRAKRIIANRQSAARSKERKARYILELERKVQTLQTEATTLSAQLSLFQRDTTGLSSENTELKLRLQVMEQQAKLRDALNEQLKKEVERLKFATGEVSPADAYNLGMAHMQYQQQPQQSFFQHHHQQQTDAQNLQQMTHQFHLFQPNNNQNQSSRTNPPTAHQLMHHATSNAPAQSHSYSEAMHEDHLGRLQGLDISSCGRGSNFGRSDTVSESSSTM

>AtbZIP19

MEDGELDFSNQEVFSSSEMGELPPSNCSMDSFFDGLLMDTNAACTHTHTCNPTGPENTHTHTCFHVHTKILPDESDEKVSTDDTAESCGKKGEKRPLGNREAVRKYREKKKAKAASLEDEVARLRAVNQQLVKRLQNQATLEAEVSRLKCLLVDLRGRIDGEIGSFPYQKPMAANIPSFSHMMNPCNVQCDDEVYCPQNVFGVNSQEGASINDQGLSGCDFDQLQCMANQNLNGNGNGSFSNVNTSVSNKRKGGHRASRAV

>AtbZIP20

MADTSPRTDVSTDDDTDHPDLGSEGALVNTAASDSSDRSKGKMDQKTLRRLAQNREAARKSRLRKKAYVQQLENSRLKLTQLEQELQRARQQGVFISGTGDQAHSTGGNGALAFDAEHSRWLEEKNKQMNELRSALNAHAGDSELRIIVDGVMAHYEELFRIKSNAAKNDVFHLLSGMWKTPAERCFLWLGGFRSSELLKLLANQLEPMTERQLMGINNLQQTSQQAEDALSQGMESLQQSLADTLSSGTLGSSSSGNVASYMGQMAMAMGKLGTLEGFIRQADNLRLQTLQQMIRVLTTRQSARALLAIHDYFSRLRALSSLWLARPRE

>AtbZIP21

MANHRMSEATNHNHNHHLPYSLIHGLNNNHPSSGFINQDGSSSFDFGELEEAIVLQGVKYRNEEAKPPLLGGGGGATTLEMFPSWPIRTHQTLPTESSKSGGESSDSGSANFSGKAESQQPESPMSSKHHLMLQPHHNNMANSSSTSGLPSTSRTLAPPKPSEDKRKATTSGKQLDAKTLRRLAQNREAARKSRLRKKAYVQQLESSRIKLSQLEQELQRARSQGLFMGGCGPPGPNITSGAAIFDMEYGRWLEDDNRHMSEIRTGLQAHLSDNDLRLIVDGYIAHFDEIFRLKAVAAKADVFHLIIGTWMSPAERCFIWMAGFRPSDLIKILVSQMDLLTEQQLMGIYSLQHSSQQAEEALSQGLEQLQQSLIDTLAASPVIDGMQQMAVALGKISNLEGFIRQADNLRQQTVHQLRRILTVRQAARCFLVIGEYYGRLRALSSLWLSRPRETLMSDETSCQTTTDLQIVQSSRNHFSNF

>AtbZIP22

MEMMSSSSSTTQVVSFRDMGMYEPFQQLSGWESPFKSDINNITSNQNNNQSSSTTLEVDARPEADDNNRVNYTSVYNNSLEAEPSSNNDQDEDRINDKMKRRLAQNREAARKSRLRKKAHVQQLEESRLKLSQLEQELVRARQQGLCVRNSSDTSYLGPAGNMNSGIAAFEMEYTHWLEEQNRRVSEIRTALQAHIGDIELKMLVDSCLNHYANLFRMKADAAKADVFFLMSGMWRTSTERFFQWIGGFRPSELLNVVMPYVEPLTDQQLLEVRNLQQSSQQAEEALSQGLDKLQQGLVESIAIQIKVVESVNHGAPMASAMENLQALESFVNQADHLRQQTLQQMSKILTTRQAARGLLALGEYFHRLRALSSLWAARPREHT

>AtbZIP23

MDDGELEFSNSNMGGELPSCSMDSFFDELLRDSHACTHTHTCNPPGPENTHTHTCLHVHTKILPDKVSTDDTSESSGKKRPLGNREAVRKYREKKKAKAASLEDEVMRLKAVNNQLLKRLQGQAALEAEVTRLKCLLVDIRGRIDGEIGAFPYQKPAVTNVPYSYMMHPCNMQCDVDNLYCLQNGNNGEGASMNEQGLNGCEFDQLECLANQNLAGKEIPVCSNGIGTFTVNGSGVNKRKGEPRAAKAV

>AtbZIP24

MFCCCKDCRGNQRVSNFDSLTGVFFGDLEFGPQNQRYIKMNEEEDKDQDRVTRGCSHTHSCNPPGPEDASHSHTCFHAHTHLIISQDQQENDHSDSSNKKRLCGNREAVRKYREKKKARTAYLEDEVMRLQSLNEQFLRKLQSQEMVETELIRLRALLVEMQGKIEVELCSFSFQKQCNGSGFVFKEDGCNLATSNMMCEAARVECEEGQTLHDPIQSFVPQPPPFSR

>AtbZIP25

MHIVFSVDDLTESFWPVPAPAPSPGSSSTPSPTQNVADGMTRSQSEWAFHRLINELSGSDSSPTTNTIERSPPPVQSLSRLEETVDETEDVVEIQKPQNHRRLPVDDQGKNRNRAPSSDPVDSSAPVVVDPNQYHAILKSKLELACAAVARRVGTVKPEDSSASASNQKQAQGSIVAQTSPGASSVRFSPTTSTQKKPDVPARQTSISSRDDSDDDDLDGDADNGDPTDVKRARRMLSNRESARRSRRRKQEQMNEFDTQVGQLRAEHSTLINRLSDMNHKYDAAAVDNRILRADIETLRTKVKMAEETVKRVTGVNPLHWSRPNMGIPFSNTPSASSSIPPNSNHILKPANSSTNTSAGLAQNQRVETANFLPEQVNREGMQNPFAPDSNLYETLPHWNHKH

>AtbZIP26

MGDTSPRTSVSTDGDTDHNNLMFDEGHLGIGASDSSDRSKSKMDQKTLRRLAQNREAARKSRLRKKAYVQQLENSRLKLTQLEQELQRARQQGVFISSSGDQAHSTAGDGAMAFDVEYRRWQEDKNRQMKELSSAIDSHATDSELRIIVDGVIAHYEELYRIKGNAAKSDVFHLLSGMWKTPAERCFLWLGGFRSSELLKLIASQLEPLTEQQSLDINNLQQSSQQAEDALSQGMDNLQQSLADTLSSGTLGSSSSGNVASYMGQMAMAMGKLGTLEGFIRQADNLRLQTYQQMVRLLTTRQSARALLAVHNYTLRLRALSSLWLARPRE

>AtbZIP27

MEEVWKEINLGSLHYHRQLNIGHEPMLKNQNPNNSIFQDFLNMPLNQPPPPPPPPSSSTIVTALYGSLPLPPPATVLSLNSGVGFEFLDTTENLLASNPRSFEESAKFGCLGKKRGQDSDDTRGDRRYKRMIKNRESAARSRARKQECVSPHSSTF

>AtbZIP28

MTESTSVVAPPPEIPNLNPSMFSESDLFSIPPLDPLFLSDSDPISMDAPISDLDFLLDDENGDFADFDFSFDNSDDFFDFDLSEPAVVIPEEIGNNRSNLDSSENRSGDGGLEGRSESVHSQVSSQGSKTFVSDTVDASSSPESSNHQKSSVSKRKKENGDSSGELRSCKYQKSDDKSVATNNEGDDDDDKRKLIRQIRNRESAQLSRLRKKQQTEELERKVKSMNATIAELNGKIAYVMAENVALRQQMAVASGAPPMNPYMAAPPLPYQWMPYPPYPVRGYGSQTPLVPIPKLNPKPVSSCRPKKAESKKNEGKSKLKKVASISFIGILFFVFLFGTLVPFMNVNFGGERGSFGGLSKYDGHRYYDEHKGRVLMVGDGSDVRRNSGISEGNIHSSRISHGERDSCGGVDYNAHPKVEGRPSSLSNASDPLFASLYVPRNDGLVKIDGNLIIHSVLASEKARGLGKKNITETVKTKEPDLTIPGALSSALAVPGVRGNAAMLPHSTALSSEGKRLHQWFHEGGSGPLMDYSMCTEVFQFDIAPGAIVPSSVSSISAEHLQNVTTHGKRMKNRRILEGLPVSLVASELNITGTQPNKDAQNKTFNGNTNKPTSSSSMVVSVLLDPREVVDSETDRVVPPNPKSLSRIFVVVLLDSVKYVTYSCVLPRSGLHLVAT

>AtbZIP29

MGDTEKCNSDMIQRLHSSFGTTSSSIPKNPISQLDLNPNFIRSSAPQFSKPFSDSGKRIGVPPSHPNLIPPTSPFSQIPTTRQPGSHNFNPGGANHSRSMSQPNSFFSFDSLPPLSPSPFRDHDVSMEDRDSGVFNSNHSLPPSPFTRCNSTSSSSLRVGESLPPRKSHRRSNSDIPSGFNSMPLIPPRPLERSFSGGECADWSKSNPFVKKESSCEREGVGEREAMDDLFSAYMNLENIDVLNSSEADDSKNGNENRDDMESSRASGTKTNGSDTEGESSSVNESANNNMNSSGEKRESVKRRAAGGDIAPTTRHYRSVSVDSCFMEKLSFGDESLKPPPSPGSMSRKVSPTNSVDGNSGAAFSIEFNNGEFTAAEMKKIMANDKLAEMAMSDPKRVKRNDPLFRILANRQSAARSKERKMRYIVELEHKVQTLQTEATTLSAQLTLLQRDMMGLTNQNNELKFRLQAMEQQARLRDALNEALNGEVQRLKLAIGESSQNESERSKMQSLNAEMFQQLNISQLRQQPQQMQQQSHQQNHQNGTMATKSESNE

>AtbZIP30

MGGGGDTTDTNMMQRVNSSSGTSSSSIPKHNLHLNPALIRSHHHFRHPFTGAPPPPIPPISPYSQIPATLQPRHSRSMSQPSSFFSFDSLPPLNPSAPSVSVSVEEKTGAGFSPSLPPSPFTMCHSSSSRNAGDGENLPPRKSHRRSNSDVTFGFSSMMSQNQKSPPLSSLERSISGEDTSDWSNLVKKEPREGFYKGRKPEVEAAMDDVFTAYMNLDNIDVLNSFGGEDGKNGNENVEEMESSRGSGTKKTNGGSSSDSEGDSSASGNVKVALSSSSSGVKRRAGGDIAPTGRHYRSVSMDSCFMGKLNFGDESSLKLPPSSSAKVSPTNSGEGNSSAYSVEFGNSEFTAAEMKKIAADEKLAEIVMADPKRVKRILANRVSAARSKERKTRYMAELEHKVQTLQTEATTLSAQLTHLQRDSMGLTNQNSELKFRLQAMEQQAQLRDGMHIIKTLSEKLNEEVQRLKLVIGEPNRRQSGSSSSESKMSLNPEMFQQLSISQLQHQQMQHSNQCSTMKAKHTSND

>AtbZIP31

MNGSDNSTPSRPRSITQPSLAFSSLPPLSPSPSSSRRNSIPLMNPSASVESRDSSIRVKKKSLLPPLCVKRRAPKDDIEPFKRHYRSLSVDSCLSDLLKLPPSPNNVSSSRSVDGEQNASELEFDTSVYTDDELNKIAKSTKLKEVASDPKEVRRILKNQESAARSKQKKLQYMINLELKINFLENKNASIFEKIKLLENDKTMRMNEKKEIMIRIESLEQHAELRDALTEHLHVEIERLKAVLISNEKGNEKSPKSRDDNM

>AtbZIP33

MNGSDNITTPSGSRSMTQPSLASSSLPPLSPSSSTFRRNSLPSMSFSPSAPVESRDSSIKVNKNPSLPPLVDGEEDASWLEFGSSDYTDDELNKIAKSTKLQDIAKSTKLQEIVSDPKKVRRILKNRELAASSKQRKLKYMIDLEHRIKFLENKNALIFEKIKLLEKDKTILMNEKKEITIQIESLEQQAQLRDALTEKLHVEIERLKVITISNEKGSVELQRLKMETCEVLQYRREFDRSNMQGMDPNMFTWSQPNLGFYGQI

>AtbZIP34

MAQLPPKIPNMTQHWPDFSSQKLSPFSTPTATAVATATTTVQNPSWVDEFLDFSASRRGNHRRSISDSIAFLEAPTVSIEDHQFDRFDDEQFMSMFTDDDNLHSNPSHINNKNNNVGPTGSSSNTSTPSNSFNDDNKELPPSDHNMNNNINNNYNDEVQSQCKMEPEDGTASNNNSGDSSGNRILDPKRVKRILANRQSAQRSRVRKLQYISELERSVTSLQAEVSVLSPRVAFLDHQRLLLNVDNSALKQRIAALSQDKLFKDAHQEALKREIERLRQVYNQQSLTNVENANHLSATGAGATPAVDIKSSVETEQLLNVS

>AtbZIP35

MGTHIDINNLGGDTSRGNESKPLARQSSLYSLTFDELQSTLGEPGKDFGSMNMDELLKNIWTAEDTQAFMTTTSSVAAPGPSGFVPGGNGLQRQGSLTLPRTLSQKTVDEVWKYLNSKEGSNGNTGTDALERQQTLGEMTLEDFLLRAGVVKEDNTQQNENSSSGFYANNGAAGLEFGFGQPNQNSISFNGNNSSMIMNQAPGLGLKVGGTMQQQQQPHQQQLQQPHQRLPPTIFPKQANVTFAAPVNMVNRGLFETSADGPANSNMGGAGGTVTATSPGTSSAENNTWSSPVPYVFGRGRRSNTGLEKVVERRQKRMIKNRESAARSRARKQAYTLELEAEIESLKLVNQDLQKKQAEIMKTHNSELKEFSKQPPLLAKRQCLRRTLTGPW

>AtbZIP36

MDGSMNLGNEPPGDGGGGGGLTRQGSIYSLTFDEFQSSVGKDFGSMNMDELLKNIWSAEETQAMASGVVPVLGGGQEGLQLQRQGSLTLPRTLSQKTVDQVWKDLSKVGSSGVGGSNLSQVAQAQSQSQSQRQQTLGEVTLEEFLVRAGVVREEAQVAARAQIAENNKGGYFGNDANTGFSVEFQQPSPRVVAAGVMGNLGAETANSLQVQGSSLPLNVNGARTTYQQSQQQQPIMPKQPGFGYGTQMGQLNSPGIRGGGLVGLGDQSLTNNVGFVQGASAAIPGALGVGAVSPVTPLSSEGIGKSNGDSSSLSPSPYMFNGGVRGRKSGTVEKVVERRQRRMIKNRESAARSRARKQAYTVELEAEVAKLKEENDELQRKQARIMEMQKNQETEMRNLLQGGPKKKLRRTESGPW

>AtbZIP37

MGSRLNFKSFVDGVSEQQPTVGTSLPLTRQNSVFSLTFDEFQNSWGGGIGKDFGSMNMDELLKNIWTAEESHSMMGNNTSYTNISNGNSGNTVINGGGNNIGGLAVGVGGESGGFFTGGSLQRQGSLTLPRTISQKRVDDVWKELMKEDDIGNGVVNGGTSGIPQRQQTLGEMTLEEFLVRAGVVREEPQPVESVTNFNGGFYGFGSNGGLGTASNGFVANQPQDLSGNGVAVRQDLLTAQTQPLQMQQPQMVQQPQMVQQPQQLIQTQERPFPKQTTIAFSNTVDVVNRSQPATQCQEVKPSILGIHNHPMNNNLLQAVDFKTGVTVAAVSPGSQMSPDLTPKSALDASLSPVPYMFGRVRKTGAVLEKVIERRQKRMIKNRESAARSRARKQAYTMELEAEIAQLKELNEELQKKQVEIMEKQKNQLLEPLRQPWGMGCKRQCLRRTLTGPW

>AtbZIP38

MGTHINFNNLGGGGHPGGEGSSNQMKPTGSVMPLARQSSVYSLTFDELQNTLGGPGKDFGSMNMDELLKSIWTAEEAQAMAMTSAPAATAVAQPGAGIPPPGGNLQRQGSLTLPRTISQKTVDEVWKCLITKDGNMEGSSGGGGESNVPPGRQQTLGEMTLEEFLFRAGVVREDNCVQQMGQVNGNNNNGFYGNSTAAGGLGFGFGQPNQNSITFNGTNDSMILNQPPGLGLKMGGTMQQQQQQQQLLQQQQQQMQQLNQPHPQQRLPQTIFPKQANVAFSAPVNITNKGFAGAANNSINNNNGLASYGGTGVTVAATSPGTSSAENNSLSPVPYVLNRGRRSNTGLEKVIERRQRRMIKNRESAARSRARKQAYTLELEAEIEKLKKTNQELQKKQAEMVEMQKNELKETSKRPWGSKRQCLRRTLTGPW

>AtbZIP39

MVTRETKLTSEREVESSMAQARHNGGGGGENHPFTSLGRQSSIYSLTLDEFQHALCENGKNFGSMNMDEFLVSIWNAEENNNNQQQAAAAAGSHSVPANHNGFNNNNNNGGEGGVGVFSGGSRGNEDANNKRGIANESSLPRQGSLTLPAPLCRKTVDEVWSEIHRGGGSGNGGDSNGRSSSSNGQNNAQNGGETAARQPTFGEMTLEDFLVKAGVVREHPTNPKPNPNPNQNQNPSSVIPAAAQQQLYGVFQGTGDPSFPGQAMGVGDPSGYAKRTGGGGYQQAPPVQAGVCYGGGVGFGAGGQQMGMVGPLSPVSSDGLGHGQVDNIGGQYGVDMGGLRGRKRVVDGPVEKVVERRQRRMIKNRESAARSRARKQAYTVELEAELNQLKEENAQLKHALAELERKRKQQYFESLKSRAQPKLPKSNGRLRTLMRNPSCPL

>AtbZIP40

MASFKLMSSSNSDLSRRNSSSASSSPSIRSSHHLRPNPHADHSRISFAYGGGVNDYTFASDSKPFEMAIDVDRSIGDRNSVNNGKSVDDVWKEIVSGEQKTIMMKEEEPEDIMTLEDFLAKAEMDEGASDEIDVKIPTERLNNDGSYTFDFPMQRHSSFQMVEGSMGGGVTRGKRGRVMMEAMDKAAAQRQKRMIKNRESAARSRERKQAYQVELETLAAKLEEENEQLLKEIEESTKERYKKLMEVLIPVDEKPRPPSRPLSRSHSLEW

>AtbZIP41

MGTSEDKMPFKTTKPTSSAQEVPPTPYPDWQNSMQAYYGGGGTPNPFFPSPVGSPSPHPYMWGAQHHMMPPYGTPVPYPAMYPPGAVYAHPSMPMPPNSGPTNKEPAKDQASGKKSKGNSKKKAEGGDKALSGSGNDGASHSDESVTAGSSDENDENANQQEQGSIRKPSFGQMLADASSQSTTGEIQGSVPMKPVAPGTNLNIGMDLWSSQAGVPVKDERELKRQKRKQSNRESARRSRLRKQAECEQLQQRVESLSNENQSLRDELQRLSSECDKLKSENNSIQDELQRVLGAEAVANLEQNAAGSKDGEGTN

>AtbZIP42

MQPQTDVFSLHNYLNSSILQSPYPSNFPISTPFPTNGQNPYLLYGFQSPTNNPQSMSLSSNNSTSDEAEEQQTNNNIINERKQRRMISNRESARRSRMRKQRHLDELWSQVMWLRIENHQLLDKLNNLSESHDKVLQENAQLKEETFELKQVISDMQIQSPFSCFRDDIIPIE

>AtbZIP43

MQPSTNIFSLHGCPPSYLSHIPTSSPFCGQNPNPFFSFETGVNTSQFMSLISSNNSTSDEAEENHKEIINERKQKRKISNRESARRSRMRKQRQVDELWSQVMWLRDENHQLLRKLNCVLESQEKVIEENVQLKEETTELKQMISDMQLQNQSPFSCIRDDDDVV

>AtbZIP44

MNNKTEMGSSTSGNCSSVSTTGLANSGSESDLRQRDLIDERKRKRKQSNRESARRSRMRKQKHLDDLTAQVTHLRKENAQIVAGIAVTTQHYVTIEAENDILRAQVLELNHRLQSLNEIVDFVESSSSGFGMETGQGLFDGGLFDGVMNPMNLGFYNQPIMASASTAGDVFNC

>AtbZIP45

MADTSSRTDVSTDGDTDHRDLGFYYLYNVTPGRLVPESLGKTWGILPSDRGHMHAAASDSSDRSKDKLDQKTLRRLAQNREAARKSRLRKKAYVQQLENSRLKLTQLEQELQRARQQGVFISSSGDQAHSTGGNGALAFDAEHSRWLEEKNRQMNELRSALNAHAGDTELRIIVDGVMAHYEELFRIKSNAAKNDVFHLLSGMWKTPAERCFLWLGGFRSSELLKLLANQLEPMTERQVMGINSLQQTSQQAEDALSQGMESLQQSLADTLSSGTLGSSSSDNVASYMGQMAMAMGQLGTLEGFIRQADNLRLQTLQQMLRVLTTRQSARALLAIHDYSSRLRALSSLWLARPRE

>AtbZIP46

MQSSFKTVPFTPDFYSQSSYFFRGDSCLEEFHQPVNGFHHEEAIDLSPNVTIASANLHYTTFDTVMDCGGGGGGGLRERLEGGEEECLDTGQLVYQKGTRLVGGGVGEVNSSWCDSVSAMADNSQHTDTSTDIDTDDKTQLNGGHQGMLLATNCSDQSNVKSSDQRTLRRLAQNREAARKSRLRKKAYVQQLENSRIRLAQLEEELKRARQQGSLVERGVSADHTHLAAGNGVFSFELEYTRWKEEHQRMINDLRSGVNSQLGDNDLRVLVDAVMSHYDEIFRLKGIGTKVDVFHMLSGMWKTPAERFFMWLGGFRSSELLKILGNHVDPLTDQQLIGICNLQQSSQQAEDALSQGMEALQQSLLETLSSASMGPNSSANVADYMGHMAMAMGKLGTLENFLRQADLLRQQTLQQLHRILTTRQAARAFLVIHDYISRLRALSSLWLARPRD

>AtbZIP47

MNSTSTHFVPPRRVGIYEPVHQFGMWGESFKSNISNGTMNTPNHIIIPNNQKLDNNVSEDTSHGTAGTPHMFDQEASTSRHPDKIQRRLAQNREAARKSRLRKKAYVQQLETSRLKLIQLEQELDRARQQGFYVGNGIDTNSLGFSETMNPGIAAFEMEYGHWVEEQNRQICELRTVLHGHINDIELRSLVENAMKHYFELFRMKSSAAKADVFFVMSGMWRTSAERFFLWIGGFRPSDLLKVLLPHFDVLTDQQLLDVCNLKQSCQQAEDALTQGMEKLQHTLADCVAAGQLGEGSYIPQVNSAMDRLEALVSFVNQADHLRHETLQQMYRILTTRQAARGLLALGEYFQRLRALSSSWATRHREPT

>AtbZIP48

MIPAEINGYFQYLSPEYNVINMPSSPTSSLNYLNDLIINNNNYSSSSNSQDLMISNNSTSDEDHHQSIMVLDERKQRRMLSNRESARRSRMRKQRHLDELWSQVIRLRNENNCLIDKLNRVSETQNCVLKENSKLKEEASDLRQLVCELKSNKNNNNSFPREFEDN

>AtbZIP49

MAEPVLEDTYLTFSSDFDYIAIAPSPFDNFCNSNSDQVRNSISDLRFLIDDDDSFDDLYFPSENESFCIPPDATKREMSGDFTPASGISGDCVNEDTEKNTNGVLISTSSCYNRESPTDSDFSGTSQSLSFSGQDSAKRKTEIEEDSSDESRRLGKDGFASVIKVGGEEDDEKKKNVRLVRNRESAHLSRQRKKHYVEELEDKVKNMHSTISELSSKMSYFVAENVTLRQQMGTRFSSGPPMVPIVYPWMQYPAYMVKPQGSQVALLPIPRLKPKHSVAKVKKFKKVASFSVFGFLFCMFLFGALVNISYGEYKSNYVTDGVYDQSRGRVLVVDSSRVHCGGDSDQGVGRNVSETENLGPPRNSSEPLVASLFVPRNEKLVKIDGNLIIHSVLASEKARDSETKNEEGKSVLATTTKTLSPALPLPDSTSPRTRDVSKHLYSETGKGLSSSGSDDASNDQLKSTIANGKMQQWFREGVAGPMFSSGMCTEVFQFDVSSNSGAIIPASPHTQQCKNTSDTQKGKKNRRILSGGLPVSDFNLTKEDHNSSSKDKFRETKPGPSMVVSVLVDPREGGNGDIDGMMGGTKPQSRVFIVVLVDGVKYITYSCVLPRPDVPHLMTS

>AtbZIP50

MMSSSSPTQLASLRDMGIYEPFQQIVGWGNVFKSDINDHSPNTATSSIIQVDPRIDDHNNNIKINYDSSHNQIEAEQPSSNDNQDDDGRIHDKMKRRLAQNREAARKSRLRKKAYVQQLEESRLKLSQLEQELEKVKQQGHLGPSGSINTGIASFEMEYSHWLQEQSRRVSELRTALQSHISDIELKMLVESCLNHYANLFQMKSDAAKADVFYLISGMWRTSTERFFQWIGGFRPSELLNVVMPYLQPLTDQQILEVRNLQQSSQQAEDALSQGIDKLQQSLAESIVIDAVIESTHYPTHMAAAIENLQALEGFVNQADHLRQQTLQQMAKILTTRQSARGLLALGEYLHRLRALSSLWAARPQEPT

>AtbZIP51

MEGGGRGPNQTILSEIEHMPEAPRQRISHHRRARSETFFSGESIDDLLLFDPSDIDFSSLDFLNAPPPPQQSQQQPQASPMSVDSEETSSNGVVPPNSLPPKPEARFGRHVRSFSVDSDFFDDLGVTEEKFIATSSGEKKKGNHHHSRSNSMDGEMSSASFNIESILASVSGKDSGKKNMGMGGDRLAELALLDPKRAKRILANRQSAARSKERKIRYTGELERKVQTLQNEATTLSAQVTMLQRGTSELNTENKHLKMRLQALEQQAELRDALNEALRDELNRLKVVAGEIPQGNGNSYNRAQFSSQQSAMNQFGNKTNQQMSTNGQPSLPSYMDFTKRG

>AtbZIP52

MEKSDPPPVPKPGATIIPSSDPIPNADPIPSSSFHRRSRSDDMSMFMFMDPLSSAAPPSSDDLPSDDDLFSSFIDVDSLTSNPNPFQNPSLSSNSVSGAANPPPPPSSRPRHRHSNSVDAGCAMYAGDIMDAKKAMPPEKLSELWNIDPKRAKRILANRQSAARSKERKARYIQELERKVQSLQTEATTLSAQLTLYQRDTNGLANENTELKLRLQAMEQQAQLRNALNEALRKEVERMKMETGEISGNSDSFDMGMQQIQYSSSTFMAIPPYHGSMNLHDMQMHSSFNPMEMSNSQSVSDFLQNGRMQGLEISSNSSSLVKSEGPSLSASESSSAY

>AtbZIP53

MGSLQMQTSPESDNDPRYATVTDERKRKRMISNRESARRSRMRKQKQLGDLINEVTLLKNDNAKITEQVDEASKKYIEMESKNNVLRAQASELTDRLRSLNSVLEMVEEISGQALDIPEIPESMQNPWQMPCPMQPIRASADMFDC

>AtbZIP54

MGSNEEGNPTNNSDKPSQAAAPEQSNVHVYHHDWAAMQAYYGPRVGIPQYYNSNLAPGHAPPPYMWASPSPMMAPYGAPYPPFCPPGGVYAHPGVQMGSQPQGPVSQSASGVTTPLTIDAPANSAGNSDHGFMKKLKEFDGLAMSISNNKVGSAEHSSSEHRSSQSSENDGSSNGSDGNTTGGEQSRRKRRQQRSPSTGERPSSQNSLPLRGENEKPDVTMGTPVMPTAMSFQNSAGMNGVPQPWNEKEVKREKRKQSNRESARRSRLRKQAETEQLSVKVDALVAENMSLRSKLGQLNNESEKLRLENEAILDQLKAQATGKTENLISRVDKNNSVSGSKTVQHQLLNASPITDPVAAS

>AtbZIP55

MGNSSEEPKPPTKSDKPSSPPVDQTNVHVYPDWAAMQAYYGPRVAMPPYYNSAMAASGHPPPPYMWNPQHMMSPYGAPYAAVYPHGGGVYAHPGIPMGSLPQGQKDPPLTTPGTLLSIDTPTKSTGNTDNGLMKKLKEFDGLAMSLGNGNPENGADEHKRSRNSSETDGSTDGSDGNTTGADEPKLKRSREGTPTKDGKQLVQASSFHSVSPSSGDTGVKLIQGSGAILSPGVSANSNPFMSQSLAMVPPETWLQNERELKRERRKQSNRESARRSRLRKQAETEELARKVEALTAENMALRSELNQLNEKSDKLRGANATLLDKLKCSEPEKRVPANMLSRVKNSGAGDKNKNQGDNDSNSTSKLHQLLDTKPRAKAVAAG

>AtbZIP56

MQEQATSSLAASSLPSSSERSSSSAPHLEIKEGIESDEEIRRVPEFGGEAVGKETSGRESGSATGQERTQATVGESQRKRGRTPAEKENKRLKRLLRNRVSAQQARERKKAYLSELENRVKDLENKNSELEERLSTLQNENQMLRHILKNTTGNKRGGGGGSNADASL

>AtbZIP57

MNTTSTHFVPPRRFEVYEPLNQIGMWEESFKNNGDMYTPGSIIIPTNEKPDSLSEDTSHGTEGTPHKFDQEASTSRHPDKIQRRLAQNREAARKSRLRKKAYVQQLETSRLKLIHLEQELDRARQQGFYVGNGVDTNALSFSDNMSSGIVAFEMEYGHWVEEQNRQICELRTVLHGQVSDIELRSLVENAMKHYFQLFRMKSAAAKIDVFYVMSGMWKTSAERFFLWIGGFRPSELLKVLLPHFDPLTDQQLLDVCNLRQSCQQAEDALSQGMEKLQHTLAESVAAGKLGEGSYIPQMTCAMERLEALVSFVNQADHLRHETLQQMHRILTTRQAARGLLALGEYFQRLRALSSSWAARQREPT

>AtbZIP58

MNTIPAELTGYFHYLPPDKYNNQTPIMESEYFNMPSSPTSCSSFYHLNGLINNNNYSSSSNGQDLMTSNNSTSDEDHQQSMVIDERKQRRMISNRESARRSRMRKQRHLDELWSQVIRLRTDNHCLMDKLNRVSESHELALKENAKLKEETSDLRQLISEIKSHNEDDNSFLRELEDSISNSRSDSNQSGRDFELC

>AtbZIP59

MDKEKSPAPPCGGLPPPSPSGRCSAFSEAGPIGHGSDANRMSHDISRMLDNPPKKIGHRRAHSEILTLPDDLSFDSDLGVVGNAADGASFSDETEEDLLSMYLDMDKFNSSATSSAQVGEPSGTAWKNETMMQTGTGSTSNPQNTVNSLGERPRIRHQHSQSMDGSMNINEMLMSGNEDDSAIDAKKSMSATKLAELALIDPKRAKRIWANRQSAARSKERKTRYIFELERKVQTLQTEATTLSAQLTLLQRDTNGLTVENNELKLRLQTMEQQVHLQDELNEALKEEIQHLKVLTGQVAPSALNYGSFGSNQQQFYSNNQSMQTILAAKQFQQLQIHSQKQQQQQQQQQQQHQQQQQQQQQYQFQQQQMQQLMQQRLQQQEQQNGVRLKPSQAQKEN

>AtbZIP60

MAEEFGSIDLLGDEDFFFDFDPSIVIDSLPAEDFLQSSPDSWIGEIENQLMNDENHQEESFVELDQQSVSDFIADLLVDYPTSDSGSVDLAADKVLTVDSPAAADDSGKENSDLVVEKKSNDSGSEIHDDDDEEGDDDAVAKKRRRRVRNRDAAVRSRERKKEYVQDLEKKSKYLERECLRLGRMLECFVAENQSLRYCLQKGNGNNTTMMSKQESAVLLLESLLLGSLLWLLGVNFICLFPYMSHTKCCLLRPEPEKLVLNGLGSSSKPSYTGVSRRCKGSRPRMKYQILTLAA

>AtbZIP61

MAQLPPKIPTMTTPNWPDFSSQKLPSIAATAAAAATAGPQQQNPSWMDEFLDFSATRRGTHRRSISDSIAFLEPPSSGVGNHHFDRFDDEQFMSMFNDDVHNNNHNHHHHHSINGNVGPTRSSSNTSTPSDHNSLSDDDNNKEAPPSDHDHHMDNNVANQNNAAGNNYNESDEVQSQCKTEPQDGPSANQNSGGSSGNRIHDPKRVKRILANRQSAQRSRVRKLQYISELERSVTSLQTEVSVLSPRVAFLDHQRLLLNVDNSAIKQRIAALAQDKIFKDAHQEALKREIERLRQVYHQQSLKKMENNVSDQSPADIKPSVEKEQLLNV

>AtbZIP62

MELEPISSSCCSSSSSSSGEENTAAANMTEMEAAEALADLAQLAIMREQVFESAASWGSKGKRVRKRVKTESPPSDSLLKPPDSDTLPTPDLAEERLVKEEEEEEEVEPITKELTKAPVKSEINGETPKPILASTLIRCSRSNGCGRSRQNLSEAEREERRIRRILANRESARQTIRRRQAMCEELSKKAADLTYENENLRREKDWALKEFQSLETINKHLKEQVLKSVKPDTKEPEESPKPSQVEMSTSSTPFYFYNQNPYQLFCWPHVTQSSNPMISPLEFPTSGGASAKTITTQEHENAADDNGQKTHFYVVPCPWFLPPPDHSNGVPFGLQDTQRGTFSNGHHIDDSSARPMDVTETPRSHLPTRIKEEDSGSPETRPLYDLNESATEVLSEGGDGFPVTQQAYSLKHEDVSETTNGVTLMPPGHHVLISLPEKKHGSLAAAEARKRRKELTRLKNLHGRQCRMQVG

>AtbZIP63

MEKVFSDEEISGNHHWSVNGMTSLNRSASEWAFNRFIQESSAAADDGESTTACGVSVSSPPNVPVDSEEYRAFLKSKLNLACAAVAMKRGTFIKPQDTSGRSDNGGANESEQASLASSKATPMMSSAITSGSELSGDEEEADGETNMNPTNVKRVKRMLSNRESARRSRRRKQAHLSELETQVSQLRVENSKLMKGLTDVTQTFNDASVENRVLKANIETLRAKVKMAEETVKRLTGFNPMFHNMPQIVSTVSLPSETSNSPDTTSSQVTTPEIISSGNKGKALIGCKMNRTASMRRVESLEHLQKRIRSVGDQ

>AtbZIP64

MSLQRPNGNSSSSSSHKKHKTEESDEELLMVPDMEAAGSTCVLSSSADDGVNNPELDQTQNGVSTAKRRRGRNPVDKEYRSLKRLLRNRVSAQQARERKKVYVSDLESRANELQNNNDQLEEKISTLTNENTMLRKMLINTRPKTDDNH

>AtbZIP65

MQGHHQNHHQHLSSSSATSSHGNFMNKDGYDIGEIDPSLFLYLDGQGHHDPPSTAPSPLHHHHTTQNLAMRPPTSTLNIFPSQPMHIEPPPSSTHNKEGNRKGLASSDHDIPKSSDPKTLRRLAQNREAARKSRLRKKAYVQQLESCRIKLTQLEQEIQRARSQGVFFGGSLIGGDQQQGGLPIGPGNISSAEAAVFDMEYARWLEEQQRLLNELRVATQEHLSENELRMFVDTCLAHYDHLINLKAMVAKTDVFHLISGAWKTPAERCFLWMGGFRPSEIIKVIVNQIEPLTEQQIVGICGLQQSTQEAEEALSQGLEALNQSLSDSIVSDSLPPASAPLPPHLSNFMSHMSLALNKLSALEGFVLQADNLRHQTIHRLNQLLTTRQEARCLLAVAEYFHRLQALSSLWLARPRQDG

>AtbZIP66

MDSQRGIVEQAKSQSLNRQSSLYSLTLDEVQNHLGSSGKALGSMNLDELLKSVCSVEANQPSSMAVNGGAAAQEGLSRQGSLTLPRDLSKKTVDEVWKDIQQNKNGGSAHERRDKQPTLGEMTLEDLLLKAGVVTETIPGSNHDGPVGGGSAGSGAGLGQNITQVGPWIQYHQLPSMPQPQAFMPYPVSDMQAMVSQSSLMGGLSDTQTPGRKRVASGEVVEKTVERRQKRMIKNRESAARSRARKQAYTHELEIKVSRLEEENERLRKQKEVEKILPSVPPPDPKRQLRRTSSAPF

>AtbZIP67

MSVFESETSNFHVYNNHEIQTQPQMQTFLSEEEPVGRQNSILSLTLDEIQMKSGKSFGAMNMDEFLANLWTTVEENDNEGGGAHNDGEKPAVLPRQGSLSLPVPLCKKTVDEVWLEIQNGVQQHPPSSNSGQNSAENIRRQQTLGEITLEDFLVKAGVVQEPLKTTMRMSSSDFGYNPEFGVGLHCQNQNNYGDNRSVYSENRPFYSVLGESSSCMTGNGRSNQYLTGLDAFRIKKRIIDGPPEILMERRQRRMIKNRESAARSRARRQAYTVELELELNNLTEENTKLKEIVEENEKKRRQEIISRSKQVTKEKSGDKLRKIRRMASAGW

>AtbZIP68

MGSSEMEKSGKEKEPKTTPPSTSSSAPATVVSQEPSSAVSAGVAVTQDWSGFQAYSPMPPHGYVASSPQPHPYMWGVQHMMPPYGTPPHPYVTMYPPGGMYAHPSLPPGSYPYSPYAMPSPNGMAEASGNTGSVIEGDGKPSDGKEKLPIKRSKGSLGSLNMIIGKNNEAGKNSGASANGACSKSAESGSDGSSDGSDANSQNDSGSRHNGKDGETASESGGSAHGPPRNGSNLPVNQTVAIMPVSATGVPGPPTNLNIGMDYWSGHGNVSGAVPGVVVDGSQSQPWLQVSDEREIKRQRRKQSNRESARRSRLRKQAECDELAQRAEVLNGENSSLRAEINKLKSQYEELLAENSSLKNKFSSAPSLEGGDLDKNEQEPQRSTRQDVA

>AtbZIP69

MDKEKSPAPPPSGGLPPPSGRYSAFSPNGSSFAMKAESSFPPLTPSGSNSSDANRFSHDISRMPDNPPKNLGHRRAHSEILTLPDDLSFDSDLGVVGAADGPSFSDDTDEDLLYMYLDMEKFNSSATSTSQMGEPSEPTWRNELASTSNLQSTPGSSSERPRIRHQHSQSMDGSTTIKPEMLMSGNEDVSGVDSKKAISAAKLSELALIDPKRAKRIWANRQSAARSKERKMRYIAELERKVQTLQTEATSLSAQLTLLQRDTNGLGVENNELKLRVQTMEQQVHLQDALNDALKEEVQHLKVLTGQGPSNGTSMNYGSFGSNQQFYPNNQSMHTILAAQQLQQLQIQSQKQQQQQQQHQQQQQQQQQQFHFQQQQLYQLQQQQRLQQQEQQSGASELRRPMPSPGQKESVTSPDRETPLTKD

>AtbZIP70

MESSSVHRSHCFDILDGVPLHDDHFNSAFLPNTDFNVHLQSNVSTRINNQSHLDPNAENIFHNEGLAPEERRARRMVSNRESARRSRMRKKKQIEELQQQVEQLMMLNHHLSEKVINLLESNHQILQENSQLKEKVSSFHLLMADVLLPMRNAESNINDRNVNYLRGEPSNRPTNSPFASSTMIDAMYATSDYYSSNYLLAFVCRY

>AtbZIP72

MSFPVVATSFGVSQSGSQAGKKKGGYVNYEVEPGFTIRMRQNIDPTTDPKILKRIISNRVAAQKSRWKKVQYLDALVKRSMELQREVSELRSQLAITSEQKRYLENEQRQLKECISARVQHCINSDGVIEEYKTEIERLKTNLAPLSNLT

>AtbZIP74

MNGRGNMTQYQQNPFSTDDGGQSTGVSLSSRTSLSPPLIRYPAGSPDFSPGPRCTTQPSPTFSDFTQASPSLTSFNNPASFTPSFSFSNIHQMIPTPSSSHNSKASVSSASSSSFYFPQTSPSSCSTPSSFSPDSFSHSNTGPWSIPQPSPVFSSIAPASSALSSFGPDSFSHSNTGTWSIPQPSPVFSSIAPASSAPPLFGRDSFPRSNRGKGSLIHRSPVLSVLPPAPVYSSNPMVRSSPPGHPSPSAHLEEMSNRPPLHPQPRVPVTRSNSAKVSGSRPRKYHKRTNSELSSMLVGDSSRGEEGGFGKLIYNEEAMKEFCSEYMIMPNQSAVNNSDQNRNADVLMITNTDSGGANDAKKYKRMLANRASAARSKENREKKIRDMELRVETLENTQASLFGTMTLLEKENIVMMNENKLAKIRLQLLEQQAPLLTALTKQLDELRRLEKEANERGSVDYSQLLKQLKQSEELLAEINRFKVATGQGMKNPNQFEGSTMHQSDQNVFQPQLNTYEFNQHQQLDPNIFKEQCNVNEFNHEQPNHEFYGHN

>AtbZIP75

MFQQNLHDEDDVHVQDHVYNHDQYHHQQPNVVTDEEKRLRRMASNRESARRTRMRERMMKEGLQMQVKQLMAYNQFLYNKYISLLEYNHQILQENSQLKETVSSFHDQYTISYGNHEGILGNTNDFDLNQLSSYQPS

>AtbZIP76

MASSKGSQSVRNLMYPGKHALLPPKIPFPSVSASYSEYIPTGLIGSRHGQKLSNEKTHHQRTSSESHLVEELPFWLDDLLNEQPESPARKCGHRRSSSDSYAYLDVANATNISLTLQNDFSYRNTVLSTQRGVQELDRNKNAQDAAFYSGASFLKQKSRQRDSLVATGACPSWLPFARENGGGKNLGALYMSQDATVISSERKNYAEPFSHDPKMLSSEENNSNPSPVTYEADNTKRAKQQFAQRSRVRKLQYISELERNVQTLQAEGSKVSAELDFLNQRNLILSMENKALKKRLESIAQEKLIKQLEQEVLEKEIGRLRALYQQQQQTQKPSASRGRATSKDLDSQFSSLSLNTKDSNCRRDSVSVMGQFHF

>AtbZIP77

MENLRRLSNPGNFGFIGRSQSRVPQKNMENNISPPNNMHHHSASLDDLFTEDQPAWLDELLSEPASPKINKGHRRSASDTAAYLNSALMPSKENHVAGSSWQFQNYDLWQSNSYEQHNKLGWDFSTANGTNIQRNMSCGALNMSSKPIEKHVSKMKEGTSTKPDGPRSKTDSKRIKHQNAHRARLRRLEYISDLERTIQVLQVEGCEMSSAIHYLDQQLLMLSMENRALKQRMDSLAEIQKLKHVEQQLLEREIGNLQFRRHQQQPQQNQKQVQAIQNRYNKYQPPVTQEPDAQFAALAI

>AtbZIP78

MASSKGSQNHRNLGCNGKQALLPPKSPFTGGPTFSADFVPSSVIGSKAVQKLGEGNANHHRTSSESFLIEEQPSWLDDLLNEPETPVRKGGHRRSSSDSFAYVDVPVGFDVDYTLWDGGRYNNNNGFSNHVRGPKESDYLRSQPVPFYPSAHLSKQKIRPWDSLPDSGARPNSSSGCLESSSITRSGSSGSLRDTEKAYSAADSKKDFINNFAKSSFEKRDNPLAKSATSEADTKRARQQFAQRSRVRKIQYIAELERNVQMLQV

>AtbZIP79

MSRPAPLPPRCPIPKKLSLSPVADTFYSSSSPIESYIGQYKSSTQDSRLEDQPAWLDELLCDKTDGLLTRGGPLRRSASDSVVLLGDISATFSGFDQSEDEESLSSEACGDLESACVYGPNSPRAKNNSSFSNNPIASAFSDYGSQTPPQNLDDTVKGINRSPVAENACGSMGIPNAKRNPGQRSRVRKLQYIAELERTVGMLQTVEADLSVRVASLLQTRATLSLENSQLKQQMAILKQDKLIREGEYQLLKKEAQRLKSGLGYLGSTNNSNRLVRSYSAGSNVAPRTASSHLDWNLLDLTKLNLN

>AtbZIP71

MTISYPAKEFLQTIFKTPYLKNLYLSPTMSIYDTLIGSPATTLKRYLLKSDVAVMYTSPSTYVENEDSSFRFNQNCSPMPPWVLGTKSKTIINTLPPKKSHKRTNTDIIGIHSMTSQNSSGSSYFQLLDLLADLGELIYDDEAMKGFYYEFMNLDNKNAFNNDENLNNLMITFTNLGGASNANESGLKIGATTSGVKTKVDEDIEPLCRHYKCATMDSSFIHWITHWNLLDQVFSSNFVGEGEYYTDPNEVKRILENRDLDTRSKQGKSLYIVDLEGSVRIHVMVNTSLYGMIVLLEVRHITLAKKKNDVDDEREQENEDSS

>AtbZIP32

MNGSDNTTPSRPWSITQPSLAFSSLPPLSPSPSSSRRNSLPLMNPSTSMESRDSSMRFKKNSLLPPLGVKKIAPKDIGPLKRHYRSVSMDSCLSDLLKLTPSPGNTPSSRLVDGDQNASRLEFDANDYTDDELNKIAKSNKLKEVALDPKEVRRILKNRESAAHLKQKKLQYMINLEHRINFVENENASIFEKIKLLENDKTMMMNEKKEIMIRIESMEIQAQLRDVLTEHLHGESERLKAALISNENGNGKVQKLRMATCEVLQNRHEFDKSNMEVMDSNMINWSQPNPGFNG
