# Supplementary material for: The bZIP gene family in watermelon: genome-wide identification and expression analysis under cold stress and root-knot nematode infection
Source: PeerJ. 2019 Oct 16;7:e7878. doi: 10.7717/peerj.7878 (PMC6800529; doi:10.7717/peerj.7878)
Supplement: Supplemental Information 5 [file peerj-07-7878-s005.doc]

**Table S3.** The CDS sequences of *ClabZIP* genes.

>ClabZIP2

ATGGCTTCCTCCAATGTGGAGATGGCTTCCACGTCCAAGACCAATCCGGATCTCCCGCGCCAGTCCTCTGTTTGTTCTATCTCCACAATTATCGCTGATCTACACCATACTGATCCCTCCCGGAATCTGGCTTCCATGAGCATGGATGACCTGCTTAAGAACATCTATTCCGATGCTCAAACTCACAATCAAGTTCAGGATGAAAATCCTATTATTGGCTCTTCGATCTCGCCTCAAGACACTTTTCCGCTGCCTAAGGAATTGAGCACCAGAACTGTGGAGGAGGTCTGGAAGGAGATCGTTGCTGGGGGTGATCAGCAGAGGCGGGAGTCTGCTACGGATCAGGAAATAACCCTTGAAGATTTCTTGTCTAAGGCTGGGGCTGTTTGTGACGACGATGTGAGGGTTCCTGTCATTTCTGAACCTGGGGGTTTTGCTGTGGACTCCACGCTTAATAATCAGCTTCAGATTCCGTCGCAGCAGTTGGAAGGTCCCATGGTGGGAGGATATGCGAGTGGAATCGATGGGAGAATCGTTGGAGTTGGAAGAGGGAAAAGAAGGGCTGTTGAGGAACCAGTTGACAAGGCCACTCAGCAGAAACAGAGACGGATGATCAAGAATAGGGAATCCGCTGCTCGTTCTCGGGAACGCAAGCAGGCATATACGCTAGAGCTAGAGTCTCTTGTGACCCAACTGGAGCAGGAAAATGCAAGGCTATTGAGAGAAGAGTATTGGCCACTCAAGGAGATGAGTCTCAAGTGA

>ClabZIP1

ATGGAAATCTCAGGAACCCATTTTGCAGCACCTGAAAATTATGGCATGATTCAAAATCTCAATATCAATGAATTATTGTCTTATATACCTGGTAATTCAACCTCTACTTCTGATGATGGTGATGATCTGAACCACAAACTTGGAGTTGTCATCGACGAGAGGAAGCGGAGGAGAATGATATCGAATCGGGAATCGGCGCGAAGGTCCCGGATGAGGAAACAGAAGCATCTTGATGAACTTTGGTCACTAGTGGTTCGTCTTCGTATGGAGAATCATAGTCTTATGGAGAAGTTGAGTAAGTTAACAGATTCTCAACAACAGCTTCTTCAAGAGAATGTCAAGCTCAAAGAAGAGGCGTCGGATTTTCGTCGGATGATCACTGACATTCAAATGGGCAGTCCTTACATAACTCACTTGAGAGAGCTGGAGGAAGCCCCCTGCAATACCTTTGATCTCATGGCTGAATCCTCAAGCCAATCTAACGAGTAG

>ClabZIP3

ATGCTGTCCACTTTTCCCACCGCCTCGGCGTACGAGTCCATGCTCGAAAACCCATTTCCGGCGGTTAATGGCACTTCCACGCCGTGGGAATGCCATGATCACGATCACGACCCCGAGTTTTTGCCGGTTGTGTCCCAGATGACGGAACCAACGGGATTATTTGACGTTTTCCACTCCCCAAACCCGGTGATTTCCAGTTCGAGTTCCGAAAACCGGGACGAACCGGAACCGATGGATGCCGGTCCGTGTGCCCCGGACCGGAAAGTTGAGGTGATAGACGAAAGAAAACGACGGCGGATGGAGTCGAACCGGGAGTCGGCGAGGCGGTCCCGGATGCGTAAACAGAAGCACTTAGAAAACTTAAGGAACTTGGTGAACAAGCTGAAAGTAGAAAACCGGGAGCTGTCCAACCGGTTGCGGTTCACGGTTTATGAAGTGAACCGTGTACGGACAGAAAACGACCATCTGCAGACAGAACACACAATTCTGAGGCAGAAACTTTCGTATAGCCGACAAGTATTAATTTTCCGGCAATTCCAACGACAATTGGCTTTTGCTTCCCCTTACAGCGCCCTTGAACAACCGCCAACTATCTCCCAATCGCAATCGCAATATATCCTTAATAACAACAACAACAACCTCATTAATTGCTAA

>ClabZIP4

ATGGGGACTGGGGAAGAGGGCACACCTTCTAAAACTTCCAAACCTCCCTCGTCATCCCAGGAAATAGCTCCAACACCTTCATATCCTGATTGGTCAAGCTCAATGCAGGCTTATTATGGTGCTGGTGCTACTCCACCTCCCTTCTTTGCATCTACTGTTGCTTCTCCAACTCCCCACCCTTACCTCTGGGGAGGTCAGCATCCTTTGATGCCACCTTATGGGACTCCAGTACCTTATCCAGCTATATATCCTCCTGGGGGAGTTTATGCCCATCCCAATATCACCGTGACTCCTGGCTCTGTACCAATTAATGCAGAATATGAAGGAAAATCCCCTGATGGAAAAGAAAGGGCCTCAAAAAAATCCAAGGGCATGTCTGGAAATACTGCTTCAGGTGGTGGTAGGACCGGGGAGAGTGGAAAGGTGGCTTCAAGTTCTGGAAATGATGGTGCTTCTCAAAGTGCTGAAAGTGGTACCGAGGGCTCATCAGAAGGCAGTGATGAAAATGCTAACCAACAGGAATTTGCTGCAAATAAGAAAGGAAGCTTCAACCAGATGCTGGCAGATGGAGCCAATGCACAAAACAACACGGGCGGACCAAATGGCAAATCTTCAGTGACTGGGAAACCCATCACCTCCATTCCTGGGACTAATCTGAACATGGGAATGGATTTGTGGAATACCACCACTGCAGCTCCAGGGGCTGGAAAAGTGAGAGGGAATGCCGTCTCCTCAGCTATTGTTACAGTACCGATGGTTGGTCGTGATGGTGTGATGCCTGAACAATGGGTTCAAGACGAACGCGAGCTAAAAAGACAGAAAAGGAAGCAATCTAACCGAGAGTCTGCCAGGAGGTCGAGATTACGTAAGCAGGCGGAGTGTGAAGAGCTGCAGGCAAGAGTGCAGACATTGAACAACGAGAACCGTACTCTTAGGGATGAGTTACAAAGGCTCTCCGAGGAATGCGAAAAGCTAACATCAGAAAATACTTCCATCAAGGAAGAATTGACACGATTTTGCGGGCCAGAGGCATTAGCTAACTTCGAAAAAGGAACCACCGCGCCGCCTGCTCAATCTCGAGGCAGCGGCGAGGGCAAGGATTAA

>ClabZIP5

ATGGCATCTTCAAATGGAGCGTCATCTGGGTCGGCCAACGGAAGCATCCGAACCTCAGGTTCGGAAGATGATCGGGAGGTTGAGAGGAAGCGGCGGAGAATGCAATCTAATCGGGAATCGGCTCGACGATCTCGATTGCGCAAACAAAAACATTTACATGATCTTACTCACCAGGTGAGTCAACTGAAGAAGCATAACAATGAGATGGTGACGAACATGAACGTGATGGCGAATCTGTGCGTCAACCTGGAGGCAGAGAACTCGATCCTTCGAGCTCAGATGGCGGAGCTTACTCACAGATTGCGGTCTCTAAACGATGTCGTAATGTTTATAAACTCCATGCAGGTTTTTGAAACATTTTTAGTCGATGAGGTTGGCGATTTGAATAATGGGTTCGAAGAGGAATATCATTACAACCCTTGGAGATATCCATTTGCAAATTACAACTCCAACACACAAATAATTGCCCATAAAGATTGA

>ClabZIP6

ATGGCACAGTTGCCACCCAAAATCCCAAATATGACGCCTAATTGGCCCGACTTTTCCCGGCAGAAAATCCCTTCAATGGAAACCTTTGCACCGCCCACCACCACGCCGGCGGTTACTCAAAATAATCCTTCTTGGGTCGATGAATTTCTCGATTTTTCTTCTGCTAGACGTGGATCCCACCGTCGGTCTGTTAGTGATTCCATCACATTCCTCGAAATGCCAATGCTCGAGGAAGATTGCCGGACTGCCGCTGCTGCACCGCCCCCGCCGCCCCCGGGATCCGGTGACCGGAATGAATTTGATAGATTCGATGACGAGCAATTCCTGTCGATGTTTAACGATGAAATCTCCGCCGCCGTCGCTCCCACCTTGTCGTCGTCGAACCCCTCCACGCCGTCGGATCATAATAGTATTAACGACGAGAAAGACGCCCAAAACGACGGTAAGCAAAATCAGAACAAAAACGAATCCGACGAAGTACAGAGCCAACAGCAATCGGAAAACCAAACACAATCCAATTCCATGGCGACGGCGGCAACGGCGGCTTCCACTGATCGGATAACAGACCCCAAAAGAGTCAAAAGAATTCTGGCAAATCGGCAATCGGCACAAAGATCACGAGTAAGGAAGCTTCAATACATATCAGAACTCGAACGAAGCGTTACATCGTTACAGGCTGAAGTATCGGTGCTATCGCCGCGAGTGGCGTTTTTGGACCACCAGAGATTGCTTCTTAACGTCGACAACAGTGCTCTGAAGCAAAGAATCGCCGCCCTTGCACAGGATAAGATTTTTAAAGATGCTCATCAAGAGGCATTAAAAAGGGAGATAGAGAGACTAAGACAAGTATATCACCAACAAAACATTAAGAAGATGGAAAACAATGCAGCACCATCACCGGCGATTACTCCGTCCCGACCCGTCACCGCCATCGCCACAGATGCCAAACTTCCTAACATAGATCAAAACGAGCAAGTTCCGAACATCGTCGTTTAG

>ClabZIP7

ATGGACGACGGGGAGCTTGATTTTTCGAATCAGGAAGTGTTTTCCAGCCCTAATATGGAGATTCCAAGCAGTTGTTCAATGGATAGTTTCTTTGATGAACTTCTCAAAGACACTCATACTTGTACTCATACACATACTTGCAATCCACCAGGCCCTGACTACTCTCATACCCACACATGTTTTCATGTCCACACTAAAATTGTCCCTGCCCCATCTGAAGAAGACAAGGTTGTTACTGATGACACTGCAGAATCAACTGAGAAGAAGTCGAAGAAACGCCCGTTGGGTAACCGTGAAGCTGTTCGTAAGTACCGCGAGAAAAAGAAAGCTAGGGCAGCCTCATTAGAGGATGAGGTTGTTAGATTGAGGGCATTGAATCAGCATTTGATGAAGAGATTGCAGGGGCAGGCAGCACTCGAGGCTGAGATTGCTAGGCTCAAGTGTTTGCTTGTGGATATTAGGGGCAGAATCGAAGGGGAGATTGGTTCTTTCCCTTACCAAAAAGCTGTTAATCCTAACTTGTCCAACCCAAGCATCCCTGGTGCTTTTGTGATGAACCCATGTAATATGCAGTGTGAGGATCAGGTTTACTGCCTCCATCCCGGGGTCGATGGCAACAGAAGCAGTGAAGGTGCTGTGATAAATGGACAAAGCTTTGGTGCTTGTGAATTTGAAAATCCTCAATGTTTGGCAAATCATGATTCGGGATCAAAAGAACTACCTGGGTGTGGAGTAGGGAATGCAGTTTCAAATGATATTTCTTCAGGCAAGAAGAAAGGTGAAAACTGA

>ClabZIP8

ATGGGTGATACTGAAGATGCTAACACTGAGAACATGCGAAATCTTCAATGTTCGTATGGAGTATCTTCTTCTTCAGCTGGAAATCTTCCTTTCTCGATGGATCAACTTAAAATTTCCCAAATGAACTGCTCACAAATCCGTCCACCGCACTTTCAGTCAAATTTTCTTGCGGATAATAGTAGAAGAATTGGGATACCTCCTAGCCCCAACTCACCGCAGATCCCACCAATTTCACCGTATTCTCAGATCCCGATCTCGCGTCCGATGAATCAGCAAAATTATAACCCGGTGCCTACTCATTCCCGATCGTTGTCTCAGCCTTCTTTTTTCTCTCTCGATTCTTTGCCCCCTTTAAGCCCATCTCCATTTCGTGAATCCCCAACTACATCGAATTCAGATCAGGTTTCTGCAGATACATCAATGGAGGATAGGGATAACAGTTCACATTCTTTGTTGCCTCCCTCACCTTATATGAGGGCCAATTCTTCTAAGATGGGAGATTCTTTACCTCCTCGTAAAGCACATAGGCGGTCTAGTAGTGATATTCCATTTGGATTATCTTCAATGATTCAGCCATCTCCTCTTCTCCCATTCAATAGCTCTGGTGGATTGGAACGATCAATTAGTAGTAAAGAGAATGCTGGCTTATTAAAGCCCGCCAGCCAGTTTGTTAAAAGAGAACCTAGTTTGGAGAAAAGCGTTGACAACAATTTAGAAGGAATGGGTGAGAGGAAATCTGATGGGGACAGCGTGGACGACTTGTTTTCTGCTTATATGAATTTGGATAATATCGATTTGTTCAACTCTTCTGGGACTAACGACAAGAATGGTCAAGAGAATCGAGAGGATTTGGATAGTAGGGGTAGTGGAACAAAGACAAATGGGGGTGAGAGCAGTGATAATGAAGCAGAAAGCAGTGTAAATGAAAGCGGGGATAGCGCTCAAATGCCTGGATTGAATTCATCTGCTGAGAAGAGGGAAGGGATCAAACGGACCGCAGGGGGAGATATTGCTCCAACTACCAGACATTACCGAAGTGTCTCCATGGATAGTTTCATGGGCAAGTTGCAGTTTGGTGATGAGTCTCCCAAAATGCCTCCCACACCACCTGGCGTTCGCCCGGGGCAACTTTCTTCAAACAACTTAGTTGATGGTAATTCTGCTCCATTCAGTTTGGAGTTTGGTAATGGTGAGTTCAGTGGGGCTGAACTGAAGAAAATTATGGCAAATGACAAACTTGCCGAGATTGCTTTGACTGATCCCAAGCGTGCAAAGAGGATCTTGGCAAACCGTCAGTCTGCTGCTCGATCGAAAGAGCGAAAGATGCGGTATATATCTGAGTTGGAACACAAGGTTCAGACTCTTCAGACAGAAGCTACCACACTGTCCGCTCAACTCACACTTCTACAGAGAGATTCCGTGGGCCTTACAAACCAGAATAATGAACTGAAGTTTCGACTACAAGCCATGGAACAACAAGCACAACTACGGGATGCTCTTAACGAAGCCTTAACCGCGGAGGTTCAGAGATTGAAACTCGCTACAACAGAGCTAAATGCGCAGTCTCATCCCTCCAACGGAGTAATGCCCCAATCTTCCATCAATCACCATGGCCTACAGCTTCAGCTTCAGCACCAGCAACAACAACAGATCCAGCAGAATGGGAATGCAACCACAAAACCAGAATCCAACCAATAA

>ClabZIP9

ATGGGTGATACTGAAGATGCTCGTACTGATAACCTACGAAATCTTCAATGTTCGTTTGGAACATCTTCCTCTTCTGCTTTAAAGCATCATTTCTCTATGGATCAACTTAAAATTTCTCAAATGAACTGCTCACAAGGCCGTGCACAGCATTTTCAGTCGAATTTTCTTGGAGATAATAGTAGAAGAATTGGGATACCTCCTTGTCCCAACTCACCGCAGATCCCACCAATCTCACCGTATTCCCAGATCCCGGTGTCGCGTCCGATGAACCAGCAGAGTTATAACTCAGTTCCTACTCATTCTCGATCGTTATCTCAGCCTTCTTTTTTCTCTCTCGATTCTTTGCCCCCTTTAAGTCCGTCTCCATTTCGTGACTCCCCTTCTACATCGAATTCAGATCAGGTTTCTGCTGATACGTCAATGGAGGATAGGGATGCCAGTTCACATTCTTTGTTGCCTCCCTCACCTTATACGAGAGCCAATTCTTCGAAGATGGGTGATGCTTTACCTCCTCGTAAAGCCCATAGGCGTTCTAACAGTGATATTCCATTTGGATTATCTTCGATGATTCAGTCATCTCCTCTTCTCCCTTTTAGTGGTTCAGGTGGATTGGAGCGATCAACTAGTAGTAAAGAGAATGCGGGAATATTTAAGCCGGCCAGCCAGTTTGTTAAAAGAGAGCCTAGTTTGGAGAAAAGCATTGATAACAATCTGGAAGGAATGGGTGAAAAGAAGTCTGAAGGGGACACTGTGGATGATTTATTCTCTGCTTATATGAATTTGGATAATATTGATTTGTTCAACTCCTCAGGGACCAACGACAAGAATGGTCATGAGAATCGGGAGGATTTGGATAGTAGAGGTAGCGGAACAAAAACAGGGGGTGAGAGCAGCGATAATGAAGCAGAAAGCAGTGTGAACGAAAGTGGGGATAACTCTCAAGTGCCTGGATTGAATTCGTCTGCTGAGAAGAGGGAAGGGATTAAACGGACTGCAGGGGGAGATATTGCTCCAAATAACAGACATTACCGGAGTATCTCCATGGATAGTTTTATGGGCAAGTTACAGTTTGGTGATGAGTCACCGAAAATGCCTCCTACGCCGCCCGGCATTCGTCCAGGGCAACTTTCTTCAAACAACCTAGTTGACGGTAATTCAACTCCATTCAGCTTGGAGTTCGGTAATGGTGAGTTCAGTGGGGCTGAACTGAAGAAAATTATGGCAAATGACAAACTTGCGGAGATTGCACTAACCGATCCCAAGCGTGCAAAGAGGATTTTGGCTAACCGTCAATCTGCTGCTCGATCAAAAGAACGGAAAATGCGGTATATATCTGAGTTGGAACACAAGGTTCAGACTCTTCAGACAGAAGCTACCACGCTGTCTGCCCAACTCACGCTTCTGCAGCGAGACTCTGTTGGACTTACAAACCAGAATAATGAACTTAAGTTCCGTCTCCAAGCCATGGAACAGCAAGCACAACTACGGGATGGTATTGACAATTTCTCTTAA

>ClabZIP10

ATGAATTTCAGGAACTTTGAGGATATGCCACCAGGAGAAGGCACTATGGCTAAGGCACAAGGAAATTTCACATTGACCAGACAGCCTTCCATATACTCTTTAACCTTTGATGAATTTCAGAACACTTGGAATGGACTTGGAAAGGATGTTGGTTCGATGAATATGGATGAACTTCTAAAAAACATATGGACTGCCGAAGAGTCTCAGGCAATAACATCTACAGGTGCTGTTGCAGGCGGAGTTGGAAGCACCAATGCTGGGAACTTGCAGAGGCAAGGTTCATTAACTTTACCCCGGACCATTAGTCAGAAAACGGTTGATGAAGTTTGGAAAGACTTGAGTAAAGAGAATACCAGTGTTAAAGAGGGAAATGGAATTGAAGCCATGCCTCGACGACAACCGACATTAGGGGAAGTGACTTTGGAAGAATTTTTAGCTAGAGCAGGGGTGGTAAGAGAAGAACCTCCACATATTGAGGAGAGGCCATTTAACTGTGGATTTTATGGTGGATTATCAAGAGAAGACAACAATGCTGGTTTGGCTCTTGGGATGTTCATGGGAAATCAGATAGCTGAGAACAAAAGTATGGTTTCAAATCAGAATCAAAATTCAGTGTTTTTAGGGACCGGAGTTGTAAGGTCTTCTCAGCAGCAGCAGCAACAGCAGCCACTTTTTCCCAAACCAGCTAATGTGACTTTTGCTTCTTCAATGAATTTAGTGAACAATCCCCAGCTTACTAATGGATCTGGGACTAATTTGGTTGTGGCCCCAAAACCTCCTTTACATGAGGCTTTAATTCAGGGCAGTGGCATCGGTGCAATCGGTTTAGGCACGAGAGGTGTAACTGTTGCTTCAAGATCTCCTACAAGTACCATATCATCTGATGTGATTACAAAAAGCAGCATAGAGACGTCTTCATTTTCACCCGTTCCATTTTCATTTGGCCGGGGAAGGCGAAGCAGCGGAGCTCTTGAGAAAGTTGTTGAGAGGAGGCAACGAAGAATGATAAAGAACAGAGAGTCAGCTGCAAGATCACGGGCTCGTAAACAGGCTTATACCTTAGAACTTGAAGCAGAAATTGCAAAACTCAAAGAAATGAATCAAGAGTTGCAGAAGAAACAGAGAGAGATAATGGAAACGCAGAAAAATCAGGTTTTGGAGAAGATGAAATATCAGTTGGGTGGCAAAAGACTCTGCTTAAGAAGAACACTGACTGGCCCTTGGTAG

>ClabZIP11

ATGGAAGACGGGGAGCTTGAATCCTCGAACCCTGAAGTGTTTTCGAGTTCGAATGCTGTTGAGCTTCCGAGCAGTTGCTCAATGGATAGCTTCTTTGATGAAATTCTGAAGGACACTCATGCTTGCACACATGCTCATACTTGCAACCCTCCTGGTCCTGATTATTCACATACACACACCTGTTTTCATGTCCATACGAAAATTGTCTCTTCCCCCACTGAAGAAAAGGTTTCCACTGACGACACAGCGGAGTCTGTGGACAAAAAGAACAAGAAGCGGCCGCTTGGTAATCGTGAAGCAGTTCGTAAATACCGGGAGAAGAAAAAGGCGAGGGCTGCATCATTGGAAGATGAAGTTGTGAGATTGAGGGCCCTGAATCAGCAACTGTTGAAGAGATTGCAAGGTCAGGCTGCATTGGAGGCTGAGATTTCCAGGCTCAAATGTTTGCTTGTGGATATTCGGGGAAGAATTGAAGGGGAGATTGGAACGTTTCCATATCGGAAACCAGCCAATTCAGATCTGCCCAATCAAAATGTGCCGGGTAGTTACATGGTGAATCCATGTAATGTGCAATGCAATGATCAAGCATATTGCCTTCACCCAGGGGACGATGGAAAAAGTGGAGAGAGCGTTTCGTTGAATGGGCAAAGTTTTAGTGCGTGTGACTTTGAAAATCTTCAGTGCTTGGCAAATCAAAACACGGGAGTAAAGGAACTTCCTGACTGTGGACTAGGAAACACAATTTCTAACGTTAATTGCTCAGAATTAAATCCGAAGAAAGGTAAAACCAGAGCACTGAACTTTTAA

>ClabZIP12

ATGGATAGGGTGTTTCCAGTGGGTGAAATTTCTGACCACTACTGGTCGTCGGAACCTGCCGCCGGTCCTCCGCCCCCACCGGCCGACGAAGGCTCCAAAATGAACCGTAGCGCATCGGAGTGGGCCTTTCAGCGCTTCCTTCAAGAAGCCTCTGAGACTTCCCCTCATTCCTCTGCTGCAGACCATGGGGAAGTCGTCGAGATCAAGGACTCTGCCTATAATCAATTACAGAAACTTAATGCTAATCAAGTTGGTGTTAGTAATTGTAATACCAGCAGCATTTCTTCCAATGCTGTGCCGCCGAATATTCCGATCGATTCTGAGGAATATCAGGCCTTCCTTAAGAGCAAGCTTCATTTGGCTTGTGCTGCTGTCGCCATGAAGAGGGGATCCTTTAGAATGACTCCAGCTTCGTCCACCTCAGCTGACTGCGGATCACAAGCATCTAATACGTTGGGAATTCAAGCTCCTAAAGCTTCTAATATAGGAGCTGGGAATAATTCATTAAGGTCACCAGATAAAGATATAAATGGGGCCGCTGGAGTTACTTCTTCATCCGTGGTGCCAAAAATACCTGAGCTTCGAGCTCGGCCAGCTACTAGTGGATCATCAAGAGATCTGTCGGATGATGAGGAGATTGAGGGAGACACTGAAACAAATGAAAGCAAGGACCCTGCTGATGTAAAACGTGTAAGGAGAATGCTTTCAAACAGAGAGTCAGCTCGACGATCAAGGAGAAGAAAACAAGCACATTTAACTGAACTTGAGACACAGGTTGCCCAATTAAGAGTTGAAAACTCTACCTTGTTGAAGCGCCTTGCCGATATAAGTCAAAAGTACAATGAAGCCAATGTTGATAATCGGGTTCTAAAAGCTAATGTTGAGACATTAAGAGCAAAGGTAAAGATGGCTGAAGAGACTGTCAAGCGAGTTACTGGTAACCCAATGTTCCATGCAATGTCTGAGATCTCCTCCATTGGCATCTCTTCATTCGAGGGTAGCCCTTCCGATACATCAACAGATGCTGCCGTTCCTTTGCAAGATGATCCATGTCGTCATCTATATCAATCAACATCTAATAACCCCATGGGCCCACACGACATAGTAGTCAATAATGGATTGGCTAACATTTCGCAGGTGGGGAGTGGGCAGCAGAATTCTCCATCTCAGGTACTGCCAGCCACATCGGGGAACAAGACCGGAAGATCGGAGTCTCTGCAGAGAGTGGCTAGCTTGGAGCATTTGCAAAAGCGGATCTGTGGAGTCAAAGCAAATGCAGAACAGTAG

>ClabZIP13

ATGCAAGATCCAGCGCCATCAAACCCTATCCACACTCCCAATTCCAATCAAATTCCTCCACTCAATGTTGCAGCACCGCCGCCGAAGACTCACAAACCGCCGCCGGTGGCGAATGCGACGGCCTCCTCTTCATCAATGTTTGCCAATGCGAATACTGGCAATACCTCATTTATGCCTAGAGTAGGCTCTCATCATCGGAGAGCTCATTCAGAAGTTAGTTTCCGGTTGCCGGAGGATATGATGGATATATCCGCTCTGGAGGAGATCGGATCGGAAGATGATCTGTTTTCGACCTACATTGATGTGAAGAAGCTTGGAGGTAATGGAGGAGGGAATTTTGTAGATCATAATGGTAATGGAGGAAGTGAAGGTGCTGGTGGTAGTGAAGGGGAGAAGACTTCGAAGCCAAGGCATCGGCATAGTGTTTCGGTGGATGGAACGACGTCGTCGTCGAGTATGTTTGGGGAAATTATGGAAGCGAAGAAAGCTATGCCTCCTGATAAGTTGGCGGAGCTTTGGTCCAGTGATCCCAAACGCGCTAAAAGGATATTGGCAAATCGACAGTCTGCTGCTCGCTCAAAAGAGAGGAAGGCAAGATATATACAAGAATTGGAGCGCAAAGTGCAAACCCTTCAAACAGAAGCAACTACTCTATCAGCCCAATTGACTTTGTTCCAGCGAGACACAACGGGTCTCAGTACTGAAAATACAGAGCTTAAACTTCGGTTACAGGCTATGGAGCAACAAGCCCAATTGCGTGATGCTTTGAACGAAGCATTAAAGAAGGAAGTTGAGAGGCTTAAAATCGCTACTGGGGAAATGATGAGCCCATCTGAGTCTTTTAACTTAGGAATGCATCACATGGCGTACGCCCCGTCATCTTTCATTCAACTTTCACAGCAACAACCAGGATCTGTTGGCCTTCAAAATATGCAAATGCCACCATATAGCCATTCTCCATCCAATATGACTACTCACCCTCTGCTTCCATCAGAGTCTCATTCACTCTCTGAGGTTCTGCAGACCGACTCTCTTGGCCGATTGCAGGGTCTCGACATCAGTAGTAAAGGATCGTCGCTTGTGAAATCCGAGGGTCCTTCACTCTCTGCTAGTGAAAGCAGTACAACCTTTTGA

>ClabZIP60

ATGGAGGAAAGTTACAAAATGAGGATGAACCAAGGCGGAGGAATTCCGACGATGGCAGCAGCACTGCCACCATTGCCACCGTCATGTTTAGGAAAACTGACAACTAGCGGTGAAAAAAAGCTACCGTTCTTTCAATCCAACATGAATTTAAGTATGTATGGAAATGACAAAAGTATCCTTTCTCAAAGAGAAGCTACCATAACACCACCGCCGAAGCAACACCAATCACAACTTCTAGACTCCGACAAAGATCTCACTGTCGAAGCCAAGCGACTAAGAAGGGTGATGCAAAGCAGACAATACTCTCAAAAGTATCGACTAAAACAGCTTCATTATATTACTCAGCTTGAATCAGAACTAAAAGCCCTTCAAGCAGAAGTAACAATTACCACACCGAGGATAAAATTCATGGACCGTCAAAATTCACTGCTCCGAGCCGAAAATTACTCCATCAAAGAGAAATTATCTGCATACACCGGAGAACTTCTATTCAAAGAAGCTCAATACGAAGAATTGAAAAGAGAGAGAAATATGCTTAAGGAAATCTACGAAGCATATCAGTTAAAATTGCTGGAGACTCTGAAAAGCAGCAACAACAACAACAACACAACTGCTGCCAGTGGAAGCACTTTTCAATTGGTCGAAAATTACCCACAAATTGCTACCAAATCAAACCCATTTACCATGCTCGAAAATTAA

>ClabZIP14

ATGGGGAGCCAAAGAGTTGAAGAAACTGGTCCTTCATCCCACTATCATCATCACCTTCCATATAATTCACTTCTTTATGGAATCAACAACAATAATCTTTCCTCCACTTTGATCAATCACGATGGACAATCTTTTGATTTTGGAGAATTGGAACAAGCAATTGTGCTACAAGGACTCGGAACCAAGAACAAACTCGATCATCACGAACCTAAACAAAGCTTTTTCTCAGGCAAGCCTGCAGCAACTCTGGAAATGTTTCCTTCTTGGCCAATCAAACACCAACAAACTTCAAGAGGAATTAGTTTGAGAAGAAGAGAAGAGAGCAGTGATGAGTCAGGAGCAGCTGTGAACACTCATACAATAAACAAATCTAATAATCAAGAAGGGCATTTGGAATTGGAAATAGAAAAAGAAGAATCTGAGATGAGTAAAAAGGGTTGTTGTTCATCTTCTTCTTCAGGTCAAGATCAGAGCTTTATTCAAAAGCATCTACAGCAGATTCAACCTGAAATGATACTCAGTGATATTTCAACAACTGCACTTTCATCTCAACATCAATCCCTTCAACAAAAGAGAAAAGGATGTGGTTCCATTTCAACATCACAGAAACAACTTGACGCTAAGACATTGAGACGTTTAGCTCAGAACAGAGAGGCTGCGAGGAAGAGTCGACTAAGGAAAAAGGCTTACGTACAGCAACTCGAGAGCAGTAGAATCAAGCTTACGCAGCTTGAGCAGGACCTTCAACGGGCACGATCTCAGGGTTTGTTTTTAGGTGCCGGCGGTACTGGCGGCGGCATTGTCAGCCCCGCTAGTGCAATTTTTGATATGGAATATGTAAGGTGGTTGGAAGAGGACCATCGACATACAGTGGAGCTGCGTGGGGGACTAGAGGCACATCTGCCTGACGTGGATTTGAGAGTGAGAGTAGATGCATGCATTTGCCATTACAACGAAATTTTTCGACTCAAAGGAGAGGCTGCTAAGTCCGACATCTTCCATCTCATCACCGGAATCTGGATGTCTCCGGCGGAGCGTTGTTTCCTCTGGATCGGTGGATTTCGACCCTCCGATCTCATCAAGATGTTAATGTCACAATTGGATCCAATAACAGAACAACAAGTGATGGAGATTTACAAGCTACAACATTCGTCACAACAAGCTGAAGATGCTTTATCTCAAGGACTTGATCAGCTCCATCGGTCTTTGACCGACATCATCGCCGGCGGTCCGATCATCGACGGCATCAACCACATGGTTTTGGCCATGGACAAGCTCTCTAGTCTCCAAGGCTTTCTCCATCAGGTAATTATTACTTTCTTTTGA

>ClabZIP15

ATGGAACTGTGTTCGGTGCCTGTTTCTACTTATCCGCCGATTACTGTCGGTCCCGACAACCGTTGTTTAGTCAACGATTCGGTGCGGACTACTGTCAGTCCCGACAACCGTCGTTTACTCAAGGATTCGGTTACGGACTGGCGGAGATCCTTTGTGGTTGGCTGCCGGAGGGAAGATGAAGAAGGGGGGAGGGCAGTGCAGCTGCCTCCTTTCAGCAAGATTGGCGGCGGAGAGATGAACTCTGGCGGTGACGGTGCCGAGAATGTCGACGGCAGAAATCCCGAGCAGATCTTGTATGGTCGTAATATTGAGCCTAATATGGATCCAAGGAAACTAAAACGAATAATGTCGAATCGAGTCTCAGCTCAAAAATCTAGACTTAAAAAAGTTCAATATGTAGCAGATATGGAAAGGAAGCTGAAAGCTCTAGAGGCACATATAGCAGTTTTATCTCCTCAAGTAGAGCTGTACAGAAACCAACAGCAAGTGTTGCAAATGGAGCAAAAGAGGCTGAATCAGAAGATTTTGAATTGCTCAAGGAACAAGCTTTTGAGAGACGCTGAAATAGAAGAGAATAGAGCTGAAGTGAATAGGCTGAGGGAGCTCCATATGAAGCAGCAGTGTGAAGCAAATGGATGGGATAGTAATGTTTTCACGATGCCACCAGATCTTCATGCATCTGAGCTCAGCAACATCGTCTCTCCCCAACCAACCCAAACATAA

>ClabZIP16

ATGGGGATCCAGACGATGGGTTCTCAATTGAACGGCCAACAATCTCATTTACAACCTGCCCCATTGACAAGGCAAAACTCATGGTACGGTCTTACTCTTGATGAGGTTAAAAACCAGTTAGGTGAAATGGGGAAGCCATTAGGCAGCATGAACCTTGATGAGCTTCTTCATAATATCTGGACAGCTGAAGCCAATCAATCCATGGGAATGGAGAGTGAGAGTTCCTCTTCAGTATATTCTCTCAAACGTCAGGCCAGTTTCAAACTGGCCAGAGCATTGAGTGGGAAGACTGTTGATGATGTGTGGAGGGAGATTCAACAAGGGCAGAAGAAAAAAAATCACGAAAATTTGAAAAGTGAGAATAGTGAGATTATACTTGGTGATATGACTTTAGAGGATTTCTTGATACAAGCAGGGATTTATGCTGAGGCTTCTCCAAGTCCCATTATGGGCTTGGATGCCATTGATACTATGGCATTGGCAGAGAAGAATTTTTCACAGAAAATGGGCTTGTTGTCATCATCCCCTTCACTAGGCACACTGTCAGATACAACGACACCAAAACGGAGAAGGGATCCCTCAGACACACTAGAGAAGACTATGGAGCGGAGGCTAAAGAGAAAAATCAAGAACAGGGAGTCTGCTGCTCGCTCTCGAGCAAGAAAACAGGCCTACCATAATGAATTGGTGAACAAGGTCTCACGCCTTGAAGAGGAGAATTTAAAGCTCAAGAAAGAGAAGTGA

>ClabZIP17

ATGATCGATAGAGTATTCTCCGTCGACGGAATCTCCGATCACTTCTGGACGTCGCCGGAGGAATCGTCGAAGTTGAACCGGAGCGCATCCGAATGGTCCTTTCGGAGATTTCTTCAAGAAGCTGCCTCCGTATCGGATTCCTCCATTTCTCCGCCTCCGGCTTCGCCTTCCGCGGCGGAAATTCGATCTAACGCCGTCGAGATTCGGGAGAAACAGAGTAATCAGAACGTTGGAGTTATGAAGGAAAGGGAAATATGCAGTAGTAGTGCGAGGGAGAAAAGTAGGGCGGCGGCGGCGGCGGATTCCGATGAGTATCAGGCGTTTCTGAAAAGCAAGCTGAATCTGGCGTGTGCGGCTGTGGCCTTGTGTCGAGGATCTTTTATGAAGACTCAAGATTCTTGTGCAAGTTCCACTCAAGCCGGTGCCTCACACTTAACATCTCAATCCTCTTCAAAAGGAATTTCTTGCTCACCTTGTGTGCAAAAAAGGGCTGGAATTCTGGTCAGCTCAGCAAATATATCATCATCAAGAGAACAGACTGATGAAGATGATGATGTTGAAGGTGAAAACAATATGAATGAGCAAATAGATCCTGCATCCGCTAAACGTGTTAGAAGAATGCTTTCAAATAGAGAATCAGCTAGACGCTCAAGAAAGAGAAAGCAAGCACATTTGACAGAGCTTGAAACACAGGTTGCTGAATTAAGAGTTGAAAATTCAGCACTACTGAAGCGTTTCGGTGATATAAGCCAAAAGTACAATGAAGCAGCCGTTAATAACAGAGTTCTGAAAGCTGACCTTGAAACTTTAAGAGCAAAGGTACAGATGGCTGAGGAAACTGTAAAGCGAATCACTGGTATGAAATCTATGGTCCATGCCATGTCGGAAGTATCCTCAATTAGCATCCATTCCTTTGAGGGAAGCCCTTCAGAGATATCAACAGATGCACCTAACAATCATATTGCCGACATTTCTTCTGCAAATATTCAGAAGAATTCTCTGGAAATGGCAACCGTGTCAAGGAACAAGATGGCAAGAACAGCTTCCATGCGGCGAGTAGCAAGCTTGGAGCATCTTCAGAAGCGCATACGGGGGAGTTCCAGCTCCTGTCATCCATCAGGAAAGGGAGATCAGCAGTAA

>ClabZIP61

ATGGAGATGAATGAAGATTTGGGATTTGGTGAAAATCCATTTAATGGAAGCTTGAAAAGGCATTGTTCTTCACATTTGATCATGGAAACAAATAGGATGATGAGAAGAGAAGGTGATGATCATGAAGATGAATCAAATGGTTGTTGTGGTTTCCATAGAGAAATATTGTTCCCTACTATGATCACTACTACTGGCCAAGCCCCAACCACCACCAACAACAACAATTGTAATGTCTTCTCTCCCAATTCCGCTTCTTGCTATAGCAACGACAACATTTTGGACGTCGTTGAAGTTCTCGACATCCATCGTCATCACCATCTGACTGTAATGGCCGAGAGGAAGCTAAGAAGAATGATATCAAATCGAGAATCCGCAAGGAGGTCACGAATGAGAAAGAAGAAGCAGATCGAAGAGTTACAATACCAGGCAAGATTTTCAAATATATATCTACCTTAA

>ClabZIP18

ATGGGAACTAGTGAAGAGGCGAAGTCTGTGAAGACTGAAAAACCATCCTCACCAACACCGGATCAGAATAGTGTGCCAAATTCAGCCAGTATTCATGTTTTTCCTGATTGGGCAGCCATGCAGGCATATTATGGTCCTAGAGTTGCAGTTCCACCATACTACAACTCTGCTGTAGCTTCAGGTCATGCTCCTCACCCTTATATGTGGGCTCCACCACAGATGATTCCTCCATATGGTACTCCTTATGCTGCAATCTATTCTCATGGAGGTGTTTATGCCCATCCTGCAGTTTCTATGGGACCACATTCACATGGCCCTGGTGTTCCCTCATCACCTGGGGCTGCACCTCCTTTGAGTATTGAAACACCTTCAAAGGTATCTGGAAATAGCAGTCAAGGTTTAATGAAGAAACTGAAAGGTTTCGATGGCCTGGCAATGTCCATAGGCAATGTTAGCACTGAGACTGCTGAAGGGGGAGCTGAGCATGGACAATCAGAGAGTATGGAAACTGAAGGTTCCAGTGATGGAAGTGATGGAACTACTGCTGGGGCAAATCAAACCAAAAGGAAACGAAGCCGGGAGGGAACACCTACCACTGGTAAGGATGCAAAAATTGAGCCACAGGCAAGTCCAGTTACTGCTGCTGAAATGAATGAAAGCTCTAGCAAATTATTGGGCACGACCAAAGCAGCTAATGCGACTGGAAAACTTGGATCTGTGATTTCTCCTGGAATGTCTACTGCATTGGAACTGAGAAATCCTTCCAGTATAAATGCCATGACAAGTCCAACGACGGTTCCACCTTGTTCAGTACTGCCTTCTGAAGTCTGGCTCCAGAATGAAAAGGAGCTAAAACGGGAAAGGAGAAAACAGTCGAATAGAGAATCCGCTAGGAGGTCAAGGTTGAGGAAGCAGGCTGAGACAGAGGAACTAGCTCGTAAAGTTGATTCGCTGACTGCCGAGAATGTTGCTATTCGATCTGAAATAAGTAGATTGTCAGAGAACTCCGAGAAACTTAAGAAAGAAAACTCTACCTTGATGGAAAAGCTTAAAAGCGCTCAATCAGGACGAACAGAAGCATTAGACATGAATGAAAAGAGGATGCAACAACCTGTAAGCACAGAAACCAAAGGACCTGTGAATAAAAGCATTAACGAAGAGAGTATCATCTGCAAGAAAAACTCGAGTTCGGGTGCAAAACTGCGGCAGCTCTTGGATACGAGTCCGAGGGCGGATGCAGTCGCTGCTAGCTAA

>ClabZIP19

ATGGAGAAGGACAAACCCCAAGGCTATTCTGGTGGATTTCCAACACCATCAAGTCGTTACTCAGTTCTTTCACCAACTGGAAGCATTTTCAATGGAAAATCTGAGGCAACTTCATCTTCAATGTTGCTTCCTCCGATGCCTTCAGGTGCTCTTTCTGATTCAGGTCAATTTGGTCGTGAAAGGCCCACTGATTCTAATCGGTTCAGCCATGATATTAGTCAAATGCCTGATAACCCACCGAGAAATATTGGTCACAGACGTGCCCATTCAGAAATCCTGACCCTTCCTGATGATATTTGTTTTGACAATGACCTTGGTGTCATTGGTAGTGCTGATGGCCCTTCTTTTTCTGATGATACTGAGGAAGATTTGTTGTCCATGTACCTTGACATGGACAAATTCAATTCTTCGACTGCCACCTCCGCAATACAAGTTGGCGAGTCATCTTCTGCTGTTGGAGATGCAATATCAACTCCTACTCCAGCAATTGGAGCAGCAACCTCTAAAGATGATACTGCTGTTGGTTTGAAGGAGAGGCCGAGAGTTAGACACCAGCATAGCCAGTCCATGGATGGCTTGACAACCATAAAGCCTGAGATGCTTGTCTCAGGGTCTGAAGAAGCCTCTGCAGCCGATTCCAAGAAAGCCATGTCAGCTACAAAGCTCGCTGAGCTTGCGCTTATTGACCCCAAACGTGCTAAGAGGATATGGGCAAATAGACAGTCGGCTGCTAGGTCAAAGGAAAGAAAGATGCGGTACATTGCTGAGCTTGAACGGAAAGTTCAGACTCTGCAAACAGAGGCAACTTCTTTATCTGCTCAGTTAACCCTCTTACAGAGAGACACAAACGGTCTTTCTGCTGAGAACAATGAATTAAAGCTGCGGTTGCAGACAATGGAGCAGCAGGTTCACCTGCAAGATGGTCCTTTTTGGAAGATGATTTTATTTGCATTTTTGGCATTCATAGACATTGTCACATTGAAAGTTGCTTTAGTCTCCAACTAA

>ClabZIP20

ATGGACAAGGAAAAGCCCCAATGCCATGGGGGAGGATTTCTTCACCAATCAAGTCGTTACTCGGGGTTTTCATCTGCTGAAACTAGTTTCAATGGAAAATCTGAAGCAACCTCGTCTTCAATGTCATTCCCTCCATTGGCTCCCAGTACTAATTCTGATTGGGCTCAATCTGGCCGTGGAATGTCCACCGATTCCGCTCGGTTCAGCCATGATATTAGTCGGATGCCTGAAAATCCACGGCGAAATGTTGGTCATCGGCGTGCCCATTCAGAGATCTTGACCCTCCCAGATGATATTTGTTTCGATAGTGATCTTGGTATTATTGGTGGGGCTGATGGTCCTTCTCTTTCTGATGATACTGAGGAAGATTTGTTGTCCATGTACCTTGACATGGATAAATTCAATTCCTCGACTGCTACTTCTGCAACTCAAGTGGGCGATTCATCTTCCCCTCTTGTAGAAGCAGCAGCAACTTCTACAGATGATATTGCTGTTGGTTTGAAGGAGAGGCCGAGAGTTAGACATCAGCATAGCCAGTCCATGGACGGCTTGACAAACATCAAACCTGAGATGCTTGTCTCTGGGTCTGACGAAGCCTCTGCAGCTGATACCAAGAAAGCCATGTCAGCTGCAAAGCTTGCTGAGCTTGCTTTGATTGACCCCAAACGAGCAAAAAGGATATGGGCAAACAGACAGTCAGCTGCGAGGTCAAAGGAAAGAAAGATGCGATACATTGCTGAGCTTGAACGGAAAGTTCAAACATTGCAAACAGAGGCTACTTCTTTGTCCACTCAGTTGACCCTATTACAGAGAGATGCAAATGGTATCACTGCTGAGAACAGTGAACTAAAGCTGCGGTTGCAGACAATGGAACAGCAAGTTCACCTGCAAGACGGAGCTTTCACAGTATCAGGTCCATCAGGACTTGGTACTGGAATGATTCAATTGGACATGCATTAG

>ClabZIP21

ATGGCAAATTCTAAAGGGTCATCCAACGTCAGAAGTTTTATGAGTTCTGGGAAACATGCACTACTCCCTCCTAAAAGTCCCTTTCCTAGTGTTTCTCCATCATATACGGAATATGTTCCTAATAGTGTAATAGGAGCAAAAGCTGTTCAGAGACCAAGAGATGGTAACAGCTACCATCAAAGAACTTCTTCCGAAAGTATTTTAATAGAGGAGCAGCCTTCTTGGCTTGATGATCTCCTCAATGAGCCAGAGACCCCTGTTCGCAGAGTTGGTCATCGACGTTCGTCGAGTGACTCCTTTGCATATACAGATGCTGCTAATGTAAATTTTGATAGTATCATGCAAGAAGAATTTAGATATGCAAATGCAATTCCTGGACACTCTTGGTTATCTCAAGAATTTGATCATCAGAGAGATGCAAGGCATGTTTCATTCTATACTGAAGCGAATATAGCAAAACAGAAGAATAGGGTGTGGGAGTCGTCTTTATCTACCATGAATAATCCCATTGCCCTTCATTCTCCTAGGGAAAACATTGGTATTCATACCTCAGGGCCACTAAACACGCCACAGGAAGCAGATGGTTTGCCTTCTACGGCAAGTGAGAAACAGGATTCAACTGAGTCTGGTTCACACGATCCAAAAGTCTCTTCTGACAGGAAAGATGCTTCTCATGGAAAATCATCTGTGTCTGATACAGAAAATAAACGCGCAAAACAGCAATTTGCTCAGCGTTCAAGGGTTCGGAAACTTCAATATATAGCAGAGCTTGAAAGGAAAGTACAAGCTTTGCAGGCAGAGGGCTCTGAAGTCTCAGCTGAGCTTGAATTTCTCAACCAACAAAATCTTATTCTTAGCATGGAAAACAAAGCCCTCAAGCAGCGGTTAGAGAATTTAGCTCAAGAGCAGCTAATTAAATACTGTAAGCTCTTATTCCCTAGATATCTCGCATTCTTTGTATGTTGA

>ClabZIP22

ATGCAGGAACGAGCCGCCGCCGCAGCCGCCGGTCGTCTGCGCTCGAGTAGCGAAAGATCGTCGAGCTCCGCTTTTCAACTTGATGTTAAAGAAGGAGCGGAGAGTGATGAGGAGGAGATAAGCAGAGAACCGCAGATCTGTGGCAACTCCGTCTCTGCCGTTGGCATCTCAGCATCTGGTAAAGCGCCTGCATCAGATAGCATAAGGAGCAGAGGACGAAGCGCCGCTGAGAAAGAAAGCAAAAGGCTGAAGAGATTGTTAAGAAACAGAGTTTCGGCACAGCAAGCAAGGGAAAGGAAAAAGGCGTATTTAAGCGAATTGGAAAGCAGAGCAACAAACTTGGAGAAAAGGAACTCGGAGCTTGAAGAGAAGTTGTCCACGTTACAGAATGAGAACCAGATGCTTAGACACTCGAGACCCACTCCTTTAGCTGTTGTTCGTTCCGAGTTCCAGCCATTGTCGTCTGCGGGTGGTCGTTTCTTCGTTCATCGTCGACTCTCTCTTTCCTGCTGGCAACTATGTCTTATCCTAGTGCTGCCACCGCCTCCACTCGATGATTTGCTGCCATGGGTTTGGTTCCTCCCTTGTCACCCGCCGCTGTTTACTGTCGCCAGAAAGGGTAATCTTTACTAG

>ClabZIP23

ATGATTATGGACTGTTCTAGTGGGAATTCTTCAGGTTCATTATCTCAGATTGTGCTTCAGAACCAAAGCTCTGGTTCTGAAGAAGAGCTGAGGCAATTGATGGACCAAAGAAAAAGAAAACGAATGCAATCGAATAGGGAATCGGCGAGGAGATCGAGGATGAGAAAACAGCAGCATTTGGATGGGCTAATGGCGCAAGTGAGTCAACTCAGAGATAACAAAAACCAAATGATTTCAAGAATTAATCTCACAACTCAACTCTTTCTCAACATCGAAGCAGAGAACTCTGTTTTGAGAGCTCAGATTCTGGAACTTACTCATAGATTAGAGTCGCTAAACCAGATCTTATCTCACATCAACGACAACGATGATGAAGAACAACATAATTTTCTTCAAAACTTTGATGATTTTGATCATAACCCATTATTCATCAACTCCTTCTTCATCACCCAACAACCCATTGTGGCTTCTGCTCATCATCACCTGCTTCACTACTGA

>ClabZIP24

ATGCTTCCAGGTGAAATGACTGGCATCCAGTTTTTTCCATCCGAAAACTCTTTTCAAATTCCTTCAAACATTGGCATGATGCAGAACAGCTTTCAGACTCTCCATCATTTCAATAGCTTTTTAGGCAACCTTCCGATGTCTCACGTCCCTCATCCCAGCCATGAATTCCTTGCACAATCCTCAAGTTTTAGCTACAACTCAACATCTGATGATGCAGAGGAACAACAGAAGAGTATTATTGATGAGAGAAAGCAGAGGAGAATGATTTCTAATAGAGAATCAGCTCGTAGATCACGGATGAGAAAACAGAAGCACCTTGATGAGCTTTGGTCTCAGGTACTTCGGCTTCGCACTGAGAACCATAAACTGATAGACAAATTGAACCATGTATCCGATAGCCACGAAAAGGTTCTTTTGGAGAATGCCAGGCTCAAGGAAGAAGCCTCTGATCTTCGTCAGATGCTCAGTGACCTCCAAATTGGCAGCCCCTACACTCCTTGCTTGAGTAACCTCGAAGATATTCCCTGCAACAGTGCACATCTCAGAGCCGAATCTTCATCATGCCAGTCCATCGCCAACTCCATAGATAACCTACTCCATTGA

>ClabZIP25

ATGTTCTATTCCGAAGAAGAAGAAGTCGTCGGGTTTCCTGGGCCGGCTCAGGAACCTAGGTTCACTCCGGCGAAAATCCAGGAGCTTTGGTCTCTACTCGAAGATCCTACCAGGTCGAGCTCCGGTTCCCAAGGGTCGTGTCAAGCCGTATCTTTGATCGACGAGGAGAGGAGGCGGAAGCGAATGATATCGAACCGAGAGTCAGCGAGGCGGTCGAGGTTGCGGAAGAAGAGGCATTTGGAGAACTTGGCGGTTCAAACGGACCGGCTGAAGATGAAGAACCAGGAGCTGAAAAGGCAGCTAAATTTAGTAGTGAACCGTTGTTATATGGTAAGAAGACAGAATGAAAGATTATGGTCGGAATTTGTGGCGCTTCATGCACGGCTGTCGGACCTTTACCGGATTTCTGTTCCCATGCAAGAGAAGGAGAATTCATGCATGCAAATATCATTCAATTATTTCTCTTAG

>ClabZIP62

ATGGTAACCGAGGGCGAGTGGACCAGGCAAGTTGAGAAAGAAATGAGAAGAAAGTGGCAAACGGGATGCCAGGTTTCGCTTCAGAGATGTCCATTTGTAACCAACTTGATGCCCTTGGTTTCCTTACCTTGCAGTATTACAGAGAACCTCAAAGTTTATGCCATGACTGATGTCAGTCCAAGGACTGATATTTCCACTGATGTCGACACCGATGAAAAAAACCAGAGGCTCGATATGCTTCAACGGAATGTTGTGGCTTCTGATTCCAGTGATAGAACAAAAGATAAGTCTGATCAGAAGACTCTACGTAGGCTTGCACAGAATCGTGAAGCTGCCAGAAAAAGCCGACTGCGAAAAAAAGCATATGTCCAACAGCTGGAGAGTAGTCGTTTAAAGTTGACCCAACTAGAGCAGGAGCTTCAGCGAGCACGGCAGCAGGGAATCTTCATATCAAGCTCAGGAGATCAGGCCCATTCGATGGCTGGAAATGGGGCCATAGCATTTGATGTGGAATATGCCCGTTGGTTGGAAGAACAAAACAAGCAGATCAATGAACTGAGAGCAGCAGTAAACTCTCATGCAAGTGATACTGAACTTCGAATGATTGTCGATGGCATATTGGCTCATTATGATGAGGTTTTTCGGCTAAAGGGGGTCGCTGCAAAGGCTGATGTTTTCCATTTATTGTCTGGCATGTGGAAAACACCTGCTGAAAGATGTTTTTTGTGGCTTGGTGGTTTCCGTTCATCAGAGCTCCTAAAGCTTCTTGTCAATCAATTAGAGCCCCTCACAGAACAGCAGTTGATGGGCATATCCAACTTGCAGCAGTCTTCCCAACAGACTGAAGATGCATTGTCACAAGGCATGGAAGCGTTGCAACAATCGTTGGCTGAGACGTTATCGAGTGGATCTCTTGGCTCCTCGAATATGTCTGGCAATGTGGCAAATTATATGGGTCAAATGGCCACAGCCATGGGTAAACTTGGGACCCTTGAGGGGTTTATTCGCCAGGCTGACAATCTACGGCAACAAACACTGCAACAAATGCATCGGATTTTGACAATTCGTCAGTCAGCTCGTGCACTTCTTGCAATTCATGATTACTTCTCCAGACTACGAGCCCTTAGTTCCCTTTGGCTTGCTCGCCCAAAAGAGTGA

>ClabZIP26

ATGGAACTCCCCAATTCCACTAATCAAATGGCTTCCTCTTCCAACCCCACCACCCCATTTCGCGGTTCCTTCCACCGTCGAGCCCATTCCGAGGTCCATTTCCGGATTCCCGATGATCTCGATCTTGTCTCCGACCCTTTCGACGCCCCTTCTTCTGGATTTGGAGATCTGGGCTTCGAGGATGATCTCTTGTGCACTTTCATGGACATCGAGAAGATCGGATCCAAAATCGACAATGGGTCTTCGTCGAATCCTGAAGTGGCTGGCGGCGGTACTGCGGCGGAGAATGTTGAAGGGGAGAAAATCTCCCGCCCCAGGCACCGTCATAGCAATTCTGCTGATGGGTCTTCTATTATGGAGTCTATTGAGGCTAAGAAGGCCATGGATCCTGATAAGCTCGCTGAGTTGTGGACAATTGATCCTAAACGTGCCAAAAGGATTTTGGCAAATAGACAGTCTGCTGCTCGGTCAAAAGAGAGAAAAGCTCGCTACATAATGGAACTTGAGAGAAAAGTTCAAAGCCTTCAGACCGAAGCGACAACACTCTCTGCACAGCTCACGTTGTACCAGAGAGATACCACGGGGCTTTCAACAGAAAACTCAGAGCTCAAACTTCGTTTACAAGCTATGGAACAGCAAGCTCATCTTCGCGATGCTCTAAATGAAGCTTTGAAAAAGGAAGTCGAGCGGCTCAAGATCGCAACTGGAGAAGTAATGACAGCTACAGATTCGTATAACTTTGGAATGTCGCAAGTTTCATATCCCCAATCTTGCTTCTCACACCAACCACAACCTGAGCGACACAACCCACAGAGGACGACGCAAGGGCCCCAAGTTCATCCATTCCATTCTAGTTTACCAAACCCACATCAGTCTTTGTTTGTTGCATCGCACCAGCCTCACGCCTTGACAGAAATGTTTCATCAAGATCCTATTAGCCGATTGCAGGGTCTCGACATCAGTAGCAGAGGCACAGAAATAAAGCCAGAAGGCTCCTCTATATCCGTCAGTGAAAGTAGCAGCACATTTTGA

>ClabZIP27

ATGGCGTCTCCTGTAGGAAGTTCATCTGGATCTCCGAGCTCCGACGAAGATCTGCGGCAAATCGTGGATCAGAGGAAGAGGAAGAGAATGATATCGAATCGAGAATCGGCTCGCCGATCTAGGATGCGAAAACAGAAGCAACTAGATGATCTGACGTCTCAGGTGGGCCAAATCAGAACAGAGAATGAACAAATCGCCGTCAATATCAATTTCACCAACCAACTTTATGTGAATCTAGAGGCGGAGAACTCTGTTCTCCGAGCTCAGATGGTGGAGCTCCGCCACAGATTGGACTCGCTTAACGAAATCATAAGCTTCATGAACTCAAGTACTAGAAATCTGTTTGATTCTGAGGACCATTACGAAGCTCCTGGCATTGATGGGTTTGTTGATTCTTGGGGATTCCCATTTCTCAACCAGCCAATCATGGCGGCTGGTGATTTGTTTATGTGTTGA

>ClabZIP28

ATGAACTCATCATCCACAAAATTTTACAATCCAGGAAGGATGGGATTGTATGAACCTCTCCACCATATTGGAATGTGGGGAGAAACCTTTAGAACCAGTGCCAATTTAGATGCACAATCCTCCTTTATTATTGAAGCTGATACCAAGCTTGAAACTCAGTCGGATGATGCTTCACTCGGCTCACTTGGAGATCCTCATGTATATGACCAAGATGATACCAAACGCATTGATAAGATTCAAAGACGGCTGGCACAAAATCGGGAAGCAGCTCGCAAAAGCCGGCTGCGGAAAAAGGCCTATATTAAGCAATTGGAAACAAGCCGTGTAAAACTCATTCAATTGGAGCAAGAGCTTGAAAAGGCAAGACAACAAGGTCTCTTGGCTGGATCCCGATTTGACAATAACCAGTTGGGTTTATCAGGAACCACAAATTCAGGCATCTCCGCATTTGAATCAGAGTATGAGCAATGGGTGGAGGAGCAGAACAGGCAGATTTGTGATCTGAGGACTGCTGTGCATGCTGATATTACTGATATCGAGCTTCGAATACTTGTCGAAAATGCAATGAGACACTACTTTAAATTTTTTCGCATGAAAGCTAAAGCTGCAAAAGCCGATGTCTCTTACATAATGTCAGGCATGTGGAAAACATCGGCCGAAAGACTTTTCTTATGGATTGGAGGATTTCGACCTTCAGAACTCCTCAAGGTTTTGATACCTCAGCTGGAGACATTAACCGAACAACAAATTTCGGAAACTGGTAGCCTTAGGAAATCTTGTCTACAAGCAGAAGATGCTCTTAGACAAGGTATGGAAAAACTACAACAAAATCTATTTGAGAGCGTAGTGGCTGGTCAGCTGGGTGAAGGAAGTCATCCTCTACAAATGACTGCTGCAATGGAGAGATTAGAAGCGCTCATTAGCTTTGTGAATCAGGCTGACCATCTACGACAGGAAACATTGCAACAGATGTACAAAATTCTATCGACTCGACAATCTGCTCAAGGCCTTCTTATTCTTGGAGAGTTTTTCCAACGACTCCGAGCATTGAGCTCACTTTGGGCTAATCGTCCTTGTGAGCCTGCATAG

>ClabZIP29

ATGAATCCACCATCCTTAAGCAGTAACTCAACTTCTGATGAAGCAGAAGACCAACAACTAAGCCTCATCAATGAGAGGAAACAGAGGAGGATGATATCTAATAGAGAGTCTGCACGTCGGTCGCGAATGCGCAAGCAGAAACATTTGGATGAGCTGTGGTCTCAAGTCCTTTGGCTCCGGAATGAAAACCACCAGCTAATAGATAAGTTGAACCAAGTCTCTGAGTGTCATGATCGGGCTCTACAGGAGAATGCTCAGCTTAAGGAGGAGGCATCTGAACTTCGTCAAATGCTTACAGACTTCCAACTCCATAATCCTTACCTACCTTGA

>ClabZIP30

ATGGATCTTCCAAGTGAAATCCCAGAAGAAACCCAGTTATTATTAGGAGCTGAAGAAAAGTACTTTTGGACGATTGAAAGGAATGATCTTCAAAACATGATCTCTAGTGATCAGTTTTGTCGTCCAACAAGTAATTACAACAATAATAACAACAGGAGAAGCGCGGAAATTATGGTGGATGAAAGGAAGCAGAGGCGGATGATATCGAACCGAGAATCGGCTCGAAGGTCCCGAATGAGGAAGCAGAAGCATGTAAAGGAGCTTTGGTCTCAGCTGGCTCAGCTATGCACTCAGAACCATGACCTTGAGGAGAAGTTGAGGCATTTGATGGAGTCCCAGCAGCGCCTTCTCCAAGAGAATGCAAGCCTTAAGCAACAAGCTTCTGCTTTCCGCCAAATCCTTAGAGATATGGAACTTGAACAACTCGTCACCCAATTTTGA

>ClabZIP31

ATGCAGAGCTTTGAAAACCCAGAACAGTTCTATGCCCATTCTTCTTCTTCTTCTTCAATTTTTCTTCGGGGAGACGACAGTGGGCGATTCCATACGCGTTTTCTTCCGGATATTGAGGAACTTCAACAATCAGCTGCTGCTATTGCTGCTTTTCATCAAGATGATGCTGTTGATTTAAGCCCAAGCTCTGTTTTCAGTTTAAAATCGACCCACAATACAGCTTTTCCAATCTACCTACCATACGGGAATACGGATGTGGGTTCAATAGGAAGAACAGGGTATTTGGACACAGGACAGGAATTGATGCGGTTGAAGAGGCTGGCTCCGCCGCAGTCGGTGGCGGTGACGGTGGCAGCTTCGTCATCGTTGGGAAACGGGAGCTTTGAGAACTGGGGAGAGTCAGCCATGGCAGATAATAGCCAGCAGACTGACACTTCTACAGACATCGACACTGATGAACGAAACCAGTTTCAAGGAGCTGCACATGGAGCTCTCATGGCGGTGGACTCCATGGATCAATCAAAACCAAAATCTGCAGATCAGAAGACTCTTCGTAGACTTGCTCAGAATCGAGAAGCAGCCAGGAAGAGTCGACTGAGGAAGAAAGCATATGTCCAGCAGCTGGAAAATAGTCGACAGAGGCTTGCACAGCTAGAGCAGGAGCTTCATCGAGCACGCCAACAGGGCATTTTTGTGGCTTCCGGGGCAGGAGATCATTGTGCATCAATGGCAGGAAATGGTGCATTGGCATTTGATCTCGACTATGCACGTTGGCTTGACGAGCATCAACGGCTGATCAATGATTTGAGAGCATCTGCAAATTCTCAGTTGGGGGACGACGAATTGAGATTTCTTGTTGATGGGGTGATGGCACATTATGACGAACTCTTCAAGCTGAAAAGTGTAGGTGCTAAGGCCGATGTATTCCACATTCTTTCGGGCATGTGGAAGACTCCTGCTGAGAGGTGTTTCATGTGGCTGGGAGGATTCCGTTCATCCGAGCTTCTAAAGATAGTAGGGAGCCATTTGGAGCCTCTAACAGATCAGCAGTTGATGGGTATCTGCAATTTGCAGCAATCTTCCCAACAAGCTGAAGATGCATTGTCGCAAGGAATTGAAGCTTTGCAACAATCTCTTGTAGAAACTCTTTCCACTGCTTCTTTAGGTCCAGCCAGTTCAGGAAATGTTGCTGATTACATGGGCCAAATGGCAATCGCGATGAGCAAGCTAACAACGCTCGAGAACTTTCTACATCAGGCGGATCTTCTGAGGCAGCAAACACTCCAACAGATGCATCGGATATTAACTACTCGTCAAGCTGCACGAGCGCTTCTTGTGATCAGCGATTACATTTCACGTCTTCGAGCTCTCAGTTCTCTGTGGTTAGCACGGCCGAGAGACTGA

>ClabZIP32

ATGATGGATTTGAACTTTACCTCCCGGAAGCCACCGCAGACGGCGCCGAGGATGGACATCGAACAGATGCCGGAGGCTCCACACCGTGGGTATCACCACCGCCGTTCCCACTCCGATACCTCCTTCCGCTTCGCTAATTTTGACGATCTCCTCCTCTTCGACCCTCCCGACATCGATTTATCATCTGCTCTTCCTTCTCCCTCGCCGTCACCGTCTCCGACGCCGTCAGGGGCTCGTATGGCCGTCGATTCCTTCAACTCGAGGTCCCCTGAAGACGTCTCTGACACAAAACCTAGGGCTGGAAACGGTAACTCCGCTTCCTTCTTCAGTTCTCACTACCGGAGCTTGTCGATGGACTCCGACTTCTTTGAGGGATTGGGGATGGCCGGAGATGGGAGTGACGGTGAAGTACTTGGCGGGAGAGTGACGGCGGGGGAGAAGAAGATGAGTCGCCATAGGCATAGCAATTCCATGGATGGGTCTTTGACCTCCTCGTTTGAAGCTAATTCCACCAAGAAAGCCATGGCACCTGATAAACTCGCTGAGCTTGCTTTGATGGACCCTAAAAGAGCTAAGAGGATTCTGGCAAATAGACAATCAGCTGCACGATCCAAGGAGAGGAAAATACGTTACACTAATGAACTAGAGAAGAAGGTGCAGATGCTTCAGTCAGAAGCCACTTCCCTTTCTGCTCAGGTCACAGTGTTACAGAGAGATACCACTGGATTGACTACTGAGAACAGAGAACTCAAGCTAAGGTTGCAAGCCATGGAGCAGCAAGCCCATCTGAGGGATGCTTTGAATGAGACACTGAAGGAGGAAGTGCAGCGGCTTAAGATAGTTGCGGCCCAACTGCCTGTTGCAAATGGCAACTCATTCAACATTAGAGGAGTGCTACCTCCTCAATTCCCTCCTCTCCAAACATCATCATTGCTTCAATTTGGCAACTCCCAGAATCATCATCAACAGCCACAACTGCTTCACATGTCTCAGCCTGATGCTCGGGGAGGGTCTCCCCCGTCCCAGCCTCCCGGAGTTTAA

>ClabZIP33

ATGACCGTCGACGGCTTACTTCGTAATGGCTACAATTCCAATCCCACGGAATCGTCTATTCTCTTAGATGCACAGATTACTCTTGTTGATTCCCCTAACCCCTCTTCGCTTCCCATGAATACCACCACCACTACCACCACTAATTCCTCTGCTGTCATTGATAGTAACCATAATTCTTCCTCTGGTGCGCCGCCGCCCAAAACTGTGGATGATGTGTGGAGGGAGATTGTTTCTGGTGAGAGGAAGGAATTGAAGGAGGAGGTTGCTGACGAGATGATAACCCTCGAGGATTTTCTTTTGAAATCTGGAGCTGTGCCTGTTGAGGATGTTAAATTGCCGCAGACAGAGAGGCTGAGTGGAGGGGTTTTTTCGTTTGATCCAATTCCTTCCACTACATTTCAGGCATTGGATAAGGTTGAAGGATCCATTATTGGGTTTGCTAATGGGGTTGATTTGATTGGAAGTGGTGGAAGTGGGGGCAGAGGTAAAAGAGGGCGAGCTGCTTTGGAACCTTTAGATAAGGCTGCAGAGCAAAGACAGAGGAGGATGATCAAGAACAGGGAGTCTGCAGCAAGGTCTAGGGAACGGAAGCAGGCTTATCAAGTGGAATTAGAGTCGTTAGCTGTAAGATTAGAGGAAGAGAATGAGCGGCTTTTGAGGGAAAAGGTATTCCTTTTTTCTTTCGATATTCATATGAGAACATCTTTCTGA

>ClabZIP34

ATGGGGTCTCAGGCCGGAGGTGATCCAAATGGCAAACAATCACAATTCCAGCCATTGGTTCGTCAAAATTCACTGTATAGTCTCACACTGGATGAGGTTCAAAATCAGTTGGGTGATCTAGGAAAGCCTTTAATCAGCATGAACCTTGATGAGCTTCTTAAAAATGTGTGGACTGCTGAGGCCAACCAAACTGTTGGTAAAGACAATGAGGACAATAATGTCTTGGCCAATCAAACATCCCTACAACGTCAGGCTAGTCTTTCATTGAATGGTGCTCTGAGTAAAAAGACTGTCGATGAGGTGTGGAGAGACATTCAACAAAGCAAAGACAGTGAGGAGAAGAAGTCTCGTGAACGGCAACCTACGTTGGGAGAGATGACATTGGAAGATTTTCTGGTGAAAGCAGGGGTGGTTGCTGAGACATCGTCGAATAAAAAAGGTGCGGGTCCTGTTGTTGAAATTGACGCAAATATCACACCCCAGTTTCAACAAACGCAGTGGATGCCGTACCCTCAACCTCAGTATCAGTCTCAACAAGCAGCAATGATGGGGGTTTACATGTCAGGCCAGCCTATTCCCCAGCCACTGCATGTGGGAGGTGGTGCTGTGATGGATGTTCCATATGTAGACAACCAGTTGGCATTGCCAACTCCTTTGATGGGAGCTTTATCTGATACACAGACATCTGGAAGAAAAAGGGGAGCGCCTGAAGACATGATTGAGAAAACTGTCGAGCGTAGACAGAAGAGGATGATAAAGAATCGGGAATCTGCCGCTCGTTCACGAGCAAGGAAGCAGGCATATACCAATGAACTGGAGAATAAAGTTTCACGTTTGGAAGAGGAGAATGAAAGGCTCAGGAAACGGAAGCTGAACGGATTGACATTTGTCACCTCTCATTAG

>ClabZIP35

ATGGCTACTGAGGGCAGTCCCAGGACTGATATCTCAACAGATGGGGACACAGATGAAAAAAGCCGGAGGCCTGATAGAGGCCAACTTACTCTTACTATGGCTTCTGATTCTAGTGACAGATCCAAGGATAAAACTGATCAGAAGACTCTTCGCCGGCTAGCACAAAATCGTGAGGCTGCAAGGAAAAGCCGATTGAGAAAAAAAGCATACGTTCAACAGCTAGAGAGCAGTCGATTAAAATTGACACAACTTGAGCAGGAGCTTCAGCGAGCACGACAGCAGGGAATTTTCATATCAAGCTCAGGTGATCAGACCCATTCAATGAGTGGAAATGGGGCCATGGCATTTGATGTAGAATATGCTCGATGGCTTGAGGATCACAACCGTCAATTGAATGAATTGAGATCAGCTGTTAATTCTCATGCAAGTGACCCGGAACTGCGTATTGTTGTCGATGGCATCTTGGTTCATTATGATGAGCTCTTTAGGCTCAAGGGAAATGCAGCAAAGGCTGACGTTTTCCATTTATTATCAGGCATGTGGAAAACACCTGCAGAAAGATGTTTTCTTTGGCTCGGGGGTTTTCGTTCATCCGAGCTCCTAAAGCTTCTTGTAAATCAACTGGAGCCTCTAACAGAGCAGCAGTTGGTGGGAATAACAAACTTGCAGCAATCATCGCAGCAGGCAGAAGATGCATTATCACAAGGGATGGAGGCCTTGCAACAGTCCCTGGCAGAGACATTGTCGAGTGGAGCTCTCGGCTCCTCTGGTTCGTCAGGGAACGTTGCAAACTACATGGGTCAAATGGCCATGGCCATGGGTAAACTTGGGACCCTCGAGGGCTTTATACGCCAAGCTGACAACTTGCGACAACAGACACTTCAACAAATGCACCGTATATTGACAACTCGTCAGTCTGCTCGTGCTCTTCTTGCCATACATGACTACTTCTCCAGGCTACGAGCTCTTAGTTCGCTCTGGCTTGCTCGTCCGAGAGAGTAA

>ClabZIP36

ATGAAGCGTAGCGCCTCCGAGTTGGCTCTTGAAGAGTTCCTCAAAAAAGCCGCCATCATCTCTCCCGACGACGACGTTATCGACCCCGACGACGAGGACGTTTTCAAAATCGAACAACAACAAATCATCAGAAGCCCAAAAAGAGGCAGAAACTTTCAGGATTCCGCCGATGCCACCTGCTTCTTCGGCGATATTGATTTCAGCTGTTTCCTCGTCAAAAATAATCGGGAGATAATGGACGCAATTGTTAACTGTGGAGGTGGGCTGGCGGAGGCTCCTTTATGGTCACAGAATCTCACTCCTAAGCATTCAAGCTTTTCAGCCACTATAGATTCCCAGTCTTCTATTGTGAGCAGCCCAACATCAGCCAGTAATTTGATGGGCAGAGAGCACCAAAGAGGTAACAATAGTGGGTCCTCTGAAGATCAATCTGATGATGAAATTGAAGCTGGCTCTTGTGAACAAAGCACTGACCCTCTTGCTTTGAAGAGAATCAGAAGAATGATCTCTAACAGGGACTCTGCTAGAAGGTCAAGAAGAAGAAAACAAGCTCATTTGGCTGAACTCGAGAACCAGGTGAAACAATTGAAAGGAGAAAATGAAACCTTGTTCAACCAACTTTTAGATGCTAGTCAACAATATCGTGATGCCAATACGAACAACCGTGTGCTTAAATCAGACGTAGACGCATTGAGAGCTAAGGTGAAGTTGGCGGAAGATACACTTGCTAGAGGCTCAATGACCTGTAGCTTGAATCAGCTGCTGCAGAGTCATTTGAGCACACCACAGCCACTCACGGCACTCAGACGAATGTCAAATGTCGCACCGCCGCTCGGTTTACCTGGAGACGAAGTTTCTTATTCCTGCGTGACAATGTCGGGGCAGAACCCGACCGTGGGGCTCCCGAACTCAGATATGCATATGAAAACAGGAATGGGTAGTGAGGCTGTTAGCTGTGTTTCAGGGATTTGGCCTAGAAATTAG

>ClabZIP37

ATGCAGGAGCAAGCCACGAGTTCAGCCGCTGCTAGTTCTCTGCCTTCCAGCAGTGAAAGATCCTCCAGCTCTGCTCTTCATCTCGAAGTTAAAGAAGGAATGGAGAGCGATGAGGAGATCCGAAGAGTGCCGGAGATAGGCGGTGAATCGGCGGGAACATCCGCTTCCGGGAGGGATACTGGTTCGGTTGCCGGTCCGGACCGGGTTCAAGTTTCTCGGGAGGGTCAAAGGAAAAGAGGGAGAAGTCCGGCTGATAAAGAAAGCAAGAGACTGAAGAGATTGCTGAGGAATAGAGTATCGGCACAGCAAGCGAGGGAGAGAAAAAAGGCATATTTGAATGACTTAGAGATAAGGGTGAAGGATTTGGAGAAGAAGAACTCAGAACTTGAAGAAAGGCTTTCCACTTTACAAAATGAGAATCAGATGCTTAGACAAATTTTGAAGAACACAACGGCAAGTAGGAGAAGTGGTGAGTGA

>ClabZIP38

ATGGATTCTGCGAGCGGAAATTCCTCTGGTTCCACTAGGCTTCAAAACTCAGGCTCCGAAGAGGATTTGCAGGTTCTGATGGATCAGAGGAAACGGAAGAGAATGCAATCGAATCGCGAATCGGCGAGACGATCTCGGATGAGGAAGCAACAGCATCTGGACGAATTGATGGCGCAAGTGACTCAATTAAGGAAGGATAACGCTCAGATTCTTTCAAATATCAACATTACATCGCAGCTTTATATGAACATCGAGGCCGATAACTCGGTTCTGAGAGCTCAAATGGCGGAATTGACTCAGAGATTGCAATCGCTCGAGGAGATCGCTAATTGCATCAACACCGGCAGCAGCAATGGCGGATTTGGAGAAACGGAGGAGGAAGCGTTTCAGATTCAGACAAATGTGGCCGCCGATAGCTTCATGAACTCGATGAATTTCCTGTATGTCAACCAGCCGATCATGGCCGCTGCTGATATCTTCCATTATTAA

>ClabZIP39

ATGAATTTCAAGGATTTTGGGAATGACCCATCTGCCGGAAACGGCGGAGGAGGCGGAAGACCTCCGGCGAACTATCCATTAGCGCGACAATCATCGATCTACTCGCTGACCTTCGATGAGTTTCAGAGCATGGGGAGTATAGGCAAGGATTTTGGGTCGATGAACATGGATGAGCTGTTGAAGAACATTTGGAGCGCTGAAGAAATGCAAACGATGGCATCTTCTGCTGCTGCTGTTGATAAAGAAGGGGCTGGTAGTGTTGGTCGTAGTGGTGGGTATTTGCAGAGACAGGGCTCCTTGACGCTGCCTCGGACACTCAGCCAGAAGAAGGTGGATGAGGTATGGAAGGACATAATCAATGAACATGCTAGTGCTAAAGATGGGGCTACTGTTGCTTCTAATTTGCAACAGAGGCAGCAAACTCTTGGGGAGATGACACTTGAAGAGTTTTTATTCAGAGCTGGAGTGGTGAGAGAGGATACTCAGGTGACTGCAAACCCCAACAATGGAGGGTTCTTTGGCAATAACACTGGTTTTGGAATTGGTTTTCAGCGGCAGGCTAAAGTTCCTGAGAATAATAATCATATTCCAATTCAATCGTCGAACTTATCGCTGAATGTCAATGGAGTCAGAACACATCAGCCGCAGCCAATTTTTCCAAAGCAGCCTGCTGTGACATATGGATCTCAGTTGGCTTTACCCAGTGATGGTCAGCTGGCTAGTCCAGGAATTAGAGGGGGAATTATGGGGATTGGAGATCAAGGGTTGAATACAAATTTGATGCAAGGCTCTGCACTGCAGGGTGGAAGAATGGGAGTGGTTAATATAGCAGCTGCTCCGTTACCTATTGCAACAGAATCGCCAAGGGACCAACTATCATCTGATGGGATTGGTAAAAGCAATGGTGATACCTCGTCTGTGTCTCCTGTGCCTTACGCGCTTAATGGTGGAATCAGGGGGAGGAGAAGTAACGGAATTGTCGACAAGGTTGTCGAGAGGAGGCAACGGAGAATGATTAAAAACAGAGAGTCCGCTGCAAGATCGCGGGCTCGAAAGCAGGCCTACACAATGGAATTGGAAGCAGAAGTTGCAAAGCTAAAAGAGGAGAACCAAGAACTCCGGCAAAAACAGGCAGAAATCATGGAAATGCAGAAGAACCGGGCATTGGAAGTAATGGATAAGCAACAAGGAATAAAGAAGCGATGCTTAAGACGAACGCAGACTGGCCCATGGTGCCGTGTAGCATATGTCTCAAGGCGTCTTGCTTGCAGAGTATCGTATAACGTATTTGCTTCGAAGTGGATCATCAAGAGTTCAGTAGCTTTTGAGTGGAAGCAAAACACATAG

>ClabZIP40

ATGGCAATGCTGAATATGGCGGAAGATGGAGTTGGGTTTCAGTGTGAGGTACTTGAGAATGATCATGAGTTCACTGCCACTGAAATCGAAGAGCTTCTGTCACTCTTTCTAGCCAACGACGGGCCCCCAAGTCCCGGTTCCGATTCTCAGGGTTCGATGCGAACCTCGGCAACTTGTTCGACCAATGACGACGAGAGGAAGCTGCGGCGGATGATATCAAACCGGGAGTCGGCCCGGCGATCGCGGTGGAGGAAGAAGAGGCATTTGGAAGATCTGACTAACGAGGTGAATCGGTTGATGATCCAGAACCGGGAATTGAAGGAACGGCTCGGCCGGGTTTTGAACCACCGCCATGTGGTTTTGAGAGAAAATGATTGGTTGTGGATGGAGTCCGTGGGCCTTCGGGCCAGACTTTCGGATCTTTGCCGGATCTTGGCCGTCATTCAATAG

>ClabZIP41

ATGCAATCGAATCGTGAATCCGCTCGACGATCGCGGATGAAAAAGCAAAAGCAATTTCAAGATCTAACAAGCGAAGTGAGACGTTTACAGATTGTGAACAGTCGGATTGTAGAAAGTGTCAACAGCAGGGAACAAGCGAGGATTGAAATTGAAACAATGAATAATCTTTTGAGAGTCGAAGCAATGGAAACGACGTACCGACTCAAGGCCTTAGATTTGGTGCTTCAAATCGTCGACAAAGCGAATGCTCTTGCAGTTGGTGTTCGTGATCCTCTGTTGGAACCTTGGCAACTCACCAGCAAAGGCAGCTGCCGCCGGCGGCGGCGGATTACGATACGATTCTTTTTTAATGCAGTTTTATTTTTAACATGTAGAATCTTCTAA

>ClabZIP42

ATGCATACTTTCTTCTCATCGGAAGACCTCTCCGATAACTCCTTCTGGCCGCCACAGCCGGCGGCGTCGTCTTCGCCGCCGTCTCATTCTCCTTTTCGGTCGCCGGATCCTTCCTTGACGATGAACCGAAGCGCCTCTGAGTGGGCGTTCGAGAGGTTTCTCGAGGAAGTTTCGGCGTTGCCTGTGAATAGTTGCCCTTCGACTACTTCCGATCGTGTGCCGGTTTCTCCCGTGGATGTGGCATCTCCTGCTTCTCAGTCTTCAACGTCGAAGCGTGATGAAGGTGATGATGAGATTGTGGAGATCAAGAAGGCGGATTGTGATCATGATCGTTCTCAGCCAATTCCGTCTTCGGATCCGTCGAAAATGGTTCGGAGTAGTTCTGATCGGTACCGTGTGTTTTTGAAGAATCAGCTTGATATGGCTTGCGCTGCTGTGGCTCTGTCGCGGGCTGCTTCTTTGGAGCCCAAAGGTCCAGTTCAGCCAGCCGATCACCGTGGGCAAAGTTCGAACGCATTCCAATTTGGTATGCAAGCTCCGGGCCAAGGTTCTGATCGTGGCACTTCAACAAAAGAAAGTGAGGTTTCTGGTAGTCCACTTGGGATTCCATCCTTACCTACAATGCCTAAGAAACTAGGGGTACAACCTGCTCAAACAACGAGTGGATCATCCAGAGATGAGTCTGATGATGATGATCTTGAAGGAGACATTGAAAACATTGAGAATATGGATCCTGCTGATGCAAGACGTGCTAGGAGGATGTTGTCAAATAGAGAATCAGCAAGACGCTCCAGACGACGAAAACAAGCACACTTGAATGAACTCGAGACACAGGTTGGTCAACTTAGAGTTGAACACTCTACTTTGTTAAAGCGTCTCACAGATGTAAATCAAAAGTATGATGATGCTGCTGTTGACAATAGAATTTTGAAAGCTGATATCGAGACTTTAAGAGCGAAGGTGAAAATGGCTGAAGAAACAGTGAAAAGAGTGACAGGAGTGAATCCACTGCTTGTTGCAATGTCTCAGACCCAAATGCCCTTTGTCAATAACCAAATGCCTATGCAATCCAACACTCAATTCTTCCATCAAAATATGTCTGCATTTGCAAACAGCCCCCCACATCACCATAATTTGGAACCTCCTCCCATTCCACATGTTGGAAGATCACAGAATGACGTGGCAACCAAAATATCTGATATGCCCTCTGTGCATATTGATCACGTGCAGAAGCAGGCCATGCATGGCCCTTTGTCAGCTTGGGATGCAGAACCTCCCCATTCAACTCCAAACCACAAGAAGAATTGA

>ClabZIP43

ATGCTGATGGAACCCAATTTCTCGTCGGCGAAGCCACCGCAGCCGGCGGCGATGGACATCGAACAGATGTCGGAGAATCCTCACCGTGGTTCCCACCACCGCCGCTCCCACTCCGACACTTCCTTCCGCTTCCCAAACCTCGACGAACTTCTCTTCTTCGACCCTTCAGAGCTCGACCTCTCGATGCTCTCTTCCCCTTCTTCTCCACCTCCTGGCGGCACTGCCATGGCAGTTGATTCGTCTAATGCTAAGTTTTCTGATGACGCCGTTCGCCCTAAGCCGGAGCCGATTGCCTCTGGACCATTCGGTGGTCACTTGCGGAGCTTGTCCATGGACTCTGATTTCTTCAAGAATCTGGACCTTGGTGGTGATAGTGGGGAGATCGATTCTTTGGGGAAGAAGACTCCGGCGAGTGAACAGCGGCCAGTTCGTCATCGACATAGCCTGTCCATGGATGGTTCTTCGTCGTCGTTTGAGGCCGATTCAACCCTAGTGATTGATGGAGTGAAGAAAGCAATGGACCCGGAGAGACTTGCGGAGCTTGCCCTAATTGACCCGAAAAGAGCTAAAAGGATTCTAGCGAACAGACAATCTGCAGCTCGTTCGAAAGAGAGGAAGATACGGTACACGAATGAGCTGGAGAGAAAGGTTCAGACTCTGCAGTCCGAAGCTACCACTCTGTCTGCGCAGGTGACGATTCTACAGAGAGATACAAGTGGATTAACTGTGGAAAATAAGGAACTCAAACTTCGGTTACAGGCTATGGAGCAACAAGCACAACTTCGAGATGCTCTGAGTGAAGCTTTGAAGGAAGAAGTTCAACGGCTTAGAATAGCTGCAGGCCAGGTTGCATCGATCAATGGAAATCTTTTCAACCGACCTCCTCAATATCCTTCATCCCGGCCACCTGTACACCATTTTAGTAGCTCCCATGCCCAGCAGGGTCAACAGCAGCAGCCACCATCCATGTTGGCTACCAACCAGCAGCAGCAATCAGATCCCAAATGGACAAACTCGTCTCAGCTTCTCAGTCGAAGTCCGGATGGCCAAGCAAAGCCTTAG

>ClabZIP44

ATGGCAATGATTTCATCCGCTTTTTCTCATACCAAGGATGAAAGAGAGCTGAAAAGACAAAGAAGGAAGCAGTCAAACAGAGAATCTGCTCGTAGATCTCGGTTGCGCAAACAGGCTGAATGCGATGAGCTGTCTCAGCGTGCGGAAGCTTTGAAGGAAGAAAATGCGAGTCTTAGATCTGAAGTGGATAGGATTAGGAGTGAGTATGAGCAACTTCTTTCAGAAAATGCCTCTCTCAAGGTGATGCCAAAAACCTGA

>ClabZIP45

ATGAGTGGCAGTGAGATGGAGAAACCACCAAAAGACAAGGAGACGAAGACCCCGCCTCCTACTACTACCACACAGGAGCAGACTACAACCACAAGCGCTGGCACAGTTAATCCTGATTGGTCGGGTTTTCAGGCATATTCTCCTATTCCTCCACATGGTTTCTTGGCATCAAGCCCCCAAGCCCACCCATATATGTGGGGAGTTCAGCATATTATGCCACCTTATGGCACGCCTCCACATCCATATGTTGCAATGTATCCACCTGGTGGCATATATGCTCACCCTTCCATTCCCCCGGGATCATATCCTTTTAGCCCCTTTACAATGCCTTCTCCGAATGGAATCACCGAGGCCTCTGGTAATACAGCAGGAAGTTTGGAGGGAGACGTTAAACCACCCGAAGTGAAGGAAAAGCTGCCAATTAAAAGATCAAAAGGAAGTTTGGGCAGTTTGAACATGATTACCGGGAAGAACAATGAGCTGGGTAAAGCATCTGGAACATCTGCCAATGGAGCATACTCTAAGAGTGCTGAGAGTGCCAGCGAAGGTACAAGTGAAGGAAGCGATGCCAATTCTCAAAATGAATCACAACCTAAGTTAGGTTCCAGGCCAGACACTTTGGAAGTTGAAGTATCTCAGAATGGTAACTCAATGCATGGTACTCAAAATGGAGGATCTAATACTCAGGCTATGGCTGCCATCCCATTGGCAACTGCTGGGGCCTCAGGAGTCGTTCCTGGTCCCACGACCAACTTAAATATTGGAATGGACTATTGGGGTGCATCATCCACTATCCCTGCTATCCGTGGAAAGGTTCAATCGACTCCAGTTGCTGGAGGACTAGTTACCGCTGGATCTCGGGATAGCATACAGTCACAGCTTTGGCTGCAGGATGAGAGAGAGATTAAAAGACAGAGAAGAAAGCAATCAAATAGAGAATCTGCACGACGATCCCGGTTACGCAAGCAGGCAGAATGTGACGAATTAGCACATCGAGCTGAAGCTTTACAAGAAGAAAATGCCAGTCTTAGGTCTGAAGTGAACCGGATAAGGAGTGACTATGAACAGCTACTTTCAGAGAATGCTTCTCTGAAGGAAAGACTCGGGGAAGTTCCGGGAAACGAAGAAGTAAGGGCCGGCAGGAAAGGCCAGCGTTTAAGTAACGAAACCACACAACCTACAGAGTCAGAGGTTGTGCAAGTTGGTAACAAGAATTGA

>ClabZIP46

ATGGAAGAAGTTTGGAAAGACATTAATCTCTCTTCTCTTCACTCTCGTTCTGACCATGACTTCTCCGCCGCAGCCGCTGCTCCCGCCACTCCCATCGGCCTCCACCACCACTCCGCCGCCGCCAACTTCCGTCACATCATCCTCCAAGACTTCCTTTCTTCGTCCACTTCCAAACTTGACTCCTCCTCCGCTGCCACCGCAGCCGCCGTCGCTGCCCCACCACCTACTGTCTTGTCCTTGAATTCCACCCGGGAATTGCATTTCCCGGATAACGCCGTCGCCGCCGCCCACTTCCGCCACCAGGATCCTGCCTCCGTCTCCGCCGCTTTCCACAGCCCCTTTGATCAAGTCCTTGGACCACCGCCCTTTGGAAAGAAAAGGGTCCCTGATTCTGACAGCAATAACTCCGGTGATCGGCGCCAGAAACGGATGATCAAAAATCGCGAATCCGCCGCCCGATCTCGGGCCCGAAAACAGGCTTACACAAACGAGCTGGAGCTTGAAGTGGCACATTTAATGGAAGAAAATGCAAGACTTAGGAGACAGCAAGAAGAGCTACGTGCAGCAGCAACAGCGCAAGTCCCGAGAAAGCACAGGCTGCAGCGGACATCGACGGCGCCGTTCTAA

>ClabZIP47

ATGGCTTCTATCCCAAGGCAAACAAGCTCGGGATCTAATGGAGGTTCGCCATCTGCTCTTCCTGATGAGAGGAAGAGGAAGCGGATGCAATCCAATAGGGAATCGGCTCGGAGATCTCGGATGAGAAAGCAGAAGCAATTGGAGGACCTTGCTGGCGAGGTAAGCCGATTACAAATTGCGAATAATCAGCTTGTGCAGAGCATTGGTGCCAAGGAGCAAGCATTTGTACAGGTCGACAACATGAACAACGTTCTCAGGGCTCAAGCTATGGAACTTACTGATCGTCTGCGGTCCTTGAACTCTGTCCTTCATATTGTGGAGGAGGTTAGCGGTCTTGCCATGGATATCCCTGAAATACCCGATCCTCTCTTGAAGCCTTGGGAGCTCTCTCGGCCAGTTCTGCCCGTTGCTGACATGTTCCTGTGTTAG

>ClabZIP48

ATGGGTAGTAGAACAGTGAAGATTGGTGCAGACGATTTAAACAAAACGGTGAACGGGATGCCTAGCTTCGTTTCTTCGATGCCCACAGCCAATAATTCCATGGGTGCAGAGGGGAGCTCTATTCCTTCATCTCGGATTTCAGACTTTAGAACACTTGAGCAAACTCTTGGATTTCACATAGAAGATGTAGACCTTACTAGAAATCCTTTGTATAATCAGATAAAGTCCAGTAGTTCGACTCTGAACAACATCCAATTTGGTTCTTTAATTAAGCCACTTGCATCCACTGATGTAAATCTGCCAACTGCTGTCATGGGGTCTCGAACACTGCCATTACAGAAGGAGTCAAATCCAAATTTGGTTTCTACTTCTGGTGGTCCTCGTGAAAATTGGGGAGAGTCTAATATGGCAGATACCAGCACAAGAACTGATACCTCCACAGATGACACGGATGACAAAAGTCAAAGGCTTGACAAGGATCAAGGAAACTCACTTGCCGCATATGATTCTAGCAACAAATCAAAAGAAAAAACAGCAGATCAGAAGACCTTACGAAGGCTTGCTCAAAATCGCGAAGCTGCTAGAAAAAGCCGTTTAAGGAAAAAAGCATATGTGCAGCAGCTAGAAAGTAGCAGACTGAAGCTTACTCAACTTGAGCAAGAACTCCAGCGGGCCCGCCAGCAGGGTATATTTATTTCAAATTCTGGAGACCAAGCTCATTCAATGAGTGGGAATGGGGCCCTAGCATTCGATGTAGAATATTCACGTTGGTTAGAGGAGCATAACAGGCTGATGAATGAGCTTAGAGCTGCAGTAAATTCACATGCTGGTGACACTGAACTTCGTACTATTGTAGATAATGTGACTACACAGTTCGATGATATTTTCAGGTTGAAAGGCATTGCAGCCAAAGCTGATGTGTTCCACATCTTGTCAGGAATGTGGAAGACACCAGCAGAGCGGTGTTTCCTGTGGATTGGTGGCTTCCGTTCATCTGAGATCCTAAAGCTTCTTGTAAACCAATTAGAGCCTTTGGCTGAGCAGCAGCTAATGGGCATTTGCAACCTGCAACAATTATCACAACAGGCAGAGGATGCTCTCTCACAAGGAATGGATGCATTGCAGCAATCCTTGGCTGAAACATTGGCCAGTGCCACACCTGCAACGTCGGGATCGTCTGGGAATGTGGCAAATTATATGGGTCAAATGGCCATGGCAATGGGAAAGCTTGGAACTCTTGAAGGCTTCCTACGACAGGCTGATAATCTTCGTCAACAAACCCTTCAACAAATGCATCGCATATTGACAACCCGTCAATCAGCCCGTGCACTTCTTGCAATAAACGACTACTTCTCCCGTCTTAGAGCCCTCAGTTCTCTGTGGCTTGCACGGCCACGAGATTGA

>ClabZIP49

ATGGCTGACCCAATTGAGCTCGTTTCCCCCTCCGATCAAAACCCTAATTCCACTACTTACGCTTCCGAATTTGATTCCCTCCCAATTCCGCCTCTCGACTCTCTGTTTTTCTCCGATCCTAATCACGACGCCCCCGGTGACCCTTTTCTTTATTCCACCGCCTTGGATTTGGGATTCGAAGAAAATGATGATTTCGAGCTCACCTTTGATGACCTTGACGACCTTTACCTCCCCTCTGAGGCTGACGATTTCCTCATCTCCGATAATTTGGATCAGACTACCAATTCGTCGAACTCCCCTCCTGATGTTCCTCTCCTGTCTGATGCTGGTACTCGTCTTTCCAGCCCTGCAGGCTCTCCAGGATCGAGGAGCTCTTCGGTTTCTTGTGAGCAATCTCCCGGTGATTGTAAGTCTCTAAACTATCAATCTTCGAAATTAAGAACAGCGGATAGCGAGTGTTTTTCGACTGGTTCTGGTGGATGGGACTCTAAGCGTTCGAGGATGGTGAATTGTCCTTCCCCGGAGGATGGTGCCGGTGGTAGTGATCATGAATTCTCAGGCGGCCCAGCATCGTCGCAGGGCTCGGGTTCGGGCGTATCTGAAGGAACGAATTACCCATCTTCTAATGCGGAATGTTATGATGTTATTTCCGACCAGAAGATTAAATCAGAGGAAGTGGGGAAAAATTGCATCACGAAGAGGAAGAAAGAACAGGATGAAGGGAATGCGGATTCAAGATCTGCCAAGTATCGAAGGTCATCTGTACCTGCAGAAACTACCAATCCCCAATTGGGTTCCTGTTCTGTAAATGAAGATGATGAGAAGAGGAAAGCGAGGTTGATGAGGAATCGAGAGAGTGCGCAGCTTTCCAGGCAGAGGAAAAAGCATTATGTGGAGGAGTTGGAGGATAAAGTTAGAAACATGCATGCAACCATTGCTGAATTGAATAGTAAGGTATCTTATATGATGGCTGAGAATGCAGGTCTAAGACAGCAGCTGAGTGGTAGTGGTATGTGTCAGCCTCCTCCTCCTCCTCCTGGTATGTATCCTCATCCTTCAATGCCTCCAATGCCTTATACATGGTTGCCATGCGCTCCTTATGTTGTAAAGCCACAAGGGTCTCAAGTCCCTCTGGTTCCAATTCCTAGATTAAAGCCCCAACAACCTGCTCCTGTAGCAAAGGGGAAAAAGAATGAGAGTAAGAAGGCTGAGGGAACAACTAAGAAGGTTGCCAGTGTTAGTTTACTGGGTCTAATGCTCTCTTTTATGCTTTTTGGTGTTCTAGTTCCTTTAGCGAATGTCAGATTTGAAAATGTTGGAGGAGGTCCTGGTAAGTTGTCATTTGTTGGTGATAGGGTATACAATAGTAATCGGAGGAGAGTTTTGAGTGTTGATGGATATTCCAATTTATCAGATGGTGAGAATGTAGGAACTCCTTGTGGGAAATCTGGTACTTCGAACCGCTTACAATGTGAAAGAAATTATAGGAAAGGGCGAGACTTGAAATACAATCAACGAGGAAAGGGATCGCAACGTTTAAATGATTCAGATGAGTCTATTAAGCTTGGAAATGCTAGCGAAGCTCTCGTTGCTTCTTTATACGTTCCAAGGAATGACAAATTAGTGAAGATCGATGGAAACTTGATAATTCATTCTTTCCTAGCTAGTGAGAAAGCTATGGCCTCTCGCAGGGCTTCTGATACCAACAAGGCTAGGGAGACTGGTCTTGCAATTCCTAGAGATCTTAGTCCAGCCCTCACCGTCCGAAACATCAGGGCACTTACTTCTGGTTCAGAGGACCATATCAAGGCAACTGCGGCTGATGGTAAACTCCAACAGTGGTTCCGTGAAGGTCTTGCAGGACCGATGTTGAGTTCTGGTTTGTGCACTGAAGTGTTCCAGTTCGATGTTTCGTCAACAGCTCCAGGAGCGATAATTCCAGCATCTTCCATTGCCAATACTTCTAGAGCACATCGTAAGAATGGTACTCATCTTAACAAGGCAAAGAACAGGAGAATCCTGGGTGGTCTTCCAGTTCCCTTAAGTGGATCAAACTTCAACATCACAGAAGAACCTGTTAGAACCCCACACAAAGACAACTTTCCAGGTAACAATAAAACAGCTTCGTCGATGGTAGTTTCTGTGCTCATTGATCCAAGAGAAGCTGGTGACAGTGAAGTTGACGGCGTGATTACGCAAAAGTCTATTTCAAGAATTTTTGTGGTCGTGTTGCTGGACAGTGTCAAGTATGTCACATATTCATGTGTTCTCCCCCGCTCGGGCCCTCATCTTGTGTCTACTTAA

>ClabZIP50

ATGGCTTCTTCCAATATTAAAAGCACCTCCATCATCCAACAAGCAAACCAGCAAGAAGAAGAAAAACAACAACAACAACAACTTCTTCTTCATCATCAACAACAACAACCTCAACTTTTACAAACTCCAAGCTTTGTCATCTCATCAAACTCAAAAAATTCCTCATTATTCAACTTCCAAAACCCCAATTTTCCTGATCATCATCATCATCATCATCAATTACCCTTCTCCATGAATCTCCCTTCCATCCCTTCAAATTTCTTAAGCAAAGATGGAGGAGGAGATGATTTGGGGGAGTTGGATCAAGCTCTTTTTCTCTATCTTGATGGACAAGAACCCTCTACAACCACTACTCAAGACCAAAGACAGAGTTTGGGCATGAGACCTCCGACTTTGAACATCTTCCCGTCGCAGCCGATGCATGTCGACCCGTTACCAATAAAAGGAAATACAGCATTGATAAACCATAGTGATTCAAAGATCAAAGCATCTGAGCCGTCCAGAGAGTTGGCCAACCAACGCAGCAGCAATGGAGCCACTGCATCTGGACCACCCCCAACCCAAACTACAACAACCGAACCTCATCCCAAACCTCCCAAACGTGAGCCCAACAAAAAAGGACTAACCTCAAGCTCAGAACAAGAAGGACCCAAAACCCCAGACCCCAAGACCTTAAGAAGATTGGCTCAAAATCGAGAGGCCGCTAGAAAGAGCAGACTTAGAAAAAAGGCGTATGTTCAGCAGTTAGAGATGAGTAGGATTAAGCTCACCCAGCTGGAGCAAGAACTACAAAGAGCAAGAACTCAGGGCATGTTTCTGGGTGGAGGAGCCGCCATTTTAGGACCTGACCAAGGACTTCCCTCTGGCTTCTATAACCTCAGCTCCGATGCTGCGGTGTTCGACATTGAGTATGGACGGTGGCAAGAGGAGCACCACCGGCTAATGTGCGAGCTCCGAGCAGCAGTGCAAGAGCAGCTACCGGAGAACGAGTTGCGGTTGTTCGTGGACAGCTGCCTGGCCCATTACGACGAGGTTCTGAACTTGAAGATGATGGTGGCTAAGTCGGACGTGTTCCACCTTGTCTCCGGCATGTGGAAAACGCCGGCGGAGAGATGCTTCATGTGGATGGGAGATTTTAGGCCCTCCGAACTCATTAAGGTACCCTTCACTATTATCATCTGA

>ClabZIP51

ATGGTGGTAACAGAATCAGAAATGATTTCACATGATGAAGTTGAATCGCCATTGCAGTCTGAGCAGCAACTCAAGCACCATGGATTCTCTTCCCTTGGAAGGCAATCCTCCATTTACTCGCTTACACTGGATGAGTTCCAGCATACTCTTTGTGAGAGTGGCAAAAATTTTGGCTCCATGAACATGGATGAGTTCCTCACCAGCATTTGGACGGCGGAGGAAAACCAAGCAATTAACGCCAGCCAATCGGGCACGTCTACCGTTGCTGCCATGGCAGCATTAAGCAATGCTCAGGCTCACTTGCCGGTGAGTGGAGCGTCCATGGAGAAACGCAACATAGAGAAGCAAGCAAGCTTACCCCGCCAAGGTTCGCTTACACTTCCTGCACCCTTGTGTAGGAAAACTGTGGATGAGGTTTGGTCTGAGATACACAAGGGCCAACAGGGGCGGAATCAAAATAGCAACAGTGGTAATGCTAACTCTCAGAATCCGGAATCTGCCACACGCCAACCGACGTTTGGAGAGATGACATTGGAGGATTTTCTGATCAAAGCTGGGGTCGTTCGAGAACCTTGCGCCGGGGCAGGAGTGTCGCAACCGTTGCCACCGCCTCAGCAATATGGTATGTACCAGAATAGCAACCACACCATTGGTGCTGGTTATGTTTCCAGGCCTATTATGGGACTTAATACATCTGCAGCTGGTGGTGGTGCTAGCAATAATCCCAGTGCCGGTGGCATTACGACATACCAACCAGTCCCCCAAGGTGGTTCTACTATTGGAGATACATCAGGATATGTTGGCAATGGTAAAAGAAACAGTGTGTTCTCATCACAGCCTCCACCTGCAGTTTGTTATGGTGGAAGAGTGGTGAATGGTGGTGGAGGTGGTGGCGGGGGAGGAGGATATCCACCGGCTCAACCAATGGGGTTGGCAGCACCTGTAAGTCCAGTATCCCCAGAAGGAATGTGCACAAATCAGGTGGATAGCTCAAATCAATTTGGGTTGGACTTGGGTGGGTTGAGAGGTAGGAAGAGAATAATTGATGGGCCAGTTGAAAAAGTTGTGGAAAGAAGACAGAGAAGGATGATAAAGAATAGAGAGTCTGCTGCAAGGTCTAGAGCTAGAAAACAGGCCTACACAGTAGAGTTAGAAGCAGAGTTGAACCAGTTAAGAGAGGAAAATGCTCATCTTAAGCAGGCCTTGGCAGAGCTTGAGAGGAAGAGAAAGCAACAGGTACCTTTTCCAAAACAACTAGCTAGATTCTGCTTGATCCCTAACCTCATACAATTTGAAGCAATGTACTTGGAGGAAACGAAGAATGTTCACACGAAAGCACAAAGAGCCAAGGAAAAGCTTCGTGTTATGAGGAGAACTTTGAGTTGTCCATTGTGA

>ClabZIP52

ATGGCTTCTTCTTCCAAGTGCTCCGACGGAACCACTTGTTCTGGTTTGAGTTCTTCGTCTTCTTCTTCCTCCTCCATGTCCTCTTCTATGGCCAAGGCGGCGGATCAGATGGTCAAGGTTGAGATTGAGGCGGCGGAGGCTCTTGCTGGTTTGGCGGTTTTGGCGGTCAGAGAGACGGGACCTCAACCGTCCGAAACCAAATGGGGGATTAAAGGGAAAGGGAAACGAGCTAGGAAGGAGGTTAAGACCGAGTCGCCGACTTCTGCCTTTGCCGACTCTTTACCTAGTCGCGCGGATCTGAACCTTCGGATTGAGCAGGAAGATAGAGGGGTGGTAAGACATCAGCCATTAGAAAAAGAATGTACTAGTCAGTCCCACCCTGAGTGGGAAACAACTGGAGAGATGATGAAGGTAGACAAGGAGGCCGAATCATGTAAAGTGAGTCCTGCATGCACCACAAGCTACCAGTTATTTGGCTGCAGGAGATCAAGACGTACTCTAACTGAGGCTGAAAAGGAAGAAAGGAGGGTACGAAGGATTTTAGCAAACAGAGAGTCAGCCCGGCAGACAATTCGGCGTAGGCAGGCTCTGTGCGAGGAGTTGACCAGAAAGGCTGCTGATCTAGCATGGGAAAATGAAAATTTAAAGAGGGAAAAGGAGTTGGCCCTGAAAGAGTACCAATCTCTGGAGACCACTAACAAGGAACTAAAGGAACAGTTGGCTGAAGCAGTAAAGCCGAAGGTGGAGGAGATCCCAGGAAACAATAGATCATCTCATGTTCAGATGCCTCCTTTACCTACCAACTACCCTCTTTTCTTGTTTAGTCGCCTTCCATATTTCTGGCCATCTGTGGTTCAACCTACAAGTCCCTATCATGAACTACCCAATGTTGTCGTCGTCCCGTCAAGTATTAATTTGCCTGCAAATAGTAATGTTTCTGTGTCTGGCTCTTCTCATGTACAAGAAAACTTTACAAACGCCTGTGGCCCGAGAACACCCTTGTGTATACTACCACCTTGTTCTTGGTTGTTGCCTCATCATGATTTTAGGAACCAACAGAGTCCTCAAATCTGGTTTCCCGCTGGAAATAATCTAGAGGATATTTATTCGAAATCCCAAAACAGTGCTAATACTTCAAAGGTTGTGTGTGCAGAAAGCAGACAGTCTTCTTTGCCTTCAGCTGAAGAAGAAAACGATGCTCCTGACTTGAATGAACCTCCTAATTTAAACGAAGCTTCGAATCCAAAGGATCATGCTCAGAACTCAGTTGGAGTATCTGTAGATGGATTTGATACCAACGCAAGACCTCAAGTTAGAGAAGTACTTTCTCCTGTAAGACTTGAATGTATCGAATCCAGTTCCGCTGTCAAACAAGGTAACCGGAGCGAAGATGATCACGGTCTGTCATCAAGAACTTGTGATGACTTATTTGATTTTGCGGAAAGAAGGCACAAACCAGAGATAGCTCCCTGTAAGAAAACCATAGATGCAATGGCTGCAACTGAGGCAAGGAGGCGGAGAAAAGAACTCACAAAGTTAAAGAATCTTTACGCCCGTCAGTGCCGTATGCATTCCTGA

>ClabZIP53

ATGAGCTCTTCATCAACTCAACTCTGTGCTTCAAGGATGGGCATCTATGAGCCATTTCACCAGATCAACTCATGGGCAAATGCATTCGGTAGTCGACTAGATACGAGCATATCACCTATTATAAAAGTGGATGACTGTGTAGACATTAAACCCGAGTTTGTTCCTTTTGAATCAATGGATCATCTCGAGAGCAGTCAAGAAATGAACAAGCCAATCGATGATAAGGTACAAAGACGTTTAGCACAAAATCGGGAAGCAGCTCGTAAAAGCCGTATGAGGAAAAAGGTTTATGTGCAGCAGTTAGAAACTAGCCGTTTGAAGCTGCGACTGTTGGAGCAGGAGCTAGAAAGAACTAAGCAACAGAAGGGTACCAGCTGCTTAGTAGATATCAGCCACTTCGGATTCAGCGGATTTGTAAACCCAGGGATTGCTGCATTTGAGATGGAATACAATCACTGGGTTGAAGAGCAACAGAGACAGATCACCGAACTCAGAAAGGCGTTGCAAGTTCATACAACTGATATAGAACTCCAAATTTTAGTGGAGAGCAGCCTAAACCATTACCACAATCTGTTTTGCATGAAAGCCAATGCTGCAAAGGCTGATGTTTTCTATTTAATGTCTGGTATTTGGAGAACATCGGCAGAGCGTTTTTTCCACTGGATAGGAGGATTTCGCCCATCAGAGCTGCTAAACGTTCTGAAGCCATACTTTGAGCCATTAAATGAACAACAAAGAGCCGATATCCACAAGTTGCAACAGTCGTCTCGGCAAGCTGAAGATGCGCTCACACAGGGAATGGAAAAACTGCACCAGAATCTATCCCTCAGCATTGCCAGTGATCCTATAGGAAGCTATATTTCTCAGATGGGTGATGGAATGGAGAAACTAGAAGCACTGGAGAGCTTCGTAAGCCAGGCAGACCATCTCAGGCAACAAACATTGAAGAGAATGTCTCACATCCTAACCACCAAACAGGCAGCACAAGGCTTGCTCGCTTTGGGCGAGTACTTCCACCGTCTCCGCATCCTCAGTTCGCTCTGGGCTACTCGACCTCGTGAACCTGCCTAG

>ClabZIP54

ATGGAGAATTCCAAGGTGTTGTCAAACATGAGAAATATGATTTACTCTGGAAAGCATGCTCTACTTCCTCCTAAGAGTCCACTTCCTAGTGGGTCATCCTCATATGCTGATTATTTCCCTAATCCCATTATTGGGTCAAGAGCAGTGCAGAATCCCAGAGAGGGAAATGTGCACCATCATAGAACATCATCTGAAAGTCTTGTGATGGAGGAACAACCTTCTTGGCTTGATGATCTCCTCAATGAACCCGAAACACCTGTTCAAAGAGGTGGTCATCGACGTTCATCAAGTGACTCCTTTGCTTACTTAGATGCAGGAAATGTTTCAAATGAAAATTATACACAAGATGACTCCCAATGTAAAAATATGTATTTACCTTCCTGGGCATCTCAAGATTTTGATTCCCATCAAGCTTCATTTCATATGAAAGCAAGCTGGATCAAACAGAAAAACAGGACACGGGAATTGCCTCCAACTACATTGACAACTAACCCAGGTGCCCGCTCTTCTGCGAAAAGTAGCATTCTTCTTGAAAGCTCAAGGTCGTTGAGTACTACACCACAGGAAGCAAATGGGTTTTCCTCAACTACTGAAAAGCAGGATTCAGCAGAAACTGGTCTGCCTGATAGGAAGCCATCTGAAAGAATGGATAGTTCTCATGTTAAGCCAGGTCTGGCTGATACAGATAATAAAAGAGCTAAACAGCAATTTGCTCAACGTTCACGTGTACGGAAACTTCAGTACATTGCAGAGCTGGAAAGGAACGTACAAGCGTTACAAGCAAATGGTTCTGAAGTTTCTGCCGAACTTGAATTTCTCAGTCAGCAAAACTTAATTCTTGGCATGGAGAATAAAGCACTCAAGCAACGATTAGAAAGTTTATCACAGGAGCAGCTTATAAAATACCTGGAACATGAAGTACTGGAGAGGGAGATTGGAAGACTAAGAATGTTGTACCAACAGCAGCAACAGCCACAGCCACCACCTTCCAGCCTTAAACGCACCAAAAGCCGAGACCTTGAGACGCAATTTGCTAAGCTCTCTTTGAGACAGAAGGATGCACGTTCAGGTTCCGAGTCTGTGGCCGGTCCAGTCCAAATCTAG

>ClabZIP55

ATGAATTTAGATGAATTAGTTTTCAGGAATGTGATGTCTGTGGAAGAAGCAGAGCTGGTACATAACCCTTCCTCTTTATCTCCTGCAGCAGCCACGGCTTCATTGTTTCTCGGGAAAAGAAACGACGACGCCGAACCGCAGCACGATCCAATGGCTGAGGTTTCAGCCCCGGCAGAAGGGATGGATTGGATGCATTACCAACGAGCAATGCTGATTGATTCCAAGTTGCCAGTTTCTCAAGCTGTTTACAATCATGGGAGTGTACCAGATATTGGTGTTTACAATATGCAGGCCATGTCGATAACGACGTCGGCCTTGTCAAACTCAGATTTCCAGGAAGGTAATTGTGGACGGAAACGAAGACAGCTGGATGATATAAAGGAGAAGACTATTGAAAGAAGGCAGAGGAGGATGATCAAGAACCGTGAGTCTGCTGCAAGGTCCAGAGCCAGGAAACAGGCAAGTTCATGA

>ClabZIP56

ATGCCCAATTTAACTCCTTCTCCTCCTCCTCCTCCGCCGCCCTCCGCCACCCACCACCACAACTCTTCTTGGGTCGATGATTTTCTTGACTTCTCCACCGCCCGCCGCGGCCTTCACCGGAGATCCATTAGCGACTCCATTGCTTTCCTTGAAACCCCATTTTCCGATCAATGTCGTAACTCCGCCCTTCTTCATCCTCCTCCTCCTTTTGACCGCCTTGACGACGACCAACTTATGTCCATGTTTACCGATGACATTTCCATCCCCATTCCTGCTTCCCCCTCCAATCCTTCCACTCCTTCCGACCAAAATAGTAACAACGACGACAAAAACCCAACCGAGGACGCCATGTTTCCGCCTCCACCGCCACCACCTCTGCCTCCGCATCAGTTGAAAAATGAACCTGGCGAGGTTGAAATTAGCTCCTCTTGCCAACTGCAGCAGCAATCTCAACCACCGCCGCCTTCCTCTGCCGCTGATAATACCATTGATCCCAAAAGAGTTAAGAGAATTTTGGCAAATCGACAATCAGCACAAAGGTCGAGAGTCAGAAAATTGCAATATATTTCTGAGCTCGAGAGAAGTGTAACCACATTACAGACTGAAGTATCAGCATTGTCTCCTAGAGTGGCATTTTTAGACCACCAAAGATTGATTCTAAATGTCGATAATAGTGCTCTCAAGCAGAGAATTGCAGCTTTGGCTCAAGATAAGATTTTCAAAGATGCTCATCAAGAGGCGTTAAAGAAGGAAATAGAGAGATTGAGGCAAGTTTACCACCAACAAAGTTTGAAGAAAATGAGCGGTAATCAAAATGGAGCTGCGCAACCGCAAGAACCGCCGTCCGGAGAAGGTACTCCGCCGCAAGCGGCTGAGATGGAGAAGGGGCAAGCTGTAAGTTGA

>ClabZIP57

ATGGAGGAGGATCTCCATTTCTCCGAATACGACTTAATAGGGCAGATTGATTGGAACGACTTCTTTGATGAATTCCCGGAGGTGGAGCTCCCTCTTGCCGGGGATTCAACATCGCCCCACGGATCTCCTGATTCCTTTTCTTCCTGGATCAACCACCTGGAAAACGCTTTATTGAATGACGATCAAGACAAGGGGGTTTCCCTTCCCACTCCTACCGATGATTGCTGCGATAGTTTTTTGGCTGACGTCCTCGTCGACACTCATGGAGGAGCCTCTGTAATCGACCTCGATTCCAATGCTTCCGATTGCGGTAACCAGTTTAGCAGTTCCCAGAAGGAGGATGGACATAAGGTTTCTCCCGCGCCTACTGACGATTCCTGTGGTAGTTTTATGGCTGAAGTACTTGTAGATACTCACGGGAGCTCCTCTGGGGTTGATGCTGTAGTTGACGTTCTGTCCAACGCTTCCGATTGCGGTGACGACTCGAACAATTCCCAGAAGGAGAAGATCGACGCAACAAACATCGACGATAGTGTAGGTGAAGATGTCGATGATTCGCTTTCTAAAAAGCGGAGAAGGCAGCTGAGAAATAGGGATGCCGCAGTAAGATCTAGGGAGAGGAAAAAGATGTACGTAAAGGATCTAGAGATGAAGAGTAAATTTCTTGAAGGTGAATGCCGGAGATTGGGGCGTTTGCTCCAGTGTTACTGTGCTGAGAATCAAGCCTTACGGTTTAGTCTACAGATGGGAGGTGCTTCTGGTGCTTCACTGACCAAGCAGGAGTCTGCTGTGCTCTTGTTGGAATCCCTGCTGTTGGGTTCCCTGCTTTGGTTAGTGGGCACCGTTTGCCTGTTCACCCTTCCTCAACTCCCACAATCAACTCTGGAACCTGTTCCAAGAGTAACAATGGAGGACGAAGGTCCAGGAAGCGCGCCTCTCAACGAGAATGAAAATAAGGACTCCAGATACTCATACACATCGTTACAAACTAGGAGGTGCAAAGCGGCGAGAACAAGGATGAAACCAAGTATGTTGGATGCGATGCTCGGTCCTAGTTTGGCTCTGATTTCGGTATAA

>ClabZIP58

ATGCAGCCTGGTGAAGTCACAAGTCTCCAATATCTCATCCCCTCCAATTTATCTCCATATGCTACTCACTTTCCCATGGCACAGAACAACTTACCTACAATGCAACTTAACGAATTCTCCAATAACGAATTCTCCAATCCTTTATATAATTTCCAAGGTCCTTCTCAAGTTCATGATTTTAATCGACACCCATGCTTGAGTAGCAATTCAACTTCTGATGAAGCAGATGAGCAACAGCAAAGCCTCATCAACGAGAGGAAGCAGAGAAGGATGATATCTAATAGAGAGTCTGCACGCCGGTCACGTATGCGCAAACAGAAGCATTTGGATGAGCTATGGTCTCAAGTGGTTTGGCTACGGAATGAGAACCATCAGCTTATAGATAAGCTGAACCAAGTTTCAGACTGCCATGACAAGGTTGTTCAAGAAAATGCCCAACTCAAAGAGCAGACTTCTGAACTCCGTCGAATGCTCACAGAAGTACAGGTCAATAGTCATTACCCCAACTTCAGAGAACTTGAAAAAATCCCCCCCAACACACCATCTGAGAGCTGA

>ClabZIP59

ATGGCTTCCTCTAGTGGAACATCTTCGACTTCTTCATCTATGGAAGAAGGGGAATTAGCGGCGTTGATGGAACAGAGGAAGAGGAAGAGGATGATTTCGAATCGGGAATCCGCGAGGAGATCGAGAATGAGAAAGCAAAAGCATTTGGATGATTTAATGGCTATGGTGGGTCAACTCAGAAAGGATAATCAACAAATCGTCGCTAATCTCACCGTCACAACGCAGCACTACGCCGCCGTCGAGGCTGAGAATTCCATTCTCAAAGCTCAGGCCGCCGAGCTCTCTCACCGTTTGCAATCCTTGAATGAGATCGTGGCTTTCTTGAACCCCTCTGATGGGGTTTTGGAAGATGATACCTACGGCTGCGACGGCGCCGGAGGGTTTTTTAGCCCTCTTCAAATGGCTTTCTATATGAGTCACCCTCTTACAGCTTCTGCAGATGTATTTGGAGAGTATTGA
