# Supplementary material for: The bZIP gene family in watermelon: genome-wide identification and expression analysis under cold stress and root-knot nematode infection
Source: PeerJ. 2019 Oct 16;7:e7878. doi: 10.7717/peerj.7878 (PMC6800529; doi:10.7717/peerj.7878)
Supplement: Supplemental Information 6 [file peerj-07-7878-s006.doc]

**Table S4.** The gDNA sequences of *ClabZIP* genes.

>ClabZIP2

ATGGCTTCCTCCAATGTGGAGATGGCTTCCACGTCCAAGACCAATCCGGATCTCCCGCGCCAGTCCTCTGTTTGTTCTATCTCCACAATTATCGCTGATCTACACCATACTGATCCCTCCCGGAATCTGGCTTCCATGAGCATGGATGACCTGCTTAAGAACATCTATTCCGATGCTCAAACTCACAATCAAGTTCAGGATGAAAATCCTATTATTGGCTCTTCGATCTCGCCTCAAGACACTTTTCCGCTGCCTAAGGAATTGAGCACCAGAACTGTGGAGGAGGTCTGGAAGGAGATCGTTGCTGGGGGTGATCAGCAGAGGCGGGAGTCTGCTACGGATCAGGAAATAACCCTTGAAGATTTCTTGTCTAAGGCTGGGGCTGTTTGTGACGACGATGTGAGGGTTCCTGTCATTTCTGAACCTGGGGGTTTTGCTGTGGACTCCACGCTTAATAATCAGCTTCAGATTCCGTCGCAGCAGTTGGAAGGTCCCATGGTGGGAGGATATGCGAGTGGAATCGATGGGAGAATCGTTGGAGTTGGAAGAGGGAAAAGAAGGGCTGTTGAGGAACCAGTTGACAAGGCCACTCAGCAGAAACAGAGACGGATGATCAAGAATAGGGAATCCGCTGCTCGTTCTCGGGAACGCAAGCAGGTTAGTGATTTCATCTTATATCGAAATTCTTCCTTTATTTTGTTTTTTTGTCTTTCCGGGAGGAATACAGTAATTGAGTTTTTATTTATCGTAATTTCCTTGTTTCAATGCATCACTCGTTCTGCAAAGAGTGTTGTCATTTTGGTTGTCTTGGAAACTGAGTTATTGAAGTAGGTTTGGATTTTTGAATTTCTTTTTGAACATTCCAACGAGATTTAATGTAATGCGTTCATGTTTAGGCATATACGCTAGAGCTAGAGTCTCTTGTGACCCAACTGGAGCAGGAAAATGCAAGGCTATTGAGAGAAGAGGTACCTCTGAATGAAATTTGTGTTATTCGTTCTTTTAGTGGATATTTGGTAGTTGTTTTCCACATTTAATCAACTTTAGCATCACTACATTTGTCACCCCTTCCTTTCATGCTCCATAATACCTATCGATTGGATAACCAGTTGGACTGCATGTTCTAATGGCAGTATTGGCCACTCAAGGAGATGAGTCTCAAGTGA

>ClabZIP1

ATGGAAATCTCAGGAACCCATTTTGCAGCACCTGAAAATTATGGCATGATTCAAAATCTCAATATCAATGAATTATTGTCTTATATACCTGGTAATTCAACCTCTACTTCTGATGATGGTGATGATCTGAACCACAAACTTGGAGTTGTCATCGACGAGAGGAAGCGGAGGAGAATGATATCGAATCGGGAATCGGCGCGAAGGTCCCGGATGAGGAAACAGAAGCATCTTGATGAACTTTGGTCACTAGTGGTTCGTCTTCGTATGGAGAATCATAGTCTTATGGAGAAGTTGAGTAAGTTAACAGATTCTCAACAACAGCTTCTTCAAGAGAATGTCAAGCTCAAAGAAGAGGCGTCGGATTTTCGTCGGATGATCACTGACATTCAAATGGGCAGTCCTTACATAACTCACTTGAGAGAGCTGGAGGAAGCCCCCTGCAATACCTTTGATCTCATGGCTGAATCCTCAAGCCAATCTAACGAGTAG

>ClabZIP3

ATGCTGTCCACTTTTCCCACCGCCTCGGCGTACGAGTCCATGCTCGAAAACCCATTTCCGGCGGTTAATGGCACTTCCACGCCGTGGGAATGCCATGATCACGATCACGACCCCGAGTTTTTGCCGGTTGTGTCCCAGATGACGGAACCAACGGGATTATTTGACGTTTTCCACTCCCCAAACCCGGTGATTTCCAGTTCGAGTTCCGAAAACCGGGACGAACCGGAACCGATGGATGCCGGTCCGTGTGCCCCGGACCGGAAAGTTGAGGTGATAGACGAAAGAAAACGACGGCGGATGGAGTCGAACCGGGAGTCGGCGAGGCGGTCCCGGATGCGTAAACAGAAGCACTTAGAAAACTTAAGGAACTTGGTGAACAAGCTGAAAGTAGAAAACCGGGAGCTGTCCAACCGGTTGCGGTTCACGGTTTATGAAGTGAACCGTGTACGGACAGAAAACGACCATCTGCAGACAGAACACACAATTCTGAGGCAGAAACTTTCGTATAGCCGACAAGTATTAATTTTCCGGCAATTCCAACGACAATTGGCTTTTGCTTCCCCTTACAGCGCCCTTGAACAACCGCCAACTATCTCCCAATCGCAATCGCAATATATCCTTAATAACAACAACAACAACCTCATTAATTGCTAA

>ClabZIP4

ATGGGGACTGGGGAAGAGGGCACACCTTCTAAAACTTCCAAACCTCCCTCGTCATCCCAGGTGATAGAACCTTCCTTCCTTATGCCAGTTTTCACTCTCAAGTTGTTCATATCTTGATATATTTTGATTGTTACGCACAGGAAATAGCTCCAACACCTTCATATCCTGATTGGTCAAGCTCAATGCAGGTTGATATGGATTTTTTATTTCATACTAATTGCCGTTTGAATTCATTAATCACCTAGAATAGTAGTAAAGCGAGCATAGATCAACTGGTATACTTATGTTAGTAACCACGAGGTTTGTGGTTCGAATTTTCCACCCCAATTGTACTAAAAAAACACCTAGGATTGTAGCAAACATCTTGATGAATTTTGCCGTCTCAAAGTTTAGCCTTTTTGTAGGCTTATTATGGTGCTGGTGCTACTCCACCTCCCTTCTTTGCATCTACTGTTGCTTCTCCAACTCCCCACCCTTACCTCTGGGGAGGTCAGGTACTTGTAACTATCTATTGCCCCACAAGGTCGTATGGAGTTTGATATTATCCAGAGAGGACAGGACAATAATGTTTCTATTTTTTTAATTTTGTGTTAACATTTTTCAGCATCCTTTGATGCCACCTTATGGGACTCCAGTACCTTATCCAGCTATATATCCTCCTGGGGGAGTTTATGCCCATCCCAATATCACCGTGGTAAAGTCCATTTTACATGATGGGCTAGTGGATTTTATTCATATTCAGATCAGTGAATCTTTTTCTCTTTATGGTTTCATCTTAATATCATATGCTCTTTGCCTGATCATTCCTTCTTAAATACCCACATGGATCACTAGAATAGGATAGAAGGATTTAAGTTGGTTCTTGATATACTTGTGTTTTGCTTGTAGACTCCTGGCTCTGTACCAATTAATGCAGAATATGAAGGAAAATCCCCTGATGGAAAAGAAAGGGCCTCAAAAAAATCCAAGGGCATGTCTGGAAATACTGCTTCAGGTGGTGGTAGGACCGGGGAGAGTGGAAAGGTGGCTTCAAGTTCTGGAAATGATGGTGCTTCTCAAAGGTGTTTTAACTTGAGGCTAAGTGCAGGTTGTATTCTGTTTTTAAACCAATCTCATCTGATTAAGAATTTCTGGTATTTACAGTGCTGAAAGTGGTACCGAGGGCTCATCAGAAGGCAGTGATGAAAATGCTAACCAACAGGTATGTGCCTTTTTCTCTTCAGGATCTTTCAATTATCTGTACTGGTTCAACTTGTACTAGTGTGAGAGTATTCCCATTTCTTTTCAGGAATTTGCTGCAAATAAGAAAGGAAGCTTCAACCAGATGCTGGCAGATGGTATGATTTTCAATTTTAATTTAAGTTATACTGCTCATATATGCTGCTGTCGATGCTGTAAATGAATTGCAATTTTCAGGAGCCAATGCACAAAACAACACGGGCGGACCAAATGGCAAATCTTCAGTGACTGGGAAACCCATCACCTCCATTCCTGGGACTAATCTGAACATGGGAATGGATTTGTGGAATACCACCACTGCAGCTCCAGGGGCTGGAAAAGTGAGAGGGAATGCCGTCTCCTCAGCTATTGTTACAGTACCGATGGTTGGTCGTGATGGTGTGATGCCTGAACAATGGGTTCAAGTATGAGCTACCTCCCTGAAATAATCTCTCGACTTCTCTTCATATTGATATTCTTTGAAGTCCTTAAACCATAGGGGGAACGGATTTATTTATTTTTGTTAAATTTTGGACATACCACCTATGGCAGTTCGTTTCTCATTTATACACTCAATCTGTCAGTATTTTACTTTTGGATACTCTGATATGCTGTAAATTTTCCTTGAAATTCTTCAATCTTGGAGTATCTGTCGATTGGTAGGACGAACGCGAGCTAAAAAGACAGAAAAGGAAGCAATCTAACCGAGAGTCTGCCAGGAGGTCGAGATTACGTAAGCAGGTATACATTCCCACATATGTTAACATTCACTCCGAATTTTGTTACCGTATTGTGTAAGTATATATCGAGCATTTACTCTGTGAGCATGATTTTCATGTCATCTCTTACTTTCTTCCCGCTCACGAATTATTTTCTGCTATTTTAGACAAGTATCACCTTTACATTATTTCTTTCGACTTCGAGTCTTTGTACTTAAGAAAATTTGCATATTCTATTGACCGAACTTTTTCATCATTATTCATGGGAAAGTTTAGGACGTATAGTATTTACTAGTAGACAAAGCAGCCGTGCGGAATGCAATTATTTAGATCATAAAAATGTGGAGAAATCAGATTCTTTTTGCAACTTTTTGACGTGTCACGTTCTTATGTGAACTGGGAAAATTTATGTTTCTACGGTTCTCCATCTTACTTCTATCCTTATTCTTTGAAAGCCATCGTAACGGAATCGTGAAGTCCATGAAATTAAAATAATATACTATATAATTGAATGACATGTCAAATTCTTAATAGAAGGATTATCCCGGACTAGTAGTAGCTGTAATGGGGAGAAATTTTGAAGTACGAGTGCTACTTTCATTAGGTGGCGGTTATCCCAACATTCTTTTGAAAATGAGGTGGAATCTACCGGACGAAAGCAAAATTATATTGCGAACAATAGGAGTTTTTTCTATTAGTTTTTAATGGTGTTTATTAATGACAGGCGGAGTGTGAAGAGCTGCAGGCAAGAGTGCAGACATTGAACAACGAGAACCGTACTCTTAGGGATGAGTTACAAAGGCTCTCCGAGGAATGCGAAAAGCTAACATCAGAAAATACTTCCATCAAGGTCAAATATTCTCTTTTCATTTTATGCTTGTTCTTAACGCAATTAAATCCTCTCCTGATAGGATAGATTCAACTCTCTTTATATTTGTTGAAACAGGAAGAATTGACACGATTTTGCGGGCCAGAGGCATTAGCTAACTTCGAAAAAGGAACCACCGCGCCGCCTGCTCAATCTCGAGGCAGCGGCGAGGGCAAGGATTAA

>ClabZIP5

ATGGCATCTTCAAATGGAGCGTCATCTGGGTCGGCCAACGGAAGCATCCGAACCTCAGGTTCGGAAGATGATCGGGAGGTTGAGAGGAAGCGGCGGAGAATGCAATCTAATCGGGAATCGGCTCGACGATCTCGATTGCGCAAACAAAAACATTTACATGATCTTACTCACCAGGTAATTTAATTATAATTAAGGAATAATAGATGCCAAATATTTTACTCCCTTCCTAATTAATTATTTCAAAATTTAAATTTGCAGAAACAAGATTGCCTACTAAATAATTGTCAGATTTTAAGTGATAGTAAAAAGGGTCTCTTGACAAAAAAATATATATATATATATTAATAATTGGCGACATATGAGGTCACTCCATACGTGATGTTGAACAGAAGTTTGGATTTCTTTTAAATACATATTAAAATATCAGAATAATAAGTAATATTTGGCCTCATTTATGTTTTAAAAGAGTGTTGGGCCTCCTAGTTTTTGAACGAGACTCATCGACATTTTCTTTAGGTGTTCTAAAACTGTCTTACTCGACTGTTACAGGTGAGTCAACTGAAGAAGCATAACAATGAGATGGTGACGAACATGAACGTGATGGCGAATCTGTGCGTCAACCTGGAGGCAGAGAACTCGATCCTTCGAGCTCAGATGGCGGAGCTTACTCACAGATTGCGGTCTCTAAACGATGTCGTAATGTTTATAAACTCCATGCAGGTTTTTGAAACATTTTTAGTCGATGAGGTTGGCGATTTGAATAATGGGTTCGAAGAGGAATATCATTACAACCCTTGGAGATATCCATTTGCAAATTACAACTCCAACACACAAATAATTGCCCATAAAGATTGA

>ClabZIP6

ATGGCACAGTTGCCACCCAAAATCCCAAATATGACGCCTAATTGGCCCGACTTTTCCCGGCAGAAAATCCCTTCAATGGAAACCTTTGCACCGCCCACCACCACGCCGGCGGTTACTCAAAATAATCCTTCTTGGGTCGATGAATTTCTCGATTTTTCTTCTGCTAGACGTGGATCCCACCGTCGGTCTGTTAGTGATTCCATCACATTCCTCGAAATGCCAATGCTCGAGGAAGATTGCCGGACTGCCGCTGCTGCACCGCCCCCGCCGCCCCCGGGATCCGGTGACCGGAATGAATTTGATAGATTCGATGACGAGCAATTCCTGTCGATGTTTAACGATGAAATCTCCGCCGCCGTCGCTCCCACCTTGTCGTCGTCGAACCCCTCCACGCCGTCGGATCATAATAGTATTAACGACGAGAAAGACGCCCAAAACGACGGTAAGCAAAATCAGAACAAAAACGAATCCGACGAAGTACAGAGCCAACAGCAATCGGAAAACCAAACACAATCCAATTCCATGGCGACGGCGGCAACGGCGGCTTCCACTGATCGGATAACAGACCCCAAAAGAGTCAAAAGGTGAAAACTTTATCATTTAATTCCCTCCATTAACAAAATAAAGCTCAAAGCTTTCATCTGGGTCTAAACAAATCTAAGAATTTTCAAATGGGGTTGTAATTTTTTTTTTTTTTTTTTTCATTTCTCGATTTAGAATTCTGGCAAATCGGCAATCGGCACAAAGATCACGAGTAAGGAAGCTTCAATACATATCAGAACTCGAACGAAGCGTTACATCGTTACAGGTACATTGCAAAGATCATTTCTTAATTAATAATTAGGAGTTCATAATCTCAAATTATCTCATCATAAATTTTTAAAATGCGAAGGCTGAAGTATCGGTGCTATCGCCGCGAGTGGCGTTTTTGGACCACCAGAGATTGCTTCTTAACGTCGACAACAGTGCTCTGAAGCAAAGAATCGCCGCCCTTGCACAGGATAAGATTTTTAAAGATGGTACGTAATTAATTACTCCATTATCTTCTTAATTAATTATATTTTACCCCCTTAATTATCTCATAATCAAACGGTAATTGGGTTACTCTTATAATGGGAAACATAGAGGAGGGAGTATGGAATTTTTTTTCATAATGCTATAAATTATTTTCTTTAGAATAAGCCAACTTAAATAGTAGCCATACTAATTAAGGCAAGAATTTGTTTCGGAATTTCTCAATTATGTCTTAGAGTTTGAAATTTTTATTTTTATCTTTATTTAAAATAAGATGTAAATTGAAATTTGGAAACTATGTAATGTGTAAAAATTGGTTACGTTAAAATCTATATTTTGAGATAAAATAAATAATATTAACAATTTTATTCTTTTATATAGTTGTATATCATATATAATATATTTTTTCTAATTTTTACATATTAATTATCACATCCATTTTACATTTTTTTTCTTTTTTTTGCAATAATTAGGTGTACTACACTCCCTTCTTTAACACAATTTTTAATTTTTTTTTTTTTTAAAAAAAGACTCATTTAAAAAATATTGTCGTGTGTCAGTTTTTTTCTAGACAGTTTTATAAGTTACACTAAGTATTTTTTTTTTTTTTACACGAAAAGATATTTTTTAGTACAATTTTAAAGTTTAAAGGTATTTTTTGAAACAAATTTTAAAATTCAAAAGGTAAAAATTAACATTTTCACTAAAGTCACCATTTAAACTTCAAAGTTTTGTAGTAATTTGTTAATTTAAACTTTGTTTAATAAACAAAAAATGAGGATATAGATTTGTAACTTCAATTTCAAACCATGAGTAAATGTATTTATAGGTGAAATATCCTCATGTTTGGCCCACCCCCACATGGTTGAACTCAATGACAAAAATTGAGGGTAGATAGTATATTCCTTAATTATATAGGACATTTTACATCTTTTTAATTAACAGATAAAATTTATTTACATGCATCTCATTTTTTTTACCGTCCAGTTTACCATGTAATTAACTTTCTTTCATTTTCTTAAAATATATTTTTGAGGTTTTCTATGAATGGCTACTATTTTGATATCTTTTTATCATCTTCAACTTCATGGAGGATATTTGGTTTTTGGATCATCATCAAGCTTTGGTCAATTTTATTAGATTTCTTTTTTTAGAACAATTTCACATATATGATAAGTTTTTTTTTTGGAGGGTTAAATAATGAATTGATGTTTATTAACATATTCTAAATATTTTAAAAGTATAAACGTGTGGGAAACTATATATCTTAATCAATTGATCAATTTTACTCAAGTTGACATTAATGTTTTTTAAAAGCAAGTATAAATTTACCTATAGGACCATTCAACTAGATATAATACATTAGTAGAAATTTTCATAAACTTTCTTATGTCTTAGGGAAAAAATGATAGTACTATATTGTAGTAGTAAAGCTTTTTGATACTACTTCAGTACTTCTATTCATGGTCTAACAGTAATAAATTATTATTTAACAATATATCAAATTATGAAGGGAAGTAGCGAGTAAAAAATTTCCTGGAATGGCAATGGAGCATGATAATAATTGTCCATTGACTCGAGGAGAGATAGTGAAGAAGCTAGAAAAAGAACGTGGCAGACACTATAAGGAAAGAGAAGTGTAACACTGTTTTGAATTATAAAATTCCTTGGCTGGCAGGCAACTGTAGCACTACTTCCTGTTTTCTCACGAAATTGCATGCATGATTTATAATCGATTCTAGACAAAGACAACCAATGAAATCATCATAATAATAACCTATAGTTTTTATTATTTTATTTTTTTACCATTACATAATACATTACCGTAATTTTATTTTATTTTTTTGCATGGATATGCATGCAGCTCATCAAGAGGCATTAAAAAGGGAGATAGAGAGACTAAGACAAGTATATCACCAACAAAACATTAAGAAGATGGAAAACAATGCAGCACCATCACCGGCGATTACTCCGTCCCGACCCGTCACCGCCATCGCCACAGATGCCAAACTTCCTAACATAGATCAAAACGAGCAAGTTCCGAACATCGTCGTTTAG

>ClabZIP7

ATGGACGACGGGGAGCTTGATTTTTCGAATCAGGAAGTGTTTTCCAGCCCTAATATGGAGATTCCAAGCAGTTGTTCAATGGATAGTTTCTTTGATGAACTTCTCAAAGACACTCATACTTGTACTCATACACATACTTGCAATCCACCAGGCCCTGACTACTCTCATACCCACACATGTTTTCATGTCCACACTAAAATTGTCCCTGCCCCATCTGAAGAAGACAAGGTTGTTACTGATGACACTGCAGAATCAACTGAGAAGAAGTCGAAGAAACGCCCGTTGGGTAACCGTGAAGCTGTTCGTAAGTACCGCGAGAAAAAGAAAGCTAGGGCAGCCTCATTAGAGGATGAGGTTGTTAGATTGAGGGCATTGAATCAGCATTTGATGAAGAGATTGCAGGGGCAGGCAGCACTCGAGGCTGAGATTGCTAGGCTCAAGTGTTTGCTTGTGGATATTAGGGGCAGAATCGAAGGGGAGATTGGTTCTTTCCCTTACCAAAAAGCTGTTAATCCTAACTTGTCCAACCCAAGCATCCCTGGTGCTTTTGTGATGAACCCATGTAATATGCAGTGTGAGGATCAGGTTTACTGCCTCCATCCCGGGGTCGATGGCAACAGAAGCAGTGAAGGTGCTGTGATAAATGGACAAAGCTTTGGTGCTTGTGAATTTGAAAATCCTCAATGTTTGGCAAATCATGATTCGGGATCAAAAGAACTACCTGGGTGTGGAGTAGGGAATGCAGTTTCAAATGATATTTCTTCAGGCAAGAAGAAAGGTGAAAACTGA

>ClabZIP8

ATGGGTGATACTGAAGATGCTAACACTGAGAACATGCGAAATCTTCAATGTTCGTATGGAGTATCTTCTTCTTCAGCTGGAAATCTTCCTTTCTCGATGGATCAACTTAAAATTTCCCAAATGAACTGCTCACAAATCCGTCCACCGCACTTTCAGTCAAATTTTCTTGCGGATAATAGTAGAAGAATTGGGATACCTCCTAGCCCCAACTCACCGCAGATCCCACCAATTTCACCGTATTCTCAGATCCCGATCTCGCGTCCGATGAATCAGCAAAATTATAACCCGGTGCCTACTCATTCCCGATCGTTGTCTCAGCCTTCTTTTTTCTCTCTCGATTCTTTGCCCCCTTTAAGCCCATCTCCATTTCGTGAATCCCCAACTACATCGAATTCAGATCAGGTTTCTGCAGATACATCAATGGAGGATAGGGATAACAGTTCACATTCTTTGTTGCCTCCCTCACCTTATATGAGGGCCAATTCTTCTAAGATGGGAGATTCTTTACCTCCTCGTAAAGCACATAGGCGGTCTAGTAGTGATATTCCATTTGGATTATCTTCAATGATTCAGCCATCTCCTCTTCTCCCATTCAATAGCTCTGGTGGATTGGAACGATCAATTAGTAGTAAAGAGAATGCTGGCTTATTAAAGCCCGCCAGCCAGTTTGTTAAAAGAGAACCTAGTTTGGAGAAAAGCGTTGACAACAATTTAGAAGGAATGGGTGAGAGGAAATCTGATGGGGACAGCGTGGACGACTTGTTTTCTGCTTATATGAATTTGGATAATATCGATTTGTTCAACTCTTCTGGGACTAACGACAAGAATGGTCAAGAGAATCGAGAGGATTTGGATAGTAGGGGTAGTGGAACAAAGACAAATGGGGGTGAGAGCAGTGATAATGAAGCAGAAAGCAGTGTAAATGAAAGCGGGGATAGCGCTCAAATGCCTGGATTGAATTCATCTGCTGAGAAGAGGGAAGGGATCAAACGGACCGCAGGGGGAGATATTGCTCCAACTACCAGACATTACCGAAGTGTCTCCATGGATAGTTTCATGGGCAAGTTGCAGTTTGGTGATGAGTCTCCCAAAATGCCTCCCACACCACCTGGCGTTCGCCCGGGGCAACTTTCTTCAAACAACTTAGTTGATGGTAATTCTGCTCCATTCAGTTTGGAGTTTGGTAATGGTGAGTTCAGTGGGGCTGAACTGAAGAAAATTATGGCAAATGACAAACTTGCCGAGATTGCTTTGACTGATCCCAAGCGTGCAAAGAGGTATGTGCAAATTATGTCATTGACTGATTTACTTAAATTACTTCGTTTATTGATTTTTAATATTGAGCAATAATTTACTTCAGGATCTTGGCAAACCGTCAGTCTGCTGCTCGATCGAAAGAGCGAAAGATGCGGTATATATCTGAGTTGGAACACAAGGTTCAGACTCTTCAGACAGAAGCTACCACACTGTCCGCTCAACTCACACTTCTACAGGTAGAAATCTTGAGTATTGACAATTATTTTCTCTTTCCATTTTCCTGCAGCTAAATTCTTGGTTTTTCCATAATTGCTCTGCATTCAGAGAGATTCCGTGGGCCTTACAAACCAGAATAATGAACTGAAGTTTCGACTACAAGCCATGGAACAACAAGCACAACTACGGGATGGTATTGAAACTTCTCTTTACCTTTTTAACTCCTATATGTATCTGAAAAGATAGTCAATTAAAGCATCAACTAAAACTGTGTTAGCCATTTTTTTTCTGGAAAATGACCTTCAATATACTTATCGCATTGATTAGTTCAGATCTTTGAAAAACCCATCATGGGGTTTTGTATTTGTTGCATTGAATCTGGGTTCTTTTCACTTCCTAGGCCTTAGATATCAGATAGTACAAATTATAACTGTAGTGTCAATCTGAGTTGATCTGTAATGTTACTTGTTTTACTATTGTCGTGCCAGTTAAGAATTTTTATTAAGAAATGTTGGTACATATTAGCGGGCCCTAGGCTTCCTGGACTTCATGGCTAACTCCTACGGGACTTGCTCTTCCTTTATTGTATATTTTAACTTGAAGCCGTTAGCGATGTTGTTACTCCTTTGTTGAGCATGATAATGTAGATCGTAGTTTGTTTCAGTTGTAGAATTCACTTATACTTCGCTTTTATTTATTTTTCTTTATCAAATGTGACAGCTCTTAACGAAGCCTTAACCGCGGAGGTTCAGAGATTGAAACTCGCTACAACAGAGCTAAATGCGCAGTCTCATCCCTCCAACGGAGTAATGCCCCAATCTTCCATCAATCACCATGGCCTACAGCTTCAGCTTCAGCACCAGCAACAACAACAGATCCAGCAGAATGGGAATGCAACCACAAAACCAGAATCCAACCAATAA

>ClabZIP9

ATGGGTGATACTGAAGATGCTCGTACTGATAACCTACGAAATCTTCAATGTTCGTTTGGAACATCTTCCTCTTCTGCTTTAAAGCATCATTTCTCTATGGATCAACTTAAAATTTCTCAAATGAACTGCTCACAAGGCCGTGCACAGCATTTTCAGTCGAATTTTCTTGGAGATAATAGTAGAAGAATTGGGATACCTCCTTGTCCCAACTCACCGCAGATCCCACCAATCTCACCGTATTCCCAGATCCCGGTGTCGCGTCCGATGAACCAGCAGAGTTATAACTCAGTTCCTACTCATTCTCGATCGTTATCTCAGCCTTCTTTTTTCTCTCTCGATTCTTTGCCCCCTTTAAGTCCGTCTCCATTTCGTGACTCCCCTTCTACATCGAATTCAGATCAGGTTTCTGCTGATACGTCAATGGAGGATAGGGATGCCAGTTCACATTCTTTGTTGCCTCCCTCACCTTATACGAGAGCCAATTCTTCGAAGATGGGTGATGCTTTACCTCCTCGTAAAGCCCATAGGCGTTCTAACAGTGATATTCCATTTGGATTATCTTCGATGATTCAGTCATCTCCTCTTCTCCCTTTTAGTGGTTCAGGTGGATTGGAGCGATCAACTAGTAGTAAAGAGAATGCGGGAATATTTAAGCCGGCCAGCCAGTTTGTTAAAAGAGAGCCTAGTTTGGAGAAAAGCATTGATAACAATCTGGAAGGAATGGGTGAAAAGAAGTCTGAAGGGGACACTGTGGATGATTTATTCTCTGCTTATATGAATTTGGATAATATTGATTTGTTCAACTCCTCAGGGACCAACGACAAGAATGGTCATGAGAATCGGGAGGATTTGGATAGTAGAGGTAGCGGAACAAAAACAGGGGGTGAGAGCAGCGATAATGAAGCAGAAAGCAGTGTGAACGAAAGTGGGGATAACTCTCAAGTGCCTGGATTGAATTCGTCTGCTGAGAAGAGGGAAGGGATTAAACGGACTGCAGGGGGAGATATTGCTCCAAATAACAGACATTACCGGAGTATCTCCATGGATAGTTTTATGGGCAAGTTACAGTTTGGTGATGAGTCACCGAAAATGCCTCCTACGCCGCCCGGCATTCGTCCAGGGCAACTTTCTTCAAACAACCTAGTTGACGGTAATTCAACTCCATTCAGCTTGGAGTTCGGTAATGGTGAGTTCAGTGGGGCTGAACTGAAGAAAATTATGGCAAATGACAAACTTGCGGAGATTGCACTAACCGATCCCAAGCGTGCAAAGAGGTATGTGTAAATAATGTTATCAACTGATATTCTAAAATGGCTTCGTCCATTGGTTATTAATCTTGAGTTACTGGTTTACTTCAGGATTTTGGCTAACCGTCAATCTGCTGCTCGATCAAAAGAACGGAAAATGCGGTATATATCTGAGTTGGAACACAAGGTTCAGACTCTTCAGACAGAAGCTACCACGCTGTCTGCCCAACTCACGCTTCTGCAGGTAGATATTTTGAGTTTTGAGTGATGACAATTATTTTTATTTTCATTTTTCTGTGCCTAAATTTTGCGGTTTTACTGTGACTATATTATACTCAGCGAGACTCTGTTGGACTTACAAACCAGAATAATGAACTTAAGTTCCGTCTCCAAGCCATGGAACAGCAAGCACAACTACGGGATGGTATTGACAATTTCTCTTAA

>ClabZIP10

ATGAATTTCAGGAACTTTGAGGATATGCCACCAGGAGAAGGCACTATGGCTAAGGCACAAGGAAATTTCACATTGACCAGACAGCCTTCCATATACTCTTTAACCTTTGATGAATTTCAGAACACTTGGAATGGACTTGGAAAGGATGTTGGTTCGATGAATATGGATGAACTTCTAAAAAACATATGGACTGCCGAAGAGTCTCAGGCAATAACATCTACAGGTGCTGTTGCAGGCGGAGTTGGAAGCACCAATGCTGGGAACTTGCAGAGGCAAGGTTCATTAACTTTACCCCGGACCATTAGTCAGAAAACGGTTGATGAAGTTTGGAAAGACTTGAGTAAAGAGAATACCAGTGTTAAAGAGGGAAATGGAATTGAAGCCATGCCTCGACGACAACCGACATTAGGGGAAGTGACTTTGGAAGAATTTTTAGCTAGAGCAGGGGTGGTAAGAGAAGAACCTCCACATATTGAGGAGAGGCCATTTAACTGTGGATTTTATGGTGGATTATCAAGAGAAGACAACAATGCTGGTTTGGCTCTTGGGATGTTCATGGGAAATCAGATAGCTGAGAACAAAAGTATGGTTTCAAATCAGAATCAAAATTCAGTGTTTTTAGGGACCGGAGTTGTAAGGTCTTCTCAGCAGCAGCAGCAACAGCAGCCACTTTTTCCCAAACCAGCTAATGTGACTTTTGCTTCTTCAATGAATTTAGTGAACAATCCCCAGCTTACTAATGGATCTGGGACTAATTTGGTTGTGGCCCCAAAACCTCCTTTACATGAGGCTTTAATTCAGGGCAGTGGCATCGGTGCAATCGGTTTAGGCACGAGAGGTGTAACTGTTGCTTCAAGATCTCCTACAAGTACCATATCATCTGATGTGATTACAAAAAGCAGCATAGAGACGTCTTCATTTTCACCCGTTCCATTTTCATTTGGCCGGGGAAGGCGAAGCAGCGGAGCTCTTGAGAAAGTTGTTGAGAGGAGGCAACGAAGAATGATAAAGAACAGAGAGTCAGCTGCAAGATCACGGGCTCGTAAACAGGTAGCTATGCTAATGGTCATCATAGTACTGATGCACTATTTAACTGTGAAATGCTCTTTCCTATTTGCCTTGGTTTTTCAAAGTAATATTCATTTGTTGTACACTTTCTTAGGACTTGTTTTAAAGTAGTGAGCCACTCAAAAAGCTTCTAACCCAATGAAATTCTTGTGTTTTACTCCATTTACCTTCTTGTTTTTTTCTTTTTTTTTTTTCTTCTTTTCTTTCTTTCTTTCTTTTTTTCTTCCATCATTATCATTCATTCATTCATTCATGGAGGTCACTTACCTACAATTTAATATCCTACGAGTTTCCTTGATACCCAAATGTTGTAGGGTTAGGCGGGTTGTCCTGTGAGATTAATCGAGGTGCCCATAAGCGGGCCTGGACACTCACTCCATTTAGCTTCTTGAAACTTCTAGCAAAAATATTATGGATGGATTCCTTGTATCATACCTGTAAAACTCGCAGTTTCAGTTCAAAGATGGTAGATTCCATAACATTTTATTGTTATGATCTCTGTGATATCTTCCATCTCAAATGCCATGCAAAGATAAAGATTCACACTTATGCTGAAATGATTTCTCTGGGGACCTTTCTTCAGTAGGGAAAATTCTAAATATGACAGGAGAAACAGGCTGAGGCTGTCATAAACACAAGAAAAACATAGGGACCTTTATAACTACTATTTCCCTTGCCAGAATAAAAACAACTAAAAGAATCCAGAAGAGAACCAAACTATATTGAAATATAAAAGATAAACTTACAAGAGAAACTAACTAATTTGAGAAACTCGGACCTTTCCTTTCCTTAGATTGCTCAAGCTCTAAACCAACTAAATCAATCTCTAACTTTCTCCTTCTCCAATTTTGCTATTTATAATAAACTAGCGTGAACACTTCTCTAGCCAATTTCGAATACACGTTTAAAATTCTAATAATATTCCTAGTCTTATCCTAAAACCATTCCCATCAACAACTTTTTCCCTGTGATTCACAAAATATGTAAACCACATGATTGTAAACCATGCAAGTAGTATTATCAGTTAAGTTCTAATGTTATTTTCTTTGGAATTGGTATGCAAGGCCATTTTACGTCTTAGTATATAATCTGTTACCAATCGTAGATGTCAAAAAATGCATACAAATTTTGGAAGAAAGAAGTATTTTTTTCCAAATCTGCAGTTTATTCTCAAGAGAGAGTCTAAAAACATTCTTTGATGCTGCCAACATTTCCAGCCAATCACATAAAACAACTTTGAAAATGGAACTGTTGCTAAAGTATCTTTAAAATATCGATCAAAATAGGACTGGAGAATCCATTTTGATAATTTAAACTCTGCTTTACAAGTTTTAGATTCCTCCACGATAACGGTTCAATGATATTTCAGGCTTATACCTTAGAACTTGAAGCAGAAATTGCAAAACTCAAAGAAATGAATCAAGAGTTGCAGAAGAAACAGGTATATTACATGTCAAAGAATGATTTTATTTGGTATTATTTGGATAAAGAATCATCTTGATCTCCATTTATGCATCTTATCATCTGCAGAGAGAGATAATGGAAACGCAGAAAAATCAGGTACATGTTTCTCTGATCTGTCTCAAACTGCCAACTCATTTGTTTTAAAACTCTGCTTCTTTAGAAACACAGAAGCTTTCATTTTAACAACCCTTTGCTTATCCTTTTCTTTTGACAGGTTTTGGAGAAGATGAAATATCAGTTGGGTGGCAAAAGACTCTGCTTAAGAAGAACACTGACTGGCCCTTGGTAG

>ClabZIP11

ATGGAAGACGGGGAGCTTGAATCCTCGAACCCTGAAGTGTTTTCGAGTTCGAATGCTGTTGAGCTTCCGAGCAGTTGCTCAATGGATAGCTTCTTTGATGAAATTCTGAAGGACACTCATGCTTGCACACATGCTCATACTTGCAACCCTCCTGGTCCTGATTATTCACATACACACACCTGTTTTCATGTCCATACGAAAATTGTCTCTTCCCCCACTGAAGAAAAGGTTTCCACTGACGACACAGCGGAGTCTGTGGACAAAAAGAACAAGAAGCGGCCGCTTGGTAATCGTGAAGCAGTTCGTAAATACCGGGAGAAGAAAAAGGCGAGGGCTGCATCATTGGAAGATGAAGTTGTGAGATTGAGGGCCCTGAATCAGCAACTGTTGAAGAGATTGCAAGGTCAGGCTGCATTGGAGGCTGAGATTTCCAGGCTCAAATGTTTGCTTGTGGATATTCGGGGAAGAATTGAAGGGGAGATTGGAACGTTTCCATATCGGAAACCAGCCAATTCAGATCTGCCCAATCAAAATGTGCCGGGTAGTTACATGGTGAATCCATGTAATGTGCAATGCAATGATCAAGCATATTGCCTTCACCCAGGGGACGATGGAAAAAGTGGAGAGAGCGTTTCGTTGAATGGGCAAAGTTTTAGTGCGTGTGACTTTGAAAATCTTCAGTGCTTGGCAAATCAAAACACGGGAGTAAAGGAACTTCCTGACTGTGGACTAGGAAACACAATTTCTAACGTTAATTGCTCAGAATTAAATCCGAAGAAAGGTAAAACCAGAGCACTGAACTTTTAA

>ClabZIP12

ATGGATAGGGTGTTTCCAGTGGGTGAAATTTCTGACCACTACTGGTCGTCGGAACCTGCCGCCGGTCCTCCGCCCCCACCGGCCGACGAAGGCTCCAAAATGAACCGTAGCGCATCGGAGTGGGCCTTTCAGCGCTTCCTTCAAGAAGCCTCTGAGACTTCCCCTCATTCCTCTGCTGCAGACCATGGGGAAGTCGTCGAGATCAAGGACTCTGCCTATAATCAATTACAGAAACTTAATGCTAATCAAGTTGGTGTTAGTAATTGTAATACCAGCAGCATTTCTTCCAATGCTGTGCCGCCGAATATTCCGATCGATTCTGAGGAATATCAGGCCTTCCTTAAGAGCAAGCTTCATTTGGCTTGTGCTGCTGTCGCCATGAAGAGGGTAAACTTTATCCTCTATTTTTCTTCTTCTTCTTCTTCTTCTTCTTCTTCTTTTCTTGTTATTGATGGGGATTGTTGATTTCTTTTCTGTGTTTTGTTGATGAGGGAATTTTCAGAGAGTGAACAATATATAGGAAATCTGAACGATGATTTCTGACATTGTGGAAGTGTTTTTGGTTGTTTTATCCGTTCACTTATGTATTGATTCACTACGGGACTGCTTGGTCGAAACTCAACTTCTTTAATTTCTTCTTTTTCCTTTTCTCAATCGGTTATATTCACATTTTTAGTTTTTGTGGAGGAAGGGAGTTAGCTCTAACGTACTGGATTGCTGGGTTAAGATTAGGAGTTGGGGGTTTTTCGTTAATCATCTTGTGGGGTGTTGTAGATATAATTAGTATGATCAAAGCGTTTTCTTATATTTCTTATGTGAATTTCAACTTCTTCATATATTAAAACGAGATATTAACCCTTCTAGAAGCTCCAGCCTCTACGGTACTAGCATGGGAGGCTTGTCTTTGAAGTAAATTTTCAGATGAATTACGGACTACAGCACTCACCTTCTTTGCTTTTGGTGAGGAAAAGGCCAATCGTGACTTAAAAATGGTGTGTCTCACGCTTTGTGGTGCCTTTCATGATATCCAAAATTTTCATCCCATTGTAGTTAAACATGGAGTTTACAACTTTACTGCATTCGCCTTAGCGATAGGAGGTTGTATGACAAGCTGAAAACAATATGAAGGGGAGAGCAGCTGCTGTGATCTTTACAAGTTTTGGAAACTAGGAAGGGAATAATTTTTAATGATGTTAGGAGGAGATGAGGAAGAGTTTTGAGATATTTGATAACCATAGATATTTATTTTAATTTATGTGTGCCTTCACTAATACAAATGAAAAACTGCAGGACCTTACAAGGCCCTTATAGGATGTTTAATATTTCTTTTGGGTAGGTGCTACCATAGATTGTATCTACGACCTTCATGACTTCTAGATCTCCAAGTTACTACTTAGGGCCACTTGAGGCTTATGATTTATTAGCTGAATGCTTTCTTTGGACGCACTATTCCAATGTCCTCTTATACCTGTACACTTCTAGCCTGACTCCTAGTCATTGTTATGCTAATTTCATACCCTTTTCTAAGAGACAAAGCAAGCTTCAGTAGAAGATGGTGATAGTTAAAACAAAACAATGACAAAGGGACAAGACATCCCTTGTAATAGTTTAATTCAATTGTTTCTTCTATCACCAAAGGAAAAGAAAGGAAGACGGAAAACAAGTATTATTAACCATTGGACAGTTGAAAAATACCCTGTCTGAGGAAGTAATATATTATCAACTGCTGGAAACTAGAAAACTTATCTTTTTCTTTTACTTATTTATTCTGTCTTGTGTATGTGATCTACAATCGTCAAATCCTTGCTAGATCTAGGATGAAGTAGAAGCCTTCCATTCATATTAATTGATCTGGAGAGTACCCATGCCTCTTTCAAGGTAAGGGTGGTGACATGTTGTTTTCTTATAAGCAGGGATCCTTTAGAATGACTCCAGCTTCGTCCACCTCAGCTGACTGCGGATCACAAGCATCTAATACGTTGGGAATTCAAGCTCCTAAAGGTTAGGATTACGACATAAATATTTTTTGTTTGTCACAAATAATATGGGATGTAGTTAGATTTTTTGCCTTTATTTATGTAGTTTATATGTACTGTCTCTTCTTTCTCTCTCATCATGTGGATGGTTAGTTAAACGAGCTGCTGAATTTAGATTTTAATTTTTATCTATATCCTTCTCTTCCATAGCTTCTAATATAGGAGCTGGGAATAATTCATTAAGGTCACCAGATAAAGATATAAATGGGGCCGCTGGAGTTACTTCTTCATCCGTGGTGCCAAAAATACCTGAGCTTCGAGCTCGGCCAGCTACTAGTGGATCATCAAGAGATCTGTCGGATGATGAGGAGATTGAGGGAGACACTGAAACAAATGAAAGCAAGGACCCTGCTGATGTAAAACGTGTAAGGAGGTAACAATCTGCTAAGATCTAATTTGGGGATTGCAGAGTTTTTAAGTCTGTCCTCACTTTTTGTGTTTATTGACAATCTTTATGGTGGAGGTTTCGTATAAATAACAAACCAGTTTTATTGCAAATGCATTGTGTGAATTCTGATGGTACAAATAAAATTTAATTCATATCTGATTGGACTTTCTATAGTTTCTTCCGCTGTCCTAGATGATTTGTTCTTTTGGATTTAGAAATTTCTCATTAAGCAGAATCTATTTTGGCTTTTGCATGCTTTAAAATTATCCATGTTTATTAAGTCGTTTGTACATGTTATGGGACTTAAAAAGGGGTGGTCTTTGCAATGAAGTGGTTGTTTTAGTTGCATGGGAATTGCAATTGAGATTAGATCATAGTGCAAATTAGTCATGGAGTTTTTGTTCCTTCTTTATCTTAATAACACTACAAAAATTTTATTTCCTATTAATTGTTCTTCACATCGTTTTCAGAATGCTTTCAAACAGAGAGTCAGCTCGACGATCAAGGAGAAGAAAACAAGCACATTTAACTGAACTTGAGACACAGGTGTTTATTATTAGCTCGTACCAACATTGTCCAATGCCTTTATACGTCTTTTCTAATCTGCTGAACTGATGCATTGATTCATTTTCTTTGCATAAACCAAGGTTGCCCAATTAAGAGTTGAAAACTCTACCTTGTTGAAGCGCCTTGCCGATATAAGTCAAAAGTACAATGAAGCCAATGTTGATAATCGGGTTCTAAAAGCTAATGTTGAGACATTAAGAGCAAAGGTACAACGACAATCTATTCATTCCCAGTTTTCCTAATGCGTCTGAAATTTTCTTGCTCACAAGCTTTGTTAATAAGTGCAATATACTTTTCCTCTCATTCCGGAGACGAAAAATGCTCAATACGTAAAGTTACCACTTGTTCAAAGATGTAATCTATGTTGAGCACTAAATGCAACTAATATTATTTCCATCTTGGCAGGTAAAGATGGCTGAAGAGACTGTCAAGCGAGTTACTGGTAACCCAATGTTCCATGCAATGTCTGAGATCTCCTCCATTGGCATCTCTTCATTCGAGGGTAGCCCTTCCGATACATCAACAGATGCTGCCGTTCCTTTGCAAGATGATCCATGTCGTCATCTATATCAATCAACATCTAATAACCCCATGGGCCCACACGACATAGTAGTCAATAATGGATTGGCTAACATTTCGCAGGTGGGGAGTGGGCAGCAGAATTCTCCATCTCAGGTACTGCCAGCCACATCGGGGAACAAGACCGGAAGATCGGAGTCTCTGCAGAGAGTGGCTAGCTTGGAGCATTTGCAAAAGCGGATCTGTGGAGTCAAAGCAAATGCAGAACAGTAG

>ClabZIP13

ATGCAAGATCCAGCGCCATCAAACCCTATCCACACTCCCAATTCCAATCAAATTCCTCCACTCAATGTTGCAGCACCGCCGCCGAAGACTCACAAACCGCCGCCGGTGGCGAATGCGACGGCCTCCTCTTCATCAATGTTTGCCAATGCGAATACTGGCAATACCTCATTTATGCCTAGAGTAGGCTCTCATCATCGGAGAGCTCATTCAGAAGTTAGTTTCCGGTTGCCGGAGGATATGATGGATATATCCGCGTCGGATCCGTTCAATGGCGGTTCATGACTATATCCGCGGCGGATCCGTTCAATGGCGGTTCGTCCACCGCAAGTCTGGAGGAGATCGGATCGGAAGATGATCTGTTTTCGACCTACATTGATGTGAAGAAGCTTGGAGGTAATGGAGGAGGGAATTTTGTAGATCATAATGGTAATGGAGGAAGTGAAGGTGCTGGTGGTAGTGAAGGGGAGAAGACTTCGAAGCCAAGGCATCGGCATAGTGTTTCGGTGGATGGAACGACGTCGTCGTCGAGTATGTTTGGGGAAATTATGGAAGCGAAGAAAGCTATGCCTCCTGATAAGTTGGCGGAGCTTTGGTCCAGTGATCCCAAACGCGCTAAAAGGTTTTTTTTCTTTCCATTTCTATTTTGATTTTCTGTCTCTGTTTTCTTCTGCGTAATTCTTTTGCTTTTCGATCCATTTTTTGTTCGATTTTTCCATTATGTCTGTCTCGCCTGCTAGATTATCGCCATTTTCAATGCTCTTGAGTTTTTATTTTCAATAATCTTCAGTCGCCAATCACAGAAGTTTTCAGCATTTCTGTTTAGATTCTGAAATGAGATACCAAGCTAATTATACTTCATAATTTTAGCTGATTCAACTTCAGTCCTTGCTCCACATTGGTTTTCTTCATTTTCAAAAACTGGGCTTGGTTAAATTTTAGTATCAAGTTGAGGGGTAGAGGGATCTGATTCCGTTTATCTATTCTTTTTTCATTTGAACTTTCGATTACATTCCCAGATCCAAGCGTAATTTGTTTTCAAATTTTTATTATAGTGTGTAAGTCAGTCGACGATCTCCCCTCTGTTGCTGGATTTATTTCTTGATTCCAGAATTTGGGGGTTTTATGAAGCTTGCTGCTACATGACATTTAACAGAAATAATCATTTACTTAAGTTGAGTAAAGCTATAGTTAGAATCAAATTATGCAGCTTTTAAAACGTAACCATGCACCTCCATCCCCGTATTTGTTCATGTATCGTTTTAAAGTATGGTATCTCGTTGGGTTATTAGTTAGATCTTTTGGTTAGTTCAACAACATGCGGGTTTGGAGATTCAAACGAGTAAGAGTTCAAGTCTTAACCAGAAAAACCGACCAAACCAGCCAAAACCACCGTTGGTTCAGCATCTGTTTGGATGTGGTTTCCCAAGTCTGGAAGCCGACCAAATCGGTTCATCAGTTGGCATGTCAGTTTCCACCCCGAACCGACCGAAAATCCAAAGTTTCCTTCTTTTTTTTTTTTTTTTTTTTTCCCGATTTGCAAATTGTTTTCTGTCTGCCGTGTGGCATCAAGCTATATTTAGTCAACTTACTATATTTTGTCCGATTTTCAAGAAGTGAACAATTGACATTAGGTTATCATTTGGGAATGAATTAATGATAAAGACGAATTCCTTATTGTATTAAACAACTAATTTGTTTCTTTTCTCTTGTGCCATCAATATACAAACAATTTGGAGAGGAATAGGTGGGAAAGATGATGAATTCATTGTATTGTATGATTTGCTTCATCTCGATATGTATCGATATACATAATGATTTCAGCTTTCCTGGGAAACCTTCTCCTCATTGCTGTTCTGTTTAGGATATTGGCAAATCGACAGTCTGCTGCTCGCTCAAAAGAGAGGAAGGCAAGATATATACAAGAATTGGAGCGCAAAGTGCAAACCCTTCAAACAGAAGCAACTACTCTATCAGCCCAATTGACTTTGTTCCAGGTTTGTGTCTATCAATATAAACCTCAGCTTCCATTTTTCCCAGAACTTCCTCTCTCTCTCTGACTTGATGTCTTGGATGGAATGCATTCCAATTGTGGCTAGGGATACATAAAGTGATACATAAAGTCACTGCGATAATATGCTTGAAACTTATGTTCTTGGTTGAAAAAAAGGGGCTGCCATTTTATCATAGATTTTGAATTTTGTCGTCTAGACTAAAATACTATTTTAGTCCAAGTCCACTTTAGTCGTACTTTCAATAAATCGAAATCTTAAATTTAGTTCCACTGAGGAGGGAATATACTTATAAAGAAAATGTTAATAGAAACTTTTATTGAAAGAATCAACCAAAGGATAAAAAATAAAAAACAAACTCCATGGGTTACTTTTAAGATTGAGTAATATTTGGGTACAGTTGAGTTGCAACAATCTCCCTGCATTCTTTCCAATTGCAAAGAGAAAAAGTTCTAAAAAATAATCAATAAGAAAAGTTTGCCATATGGCAAGTTGAGATTTATTGAATGTCTTGGACTATTCTTAGGTACATGAATCAAAATGGAATGAAACTCAAAATTTAGGTGTCAAAACGGTATTTTAATCATTAAAATATTATTTCACTAACTTGACCATCAACTATATATTATATGTAATAATAAGGATGGGTCCTCATATTCTCGTTAGCTAGAAAACAACTTAGAACTATGATTTGATCGGAACTTATCATCTTATACAGCGAGACACAACGGGTCTCAGTACTGAAAATACAGAGCTTAAACTTCGGTTACAGGCTATGGAGCAACAAGCCCAATTGCGTGATGGTGAGAATTATACTTCATTACTTAATGTGTAATTGTAAAGCTCGAAAGCTTTTGAACATATTATTGATTTCTCAATCTTGTTTATGCAGCTTTGAACGAAGCATTAAAGAAGGAAGTTGAGAGGCTTAAAATCGCTACTGGGGAAATGATGAGCCCATCTGAGTCTTTTAACTTAGGAATGCATCACATGGCGTACGCCCCGTCATCTTTCATTCAACTTTCACAGCAACAACCAGGATCTGTTGGCCTTCAAAATATGCAAATGCCACCATATAGCCATTCTCCATCCAATATGACTACTCACCCTCTGCTTCCATCAGAGTCTCATTCACTCTCTGAGGTTCTGCAGACCGACTCTCTTGGCCGATTGCAGGGTCTCGACATCAGTAGTAAAGGATCGTCGCTTGTGAAATCCGAGGGTCCTTCACTCTCTGCTAGTGAAAGCAGTACAACCTTTTGA

>ClabZIP60

ATGGAGGAAAGTTACAAAATGAGGATGAACCAAGGCGGAGGAATTCCGACGATGGCAGCAGCACTGCCACCATTGCCACCGTCATGTTTAGGAAAACTGACAACTAGCGGTGAAAAAAAGCTACCGTTCTTTCAATCCAACATGAATTTAAGTATGTATGGAAATGACAAAAGTATCCTTTCTCAAAGAGAAGCTACCATAACACCACCGCCGAAGCAACACCAATCACAACTTCTAGACTCCGACAAAGATCTCACTGTCGAAGCCAAGCGACTAAGAAGGTTCACTCTCTTGAAACTAATCTAGAACTTAAAAGTAAAATTTAAAAAGTCATTTCAAACAACCCTAACGTTCTTTTATTCATGTACGTACGTAAATACGTACATAAATGTTTTTTTTTTTTTTTTTACATGGGTTAATTTTTTATTTTTTACTATAGGGTGATGCAAAGCAGACAATACTCTCAAAAGTATCGACTAAAACAGCTTCATTATATTACTCAGCTTGAATCAGAACTAAAAGCCCTTCAAGTAATTACATAAATCAATAAATAATATCAATCAATTAATTAATTAATTAAATTAGTTGATTAATCACCTTTTTTGGTTGAACGAATTAAAACAGGCAGAAGTAACAATTACCACACCGAGGATAAAATTCATGGACCGTCAAAATTCACTGCTCCGAGCCGAAAATTACTCCATCAAAGAGAAATTATCTGCATACACCGGAGAACTTCTATTCAAAGAAGGTAAATTAAATTAAAATTTTTATTTTATTTTTAAAAATTTTAATATGATATTTTTGCATGGGGCAGCTCAATACGAAGAATTGAAAAGAGAGAGAAATATGCTTAAGGAAATCTACGAAGCATATCAGTTAAAATTGCTGGAGACTCTGAAAAGCAGCAACAACAACAACAACACAACTGCTGCCAGTGGAAGCACTTTTCAATTGGTCGAAAATTACCCACAAATTGCTACCAAATCAAACCCATTTACCATGCTCGAAAATTAA

>ClabZIP14

ATGGGGAGCCAAAGAGTTGAAGAAACTGGTCCTTCATCCCACTATCATCATCACCTTCCATATAATTCACTTCTTTATGGAATCAACAACAATAATCTTTCCTCCACTTTGATGTAATTTTTTTAATCAAAAACTTTTTTTTTTTTTTTTTTATCTGTTCTGTTTCTTTTCATGAATGTGTCAATTTTAAAACTGTCATTTTGATTACTTTTGTCTCTCAGCAATCACGATGGACAATCTTTTGATTTTGGAGAATTGGAACAAGCAATTGTGCTACAAGGACTCGGAACCAAGAACAAACTCGATCATCACGAACCTAAACAAAGTATGTATTCTTATAAATTATATTATCTCATGTAGAACTATCAGTTACATTGTTTTAAGTCCGTCCAAAGAATTAATGACATAGGACAGAGAATAAGAATTATAACCAACAAGAGAGAGAAGAGAGTTTGTGGAGCTGTGGGGGCTATTTCTGTTTTGATGACAATAATAATTTGTAAGAGGAGAGTTGAATGGCATGAGGGTACAAAGAAGGAAACTTGTAATCTGCTCACTGACTTCTCAATAACTGCAAACTAAACCCAAAATAAATAAATAAAATAAATTAAGAATAGGGTTTCTTCTACTTTAGATACTTTGAAAAATCCTTCAGTGTTTTAATTTTTTAAAATAATTTTCTTTTTAAATTGGGTGATTCTGTGAGATTTGCTTTTATGATTCTTGCAGGCTTTTTCTCAGGCAAGCCTGCAGCAACTCTGGAAATGTTTCCTTCTTGGCCAATCAAACACCAACAAACTTCAAGAGTAAATTTTTTTCTTTCCCCCCAAACTTTAATTTCTTCATTTTGAAAGGAAAAAAATGGTGGGGTTTGTTTATTTTATAATTTTTTTTTTTTTTTAAATGTGACAGGGAATTAGTTTGAGAAGAAGAGAAGAGAGCAGTGATGAGTCAGGAGCAGCTGTGAACACTCATACAATAAACAAATCTAATAATCAAGAAGGGCATTTGGAATTGGAAATAGAAAAAGAAGAATCTGAGATGAGTAAAAAGGGTTGTTGTTCATCTTCTTCTTCAGGTCAAGATCAGAGCTTTATTCAAAAGCATCTACAGCAGATTCAACCTGAAATGATACTCAGTGATATTTCAACAACTGCACTTTCATCTCAACATCAATCCCTTCAACAAAAGGTTCTCTTTCTTCTCAATAATTTTTTTTCTTTAAAAAAAAAAATATGAAAGTTTGTATCTTTTCTACTTTTTTTCAAATAATGTGGCTAACAATTTACATTGTTTGGTAAAAAATTGTACTTGGATAAGCTGCACTCATGTTTTTTTTATTTTTTTCGTCTGGACAAAAAGGGTCCATTTAATTTATTTTTATATTATGTGTGTATATATATATTTGTATTAGGGCACTGATCCCCAGAACTATTAGAAAATCAAATAAAAGGACAGAGTTTTTCAGATTTTTTAAAATTTTTTATTTATATGATGGAATGTCTGACATGAAAAAGATAGAAGGAAGAGAGAGCTGCAGTACCATCAAAAAGCCAATGAGAGGAATCTTCACAGTGTTTTTTTTTTTTTTTTTTTTTTTTTTTTTTAATAATTATAAAATGACTAACTTTTTACTAAAAACCCAGAGAGCAAAACATGAAGATCTTTCTTTGTCGGTTATTCAAAAGAAAAAACGTAAGTAAAATTGAAATAATTAGTTTTGTAATAGAACAATGAATCAGATAATGATGTTTTCTCGCTAAATACATTTTCATTTTGTCGACATGTTTTTAGCTATGTTTGGCATATAGTAGGTGCAATTTTGATAATTTATAATATTAATTTAATTTTATGATATTATTATCAATAAAATTCACCAACCACTTAAGAGAGTTTTATTCATATATTTTTAAATGTGACCAATAATGCAGAGAAAAGGATGTGGTTCCATTTCAACATCACAGAAACAACTTGACGCTAAGGTCTTTTAATTATTATAAACAAACATTGTTAATGTTCTTGTCTTGTTTTTTTTTTTTTTTTTTTTTTTTTTGTATAAATAATTTGAATTTGTGCTTAGACATTGAGACGTTTAGCTCAGAACAGAGAGGCTGCGAGGAAGAGTCGACTAAGGAAAAAGGTTATAATTTTTAGGTTTGACATTTTTTTTTTGTTGCCATTATTTGATATTATTTTTAATGTAAAGAGTAATTAATGAGATTTGAATTATGGTGATTAATTATATATAGGCTTACGTACAGCAACTCGAGAGCAGTAGAATCAAGCTTACGCAGCTTGAGCAGGACCTTCAACGGGCACGATCTCAGGTTCCAAATCTTTTTTCTTTTTTACACTCCAAATTATATATACTCCTAAAATAAATCAATTTAATTTCAAATATAAAATAATGAACCAAAATATTTATTAATATAACAAAATTTCATTATCTATCTAGACAGATTGTCATAGACTATTATATGTGTCATATCGTGATCTATTATGATCTATCATAAATCGACAATGATATTTTGATATTATCTACAAATATTTTCAGTAGTTTTGTCATTTAAAATAAATTTTCAATAATCTATAAAAACTCTATTTATTCTTTTTCTTTTTTTTAGAAAAATATATTTTTTTATCTCTAAAATTTAGATTTAGTTTTCACCTTTTACTTCTTTTACTTATTTAATTTTTATTTGGACGCAAAGTTTCTAAATTTTATCTTTAAGACTTTAAGGTTTAATTTTTAATCTTGATTTTGTCTACTATATACTTACATACGGTTCTTCATATCCGTTAATGCAGGTTAATTGAGTTAATAGATTTAAATTAATTATAATGAAATAATTTTTTCAATTTTCCATTACTATTAAAAATTAATTAAAATTTTCCCATCATAAAGGTTTTAAATTAATTAATATAAAATTAATTTTCACTAATTTTTCATTACTATAAAATATTTAATAACAATTTTGATATCATAATTGTTTTAAATTAATTAACTAAAAGTTAACGTTTAATATCATAGTAAACATCATTAAAAACTCAAGGATAAATATATAAAATATCAAAATTTAATCATTTAATGGTTAAATAAAAATTAAATTCAAAATTTGAGAATGTAAAAATGTAATATTTTAAGAGGTACAGATCAAATAAAAATTAAACTATAAAAAGTACATCTTCAAATTTAAGAATATCAAGGTATCAATAGTATTTTTTCTTCTTTTTAATAAATAAATTAATAGACTTAATATATATATCTCTTTTAAGTAACAAAAAAAATTGTTGTTTTCCTTTTTCTTATAGGGTTTGTTTTTAGGTGCCGGCGGTACTGGCGGCGGCATTGTCAGCCCCGGTAAGCTTCAAGCATCTGCTCAAATAATCAAATTTCATCTATAAATTTTTTCATGATTAATAAAGAAAAAAAAATACGCACAAAATTAGACCATAACACTTACTACTCATATTCATTGTTTGATGTAAAAAAAAATTTCATAAAGATTTATTTTATTTTTTACTTCAAAAAATATAGTTGTTAGATTTATGTAAATGACTAATAAGTAATAAGACCTATGTCCACTGTAAAAATAGCAATAGCTTAAAAACTATTTAGATATAATACATCGAAATGGTTACCATTCTTTATGTAAAATGCTAAACTAAACATAATTTTAGATCTTATACAAAATCAAAAGTTGAATAAATGGCTATGACTTTATAATTATATGGAGAGGTTTTGAAAAATGGTAGCAAGTATTGGGAAAAAGAAAAATCTCTCACAATAACAGGAAAAAAAATAAAATTTCACCATGAAAAAAATTATTACAATCATATAGAATAATTCTCTCTCCCGCTTTTATACAAAAAGAGAATTTTAACTATAGTTATCATACGTAAACTTAAAAGGTATTTTTTTAAATTAGGGGTATAAACTCTTTCTTCATATTTTTCTAATGTAAGACAAACTGCAATTCTAATATTTTGTCAAAATTCGACAGCTAGTGCAATTTTTGATATGGAATATGTAAGGTGGTTGGAAGAGGACCATCGACATACAGTGGAGCTGCGTGGGGGACTAGAGGCACATCTGCCTGACGTGGATTTGAGAGTGAGAGTAGATGCATGCATTTGCCATTACAACGAAATTTTTCGACTCAAAGGAGAGGCTGCTAAGTCCGACATCTTCCATCTCATCACCGGAATCTGGATGTCTCCGGCGGAGCGTTGTTTCCTCTGGATCGGTGGATTTCGACCCTCCGATCTCATCAAGGTTTGCAATTTTTTTTTTAGAGAAAGATATATATATATATATATAGATGAAAATGTACTTGAGTTTAATTGGAATTTGTTGATTGATGAACAGATGTTAATGTCACAATTGGATCCAATAACAGAACAACAAGTGATGGAGATTTACAAGCTACAACATTCGTCACAACAAGCTGAAGATGCTTTATCTCAAGGACTTGATCAGCTCCATCGGTCTTTGACCGACATCATCGCCGGCGGTCCGATCATCGACGGCATCAACCACATGGTTTTGGCCATGGACAAGCTCTCTAGTCTCCAAGGCTTTCTCCATCAGGTAATTATTACTTTCTTTTGA

>ClabZIP15

ATGGAACTGTGTTCGGTGCCTGTTTCTACTTATCCGCCGATTACTGTCGGTCCCGACAACCGTTGTTTAGTCAACGATTCGGTGCGGACTACTGTCAGTCCCGACAACCGTCGTTTACTCAAGGATTCGGTTACGGACTGGCGGAGATCCTTTGTGGTTGGCTGCCGGAGGGAAGATGAAGAAGGGGGGAGGGCAGTGCAGCTGCCTCCTTTCAGCAAGATTGGCGGCGGAGAGATGAACTCTGGCGGTGACGGTGCCGAGAATGTCGACGGCAGAAATCCCGAGCAGATCTTGTATGGTCGTAATATTGAGCCTAATATGGATCCAAGGAAACTAAAACGGTAAACCCTAAAAGCTATAATTCTTTTAATTTTTCATTCATTATAATTTAATTTGTCAGAGCAAGAAATAATTTCATAAAGGATGGAAATTTTGAAGTTTTGATTTTTTATATAAATATTTCTAATTTAAAATTTTTTAAGGAACAATTGAGGAAGAAGAGTTGAAAATCATAGTTTGTTTGTGTATTGTTTTTTTAAAATCTAAGATGAATGAATTAGAAGGAAGAGAGAAATTTTTGTGTAGAGTTCCTTTGTTTGTTACACTGATCTGTCCTTTATAATTTGCTTTCTCTTTTAATTTAGGGTTTGGTTTTTGTTGTAGAATAATGTCGAATCGAGTCTCAGCTCAAAAATCTAGACTTAAAAAAGTTCAATATGTAGCAGATATGGAAAGGAAGCTGAAAGCTCTAGAGGTATTCATCATCTTCTTCTTCATTTTTTTTTTTCTTTTTAATCAAATTTTTAAATTAAAATATGATTTAAAAACTCGACTCAGTTGGATAATTATTTGGATTTTTAAAAATTAAACTTATGAACAATATTTTTAATTATAAGTTTTTTTTTTCACTTCCTTCCCATGTTTTAAAAATTTAAGCAAATTTTAGAAACTAAAAAACATCCATTTTCTAATTTTTTTTGTTTTTTTTTTTAATGAATTTGACTCAGATGGTAAGTGTTTATAAAAAATAAAAATAAAAAGTAAAAATTGTAGTATGAGATTTAGGAGTAAACAAACATAAGTTTTAGAAGAAAAAATGAAAACTTTATCAAACATAGCTTTGGTTTTTTTTGTTATGAAAATTATGCTTATAAACACTAATTTCAGCTACAAGTTTCAATGTTTTATATTATCCACACTTCATATCAATATTTTCAAAAACATCACCAAAATTTGAAAATTAAAAAAGGTATTTCAAAATTTTGTTTTTTTTTTTTTCTTCATAAATTTGATTAGGAATTCAAGTGATTATTTTATCGAGATCAAATAATGATTGAAAAAATTAGGAAGAGACAAATTTCAAAAACAGAAAACCAAGGTCTTGTTTGGTAACCATATTTGATTTTAGTTTTTTGTTTTTTAAAATTAAGCTTATAAACACCACTTTCATCTATTATTTTTTATGTTTGGTTGTCTACATTTTTTGAGTGTTTTCAAAATCAAAGCCAATTTTGGAAACTAAGAAAAAAAGTAGTTTTCAAAAACTTGTTTTTGTTTTTGAAAATTGGCTAAAAATTCTAATTTCTACATAGGAAATATGACAACCACGAGTAGGAAATTGTCAGTAAAGGAGTACAATTATCAAAAACCAAAACCAAAATATTAAATGATTACCAACCAGTGTCTAAAAACATAAAATCGATATCAAACAATCGTGATATTGTGTTTATTAGTTTATGGAAGTGTTGATGTAAAAAATTATTTAGAGATAAATTTATGAAAATACATAAATCAAAAGGATGAAAAAAAAATAATGTTTTGATTTGATGAACGAAAAAGTTACTTTTAACGCTTTATAAAACACGTGATACATTCATCATTACAAGTTAATGTCAGTTGAGCTATAGCTATATTTTAGTTGATTTCTATATATTAAAAGTTTAGATATATGCTCTATCGTTTTGTTTACCATTAAAATGTTATTGTTTCTATTTGTAGACACTTTGTATGAATCAATCTTAATCGTTACACCATTAAATATCTAGTATATGTATATAAAAACAAATATTATGGGTCTTGAATTATGTATTTGCATCAGATCTCTTGTAATATTAAGTTTTGGTTCATAAACTTAAAGATATGGTAGAATATATAGCAACCACTACTAATTAATTAGATTTTATGAAAGTTAGATCAAAACAAGATACATGAATCAAAATAAAGTAAAATTAAAGATTGATAGATCTACATAGAAAATTTCTTTAAACCTAATTCTAAATCTATTTAATACTAGTATTGTTTTTATTAAGACTATTTTACATATCTATTGAAAATGTATTTTTAAGTAAAACACTTATTCTATTAGCACTTTTAGAAGAAAATTAGTTAAGTGTGTTTTATATTTTTCTTTAAAATTTTTAAACCCCTCTGATTATAATAAAATTAACAATTAATATCGCTTTTAATTAGTTAATAAAAACACTTTATATATACCACTCATTATAACTTTAACCAAACAATATCCAACCTCTTTGAAAAACCTTTATATGTTATAATCATTTATAAATACTTTAAAATACATATGGATATTATAGCAACACAAATTTGTATTTTGTTTGATAATCTCAAAACTATTCAAGAAAATCCTAAACATACTAAAAAAAGCACCAAAGAAATGGTCTCAAGTTAATAGGCAGGCCAAATACATTGAAAATACAAATTTGTATAGCCAATTGATTTGATATTCAAAAGTAATTCTTAATATTCTAAAAATGCTAACAAAATCTAAAGTATTATTTAGAGAGAAATTAGTACTTATTTCCATATTACTCATAGATTTTGGAGCACAGCAAAAAAGGAAGGTTTTTATTGCCTGACTTTAAATTAGAAAATTTAATGTATAAACTTTTTGTCACGTCTCTTGATTCTTTGTCGATGAATTTTTGACCTTTTCCGACCCTTTTTCATATCGAATCTTGCACTGTTGTAGTCTTTTAAAGGCAATTTTTCTTTAAAAGTCATTTTAAACGAACTTCATCAGAAACCTTTCGAATTATGGAAATATCGATAACAATAATATATTAACATAGAAAAAAACAAATGCAGGCACATATAGCAGTTTTATCTCCTCAAGTAGAGCTGTACAGAAACCAACAGCAAGTGTTGCAAATGGAGCAAAAGAGGCTGAATCAGAAGATTTTGAATTGCTCAAGGAACAAGCTTTTGAGAGACGGTGACGTTTTTAATATATATTTATATATAATATGCTTAAATTATTGGGTAATTATTTTAAATAGAAAATCAATTAGAAATATTTTTAAATATAATAATAAAAAAAAGTTCAAATTGATAGATAGTCTAATAAATAGTGATAAATGTCTATCAGTGATAGACAGTAACATAAGTCTATCACTGATAGATAATGATAGACTACTTTTTACCATTTTGCTACATTTGTAAATAAATTGGTTTCTTTTGATGAAAACAATCATAAATTGTAAGTTTGGTTTTTGAATTTTTTAATTTATGTCCAATAGATTTTTGAACTTTGAACTTTGTATCTAATAATATGGGTTTGAATTTTTTTAAATCACAAATCTAGTAGTTACAAGAATTTCCAGTTTAGAGATTAATATTAGATATAAATTTCATTTTGGGAGGTTATCAAATATAGCACATTGAATCAAAATATTTACAAATATAACAAAATGTACTGATGGATAGTAATAAACACTAATAAATATCTATATATGTCTATCACTGTCTATCTGACATTTTGTTATATTCGAAAATATTTTCAACAGTTTTGTCATTTAAAATAATTACCCTTTCATTTTTATATCTAACAAAATAAGTTTAATGATGATTTGATTTTTATTTTTTAAAATTTATGATTCTTATTTTCGGTAGGTAACATTTGAAAACAAAGTTTATTATATATATTTTTAGTTTACAAATTTGGCTTGGGGAATGAAAGCGTAGGGAGGTTTTGATTTAGGGTTTGGTTTTAAAATGAATTTTGGCAGCTGAAATAGAAGAGAATAGAGCTGAAGTGAATAGGCTGAGGGAGCTCCATATGAAGCAGCAGTGTGAAGCAAATGGATGGGATAGTAATGTTTTCACGATGCCACCAGATCTTCATGCATCTGAGCTCAGCAACATCGTCTCTCCCCAACCAACCCAAAGTAATTTTCTTTACTTCCCATTATGTCTATTTCTTTATCATCATTTAAATTCCTTTTCATAAATATTTAGGTTTTGAATCCATAATCTTATAATTTTACGTTTGAGATTTGAGTTTTGTTTTAATTTGGTATCTAGATTTTAAGATTTTATACTTTTAACGTAAGTCTTTTTTCACTAAATACTCACTCCCTGTTTTTAGTTAATTTATAAGAATTATAGTTAATTAAAAAAAAAAAATTCATCACGGTCAAAATTAATTTTAAAATTCGATGACTTCATAATTATTTTTAATTAATTAATATACATTAACAATACTCAAAATTGGAAGTGAGTATTTCATGAAAAATCGAGGTTAAAAAATGTAATTATCAAAATTTAGAGACCAAATTGAAACAAATTCAAACTCAAGAATAAAATTATAACATTTTGAAATTTATGGACTATATTGTAACCAAACTCAAAACTTTAAGAACTAAAAGTATACTTAGGAACCAAGTGAAAACTAAACTCAAAATCTAAAGACCAAAAAAAAAAAATTTTCTTTTTCGTAAATATTTTTTTAGTTGTGTTGATGTGTGGTTTATGTACTTTATATGCTACTAAACACTTGAACCCACATGATCCTAAAGATCATGATGTCCAAACAGGCCGACATCCAATTTTCGAAGAAAAAAATAGGATATGATCTAAACGGGTATTCAGTAATATATATATTTGTATTTATATTAGGATTCAAGATTATCATAATTCAATAAAAAATGGTGATTTCACAGAGACACTATCTTGAAGAGTATGTTAAAGAACTCAAGTTAAAAAAAATGGAGAGATCCACACTCTTTAGTCTAAGGGAGAGTTACTATGCTCATTATTAACTTATTGTAACTATTTTATATATTTTTTTGGTGCAACTTGGATCGCACAAAAGTATTTTTGATGCTGAGTTTTTCATTAGTTTAAATATATTCATAAATCAAATCGTTTATACTATTTTTGAATTTTTTTTCTAAAAAAAAAAAAAAAAATTGCTTGTTATTTCTTACACATGAATTAAGTAGTTAAGTAACAAAATAACGCGTGGTTTTAAATCTCCTTAAGGTTATACATATGTTTTGAAGTCAATAAGGTTTAATATTCTTTAAGAGAAGATTTGTCCCAAAAAAAAAAAAAAAAAATATATATATATATATATATATATTCTTTAAGAGAAGAAAACAAGAAAAATATTTAACTCCTAAATTATACTTATTTTTCACAAGTTTTTGGTAAGTTTCTCACTTTCTTTACAAAATACTCATCTTAATTTTGAGCCAAATTTAAAAAAAAAATGTTAAAAGCTACAATACTGTTGGTAATTATTTAATTTTCGATTTTTGAGAATGAAGCTTATAAATTCTACTTGTACCTATTGATTTATTTGCTTTGTTGTCTATTTTTTAGTAGTATTTTTATAAAAAAACCAAATCAAGTTTTGAAACTAAAAAATAATTTTAAAAAAATATATTATTTTTATTTTTGAAATTTGCTTTAAAATATAAATGTTCGGCTAGGAATGACAAAAATGTAGTAAGAAAATTACAACAAATTTCAAATATTAGAGATTTATCAAACATTTTTCAACTTTCAAAATTTACAAAATTTAGGTTGGTTTATGAAAAAAACTAAAAACAAAGTAATTATGAAACGAGGCAGGTGTTATTTTCTAAGAAGCCGCAAAAATGAATAGTTTGGTTTACCAAAACATAAATCCATATTAAATATCTATCCATGATTTTTTTTTTTCTTTTTTCTTTTTCAAATTTAGTAGTCCTTGACTTGATTTGGATTTGGAATGTTCATGTATGAAAATGAACCACGGTAAATGCAACAAATTACTTTTGATGTGTGCAGTGGAAAATGGAGAGAGAACAGTTGGAGGAAATGGGCTGGGCCAGTTCAACTCAACTTCCCGTCATAAAATGGTTGAGTACAAGTGGATGCCAACACCTGGGCTTCGTCAAGTTTCAAAGCCCAACTTCAACCAATTGGGCCAACAATGAATGGAAAAGCCCAACTAAAGCCAGTATTCAGAACTGCCGTATTGAACATGAGAAGTTAATCTGAGGGCTTTAAACTACATTTGCCATATACTTTTAAAAAAAATACTGTAAATTACATCGACAAGATGGCATGGTACAATTCCAAACAGTATTTTTTTTTTCTTGTCCTAAAATAAATAAATAAACCGTATCTTTAAAAATATCTAATACTTTCTTAAAATTTTCAATTGACTTTAATAGATAATTGAAGTTTAAAAAGAGGTAATAAGTTTTAATTTTGTTTTTAATAAGTAATTGACATGTTTAAATTTTCAAAAAATTAATTGATCTATTGGATGTGAAATTGTATTTTTATATATAATGAGACTCTATACTTTTAATTTTGTGCATGATAGGTCCTTGCTTTTTTTTTTTTTTTTTTTTTTAATGTAGCATAA

>ClabZIP16

ATGGGGATCCAGACGATGGGTTCTCAATTGAACGGCCAACAATCTCATTTACAACCTGCCCCATTGACAAGGCAAAACTCATGGTACGGTCTTACTCTTGATGAGGTTAAAAACCAGTTAGGTGAAATGGGGAAGCCATTAGGCAGCATGAACCTTGATGAGCTTCTTCATAATATCTGGACAGCTGAAGCCAATCAATCCATGGGAATGGAGAGTGAGAGTTCCTCTTCAGTATATTCTCTCAAACGTCAGGCCAGTTTCAAACTGGCCAGAGCATTGAGTGGGAAGACTGTTGATGATGTGTGGAGGGAGATTCAACAAGGGCAGAAGAAAAAAAATCACGAAAATTTGAAAAGTGAGAATAGTGAGATTATACTTGGTGATATGACTTTAGAGGATTTCTTGATACAAGCAGGGATTTATGCTGAGGCTTCTCCAAGTCCCATTATGGGCTTGGATGCCATTGATACTATGGCATTGGCAGAGAAGAATTTTTCACAGAAAATGGGCTTGTTGTCATCATCCCCTTCACTAGGCACACTGTCAGATACAACGACACCAAAACGGAGAAGGGATCCCTCAGACACACTAGAGAAGACTATGGAGCGGAGGCTAAAGAGAAAAATCAAGAACAGGGAGTCTGCTGCTCGCTCTCGAGCAAGAAAACAGGTCATTATTTGGGAAACTTTTCCTCCAAAGGACTATTTATTTCTCATATTTCTCTTGTCTGTTAATCTCTATAGCTGATCCAGTGATTCAAATGTAGGCCTACCATAATGAATTGGTGAACAAGGTCTCACGCCTTGAAGAGGAGAATTTAAAGCTCAAGAAAGAGAAGGTGAATGATTTTTCCGCATGTGAATTGTTGTGTGATCATGCACCATATTCCTGTTTGATTTATTGACTTTTATGTTTCAGTGA

>ClabZIP17

ATGATCGATAGAGTATTCTCCGTCGACGGAATCTCCGATCACTTCTGGACGTCGCCGGAGGAATCGTCGAAGTTGAACCGGAGCGCATCCGAATGGTCCTTTCGGAGATTTCTTCAAGAAGCTGCCTCCGTATCGGATTCCTCCATTTCTCCGCCTCCGGCTTCGCCTTCCGCGGCGGAAATTCGATCTAACGCCGTCGAGATTCGGGAGAAACAGAGTAATCAGAACGTTGGAGTTATGAAGGAAAGGGAAATATGCAGTAGTAGTGCGAGGGAGAAAAGTAGGGCGGCGGCGGCGGCGGATTCCGATGAGTATCAGGCGTTTCTGAAAAGCAAGCTGAATCTGGCGTGTGCGGCTGTGGCCTTGTGTCGAGTAAGATTCGACTTTTTCTTTCTCTCTGCTTTTCTTTTTGATATGTTCTTCATTCCATGGGTTTGGTTTATGAGATAATGGTCTGAGTATGAAGATGCAGCTGTTGTGTTTGTTCTTTCTTTTTCTTGCATTTTCTAGATTTCCAAACACTTGTCGGTGTTTGACCCACCAATTTTTCGACTAAATAACTCAATTCTACTAACTTTATTAGGGAAGCCTACACAACTCTATCTATCTTCTCTTCTCTCTCACATTTCCTCTCATGTTTTTGATAACTTCACCCATCCATCATAGTTGATGACCAAGTACAACTTTTGTTAGTGACTATTGTCGCCTCATATTGACAATAATCATCATCGCTTACATGCTAATAGCCACCTTTAACGGCAAGTACCTCTGGTGAAGACCACATTTGACAATCCATTTTGATTATCACCTTCGACGCTGAACTCTAATGACTACCTCTGGTAGCCAACTTCGATGTGTCGACAACTCGTTGTCGATAATTCCCAAAGACTAACTCTAACGACCGATATAGCCCATCATATATTAAAAAACAAAAAAATCGATTGGTTAAACTTAAAAAATAAATAAATAAAAATTTGAAATGACTAGTTCATAGTATTTAATAATCATATTTTATAATTAAGAAAATGTTGAAATAATTGTACAAATGACCTGTTTTAAAGACAAAAAATGTAAAATGACTCCTTTTTTAAAATTAAAAAAAAAAAATGTCAAATGACCTTCCACTAATATCATGGACGAGTAAAGTTACAAATTTGTCTTTGATTTCTCTCTTCCTCCTTCCTTCTTCTTAGATGCGATCACAACTTCTCCGGTGAAATTCTTTTTTTCTTCTTCTCTCTCCTCCTCCTTCTTCTTCTTTTTCGGCGAACTACCTTAAGCGAAACTCATTTTTCCTTTCTTTCCCTCTTTCTTTCTTCTTCTTTTTCACCGACACGACCAACTTTGGCATAACTGCTTTTTCTCTCTCTTCTTTCTCTTTCTTTCCTCCTTCGTCTTCTTTGGCACAACCAACTTCGGCGAAAAACGACACACAAACTTTGGCTTTAAGAGAGCAAAATGATATGAGAGTGAGGAAAAGAAGAGTGAAAGAGTGAGATCGAGGACGAGAGAGTGAGAGAAGGGTTATTTGTGAGTGAGGAAAGGAAGAGAGAGAGTAAGATTACTTTTCTACATGTTTGAGTTTATTTTAGTAATTTTACCCAAACATAACATATGTAAAAGGTCATTTAACATTTTTTTTTTTAATAAAAAAAACCAGTTATTTGCCATTTTGAACCCAAATTTTGGATCATCCAAAAATAACTTTTTAAAAATACTGCAAATAGGCCAAATTTTAAGTTTAAAAGAACATTTAACATTCCCTATTTTTTTAACCCCATTATACATGATTAGTTACAAAATAACAATTTTGACACTCCATATTAAAAATCTATATAACAATTGAGATGTATTTTTTCATAAAACAAATAGATGCATTTTTTTTAAATATGTATTCTCAGTTTTGGACATAAAGTTGTTAAAATTTTTTATATCATGTTGACTTATACAAATGTTCTAAACCAACCATCATATATGCATATAAACTCTAACCTACAATAAACTTTAAAAAATTCATTTTTATTCAAAATCTTTTAATAAAAAGAGTTTAAAATCCATTTCAAAATCTATTTTGGGTAGTTACCAAACATTTAAACTTTTTTTCAAAATGATTTGTTTTTAAAATTAAACACTTCAAAATGTATTCTAAACACACTCATAGTAAGATTAGGGTATTATGGGCAAATCTTCAGTTTATTAGAATTAGGATTAGTTTCTTTGATTTATTAGGATTAGGATTAGGATTAGTTTCTTTAATTAATTAGGGTTAAAATTAGGATTAGTTTGCTTTTTAATTCTCTATAAATACAAGAAGATAATCTCTCTATTTAAAGAGAATTTGAAAGCAACCTAATCCTAATCCTAACTAATTAAAGAAACTAATCCTAATCCTCATTAACTAAAGAACTAATCCTAATCCTAATAAATCAAAGAAACTAATCATAATTTTAATAAATTAAAGATTTGCCCATAATACTCTAATCCTACTACATCAAATAAGATGCAATAAAATCCTCTTAAACCCCTTGATGACTTTTCCTTAAAAAAAAAAAAAAACATTTCACTCCTACATTCCACTAATGTGACAGTATTTTGTAGTTTTGTTGATTCCATAAAATATTAGTATTTTATCTACCTCAAGTACAATGGTTATATATTCTCAAATATATATATATACATACAAAAAATTGTAAAATATATGTGAAGTTTCTAAGTTACCATATCAAGTCAGTTTTGAAAATTTGGAAAAATATATGTAATTAAATAGTTAAACAGAAGTTAATTAAAGAAAAATATCTAGCAGAAAACCTTACTTATTTAGGTTAAAAAAAAAACTACATTAGAGATCATTTACTTAATAATTCAACTCTTCATCCCAAAAAAAACTAGTTTAGATTAAACAATTTTGCACTCCAAAGATGGATTTTCCAACACAAAGACATATTTACTCTAGATTTTATAACTCAATGCAGCGCTCCAACCTACAATTTGATAACTAATTACTCTAATCATTTAACCCATAATTTTTTTTTTTTAAAAAAAAATTATTTTTAATTTTTGAAAATTCAGCTAGTGGGTTAAGTGTTTTCAAAATCACACTTATTTTTAAAATTATATTAAAAAAAAACTAAAACTTGTTTTTTAAGAATTTGACCAAAAATTCAAATGCTAAAATTTTAAAAAATGAAAACCTAGATTACAAAGTAGCTATCGATAGAGCCTTGATTTTTTTTTTTAATTGTTTTTTTGAAAATTCAGCCTATGAACAACCTTTCCATCTCAAAATTTCTTGCCTTGTAAACTACTTGTCACAAATATTTTTAAAAATCAAGCCAAGTTTTGAAAATTAGAAAAAAAAGTAGGTTTTAAAAAACTTATTTGTGTTTTTAGAATTTGACTAAAATTTTGACTCTTGTTGAGTTATGTTCACTTTGTCCGTTTGAATTTCATATTAATGCTCCATTAACATTAAAGTGATGAGTTCATATATATATAGTCTAACTATATCTATCATTGTCTATCACTGATAGACAATGACATTTGATTAAAATATTTCTAACAGTTTTTGACAATTAAAACAATTACCCTTTCATATATATATATATATATATAAAAATATCAAACTATTTACAAATATCGATAGAAATCTATTGCAATCTATCACAGTCTATATTGATAGACAGTAAAATTTTTCTATATTTGTAAATAGTTTGACTTATTTTGTTATATTTGAAAACAACCCATATATATAATCATGATTACTTAATAATTTTCCTTCATTCATGTCACAATAGGGATCTTTTATGAAGACTCAAGATTCTTGTGCAAGTTCCACTCAAGCCGGTGCCTCACACTTAACATCTCAATCCTCTTCAAAAGGTTTGCTCTTCTAAATTGCTACCTACCTTCTTACTTTACCCTTAATTTTTTTTACAATATCATAACGTAAATTTGGAGTATATATTTTAACCCATGACTCACACAAATCCCTTAACCACTAAATTATAAATTTATACTTATGTTATCATGTTTACTTTCAAAATTAAATTAATTTGGAATCATCCACATGTCTATTGTAATCATTATAATAAATAATGAAGATTAATTAGTTGAGTTTTTTCCCCTTACTTTTCCATGTATATTTCCCTAAATATTTGATATTTTTAAGTTTGAGTTCTAAATTATCTAATGTAGCTTAATTAGTTAAGACATATATTATCGATCAAAAGATCATAGGTTTAAATTCCACAAACCTAACTATAATTAAACTTAAACAAAAATAAAATGTTCTAAATTACCATAGAGAAAGAAAGGAAAAGAACATAATTATCTTCTAAAAAAAACCATAATTGATTAAACATTTATTTATTTATGACTAATTGAGATACAATCATAGAACTAAACATTTATTGTTAACTTCCCTCAAGATATGATATAAAGTTCTTATGGACATACCAATATTTTTATCCATATTTTAATATACTCGAAATAAGGCATTAATTTATGTTTTCATCATGGAAATATATGTATACATATATATCAAGAAACGCCAAAATAAGAATAAAAACTTAAAAAATGATAAAAACATGAAAGGAAGAACTCCAAACATATTAGAAGTTGAATCTAAAAGTATGAAAATGATATCTACTTTAAGAACACTAAAATCTACTTTATAAACATCTCACTAAGTTATTACACCTCTAAATTTAACAATTTTAAGAACACTAATCTCGAATAATAGTTGCCTGAAAAAGTTATATTTATTTTCAGTTCAGGAGGTTAGTATGAATTATGAAGGTCACAAGATAATGACATTAATGAACCTTTAGGAATTTCTTGCTCACCTTGTGTGCAAAAAAGGGCTGGAATTCTGGTCAGCTCAGCAAATATATCATCATCAAGAGAACAGACTGATGAAGATGATGATGTTGAAGGTGAAAACAATATGAATGAGCAAATAGATCCTGCATCCGCTAAACGTGTTAGAAGGTAACATTTATAGAATTTCGGTTAAATTATAAATTTAGTCCATAAATTTTGATAGTGGTGTCTGTTTCTTTACTAGACTAAAAATGTTTCAATAGCCATTTCCTACCCTTAACCTTGTTAGGTAAATAACGATGTTACATGCCTATTAGACACATGTTTACCGCATCATGACAAGTTTGCCAACTTGCCCAATAGACATATCATGTCGTCATTTAGCTCATAATGCTATCGGTGAGGACTGCATAAAAACATAATTCAAAGTGATTTAAGACTAAATATACAAAATAGTTGTGGTTGTGGGGACCAAATGAACTTTGAGAGAAAAATTGTTATTTGGATTAACGCAGTTGTTTTCAATTTTCATTAGAATGCTTTCAAATAGAGAATCAGCTAGACGCTCAAGAAAGAGAAAGCAAGCACATTTGACAGAGCTTGAAACACAGGTGATCTTCGAACTTATCTTTGTCTCAAAATTTATATATATCGTTATCAACCAGTGATAAGCCGACATGATTCATTATTGACAGGTTGCTGAATTAAGAGTTGAAAATTCAGCACTACTGAAGCGTTTCGGTGATATAAGCCAAAAGTACAATGAAGCAGCCGTTAATAACAGAGTTCTGAAAGCTGACCTTGAAACTTTAAGAGCAAAGGTAAGTAAATAGCTGGTTTTGATTTGCAGGGAGTTGAGACAGCAATATAAAGCCTGATCTTCTTTTGTTCTCTCTTGATATGACAATGCCAACGGATCCATTTCCAGGTACAGATGGCTGAGGAAACTGTAAAGCGAATCACTGGTATGAAATCTATGGTCCATGCCATGTCGGAAGTATCCTCAATTAGCATCCATTCCTTTGAGGGAAGCCCTTCAGAGATATCAACAGATGCACCTAACAATCATATTGCCGACATTTCTTCTGCAAATATTCAGAAGAATTCTCTGGAAATGGCAACCGTGTCAAGGAACAAGATGGCAAGAACAGCTTCCATGCGGCGAGTAGCAAGCTTGGAGCATCTTCAGAAGCGCATACGGGGGAGTTCCAGCTCCTGTCATCCATCAGGAAAGGGAGATCAGCAGTAA

>ClabZIP61

ATGGAGATGAATGAAGATTTGGGATTTGGTGAAAATCCATTTAATGGAAGCTTGAAAAGGCATTGTTCTTCACATTTGATCATGGAAACAAATAGGATGATGAGAAGAGAAGGTGATGATCATGAAGATGAATCAAATGGTTGTTGTGGTTTCCATAGAGAAATATTGTTCCCTACTATGATCACTACTACTGGCCAAGCCCCAACCACCACCAACAACAACAATTGTAATGTCTTCTCTCCCAATTCCGCTTCTTGCTATAGCAACGACAACATTTTGGACGTCGTTGAAGTTCTCGACATCCATCGTCATCACCATCTGACTGTAATGGCCGAGAGGAAGCTAAGAAGAATGATATCAAATCGAGAATCCGCAAGGAGGTCACGAATGAGAAAGAAGAAGCAGATCGAAGAGTTACAATACCAGGCAAGATTTTCAAATATATATCTACCTTAA

>ClabZIP18

ATGGGAACTAGTGAAGAGGCGAAGTCTGTGAAGACTGAAAAACCATCCTCACCAACACCGGTATGTTGATTAGTGATTATTTCCTTTTTTGAGAGGGAGCAAGGTTTACCTGTTTTGAGTTGTTTGACTTGGATAATGATTTCTCTCTATACATTGACAGGATCAGAATAGTGTGCCAAATTCAGCCAGTATTCATGTTTTTCCTGATTGGGCAGCCATGCAGGTTCACCTAGGCAGCCCCCATCCACATTTTGCCTATTTGTTTCAAATAATCACTTAAACATGTGATAAGATTATAATGGCTAGATTGTTATAGCTAATTGAAGGGATTTGTCACCTTTTCAGGCATATTATGGTCCTAGAGTTGCAGTTCCACCATACTACAACTCTGCTGTAGCTTCAGGTCATGCTCCTCACCCTTATATGTGGGCTCCACCACAGGTAGTTATTAACTATTGGAGAGTTTAAAAATGATGAAGCTTTTTCTATCGAGTCATGCTTGGGGATAGAAATTTGTTTCACTGAAAGTTAATTCATTTTACCAGATGATTCCTCCATATGGTACTCCTTATGCTGCAATCTATTCTCATGGAGGTGTTTATGCCCATCCTGCAGTTTCTATGGTAAGTTTGTATTTTTGTTAGCTTCTACACGGTTTTGTTGTTATTGCTCACAAACGCATGAGAAGTAGTGTAGGTATTGTCTTTTGTCTGCCTTGCTAAACTTCTTAGCCGGAAAACCAACATTCTTTATTTTTTCCAATTATCCCTGGATAAGGTTCGTGCATGGCAATCTCAACTGAATCATAGACGAGTTAACTGTAGCAACTGATCTGTGAATTGGATTGTTGGAGATTAAAATTAAGCAGTTTGTTATTATGAAGATGAATGCTTCTTATTTTCTGCTAATCATTGAGCATTTCCAGACTAACTAATTGTATGATTATAATATTAATGCAGGGACCACATTCACATGGCCCTGGTGTTCCCTCATCACCTGGGGTAAGTGGAGCCTTCATGCTGGAGTTTGTATTTGAGTTGTTGAAATTGTATAAATTTGGTCTGTAAATATTGTGAAAAAGTATGAAATGGAGGTGAAAGAATGTACTGGCTGTTTAAGCCACTCAAGAACAAATACTAACGAAAAGCACATTGATGCTCATAGCAGGCTGCACCTCCTTTGAGTATTGAAACACCTTCAAAGGTATCTGGAAATAGCAGTCAAGGTTTAATGAAGAAACTGAAAGGTTTCGATGGCCTGGCAATGTCCATAGGCAATGTTAGCACTGAGACTGCTGAAGGGGGAGCTGAGCATGGACAATCAGAGAGGTTTACCAAATTTATTTAGTCATCATCTTCTAATTTCCTGTTGAGAATTTATTGTTCAAATTTTTCAAAATTAAACTGCCCCACATTTTTTTATACTCATATTTATCTCGTGACTCACCTAACTTAAAATTTCTTTCTGTCATGTTAACAGTATGGAAACTGAAGGTTCCAGTGATGGAAGTGATGGAACTACTGCTGGGGTAAGATTTCATAGCTAGAGAAGAATTCTTGGTTCATCTACGCTAACATCTTATAACTTTATCTGTAATTACTTGGTGATGAATAGTTGGTATTCAAAATCGTGATTTCTTTTATCTTATATTATGTTCAGGCAAATCAAACCAAAAGGAAACGAAGCCGGGAGGGAACACCTACCACTGGTAAGACTCCACTACCAAGTATTTTCCTTTCAAAACACATGGTGTTGACTGTTGCCCAATGATCTATACTTTCTCTTGTTGGTAATGGGTTTAAACATTTACATCAACATGAATACTCATTGTTTGTGATAACCCCCTTCTAATTAGCAATGAGTACATCTTAGGGTTGTTACAGTATTTGGAACTTGGAAGTCCATGGACGCCCAATCTCTTCTGAATTATAGAGTGACATTAAAGAAGTGCTTCTAGTTAATTAGAAATTTTCAGAATCCTTTCAAGAGGATGCCTAATTGCTTCCATCCATGTAGCACGATGTTCTAAAACTATCATGGTTTGTTTCCATGCATGTATCATCTGCCAATTTGGTTTTTGTGAAAAATTAAGCTAGTACGATTTGCTAACCTTAAAATGCACTTCAAATTAGTCACATACAGATTCAATTTCTGAATAATCTGCTAATTTTCATGATTATGCCAAGGATTCAATAATAAGCCTTAGATGTTTCATATGTCATATGTATGAGATTTTGATTTCTTCAGTCTCATTGCAGGTAAGGATGCAAAAATTGAGCCACAGGCAAGTCCAGTTACTGCTGCTGAAATGAATGAAAGCTCTAGCAAATTATTGGGCACGACCAAAGCAGCTAATGCGACTGGAAAACTTGGATCTGTGATTTCTCCTGGAATGTCTACTGCATTGGAACTGAGAAATCCTTCCAGTATAAATGCCATGACAAGTCCAACGACGGTTCCACCTTGTTCAGTACTGCCTTCTGAAGTCTGGCTCCAGGTATAAATCGTGCTCTAGTTTTTCAACACATCCCCTTATGTTTCCAGACAAACTTTGGGTCAATTAACTTTTAATTATGCATGATCTCATGTGAACATTGCTTAATTTGATGCCTTTTCATATTCTGCGCAGAATGAAAAGGAGCTAAAACGGGAAAGGAGAAAACAGTCGAATAGAGAATCCGCTAGGAGGTCAAGGTTGAGGAAGCAGGTAATGTGGAACTTAGAAGAATAAAATATTGAATTTGAATGTCAAGCCTCCCATTGTCTTCCCCGCCTATCATTTTATTTGAAGCTAATATAATTTTATTGGATCATCATCAGGCTGAGACAGAGGAACTAGCTCGTAAAGTTGATTCGCTGACTGCCGAGAATGTTGCTATTCGATCTGAAATAAGTAGATTGTCAGAGAACTCCGAGAAACTTAAGAAAGAAAACTCTACCTTGATGGTGTGCTTCTTCCCTCTTTGATATACTCTTTAGTTTTTCCTCGTCAGAATCAAATCTAAAAATTGATAACAAAATAAACTGCCTTATAACTTGACAGGAAAAGCTTAAAAGCGCTCAATCAGGACGAACAGAAGCATTAGACATGAATGAAAAGAGGATGCAACAACCTGTAAGCACAGAAACCAAAGGACCTGTGAATAAAAGCATTAACGAAGAGAGTATCATCTGCAAGAAAAACTCGAGTTCGGGTGCAAAACTGCGGCAGCTCTTGGATACGAGTCCGAGGGCGGATGCAGTCGCTGCTAGCTAA

>ClabZIP19

ATGGAGAAGGACAAACCCCAAGGCTATTCTGGTGGATTTCCAACACCATCAAGTCGTTACTCAGTTCTTTCACCAACTGGAAGCATTTTCAATGGAAAATCTGAGGCAACTTCATCTTCAATGTTGCTTCCTCCGATGCCTTCAGGTGCTCTTTCTGATTCAGGTCAATTTGGTCGTGAAAGGCCCACTGATTCTAATCGGTTCAGCCATGATATTAGTCAAATGCCTGATAACCCACCGAGAAATATTGGTCACAGACGTGCCCATTCAGAAATCCTGACCCTTCCTGATGATATTTGTTTTGACAATGACCTTGGTGTCATTGGTAGTGCTGATGGCCCTTCTTTTTCTGATGATACTGAGGAAGATTTGTTGTCCATGTACCTTGACATGGACAAATTCAATTCTTCGACTGCCACCTCCGCAATACAAGTTGGCGAGTCATCTTCTGCTGTTGGAGATGCAATATCAACTCCTACTCCAGCAATTGGAGCAGCAACCTCTAAAGATGATACTGCTGTTGGTTTGAAGGAGAGGCCGAGAGTTAGACACCAGCATAGCCAGTCCATGGATGGCTTGACAACCATAAAGCCTGAGATGCTTGTCTCAGGGTCTGAAGAAGCCTCTGCAGCCGATTCCAAGAAAGCCATGTCAGCTACAAAGCTCGCTGAGCTTGCGCTTATTGACCCCAAACGTGCTAAGAGGTATAATTCCTTCATAATTCTCTCTCTCTCCCCCTCTCCCTCTTTAAACATGAAAAGAAACTGATAAATGTGTTATTTCAAGATGTATCTGTATCCCCAACAGATTTTGCGCATTGCTTTTTTTCAAACCCAATATACCTTTTGTGCATTGCTTTATTTTGCTCATATTTACTATTGATGTTCATATGCTAGTCAGAACTACCGTTGCTGTACAATTTATGATAACTCAACATTTGAACTTCGTTGGATGCTTTGCCTAAATTTCAAGAACCTGTGATGGTAATGAAGTGTTTTTGTCAATAGTTGACAAGTAGTTTAGGTTAAGCTTCTATTATTATTTTTCTAAAACATGAAACGAGAAGATTGTTGAGTTATCAAGTAGTTTAGGTTAACTTCTGTTATTATTAGTTATTGGTTTTTTTATTGTTGCCGATTATTTTACAGAAGTTATTCCTATTCTTTCTGTATATGAAAGGTTGTTACAAAGTTTTAGCTAGTAATTAAACTCTGGGGATTCTGTAATGTTGTACAGCATTGCTTTCTAATAAATGTTTTTTCCGTATATTTAAGTGGTAGGAACCAAATCATTTGTCTTTGGCCGTGTATGGTACTTTTTCTTTGTAATTTCTCGTTTGATGAAATTACTTTATTTATTTTCAAGATAAAATAATTTGTCTATCTAGAGTTTCTTTGGGGAATTTTAACTCTAAGAAAATGAAACTCGATCTTTTGTGCTGAGTTATGAGGCAGCTTCTAGAATTTAGCTGGTGTGTGATCATTTACATGGATTATGTTTCTCTCTTATCTCGAAGAGCCTGGAGACCGTTTTTAAACCCTGTATATTCCCTCCCCTTCTCCATTGTAAGCATGTTTTTCTTGAACTTTTTCATGTTTGGGATTATTTGCTTTAGCGTCCTTCAATTATTATGTTCATTCTGTTTCCTGTGAAAAATGGAAAAACAAGAATAGAAAATTTGTTTGTAAATCCCCTTTGTTGTGCATTGTATAGGATATGGGCAAATAGACAGTCGGCTGCTAGGTCAAAGGAAAGAAAGATGCGGTACATTGCTGAGCTTGAACGGAAAGTTCAGACTCTGCAAACAGAGGCAACTTCTTTATCTGCTCAGTTAACCCTCTTACAGGTGGATTGTCAAATAGTTTATCGTGCGTGTTTGATCGTATTTTTGGATGCTCATTTTATATTTTCATACCATTTGAATTTCAGAATTCTCTTATGTTTTACTGATTGCCTTCTTATTATCAGAGAGACACAAACGGTCTTTCTGCTGAGAACAATGAATTAAAGCTGCGGTTGCAGACAATGGAGCAGCAGGTTCACCTGCAAGATGGTAAGACCTCTATAGTTTCATTTTGATTGCACATTTTTTAAATTGAAACAAAAATTTTCATTCATGAAATGAAAAGAGGTATTGCTTGAAGTACAATAAAACATAATAAGCAAGATAATAGCGCAAAGTTGGACTTGTTCAGAAAATTAAAGTCAATACAATCAAATCCAACCCAAGAAGAAACAAATTGTCTCTTTGAATGAAAATTCCAAATGCCACTGAAACTGAAACTCTGAGAACGAGCAACCATGTGGTGAGGATAACTTCTAATAAACTGGTGATAACCAACAATCCATTAAACCAGAGCCGCAAGAGAGGAACAAACTGACTAGATTAAAAGGAGAACCACAACGCCTTAGAACCGGCAAATTCAAATAGAAAGAGACCGCTGAGAACTTGGCAAGGGGCAAACAAAATCAGAAAAATCCAACCTCCCTCCAACTATGGCAACTTGGTGACAAAGCCACCCTAAAATTCCAGCTTCTTGAAACTGATCAAGCGAAGAAAACAAGCACAACAAGGAGATCCATCTCATTTTTTCTTCCTTTTTTTCCCATACTCAGCAAACCTTGATATCAAAGCTACTTCAAAATTCTGAGATTAGAATAAATGACAAAACCTGCCCACCCTTGCAATCGAAGATCATCAATGCTTCTTTAAAATTATGAGACTTCGGTGATTGCCCATTATAGCTGCCTTCTCTTAGTTTTTATCTAATCGCTCCATTTTTAATTGTTGAAATGTAGGTCCTTTTTGGAAGATGATTTTATTTGCATTTTTGGCATTCATAGACATTGTCACATTGAAAGTTGCTTTAGTCTCCAACTAA

>ClabZIP20

ATGGACAAGGAAAAGCCCCAATGCCATGGGGGAGGATTTCTTCACCAATCAAGTCGTTACTCGGGGTTTTCATCTGCTGAAACTAGTTTCAATGGAAAATCTGAAGCAACCTCGTCTTCAATGTCATTCCCTCCATTGGCTCCCAGTACTAATTCTGATTGGGCTCAATCTGGCCGTGGAATGTCCACCGATTCCGCTCGGTTCAGCCATGATATTAGTCGGATGCCTGAAAATCCACGGCGAAATGTTGGTCATCGGCGTGCCCATTCAGAGATCTTGACCCTCCCAGATGATATTTGTTTCGATAGTGATCTTGGTATTATTGGTGGGGCTGATGGTCCTTCTCTTTCTGATGATACTGAGGAAGATTTGTTGTCCATGTACCTTGACATGGATAAATTCAATTCCTCGACTGCTACTTCTGCAACTCAAGTGGGCGATTCATCTTCCCCTCTTGTAGAAGCAGCAGCAACTTCTACAGATGATATTGCTGTTGGTTTGAAGGAGAGGCCGAGAGTTAGACATCAGCATAGCCAGTCCATGGACGGCTTGACAAACATCAAACCTGAGATGCTTGTCTCTGGGTCTGACGAAGCCTCTGCAGCTGATACCAAGAAAGCCATGTCAGCTGCAAAGCTTGCTGAGCTTGCTTTGATTGACCCCAAACGAGCAAAAAGGTATATTTGCTTCAGAACTCCTTTAAGTTTTTGTTTGACGTGTAAAGAAACTGATTATTTACTTTTCCTGGCGTATTCTTGTACGATGTATCCAGAATAGATTGTGCATGCTGCTACGTTTGCCCATTTTCTAGTCAGAATCATTTTTGCTCTTCATTTTATGTACGTAATGCAACTTTGTTTCATATTTTAACGTACTTGCGAGAACTAAATGATGGTATGCAAGTGTTTCTTGTCAATGTTTGAGAACAGGTTAGGTAGCTAACTACTAATTTTAGTTTCATGGTTTCTTCATTGTGGTTATTACTACGCCTTTGTGTGTGTATGTGACATTACAACGTTTGATCAAAATGTGATAGACTTCGGGGGCTTCCCCTTATTGGTACAGTATTATTTTATATTAAATGAAAGTTTGCTTATATTAGGATGAGTAGGTATGATTTTTCTTTTTCGGCACTGTATAGTATTTGGGGTCGCCAGCCCTGTGACACTTCGTACTGTATAATGCATTCCTTGTAATTTCACTATTCATTGAATTAACTTTGTCTATTAGCACGATTCAGAAAATATCAAGACCTTGATGGTTTTCTGCTTGAAAGAAAAAACAGAACGCAATCTTGTGCTTAAGCAGTGACTCAGCTTAGAAAATCTAACTGTATCTGATTATGGATAATGTTTCTATGCGCTCTTATCTTAATAATATTCAGTATGTTTCCTCATCGTTAATTCAATGAAAAAGTCTTGTTTCTGTTGAAAAAAAAAGATAATTTTCAGTATGTTTCCTCTCCATGCTAAAGTGAGTTTCCTCCAATCTTTTCATGTTTGAGACTCAAAAGAGGTTCATATCTGTTTTCCATGGCTTATAGTTATTGTGTTTGTAATCTTCTCTGTTGTCCAATGCATAGGATATGGGCAAACAGACAGTCAGCTGCGAGGTCAAAGGAAAGAAAGATGCGATACATTGCTGAGCTTGAACGGAAAGTTCAAACATTGCAAACAGAGGCTACTTCTTTGTCCACTCAGTTGACCCTATTACAGGTTGATTGGCATATTATTTTTGTTCTCATAGTCTCACGTCCTTGTATGCACATTTTTGGAGACTTATTTTATGCTTTTGATACTTATTTCTATCAAAAACATCATTTAGAGTCCCACGGTCTATTTTATCTCCATTTCACTTGTAGAATGATATCATTTTTTTATTGTCTTCTTACAACCAGAGAGATGCAAATGGTATCACTGCTGAGAACAGTGAACTAAAGCTGCGGTTGCAGACAATGGAACAGCAAGTTCACCTGCAAGACGGTAAGGACCGGGCATTTATATTTGCCTTATTTTAATTTTGTATCTGATCTCTCAACTTTGTAATTATAGTAGCACATGCATATACTTGTTTGAAGAAATGTTTGTATGCATTTTAGACATTTCACAGACGTCCAAAGTTGCTTTCTAGTCTCAAACTCTTCATACTGCTCTGTAGGAGCTTTCACAGTATCAGGTCCATCAGGACTTGGTACTGGAATGATTCAATTGGACATGCATTAG

>ClabZIP21

ATGGCAAATTCTAAAGGGTCATCCAACGTCAGAAGTTTTATGAGTTCTGGGAAACATGCACTACTCCCTCCTAAAAGTCCCTTTCCTAGTGTTTCTCCATCATATACGGAATATGTTCCTAATAGTGTAATAGGAGCAAAAGCTGTTCAGAGACCAAGAGATGGTAACAGCTACCATCAAAGAACTTCTTCCGAAAGTATTTTAATAGAGGAGCAGCCTTCTTGGCTTGATGATCTCCTCAATGAGCCAGAGACCCCTGTTCGCAGAGTTGGTCATCGACGTTCGTCGAGTGACTCCTTTGCATATACAGATGCTGCTAATGTAAATTTTGATAGTATCATGCAAGAAGAATTTAGATATGCAAATGCAATTCCTGGACACTCTTGGTTATCTCAAGAATTTGATCATCAGAGAGATGCAAGGCATGTTTCATTCTATACTGAAGCGAATATAGCAAAACAGAAGAATAGGGTGTGGGAGTCGTCTTTATCTACCATGAATAATCCCATTGCCCTTCATTCTCCTAGGGAAAACATTGGTATTCATACCTCAGGGCCACTAAACACGCCACAGGAAGCAGATGGTTTGCCTTCTACGGCAAGTGAGAAACAGGATTCAACTGAGTCTGGTTCACACGATCCAAAAGTCTCTTCTGACAGGAAAGATGCTTCTCATGGAAAATCATCTGTGTCTGATACAGAAAATAAACGCGCAAAACAGTATGTATATGATGTTTTACTTTATTTTAGTATCTGTGATTCTGTATACATAGACTGGAATGAATCTTGGCTAACATCTAAACCTACAGAGCTTGTTTTAATTGAATTGCCCAGCTTTTTTTTTTCTTGCTTACCACTTATAACTGTATTTTAAAGTCTTTTAGGTTTTTATAATTCTTTGAAGATTTTTATATGCCGTGAAGTGTTTTATAAAACCCATGTCTTACTAGACATTATTTTATCTGGTATTTTATTTATTTATTATTTTCTTTTATGTTACACATTTTAAATTTTTTTTTCTTTTCTAATGATATCCTATATCAAATCTCCACACCTAGAATTCCTTTTCTATCTTCGATCACTTGTTCATCATCTATAATCTTTCAACCTGGTATAGGAGTAAATGACTTTATTGAGTTGTATTTCTACAATATCTTTTTTAATGCATTGAGAATCAATTGCTTTAGAACATTCCTATATTTTTCCCTCCAAAACTCTTGATACAACTTTTACATAGAATTGTCGTGATTTCTCCTGTCAACAAGTGAATGGCTACTACATAGCAAAAATTTGAGTAGCAATGGATTAAATTCTGGTTTTCAAGTCCAGTGGCAGTAATCAATACAACTTAAACTTGCCAAGAGTTTAACAGTTAAATTATTAGAGTCAAACTATGCCCTCAAAAAGTTGAAAATAGGGAATTTTACAAGTTTTCTTGGCACCAAAATGTTGTAAAGTCAGGTGGGTTGTTTGGGTAGATTAGTCGAGGTGCATGCAAGTTGGCTTAGACACTGATAGATATAAAACAAAATTAAAGAAAAAAGTCCTATGAGTTTCCTTGACACCTAAATGTTGTAGGGTCAGTAGGTTATCCTATGAGATTAGTTTAGGTTCATAAGCTAGCTTGGACAGTTACAAATATAAGAAAAGGAAGAAGAAAGGAACTGCCGACCTTTCATTCTTTCATTTTAAAGAATAATAAGAAATACAAAGGGAGATGACACCTTCTAACTCTTGTGGCGTGACCATAAGATGGAAAATAAGGACACCTCCTCCTAGTTTGTGCTCTATGAATGAAATGTTTGTTGCTATTTGGTCATTTTCCATCTAATTCGGTGTTGAATGCCACAGTATAGGTCATTGGATTCCCTGCAGTGGTCACTAGTAATTTTTTTGGCCACAAAACTTAAATATGCAATATCATTTGAGTTTATAGACTTTGAAATGTTCCAGGGTCTTGATTATTATCTTAAATGTGTTTGGATTGGTACATATTTTATTTTTACTTGTGATATTATGACAACAATGATGGTCACAGTGCAGTACATTTCCTTCTCATGCTGATTTTCCCCCTCTTGTCTCTTCATACAATGCTAGTTTTGTGCTTTTCATGTTTCACTGGGTGTCATTCAATGATTTCTTCTTTATATTTATATATATATATATTTGTTTTCATTAATCTGACACAGCATGACATTGAGATGGTTTTATTAATACTTGATAATGTTGGTCCTTTATTTTAGAGTGGATAACCGTGATCCTGTTAAGTGCATTTGCAACTTGCATTTGGTTTAATTTTAAAATCAAATGATACAGGCAATTTGCTCAGCGTTCAAGGGTTCGGAAACTTCAATATATAGCAGAGCTTGAAAGGAAAGTACAAGCTTTGCAGGTCAGTATTGTATGCTGAAATTATGAATTAGTGGGTTCATACAGTTGATGTAAAAGCTCAAATGTCACCTCCATATTTGTTATTCCATAGGCAGAGGGCTCTGAAGTCTCAGCTGAGCTTGAATTTCTCAACCAACAAAATCTTATTCTTAGCATGGAAAACAAAGCCCTCAAGCAGCGGTTAGAGAATTTAGCTCAAGAGCAGCTAATTAAATACTGTAAGCTCTTATTCCCTAGATATCTCGCATTCTTTGTATGTTGA

>ClabZIP22

ATGCAGGAACGAGCCGCCGCCGCAGCCGCCGGTCGTCTGCGCTCGAGTAGCGAAAGATCGTCGAGCTCCGCTTTTCAACTTGATGTTAAAGAAGGTCCGATTTTAATTCCTTTCGTTTTCTTTACAGTCTCTCGCGGAGAAATTGTACGTGAGGTAGTGTAACCGGATGTTGTTACTCGTTAGGAGTAGTTGTTTTTCTTGTTCTTTTGATTGATCGGAGTCGAGTTTATTTTGAAAATCAAATCTCCTGGCTAGCTGTGACTGTAGTTTCATTCGATCATTGTTCTTGTTACTATCCTTTTTATACGGCGAAATTGCTAGGGACTGTGTTTTTGATTGAAACATATTTCCTGACCTAAATTCGTTACAACTTGATAGTTTCTCCTCCGTGATTTTCCTTTTTCGTTCAAGATTTCGAGAATTTGTTGACGTAGAACCGTGTAGATAATTAATAATCTTAAATTTCCTGGCCGAATCGGATCCTGTTTTTGTTCGTTTGCTGAAGAACTACGTTATATGATTTTTTTCTTTCTTTACCGAAGATATAGGAGCGGAGAGTGATGAGGAGGAGATAAGCAGAGAACCGCAGATCTGTGGCAACTCCGTCTCTGCCGTTGGCATCTCAGCATCTGGTAAAGCGCCTGCATCAGATAGCATAAGGAGCAGAGGACGAAGCGCCGCTGAGAAAGAAAGCAAAAGGCTGAAGAGGTAAGGTTTGTAGGCCTAGAATAGGGACCAAAATAGGCATTCAATTGCAAATTTCGGCACAGAGTTTCATTTTCGTAGCTCGTTAATCACTTTTTTCTTGAAGATTGTTAAGAAACAGAGTTTCGGCACAGCAAGCAAGGGAAAGGAAAAAGGCGTATTTAAGCGAATTGGAAAGCAGAGCAACAAACTTGGAGAAAAGGAACTCGGAGCTTGAAGAGAAGTTGTCCACGTTACAGAATGAGAACCAGATGCTTAGACACGTACGTCAAACTAATCCCCGTCGATACCACTTTCTCTTTTAGGCATCATCTTTACGTTTTCTTTCCATCACAAATGTACTAGTATAGGTCTTCTTCCTAATCTCAGCTCCATATCCACTGATTTCTTGTTGAGCAATGATTTGGTACAGTCTGCCTACGTGCTCCCTCTTTTTTTTAGACATAAAGAAAAAAAAAAGTAATTCAAATTCTTTTAAAAAATTGTCACATGACATAATTTTATTGGCCAATTTCTTGAATTTAGGTGCATCCAAGGGAGCACCCAAATTTTCTTTTTCTTAAATATATGTGTTTTTTTTCCTCTCTCTTTTTTTTTTGGACAGATTTCTTATATATGGGTTAAAGAATAGAGTTATATTTATTGTTTTTTTAAGGAAAAAGAATAGAATAGAGTTATTTATTTCTTTGATGGTTGTCTACATGAATTTATTTTGAGATTATACCTAACCGGTGTTTAGGGGTAAGGCTGGAATAGTGAAAAGTAAGTAGTCGTGAACTTTTGTGGTCTATTGTTTAAGGAGTTGATAAAATATAAAAGGAAAAAAGTTGTGAACTTTTGCGGCCCACAGTATAAGGAGTCGATAAGATATAAAAGGAAAAAAGTTGGATACAACTTCAACCCAATGAAAATTTGTCTTGTGGCAATTAAATTTATATATATTTTTTTGTGTAGAGAAAGGGGGTGTAGTTTAGTATTGCTCGATATAAAATTGATATTCCTTTTTAGTAGTGGGACTCACCAACTCTTTAGGCCAAATAAGTAGTGAAGTTCACAACTCTTATTTTCTAACTCCTTGGGCCAAATACATCCTAAAAGCTCATATTAATAAAGATATTAGCGAAGGTGTTTTTTATTTCTAATATGGAAGACTTATTTGTAACCTGATTTTTAATTATTTTTTGTTTTTAGGTTCAAATTATTAAAAAAAGAAAAAAAAGTAAATAATAAAGTATTTATTTAAAAAAAAGTGAGTTTAACTTAATGATAATCAACGTGACTTTTCTTCTTCGAGGTTGAAGGTTAGATTTCTTATTCTCCGTCATTGTATTAAAAGAAGTTGTTTATTTTCTACTCTGTCTCAAAGTTTCTAGCTTTGTCTTTGGCTGTTCGCTTAGATTTGTGTATTTCAGGGACTAAATATGTTGTGATTGAGATTATCCAGTTACTAGTTCATGTTAGTGTAGTGTTAATTATATAATGTAAAATTCATAGATTATTTCTCCATTTTTGGTACTTTGCAGGATCAAATGAATTATATTATTTTCGCCTTTCGATATAATTTGTTTACTAGATAATAATGACTTTGAATAATATATCTAAGAATCCTTGAATTCACTTGTTAATACGATTATATATAGATACTAAAGAACACAACAACCAACAAGAGAGGTGACGGCGACACTGCAAAATGCTAATGCAAACCAATCCCGTATAGCTGGTATGAAGAAGTAAATTAATTATAGTATGTGTACATACTTAACGGTATCGATGGACTTGAAGGGAATGAAAAATAATAATTATGGGATAAGCTTTCATGTGGAAAATTATTTAACATGACTAATGCAGTAGTTGATACTTTTAGGGGTGTTTGATAGTCCAACTTGAGTTGGGTTGGGTGGGTTATCAAATTTAGTCGTTGTTTGTTTCACCGATAAAAATAAAATGGTGGGTTTAATAACTCACCGATTTATCATTCTCATTATCTCATGCATTTTGGTTATCCTCCAACTCCCTCATTTACTTTTGGTTTCTCTCTTATTCATGGATTACTCCTCTTCTCCCTTCATCTTAATTCTTTTGCCATCCGGTTTGTCTTCTCTCATCTTTTGCCGTTCGGTTTGTATTTCCCATCTTCTCTCTTCTTTCTCCATTTGACTTGTATGAGTTTTATTTTCCTTCTCAACCTTTCTTGTTTCTTCTTTGATTTTGTCTCTATTACTGTTTTTTTTTTAAGATCAACCATATACAAACTTGTTTTGTTGTTGAGCTCAAAAACGTATCACTCCATTACAAAATATGGGTAGTTAATTAGGTTGAGTTATTTGCAAAGGAAATTAATATTTTTATTTGTAATTGTCTTTTATTTTTTTTATTTATCATACAACTTATATTATAAACTCAACTGGCATAAAATATGAACTATTGACTAACAAGTTAGAGGTTTGAATCTCAAATCCAATATTGTTAATCTAAAAAAAACCTTGAATTGAAAGGATGTTTTTAACTAAAACAAAATCAGTCAAATTATTTACAAATATAGAAAAGTTTCACTGTTTATCCGTGATAAACATCTATTGCTTGAGTTATAGATTACGATAGATTTTATCACTCAAGCGATAGAAGTCTATATCACTGATAGATTGTGACATCCTAAATTTTCAGGAAATTTTTAAAAAATTATCTTGAATCATTTGGGTAATTATTTAAATTTTTAAGAATTATCTTGAAAATATAATTCAATTTAGATTGAATGTGATCAAAGATTTGGGCTTAATTCAATTGGTTTAAGACTTTGGTTAATTTAAGGAAAAATGGATAAATTAGTGTGGAATTGAAATTTAAGTTGGTGGTATTGGTTGTTAGGAGATTTTTAATTAAATTATTTTGGGTGGTTAGAAATGATTTGATTTTGATGTGTGGAAAAAAAGATTAAATAAGTTTTATATTTTGTTTTGGAAGATGGAAAAGGAAAAAGAAAAGAAATAGTATATTTATATGAATATGAAATTAGGTTTTTCTAAAAAGGAAATAAGATATATATATATATATATTTATAATAGTGATTTTGTGAAGCAAAAGAAAAAGAAAAAAAAAAGAAAAAAAAAAAAGAGAGAGAGAGAAAACATTCTTTTTTCTTCTCATCTTTTTGACCAACGATAGCAGTCGAGACCCACTCCTTTAGCTGTTGTTCGTTCCGAGTTCCAGCCATTGTCGTCTGCGGGTGGTCGTTTCTTCGTTCATCGTCGACTCTCTCTTTCCTGCTGGCAACTATGTCTTATCCTAGTGCTGCCACCGCCTCCACTCGATGATTTGCTGCCATGGGTTTGGTTCCTCCCTTGTCACCCGCCGCTGTTTACTGTCGCCAGAAAGGGTAATCTTTACTAG

>ClabZIP23

ATGATTATGGACTGTTCTAGTGGGAATTCTTCAGGTTCATTATCTCAGATTGTGCTTCAGAACCAAAGCTCTGGTTCTGAAGAAGAGCTGAGGCAATTGATGGACCAAAGAAAAAGAAAACGAATGCAATCGAATAGGGAATCGGCGAGGAGATCGAGGATGAGAAAACAGCAGCATTTGGATGGGCTAATGGCGCAAGTGAGTCAACTCAGAGATAACAAAAACCAAATGATTTCAAGAATTAATCTCACAACTCAACTCTTTCTCAACATCGAAGCAGAGAACTCTGTTTTGAGAGCTCAGATTCTGGAACTTACTCATAGATTAGAGTCGCTAAACCAGATCTTATCTCACATCAACGACAACGATGATGAAGAACAACATAATTTTCTTCAAAACTTTGATGATTTTGATCATAACCCATTATTCATCAACTCCTTCTTCATCACCCAACAACCCATTGTGGCTTCTGCTCATCATCACCTGCTTCACTACTGA

>ClabZIP24

ATGCTTCCAGGTGAAATGACTGGCATCCAGTTTTTTCCATCCGAAAACTCTTTTCAAATTCCTTCAAACATTGGCATGATGCAGAACAGCTTTCAGACTCTCCATCATTTCAATAGCTTTTTAGGCAACCTTCCGATGTCTCACGTCCCTCATCCCAGCCATGAATTCCTTGCACAATCCTCAAGTTTTAGCTACAACTCAACATCTGATGATGCAGAGGAACAACAGAAGAGTATTATTGATGAGAGAAAGCAGAGGAGAATGATTTCTAATAGAGAATCAGCTCGTAGATCACGGATGAGAAAACAGAAGCACCTTGATGAGCTTTGGTCTCAGGTACTTCGGCTTCGCACTGAGAACCATAAACTGATAGACAAATTGAACCATGTATCCGATAGCCACGAAAAGGTTCTTTTGGAGAATGCCAGGCTCAAGGAAGAAGCCTCTGATCTTCGTCAGATGCTCAGTGACCTCCAAATTGGCAGCCCCTACACTCCTTGCTTGAGTAACCTCGAAGATATTCCCTGCAACAGTGCACATCTCAGAGCCGAATCTTCATCATGCCAGTCCATCGCCAACTCCATAGATAACCTACTCCATTGA

>ClabZIP25

ATGTTCTATTCCGAAGAAGAAGAAGTCGTCGGGTTTCCTGGGCCGGCTCAGGAACCTAGGTTCACTCCGGCGAAAATCCAGGAGCTTTGGTCTCTACTCGAAGATCCTACCAGGTCGAGCTCCGGTTCCCAAGGGTCGTGTCAAGCCGTATCTTTGATCGACGAGGAGAGGAGGCGGAAGCGAATGATATCGAACCGAGAGTCAGCGAGGCGGTCGAGGTTGCGGAAGAAGAGGCATTTGGAGAACTTGGCGGTTCAAACGGACCGGCTGAAGATGAAGAACCAGGAGCTGAAAAGGCAGCTAAATTTAGTAGTGAACCGTTGTTATATGGTAAGAAGACAGAATGAAAGATTATGGTCGGAATTTGTGGCGCTTCATGCACGGCTGTCGGACCTTTACCGGATTTCTGTTCCCATGCAAGAGAAGGAGAATTCATGCATGCAAATATCATTCAATTATTTCTCTTAG

>ClabZIP62

ATGGTAACCGAGGTATACTTTGCTTGTCAGTTCGATTGATTTCAAGTTGACTAGGATCTTATGTGAATTTGATCTCTTTGGTTGTCTGGGCTGGTGTTGTGTTCCATGACTCGAAAGATTGTGGCTAACATCCTGGTGATCCTGTAGTTTGGTACTTTCTTACTAATTCCATTGCTGTCCTGTTAGCTTCCTTTTCATGGCTGATACCTGATTATATCTTCTCTTCGAGGCCTCTTGCTATTTTTCCTGGCCATCGGTTGTTATGCATATATCTGAATCTCCTGCGAGTATATACATATAGTTGACTAGCTGAGCTTTTGGGGCTTTTGAATTTGGTGTCTTTAGACTTATCTTCATGATGGTTGTAGGGCGAGTGGACCAGGCAAGTTGAGAAAGAAATGAGAAGAAAGTGGCAAACGGGATGCCAGGTTTCGCTTCAGAGATGTCCATTTGTAACCAACTGTAAGTTCCTTCCCTTGTATTTTCTCTTGGCTGCTCCAAGCTGTGTGAAACTTTGAATCTCAGGCTTTTGTCCTGTCGTTCCTGTAATACTAAGAACATTAAAATCAATTTCTAGGCCGCAGTTTGCTTAGCTGCATTCAAACATAATGATTGGTTCAATTGCTAGTTAGGTTCAAGTTGTTGGACAGGAAAGTGTCAGTTCTTGGGCAGTTAATTGCAAGGTTCAGGTGGAAGATGGCTAATACCTTAACGGAATAGTGATGCAACGATTATTTATGATTGCTTTGAAGCATATCCTTTGTGGAACCCTAATTGTTATTGTGTATCACTAGCCTTGATTCCCCGTATTATTATTATTATTATTATGCCTTCGTTGTCAATAGCATCTTTGTTGCATATATTTATTTTCCTACTTTGGTTGTTCATTGCTCCTTTCTGCCTTTGGCTGTTGATTTTTCCCCCTAGCATCTTTAGTTTAGTACCTCTCTGTGTTTTTTTGGGCTAAGTTACAAGTTTGGTCTCTATCCTTGTGATTTTCAAAGCTAGAATTTCATCCTTGTGGTTCTTCACCTCTAAAACATTGGAAGGTACTGAAGTTTTGCCTTTTTTAGTCTCCACTATAAATTGTAACCAACAAGGTAACCACAATCTGTATCCAGATTACCTTAATCCAACTTGGTGGATGCAACTCCCTGCGGGGGCCACAGTTTTCTATTCACACCATTAAAGAAAAAGGAACACTTGTTTACCTTTCCAACCCGCCTCTACTAATAATTTCTACTGTTCTGGGTGGAAATTCTGCTAAGAGAAAATCACACGCACACACATTTGCTTGTGCATGCTTGTCCTCTTTTATTGGGTAGTAATATGTTGAACTCTTGATGCAGAAACCGTGCTTTAGAGTCCTATTAACAAGAGGCCAAACAGTTGCTTGTAACCTTCTATACTGGAACCTTTAGTAAGGTGAGTTTTATTTGATTGCTGGTTTTGTGTCTTCTCATAAGTTTCTCTACCTCTGTTTTCTTCCCCTATAATGAGTAAACAAACCTTGGAAGTTCATTTGTTTGTAATTCAATTATCCTAGTCTCTCACACGCACACACTCAGCACTCACATGACATTGTGGCGATTCAACTTGAAGTTCGCTCGTCATGGTCTTGACTCTTGTATAAGTTCTCAACGTTAATACCATGTAGACCTGTAATTTGTTTTAAATGATGACTTCACAGTCATTACATTAAAAGATCCATTGGAAATTTTAGAATACATGAACAGTGTTGTCCTTTTCAGTCATACTGCCACATTACTTCCCATACAGTGATGCCCTTGGTTTCCTTACCTTGCAGTATTACAGAGAACCTCAAAGTTTATGCCATGACTGATGTCAGTCCAAGGACTGATATTTCCACTGATGTCGACACCGATGAAAAAAACCAGAGGGTAATATGTCTTTCATATTCAACCGAAGTGCTATCTTTTACATCTTTTTTCATTTACATGATTCAGATTATTACACTTTATGTAGGGTATAGGATTTTGTTTAGGTTGTAACTGTTGGCACAATTGATTTTTCTTTGAACTGCATAGTTTTATTAACACTCCGATTCATCTTTAAGATGTATTTCTACTCTTATATAAATATATATATATATTTTTCCTCCCACATTTACAGCTCGATATGCTTCAACGGAATGTTGTGGCTTCTGATTCCAGTGATAGAACAAAAGATAAGTCTGATCAGAAGGTTGTACCCATTCATTAGTTATGTAATTGATATCCAAGATACATCTTCTTGGTTGTGATGACCCTGTGTGACGAATATGGATTATTATTTCACTTTTCAGACTCTACGTAGGCTTGCACAGAATCGTGAAGCTGCCAGAAAAAGCCGACTGCGAAAAAAAGTATGGCTCCATATTTTGTCTCCTGTTTTTATTACAATTCTGGCACGTTGTTGTCATTGATATTGTTTAGTTCAGAAAAGAAGCTTTTAATTAATTAAGTACAGAAAAATTTAATGTAGAAAGGCATCTCATTAAAGTGGAGGACTACAATCTCCATCTGAGGAAAAGACGAAAATGATAAATTTTACCTAAATAACTTCACAGATCCTAAATGTCATGTGAAAGCTGAAGCAGAAGAATGCACGTGAACTTAATCTTAATTATCACTGTTGATGAAATTAATAGTTGGTTTAGAAGCTTTCAGTTTTAGTACTACTACCATGTTGTCCTATACTCTCTATCAAATGATAACTTAAAGCATAGTCATTATGTTAAAATATCAAGTGAAGATTTTAGAGTTCTTGCACTGCTAGTCGCTTTCAGTCGAACTGTCACAGCACTTCCAATTAGTGAATTGTCAAACGGTCCTGCACCTTATCTTGAGTATTTGGATCACCTACTTTTGCTAAACTCCAGTAAAATGATTTTGTTTCTTGAAGTACTTGTGATTTCACATATCATTACAAAAAGGAAGGAAAAGGAGTATGTTAGGAACCAAGGTAGTATGGGCTACCTTTGTCCCACATTGGTTAGAATGGGATGACCAATGTGGTACTTAAGTGGCTTGGCTCTCCCACCATAATAGCTAGCTTTTGGGGTGTGGTTCTCCAAGGTGCTTAAGTAACAGAGTACTGTTACATGATTGACCTTCTGAACTCTCACATCTCTATCAGCATAAGCTTCATCTTTAGGGGCAAAATTCAACCCTTTGTCCTGTGCAAGATGAATGCCTCCTTCTTTATCCCTTCCTAAGTACACAGCTCCCTCTTCTTCATCATTAGATTTAAAACTTCATTCTTCCTATGATTCTTGTCTTGTAATTTTGGCTAAATTGATGCCTTTGAGGATATTTATTTGATCGAATGTCCTGATATCACGAGTGTCGACTTAATATTAGGATATTAGGACATCTGATAAGAGCTGTTTTCTACTACTTTAGTTCCCTTATTACATGTTTGCTCAACTTCTATCTAATCTTTATCACTTTATGTGAATGTTCCTTTTTCTGTGTATTTTTCGACCTTGTGAGTCTGTGATCCTTCATCATTTTTGTCTCCACGTATTATTCTTGGAGGCTGGTTTACTCGGTTGTTTTTCGAATGGTAGTGGCTTTCATACCTCTGGATTCTTTTTATGAAAGCTGGTCACAAGGTCACAAGGCCACAAAATGATGAAGGGTGTGGTCTATAACCTTTCTCCCTAACCTTATCAAGTAAATCTCAGTAATTAAGAGTACTTGGTTAGTTTAAAATAACAAGTATAATGAGTCACACTAAATCCAACTTGTTCCCTGATCTTACCTATATGTAGGCATATGTCCAACAGCTGGAGAGTAGTCGTTTAAAGTTGACCCAACTAGAGCAGGAGCTTCAGCGAGCACGGCAGCAGGTTGCCTGCATTTCCTTTTTATGATTATTTACTTGCTTATGTAATGTGTTTCTCTCTTGTTCTCTACCACTCAAGTTTCAACCTCTTCAAATTTCCTATAAATTGTGTCAATCTGTTTAGGGAATCTTCATATCAAGCTCAGGAGATCAGGCCCATTCGATGGCTGGAAATGGTATTTTTTTAATTATCTTGAATCGTAATATTTACTTTCAAGAGAACCTGAAATTTGAGATATGATTATTCGTCTGAGAGACATTGTAACCTGACATGATTATGAGCGTAACTCAGTTTCATTTATCTCCTGCATGATAATTGATACTTGTAAAGTACCCTGATTATGTAACCTTAACAACTTTTTGGCAGTAAGTGTGTTTTCTTTTAGCTATCTTTTCAGTTTGAAGTTGCATAGCATCAGCTACTTCACATTTGCAGGGGCCATAGCATTTGATGTGGAATATGCCCGTTGGTTGGAAGAACAAAACAAGCAGATCAATGAACTGAGAGCAGCAGTAAACTCTCATGCAAGTGATACTGAACTTCGAATGATTGTCGATGGCATATTGGCTCATTATGATGAGGTTTTTCGGCTAAAGGGGGTCGCTGCAAAGGCTGATGTTTTCCATTTATTGTCTGGCATGTGGAAAACACCTGCTGAAAGATGTTTTTTGTGGCTTGGTGGTTTCCGTTCATCAGAGCTCCTAAAGGTGTGCTCATTTTGGTGCTTCATACTTCCATTTGTGTGGGGCATTTCTGAAGAATCTCTATATTTGGGAACCGCGCATAGGAGGGAAAATACGAAGAGGATATATATGAACTTTTGCTAATTATGTATGCTATTTCATTCTTCAATTTCGCTTCTTAAGTTCATTTTCATGCTAGCAATGGCAATATAGAATATGCTATTGTTAGAGTGTCGATATGATTAAATTTACCATAATTCATCAGTTTAAGCTTTTAGACCAATTGGTGATTTAATATGGTATTAGAGCAGAAGGTCATGTGTTCGAACCCCTGTAATGTTATTTCCTCCTCAATTAATATTGATTTCCACTTGATGTGTCTTCTACACATTTTCAAGCCCACAAGTGAGAGAGTCTTAAAGTGTTGATATAATTGAATTTACCATAACCCATCACCTTAAGCTTTTGGATCAATTGGTGATTTAACAGTTATATGTTTGTGTTGAACTGCTTTAACTGATGTTTCGGTGTTAATACCAGTATGAAGTAGAGCTCTGAGTTTTGAGCATTTACTAAAACTGCCACTCTGTTGCAGCTTCTTGTCAATCAATTAGAGCCCCTCACAGAACAGCAGTTGATGGGCATATCCAACTTGCAGCAGTCTTCCCAACAGACTGAAGATGCATTGTCACAAGGCATGGAAGCGTTGCAACAATCGTTGGCTGAGACGTTATCGAGTGGATCTCTTGGCTCCTCGAATATGTCTGGCAATGTGGCAAATTATATGGGTCAAATGGCCACAGCCATGGGTAAACTTGGGACCCTTGAGGGGTTTATTCGCCAGGTATGCATATCACATTTGTTTTAAACCTACCCCCCACAGCACCCTTAAAGAGAAGAAAATACATTCTGCCATTTATCTTTCCATGAAGTTCTCAGTTGGGAACCCATCTTTTCTTGCATATGAAGTCCAAATCTAGATTTAGATCTCATATTCAAGCTCTGACTCCCATGCTTCCTAAAAATTTGAGTTCGAACTTAAATGGCCTTTACGTATCTTTTTATTCTATGGATCTGTACACTGACTAGGAACCCCTTTCTGCTTCACATTCACTTATGAGCCAAAGCTCTTTCCATTTCTAGTGCTATCCAAAGATTTTCATTATTCTTTTTTCCTTTTTCCCCTTTCCTTGTTTGGGATTCTTGGGTTAAAAAAAAATTAACAGGCTGACAATCTACGGCAACAAACACTGCAACAAATGCATCGGATTTTGACAATTCGTCAGTCAGCTCGTGCACTTCTTGCAATTCATGATTACTTCTCCAGACTACGAGCCCTTAGTTCCCTTTGGCTTGCTCGCCCAAAAGAGTGA

>ClabZIP26

ATGGAACTCCCCAATTCCACTAATCAAATGGCTTCCTCTTCCAACCCCACCACCCCATTTCGCGGTTCCTTCCACCGTCGAGCCCATTCCGAGGTCCATTTCCGGATTCCCGATGATCTCGATCTTGTCTCCGACCCTTTCGACGCCCCTTCTTCTGGATTTGGAGATCTGGGCTTCGAGGATGATCTCTTGTGCACTTTCATGGACATCGAGAAGATCGGATCCAAAATCGACAATGGGTCTTCGTCGAATCCTGAAGTGGCTGGCGGCGGTACTGCGGCGGAGAATGTTGAAGGGGAGAAAATCTCCCGCCCCAGGCACCGTCATAGCAATTCTGCTGATGGGTCTTCTATTATGGAGTCTATTGAGGCTAAGAAGGCCATGGATCCTGATAAGCTCGCTGAGTTGTGGACAATTGATCCTAAACGTGCCAAAAGGTTTTGTACTATTCTATTGTAATGTGCTTTAATTCATTTTTAGGTTCTGCAACTGCAATGTGTTTATGTAATGATCTCATCTTTGAATGTGTTGCTTGTCGATTGGACTTTTGATTCATGGCTGGAAGATCGAACCACTGAATGCTTTTTTTTTTTTCGCCCTTGGATGCTTTGAGTAAATGGGAATTAGTTTAATGTCTGATGGTTGGATGAAGTTTTGATGCATATATATAGAGAGAGAGTCTGTGATGATTAATTATGTCTTGGGGGGAATTAGCTATATATATGAGAGAGAGACTGATTTTGGACTTTTAGTTGGTTAATACGAGAGATTCTGGTGATATTAGATCTTACTAGGTTTTTGACAACCAGATATTGTAGGGCCACTTTGTGGGGTTCTTCCATATTTCGAAGTCTCTTTAACAATTTTTCCATTTGCCTCAATGACCAATAGGCCAACTTACGACAGTTCTTGAAATTCAATAATTAAAAATCAAGTCCCTACCTTATCGATCAAGGAATGAGAAAACATGTATTATCTTAATCTATACCTTCTAAATCAGATGTCTGCTTGGACATTCTAACCTTGTTTGACTGCTAATGCAACTATGTTGTGTGGTCTTTTGTTTTCCTGTTTCTAGTTTGTTAAAATATTTGCAACTGGTTTTATCCGTCCTTAGTACTTCATTTGCAGGATTTTGGCAAATAGACAGTCTGCTGCTCGGTCAAAAGAGAGAAAAGCTCGCTACATAATGGAACTTGAGAGAAAAGTTCAAAGCCTTCAGACCGAAGCGACAACACTCTCTGCACAGCTCACGTTGTACCAGGTTTTAACTTTTCACCAAACTCATTTTTGGCTCTTACAATGATCAAGGTCATATTACATATCTAAAGTCTGATAAAGTACACACAACATTTGGAAGGCAATTTTCTGCCTATTATTTGCTTATACAGTTCAGTGGGAACACTAGATGTCCCTTGCTATGAGATTTCTTAGCTTTTCTTGGTAAGAGATGTAATTGATTTGCATTAAAGTCACAGGAGTGTAACCCCCAAAAAGATTGATGAAGAAAAAGGAGAGAAATCATAGAAGTCCCAATGCAAGGTGGTGAGAAGAGGAACTTAGTACATTCTCCCCCAAATTATAAATAAAGATAAAAGAAGGAAAAAGAAAAATGCTCTACACTAATCTTTAAAAGTACTGTTGTTTCTGAAAGTGATTTTCTTATGTATATATATGTGTTTTTATTTATTATTAAAATTGTCTTCTTAAAATGAACAAATAAATTTTGTTTGGAGAAAAACTTGAGAGTTTGGTATTATTAAAACACCACATGCCATTTAAGTTGGAGGAATTTTCAATTTTGATTTTGTACGTTGAGTGTCCTTTTCTATTACTTCTGAAACGCTGCTTTTGGGATCAGGGGAGGCAATGGAGCCCTCCTCCGTCCAAGCTTTATTTAGTTATCATTGCTGTTACTATTATTATTGTCAAACGTATTCTTTTAAAAAAGTGAATTAATTGTTTTTCCGTTTTTGGAGAAGTTTAATTTCTTACCAAATTCTTGAAACTAAATTAAAATTTTGAAATTTTATTTTAAAATTCATTGTAAAATTTATATAAGAATAACTTACTGTGTCCTATGTATTCAAACTAATTATTTAGTCTCTGAATTTTTATATGTAACAACTTAGTTTTTAAATTTTAGTATAAACAATTTAGTTCATGTATTTTCAAAATTGTAACAATTTAATTTCTATGGTGAAAAATCCATCAAAATGAAGTGCCAATTTTTATTAGGTAACAATTAATAGTCTTTATAGATTACAAACACATTTGGTTCTTAATCAATTTATTGATCTAAATATGTAAGAAATCTCATTAAATTTTAATAATAATTTTCAAGATTGGGACTAAATCCTTAGTCCTTACAAATTTTAAAGCACATGTATTAAATTGTTACAACTTTGAAATTACAAGGACTAACTCATTACAAACTATGGTTTTAGAACTAAATTTTTACTTTGATGAATGTTTAGAGATCAAAGGTGTTATTAACTTTGATCTTTCCATTTCAAAAAAATATTTGAGAAGCTGAAAGTGGAAACCTTACAAATTATTTATTGCTTCTTACCTTTGATCTTTATATTTCTTTCTCAACTTCAAAACATGATTTTGACTTCTTTTATTTAACTTTTATGCCTTTGAAGCATTGAAATTTATTTTCTAGTTTAAAAGATGGGGAAATTTTTATTTGTTATCTTTGCATTTAGTGAAGTGAGTTTAATTTAACCTTAAAGTTTAAATTTTTATCAAATTAAATCTTTGCTTCATTGAGGGGTAAAATTAATATCCTCCATTAGTTGAGTTATTATTTTATTATTATATTTTAAAAAGGATTTATGACTAGTTGAGGGGCATTCTTGTGGTTAGATTGGGCTATTTTTGTAATTATTGGTTCTTCAGCTAGGTTAGATGTTTTTTTATTTTTGTTTGAGCATTTAAATGTCGAGGTTACGTTTTTCTTTACCTGCTTTTGATGAGATATTTGGGTGATTTGGTGTTTGTGGTTATTTGGGCTATTTGGTTGGATAGAAATAGCCTAACTCCTTAGGACATCTTCTCAACTAGAGACCCGTATAGAACCGCAAGGGTTTCTAGCTGATGTACACTTCTTAACTATTCCTCTTCTAAATTTTTAGATCTAAAACCTTATTCAGGGTGTAGGAGTGAGTGAGGTCTTGAGATGCTCAGAGTTGCTCTCTAGGTCTAGGGTTCTAGCCCCTAGGTGGAAAACTTAGGTCATGTTTGATAATGATTTCATTTATAATTTTCTATTTTATGATCTACACCTGGCTTGAATTTTGATAACTTTTAGAATTAGATAACAAAGTCAAACGGACTTGTGGGTGGAATTGATGTTTTTAAGCTAAATTTTCAAAAATCAAATAGTTACAAATAGGCCCTTGGTTATTTATAAAACTTAATAATATATAATTCTTTTTGTCTCCTATAATTACCTTCTAATACCCTGGTTAAGAAAGAAAAACCTTTGTTTTCCACAATAAAATGGACAAGGGCAACTAATCTGAAACTTGCTGAATGTATACTCCAATTGACATTATGATTTCAGCTTTTCCTGAAAAAGCAATAGGCGAAAGTTCTGATATGATTATAGAGTTTACTTTTTCTCTAAAATTAAAAATGTGAGTGTAGAGTTTCACTTTTATTTCATATATTTTGCCTTTTAGTGGCCTTCACTTAAAAAGGTTGTTGGTGCTTTGAGAGGAGGTAATGATGGAGACTTTTCTTCTTTCTCATGGAAAGAGGATACAAATGGGAATATACTTAAAGGTTTGTTGGTAAATAGAAAAAGTGAAAAAAAGTGGTAAGACTGAGATATGATCGCTGAGTGTACTAGTAGGAGTTTCCAAAAAGAGTTCTTAGTCCATCCATCACATCACATCACCTTACTTTTTCTTGTTTCTCTTTCTTTTCCCCTTCTTCCTTCTCTTTTTTAAGTTTGACCTCCACTTTCTCCTTATAAGCACCAAAATCTACCTTCACTGTGGGTTTTCTTCCCCTATCTCAGCTTCCAATTATAATTTCTTCATCTTACTCTCTCACAAATGACAAATGTCATTTCATAAAGGAACTAGAGTGATGACATGAATATTCCAGGAACATTGCAAAAATTATTAATTTAAATTTTGGATTCCAACTGCATGCTCAACATTCAATTTTGTTAACTTCAGCTTGTCTTGTGCTAATCCTAATTTTCCAAGTTATCACTGCTCGTTTAACTTTCAAATATTGTGATGTTTGGGAAGTATTGATATAGCATAGTCAGGAGTTGGTCATAAGCACATGAATGAAAACAGGTTTTACAGTAGTTTAATATGTATCTAATTAATTTAGCCTTGTTTGATAATCATTTGGTTTTGAAAATTAAGCTTCCAAATGCTACTTCTACAGATGGATTTTTTCATTTTGTTATCCACTTTTTAGGTATGTTTTCAAAATCAAAGCCAAATTTTAAATATTAGAAAAGCTAATTTTTAAAAACTTTAGTTTTTGCTTTTGGAATTTAGCTGTGAGTTTAAGTCTTTACTTAAAATTATTTTCAAGAAATTGTAAGAAAACAAGGACATTTTTCTAAAACAAAAAGCCAAATGGTTATCAAAGGCGCTTAAATTTTTTTGTGTAAAGCTACAGGAGGTTTTTCTGTTTTCTAATAAGCCCCCCACCCCAAAAAGACAATGAATACAAGGATTCTTATTTTTTTGCTTTGTTCATTTCATCTTTTCTTCATGTTCTTTCTATCAGAGAGATACCACGGGGCTTTCAACAGAAAACTCAGAGCTCAAACTTCGTTTACAAGCTATGGAACAGCAAGCTCATCTTCGCGATGGTATGCATCCACGTATACCCTGTCTATGTATTATTATTGACCTGACCCTCTTTGTTTAGCTTTTAGATGTCTGGATAATGTGATATTCACTTCTTCACGTCTGCTATTGGTTTTTCTTTCTTTATTCATAACTGCCTTGTGTCCAAGTAGGGTCTGTTTTAAATCCACCTACTTAGATGCTGCGTTATCCAGATTTTGATGATCTTGAATAAATGTTTGAATTTAGGAGCTATTGATGTCTATATTTGCACATTAATCTGGTCACCCTGATACTTTATCATCACCTTAGGTGCAGGAGAAAGTCTTGCTGTTGGACCAGCTTTTATTGTGCATTATAGTGATATTCTTGCTTTACGATTGAATGAAAAATAATGCAATAAAAATTTGTTTGACGACTTCTCGTAATATACAGCTCTAAATGAAGCTTTGAAAAAGGAAGTCGAGCGGCTCAAGATCGCAACTGGAGAAGTAATGACAGCTACAGATTCGTATAACTTTGGAATGTCGCAAGTTTCATATCCCCAATCTTGCTTCTCACACCAACCACAACCTGAGCGACACAACCCACAGAGGACGACGCAAGGGCCCCAAGTTCATCCATTCCATTCTAGTTTACCAAACCCACATCAGTCTTTGTTTGTTGCATCGCACCAGCCTCACGCCTTGACAGAAATGTTTCATCAAGATCCTATTAGCCGATTGCAGGGTCTCGACATCAGTAGCAGAGGCACAGAAATAAAGCCAGAAGGCTCCTCTATATCCGTCAGTGAAAGTAGCAGCACATTTTGA

>ClabZIP27

ATGGCGTCTCCTGTAGGAAGTTCATCTGGATCTCCGAGCTCCGACGAAGATCTGCGGCAAATCGTGGATCAGAGGAAGAGGAAGAGAATGATATCGAATCGAGAATCGGCTCGCCGATCTAGGATGCGAAAACAGAAGCAACTAGATGATCTGACGTCTCAGGTGGGCCAAATCAGAACAGAGAATGAACAAATCGCCGTCAATATCAATTTCACCAACCAACTTTATGTGAATCTAGAGGCGGAGAACTCTGTTCTCCGAGCTCAGATGGTGGAGCTCCGCCACAGATTGGACTCGCTTAACGAAATCATAAGCTTCATGAACTCAAGTACTAGAAATCTGTTTGATTCTGAGGACCATTACGAAGCTCCTGGCATTGATGGGTTTGTTGATTCTTGGGGATTCCCATTTCTCAACCAGCCAATCATGGCGGCTGGTGATTTGTTTATGTGTTGA

>ClabZIP28

ATGAACTCATCATCCACAAAATTTTACAATCCAGGAAGGATGGGATTGTATGAACCTCTCCACCATATTGGAATGTGGGGAGAAACCTTTAGAACCAGTGCCAATTTAGATGCACAATCCTCCTTTATTATTGAAGCTGATACCAAGCTTGAAACTCAGGTGATTGTTTCATAACGTTTTTGTTTCTTTTTAAAACTTTTAAAAAAAAATGTATTTGATATTTAATTTTTGTTATTTTTATTCCTTTAATAACCTTGGTTGAAAGACTACTTATATATTAATATAAGTTTTTTTTCTTTTTATATGAATATTAAACTAAAAAATCAGGTGACTGTTTGATAACGTTTTCTTTTGTCTGATTGAAAATAGGTTCTATAATAATATAAGTTATTTTTTACCTGTTTATATGAATGGTAATATTGTGTTTTTTCTAAGTAGGAAAATCACTCATGTATCTTAAAATTTAAAAAGTTATATGTATGTTTAAAATTAATTTGATTTTTAGTTTTATGTTTTAAACAATAATTTTTTTAGAAAATTATTATAAATAAAAAAATATTAAACTATTCACGAATATAATTTTTTTTTAATGTTTATTCGTGATAGACATTTATCGTTGTCTATATAGATAATGAAATTTTTCTATATTTATAAATAGTGGATCATTTTGTTATATTTGAAAACAATCATAATTTTTTTTTTTATTATTTACGTTTTTAGAAAAAGAGATTCGAGTTAAAATTTTATATTATCTATTTCAAATCTCAAGTTTTTTTTTTTAATTTTTAATTTTATATTTTAAATTATGTCATTAGATTTTAATTTTTTATAACATAAAAGTACACTTAACATAACTGTCATGTACACTTAAAATTTTTGACATGTAGAGGAAAATATGTATCAAAACTAAATGTGCTCATTAAGATTAAATTTACGTATTATATATATTTTTTGAATCTATTAGTAACCTTAAATATAACTTTTTTTTTTTTAATCTTAACTTTCTTTAGTCGGATGATGCTTCACTCGGCTCACTTGGAGATCCTCATGTATATGACCAAGATGATACCAAACGCATTGATAAGGTACCATACAAACATTTTAGTGTTTATAATTTATACTTAAAATTAATTTAACTTTTTAAGAGTTTGTGGTCAAATCTTTGTCTTTTCAAGTCAAATAGGACATAACTAAGTACTTAAAGATATTAGTAGTCTTTTTCTTAAGTCGAGAATTTAACTTTCACCCAACATTTTCAAGGTTAGATCTTTTAAAAATTATTATAAATGGAAAAAAATATTAAATTATTTGACTCGAATAGACATCTATCCGTGTCTATTTGATAAAGTGATAGATGTCTATCCAGGTCTATCGCAGTCTATCACAAATAAAAATAAAAATTTTACTATGTTTGTAGTTATTTTCAATCGTTTTTCTATTTTTTAAAACATTCCTTAATTTTTAACATAATTAAATAAATAAAATCTATTCTTAGTGTGAAATCTATTGAATACTTTATAAATCTTGATATTAAACTTGTAGTTTACTATTTTGTTATAAAACAAATACATGAATATAAGGATTTGAACACATCTAGTTTTAACTAGTTATGTTCAGTTGTCATATCTAAATATTTGTCCCAACTAATTTAAATATAGTTGAAATAACTAAATATATACATCATACTTTATGAGTCATATATTTTCAAAATTTTTCCGCTCATCATGTAAAACATTTTCTCATTTTAAAAAAATTAATGTGAATTTAAATACTTTTCCATAGAAATGTCTATGTATTCAATGAATAAAACCCGTATTCTATTTATTTATTTTTTTTTTTTTTTTGTGAGTACGAATAAAACCCGTATCTATTTCAAGTTATTAACTATTAGTTATAAAACTTAACGAAAAAAGTAAATAAATAAAATTATTATTATTCTTAAAAAGAAAAAAAACATAAAAAATGAAATAAACTTAGTCGTAGTTTAATAATCATATAATTTTTTAAAATGAAGGTCCTAAACATTGTTTTTGCGCTATCTATTTTCTACTAATGTTTTCAATATATAAGTAAACTTTTGAAAACTAAAAAAAATAGACTTGTATCAATAATTCTTTGGTTTTTTTAATTAAAAAAATTAAATCTATAAACACTTTTTACTGTTTAAATTTTTTGATATGCTATTTATTTTTTATTAATATTTTCAAAAACCAAACTATATTTTGAAGAAAAAAAAATTTTATGTTTGGACTAAAAATTCAATTTTTGCTTATAAGAAATTGAAAAGTAATAGATTTAATTTAAAAATGAAACGATTAGGAAACAAAATTGTGGTATTTAAAAAACTTGTTTTGATTTGAATTTGACTTGAAAATCAGATGGGTGAAGGTTAAATTGAAAGGTAAGAAGCATAAATTTCAATTAAAAAAAAAAAAAACCAAAAAATGAAATTATTAACAAAATGGAAATATAATAATTAGGATAAAATGTTTTTTAGTGGGATAGTTCAAATTTGTGGGAAAATACGAAGTAATTTCTTCTCCATCAAGAAAAAGAAGAATTATACTGAAAACTCAATGGCAGATTCAAAGACGGCTGGCACAAAATCGGGAAGCAGCTCGCAAAAGCCGGCTGCGGAAAAAGGTACGAACCAAAATTGTAGCATTCAATTTCCAAGTTTCAATTGCAATCACAAAACAGCGTTAATAGACTACTAATTTCAAACCCCTTTTGCAAATGCAGGCCTATATTAAGCAATTGGAAACAAGCCGTGTAAAACTCATTCAATTGGAGCAAGAGCTTGAAAAGGCAAGACAACAAGTACGAACAATAATTGCTTCTAATTGATTCACTTTCTTTAGCCCTTCATTCTACTTTTTACGGAGCATTATGTATTTGTTTCATTTCTTTTCCTCTAATTTAATGTCACGCCCTGTCTTTTTCTTCAGGGTCTCTTGGCTGGATCCCGATTTGACAATAACCAGTTGGGTTTATCAGGAACCACAAATTCAGGTTGGTTTTCTGGTGACTAATCGTTCTTTGTTTTTACCCTCTTTTTAACCTTTGATGCCCCCGTGTTTTGTCAATATTATACATCCTGTATTGCTTGGATTTCGTTGTCTGATTACTGAAAGATGGGAATTGAAAGTTACAAGATGAGAAATTAATTTGAGAGGTTAAATTTAGGGGAAGTGATCAGTGGCTAAAACTTTTGCAGGTCAAGTGTTTGTCGAATTGTCAACAATGAATATAAAGTCCAAAGTCTTAACCCAACATGTGGGTCATTGTAAACTGTAAAGGTTAGGACGTGTCTGAACGTTTTTGAGTCCTGGACGTAAAGATTATCCGACTTATTGATGTGTGGTAAAGGCAGTTTCTTCCATTGGATGTGGTTCCATGTTTCATCACTGCTTTAACAAAAGGCATTTTCCTTCTTTTTTGCTACTTTTCATGTTTTTATATCAAGATTGACTGGTCTTCCTTTCTTACCACTTCAATAATTTTCCTTGAGGATGACCCGTTTTATACGGCATCACATCTGTTCGACATAGATCCTCTTGTAGATGTATGGTATGGTGAAAAAAGGTTTGGTGCCTGAAAAGCTAGTTATGTAGGATGCCTTGGTTTTCAGAGTTTGGATATTTTTGGATGTGTCCTTCAAAGTACTAAGTTGAAGTCTCCAAATGGGAAACTTAGAGTCTGTTTGAATGACTTTCTTAAGTGCTTTAAAACTTTTCTTTTTCACTTAAAAACATTTTTGAATACTTTAAAAGTCATTTCAAACAGGTTCTTAATGAATTAAAATCCTTGTTTATCTCCCAATGCCTTTGAGGGTTGAAATGCGGAGTTGTAAGCATGCAAGGAGTGAGATACAATACCCCAGTTATGGAAACAAAAACAAAACAAAAATGCTGGTGACGTTGTAAGGAAGAGAACACTTAATGGATTTGTCTGTTCCACATACAGCTTTTTTTGTCTACATGTATAACTCTGCAAATTCATTTCAGAAGCCTCATTGACTGAAGTTATGACCTATTTACCTTAACTCTTACCAATATTCTTCTTTTTATTTGCAATCTACCTTAATCTTCACCCTTCTTATCTTCTGAATATATCCTCACTATTTGTTTTGTAGGCATCTCCGCATTTGAATCAGAGTATGAGCAATGGGTGGAGGAGCAGAACAGGCAGATTTGTGATCTGAGGACTGCTGTGCATGCTGATATTACTGATATCGAGCTTCGAATACTTGTCGAAAATGCAATGAGACACTACTTTAAATTTTTTCGCATGAAAGCTAAAGCTGCAAAAGCCGATGTCTCTTACATAATGTCAGGCATGTGGAAAACATCGGCCGAAAGACTTTTCTTATGGATTGGAGGATTTCGACCTTCAGAACTCCTCAAGGTATGTTCTTATTTAATGTGAGTGGCGAATCATAGTAAAATGTTGACATCAAAGCAAGAGTAAATCTTCCAGTCAGACAAGACTTTAGGTCGAGTTTGAATCGCAAACATGGCTTAATTCCCTATTGTATGTAGCTCCATGGACCACTCCCCCTTGAAAGAATGAAGATTAGAGCTTTAGATATACTCAGTATTCTTGTTTATTTCATCTATTGTAGTGAAATCTATGTTAGAGACATATGTTTTAAGGTATTCTTTGGATACTTCTTGACAATCATGGCCTCAAAAGTCCCACATTTTGACATTTTTTTATTGTTGAAAATCACTTGATTTCTATGTATATATTAGGTCCACAATTCTCCACCATTCCTTGATCCTACAATATTTGAAGTCAGGAAATTCAGAAAGCCTTTCGTTATTCAAGAATGAGAGTACTAAGTGGATTTATATGAATTCGTGAACACACTTAGTTTAATGACATTGACAAAGTTATGTGATATGCAGGTTTTGATACCTCAGCTGGAGACATTAACCGAACAACAAATTTCGGAAACTGGTAGCCTTAGGAAATCTTGTCTACAAGCAGAAGATGCTCTTAGACAAGGTATGGAAAAACTACAACAAAATCTATTTGAGAGCGTAGTGGCTGGTCAGCTGGGTGAAGGAAGTCATCCTCTACAAATGACTGCTGCAATGGAGAGATTAGAAGCGCTCATTAGCTTTGTGAATCAGGTAACATATTTCTTGAACTCAAGATCATAAGTCAAATGAGATATGATGTGTTCTTTTATTAACATTCTCCTTGGTCACTTAAACCTTTCATGTCCTAAGTCCAAACCATGTATGTTAGAGAGCGTTATCACCTCGATCGTTCATCCTCTAAACGTTTAGAACATGATTCATGAGTTCAAGGACCTGTAGCATCTAGATTATAACTTCTATAGATGTTCTTAGCAAGAAATATTTCAATATCTGAAAGGCATTATAGGGCTAAGATTTTCAAGGTTTAGCCGAAGCTTAGCTCATAACCCTACTAAGCTGCATTTGGTACATCTAGACTTATGTCTGTCTTCTTGAGCACTTCATGAAACATCATGTACTTTCTTTCCTTTTTTTTTTTTTTAGTTCTCTCTTTTCTAATACCCAAATAGGCTGACCATCTACGACAGGAAACATTGCAACAGATGTACAAAATTCTATCGACTCGACAATCTGCTCAAGGCCTTCTTATTCTTGGAGAGTTTTTCCAACGACTCCGAGCATTGAGCTCACTTTGGGCTAATCGTCCTTGTGAGCCTGCATAG

>ClabZIP29

ATGAATCCACCATCCTTAAGCAGTAACTCAACTTCTGATGAAGCAGAAGACCAACAACTAAGCCTCATCAATGAGAGGAAACAGAGGAGGATGATATCTAATAGAGAGTCTGCACGTCGGTCGCGAATGCGCAAGCAGAAACATTTGGATGAGCTGTGGTCTCAAGTCCTTTGGCTCCGGAATGAAAACCACCAGCTAATAGATAAGTTGAACCAAGTCTCTGAGTGTCATGATCGGGCTCTACAGGAGAATGCTCAGCTTAAGGAGGAGGCATCTGAACTTCGTCAAATGCTTACAGACTTCCAACTCCATAATCCTTACCTACCTTGA

>ClabZIP30

ATGGATCTTCCAAGTGAAATCCCAGAAGAAACCCAGTTATTATTAGGAGCTGAAGAAAAGTACTTTTGGACGATTGAAAGGAATGATCTTCAAAACATGATCTCTAGTGATCAGTTTTGTCGTCCAACAAGTAATTACAACAATAATAACAACAGGAGAAGCGCGGAAATTATGGTGGATGAAAGGAAGCAGAGGCGGATGATATCGAACCGAGAATCGGCTCGAAGGTCCCGAATGAGGAAGCAGAAGCATGTAAAGGAGCTTTGGTCTCAGCTGGCTCAGCTATGCACTCAGAACCATGACCTTGAGGAGAAGTTGAGGCATTTGATGGAGTCCCAGCAGCGCCTTCTCCAAGAGAATGCAAGCCTTAAGCAACAAGCTTCTGCTTTCCGCCAAATCCTTAGAGATATGGAACTTGAACAACTCGTCACCCAATTTTGA

>ClabZIP31

ATGCAGAGCTTTGAAAACCCAGAACAGTTCTATGCCCATTCTTCTTCTTCTTCTTCAATTTTTCTTCGGTAAGCCAGAATTGTTCTTCAATCTCTTTAATGGGTTTTGATTTTTCTTTTTTTCTTTTTGGTGGGGGTTTGATGTTATTTATTAACTTCCATGGCGGCTCTCCGTTTTTGTTATTAAAACAGGGGAGACGACAGTGGGCGATTCCATACGCGTTTTCTTCCGGATATTGAGGAACTTCAACAATCAGCTGCTGCTATTGCTGCTTTTCATCAAGATGATGCTGTTGATTTAAGCCCAAGTATTCTCTCTCCTCTTTTGTTATTCTTCTCTCTCTTAAACATTTCATCTGCTCTAACTACCTCCTGATTTTAAAATGAATTTTTCTTTATAGGCTCTGTTTTCAGTTTAAAATCGACCCACAATACAGCTTTTCCAATCTACCTACCATACGGGGTATTTCATTTTCATCTCCTTCTCTTCGTACTATTTTTCTATCAAGCTTTTGAGGGTTAATGGTTTGTGGGTTTTTTATTTTTTGAACGTTTGTGAAGAATACGGATGTGGGTTCAATAGGAAGAACAGGGTATTTGGACACAGGACAGGAATTGATGCGGTTGAAGAGGCTGGCTCCGCCGCAGTCGGTGGCGGTGACGGTGGCAGCTTCGTCATCGTTGGGAAACGGGAGCTTTGAGAACTGGGGAGAGTCAGCCATGGCAGATAATAGCCAGCAGACTGACACTTCTACAGACATCGACACTGATGAACGAAACCAGGTGTGCTTAAATTAGAAGAAGAAAGAAAGGAAATTAGAGAAAAAAAAAAACTTCAGGGTTTGAGATTATTATTCTCTTTTGTCTTTGCAGTTTCTTGTGGTGAAATAAGTTTTTGTGTATGTTTTTTTTTAGTTTCAAGGAGCTGCACATGGAGCTCTCATGGCGGTGGACTCCATGGATCAATCAAAACCAAAATCTGCAGATCAGAAGGTTCTATCTGAAAGCTCTTTCCTTTCTCTGTTTTCCATTTTCACAAATTCAGTGCTTGAAAACTTCAATGTGTAAGTAACCATTGAAATATTTGAACAGACTCTTCGTAGACTTGCTCAGAATCGAGAAGCAGCCAGGAAGAGTCGACTGAGGAAGAAAGTAATGCTTTTTCTTCCTTTCTTTGTTTCTCTTAATATTTACAAAAGCATATTCTTTGAGATCAGATGCTTCACTTAAAATTCTTCTAGTCTCTGTCTCCATGGGGAAATTGCCTTGGGATCGGAGGGGTGGGCCATGAGTATAATGCAAGAAGTGGGGAATGATATTCAGATAATGAAAAAGGCAATCATTTGTCACACATGAGCATATGTAGAAATATGGAAATTCTTTTGGATGAGTACCAAGTTTTTGCTTTTTGTATGATGAGCTGAAAGTTGTGTTCTGTCGTTGTCGTCGTTTAGGATTTTGATGTCTTGAGTTGATGAAGGGAATATATTTTGAACATTTATAGGTTTTGGTTTTGGATAGGCATATGTCCAGCAGCTGGAAAATAGTCGACAGAGGCTTGCACAGCTAGAGCAGGAGCTTCATCGAGCACGCCAACAGGTGTTCGGGTGACCGAGACTTCCCCAGTTTCATGTTATTTTTTTGTTCTTTTTTTTTTTTCGCCTCTTGTAAAGTCTTTTAAGAAGTTTATTGTGTTTGATTGATACCAGGGCATTTTTGTGGCTTCCGGGGCAGGAGATCATTGTGCATCAATGGCAGGAAATGGTACTTAAAGATGAATTGATTGTTACAAATTACAACATATGTTGGTTTCTAATGTGAACTCTGGGATGAATCAGTTGTGTTGTGTTGTAATATTGTCCCTAGGTGCATTGGCATTTGATCTCGACTATGCACGTTGGCTTGACGAGCATCAACGGCTGATCAATGATTTGAGAGCATCTGCAAATTCTCAGTTGGGGGACGACGAATTGAGATTTCTTGTTGATGGGGTGATGGCACATTATGACGAACTCTTCAAGCTGAAAAGTGTAGGTGCTAAGGCCGATGTATTCCACATTCTTTCGGGCATGTGGAAGACTCCTGCTGAGAGGTGTTTCATGTGGCTGGGAGGATTCCGTTCATCCGAGCTTCTAAAGGTAAAACTGTTAAATAAGCTCGGGATTACCTTTACTTTTGTTCGATACGTGTGTCGAGTTGTGTTTTAGTTTAACTCACTTCCTGCTCTTGGATTTGAAACCATTCAGATAGTAGGGAGCCATTTGGAGCCTCTAACAGATCAGCAGTTGATGGGTATCTGCAATTTGCAGCAATCTTCCCAACAAGCTGAAGATGCATTGTCGCAAGGAATTGAAGCTTTGCAACAATCTCTTGTAGAAACTCTTTCCACTGCTTCTTTAGGTCCAGCCAGTTCAGGAAATGTTGCTGATTACATGGGCCAAATGGCAATCGCGATGAGCAAGCTAACAACGCTCGAGAACTTTCTACATCAGGTAAAAGCTTCCACCTGTTCGATTCAACTTTTTCCATGTTTAAACCATGTGATAAACGAGTATGTATCCAGCTCTAATTGTGGAATCCTTCATGTCTTCTCTTTTAGGCGGATCTTCTGAGGCAGCAAACACTCCAACAGATGCATCGGATATTAACTACTCGTCAAGCTGCACGAGCGCTTCTTGTGATCAGCGATTACATTTCACGTCTTCGAGCTCTCAGTTCTCTGTGGTTAGCACGGCCGAGAGACTGA

>ClabZIP32

ATGATGGATTTGAACTTTACCTCCCGGAAGCCACCGCAGACGGCGCCGAGGATGGACATCGAACAGATGCCGGAGGCTCCACACCGTGGGTATCACCACCGCCGTTCCCACTCCGATACCTCCTTCCGCTTCGCTAATTTTGACGATCTCCTCCTCTTCGACCCTCCCGACATCGATTTATCATCTGCTCTTCCTTCTCCCTCGCCGTCACCGTCTCCGACGCCGTCAGGGGCTCGTATGGCCGTCGATTCCTTCAACTCGAGGTCCCCTGAAGACGTCTCTGACACAAAACCTAGGGCTGGAAACGGTAACTCCGCTTCCTTCTTCAGTTCTCACTACCGGAGCTTGTCGATGGACTCCGACTTCTTTGAGGGATTGGGGATGGCCGGAGATGGGAGTGACGGTGAAGTACTTGGCGGGAGAGTGACGGCGGGGGAGAAGAAGATGAGTCGCCATAGGCATAGCAATTCCATGGATGGGTCTTTGACCTCCTCGTTTGAAGCTAATTCCACCAAGAAAGCCATGGCACCTGATAAACTCGCTGAGCTTGCTTTGATGGACCCTAAAAGAGCTAAGAGGTTGTCGATTCGAATTTTCTCATGAATTATTGGGTTTCTGTCTCTTTCTTATATCATAGACATTGATGATGATTCTTTACTTTCGTTGAACTTTGAATGTTTCAAATTCTCTGCAATTAATCAAAAGTTAAGTCTGATTAAGTGACAAATGGGTTTGGGATTTATCTTCCATTTTAGGATTCTGGCAAATAGACAATCAGCTGCACGATCCAAGGAGAGGAAAATACGTTACACTAATGAACTAGAGAAGAAGGTGCAGATGCTTCAGTCAGAAGCCACTTCCCTTTCTGCTCAGGTCACAGTGTTACAGGTATGACTTCTATTCTTTTTGTAGCTTATGGGGTAAGAGAAAGATCATGAACAGGACTGAACTGGACAAAACATGAATCTTTTACCCATTCTTAATTTACAAGTCTTTTGGGTGGGCTGTAGCTATCAAGTGGACTTGTTTTGCTGATGGAGAACTGGTAACTTTTGACTTTTCAATTTTTGTTTTTCTTCTTGATGAATCAGATAAGATAGGAGAATGTTTTTTTTAGTTCTTCCTGGTCGAAGGCTTATACCTAAACCAATTAAGCTATGCTTAGTAATGTACTTTGGTGCAGAGAGATACCACTGGATTGACTACTGAGAACAGAGAACTCAAGCTAAGGTTGCAAGCCATGGAGCAGCAAGCCCATCTGAGGGATGGTGTGTGTGGTTCTTCATTCATAATCTTACTCTATACCTCATAGAAAAATGTGCACTTTTTTGGTTTCATTTTTCTTGAGAGTTCCATTCACACTGCAAGTTGAGATGTTTTATTCAAATGTTTGGTGACTTACAGCTTTGAATGAGACACTGAAGGAGGAAGTGCAGCGGCTTAAGATAGTTGCGGCCCAACTGCCTGTTGCAAATGGCAACTCATTCAACATTAGAGGAGTGCTACCTCCTCAATTCCCTCCTCTCCAAACATCATCATTGCTTCAATTTGGCAACTCCCAGAATCATCATCAACAGCCACAACTGCTTCACATGTCTCAGCCTGATGCTCGGGGAGGGTCTCCCCCGTCCCAGCCTCCCGGAGTTTAA

>ClabZIP33

ATGACCGTCGACGGCTTACTTCGTAATGGCTACAATTCCAATCCCACGGAATCGTCTATTCTCTTAGATGCACAGATTACTCTTGTTGATTCCCCTAACCCCTCTTCGCTTCCCATGAATACCACCACCACTACCACCACTAATTCCTCTGCTGTCATTGATAGTAACCATAATTCTTCCTCTGGTGCGCCGCCGCCCAAAACTGTGGATGATGTGTGGAGGGAGATTGTTTCTGGTGAGAGGAAGGAATTGAAGGAGGAGGTTGCTGACGAGATGATAACCCTCGAGGATTTTCTTTTGAAATCTGGAGCTGTGCCTGTTGAGGATGTTAAATTGCCGCAGACAGAGAGGCTGAGTGGAGGGGTTTTTTCGTTTGATCCAATTCCTTCCACTACATTTCAGGCATTGGATAAGGTTGAAGGATCCATTATTGGGTTTGCTAATGGGGTTGATTTGATTGGAAGTGGTGGAAGTGGGGGCAGAGGTAAAAGAGGGCGAGCTGCTTTGGAACCTTTAGATAAGGCTGCAGAGCAAAGACAGAGGAGGATGATCAAGAACAGGGAGTCTGCAGCAAGGTCTAGGGAACGGAAGCAGGTGACTGAATCTTCTCTTTATGAACAACTTTCATTCCCAAATACTTGTTTGATTCTATAATCTTTTTTGGTAATGTGTTTGATAGTCTACTTTTTGCTTTTTTGGTTTTGGGATCTCAGGCTTATCAAGTGGAATTAGAGTCGTTAGCTGTAAGATTAGAGGAAGAGAATGAGCGGCTTTTGAGGGAAAAGGTATTCCTTTTTTCTTTCGATATTCATATGAGAACATCTTTCTGA

>ClabZIP34

ATGGGGTCTCAGGCCGGAGGTGATCCAAATGGCAAACAATCACAATTCCAGCCATTGGTTCGTCAAAATTCACTGTATAGTCTCACACTGGATGAGGTTCAAAATCAGTTGGGTGATCTAGGAAAGCCTTTAATCAGCATGAACCTTGATGAGCTTCTTAAAAATGTGTGGACTGCTGAGGCCAACCAAACTGTTGGTAAAGACAATGAGGACAATAATGTCTTGGCCAATCAAACATCCCTACAACGTCAGGCTAGTCTTTCATTGAATGGTGCTCTGAGTAAAAAGACTGTCGATGAGGTGTGGAGAGACATTCAACAAAGCAAAGACAGTGAGGAGAAGAAGTCTCGTGAACGGCAACCTACGTTGGGAGAGATGACATTGGAAGATTTTCTGGTGAAAGCAGGGGTGGTTGCTGAGACATCGTCGAATAAAAAAGGTGCGGGTCCTGTTGTTGAAATTGACGCAAATATCACACCCCAGTTTCAACAAACGCAGTGGATGCCGTACCCTCAACCTCAGTATCAGTCTCAACAAGCAGCAATGATGGGGGTTTACATGTCAGGCCAGCCTATTCCCCAGCCACTGCATGTGGGAGGTGGTGCTGTGATGGATGTTCCATATGTAGACAACCAGTTGGCATTGCCAACTCCTTTGATGGGAGCTTTATCTGATACACAGACATCTGGAAGAAAAAGGGGAGCGCCTGAAGACATGATTGAGAAAACTGTCGAGCGTAGACAGAAGAGGATGATAAAGAATCGGGAATCTGCCGCTCGTTCACGAGCAAGGAAGCAGGTGATTTCTAAAATCTCAGGTTCTGTATTATCCAAAAAAGAAAAGAAAAACATAACAAAACAAAAAAGAGCTTTAGGTTTTGGGAAGTTAAAAGTTAGTAGTACTTCATTTTTTTTCTTTCTTGTTCCATTTTGCTGAGTTGCCACTTTGAACAGGCATATACCAATGAACTGGAGAATAAAGTTTCACGTTTGGAAGAGGAGAATGAAAGGCTCAGGAAACGGAAGGTTAGTTTTCTTTAAATACTGAATGTGGATGACTGTGGAATATTTTTCTTTTATAGCTGAACGGATTGACATTTGTCACCTCTCATTAG

>ClabZIP35

ATGGCTACTGAGGGCAGTCCCAGGACTGATATCTCAACAGATGGGGACACAGATGAAAAAAGCCGGAGGGTAATGTACTACTTCACATTCACATCACCCAGCCCCTTCATCCTCAGTGTATCTTTTGTTTATGATTTAAGAAAAAAAAAAGAAAAAGAAAGAAAGAAAGAAAAAAGAAAAAAAGAAAAAGAGATGCAGCCTGGATCATTCTTGTTCTCTATTGATTGTTGTCATTTCCGTAATCTAATTTCCTTTTATTTTGCAGCCTGATAGAGGCCAACTTACTCTTACTATGGCTTCTGATTCTAGTGACAGATCCAAGGATAAAACTGATCAGAAGGTTGTATTCGTTGGATCACTTTTCTAATTAATTCTTGAGTTCATCAAAATATGTATTGATGATATTTCACTAAGCAATGCAATAATCATTTGCAGACTCTTCGCCGGCTAGCACAAAATCGTGAGGCTGCAAGGAAAAGCCGATTGAGAAAAAAAGTATGGCTTAAAATGTGTACTTCAATTTTTTTAATCTATTATTTACTTATTTATTTTTAAATATCTGTGAGTGTCCTGGAAAATTTATACTTCATTTCATTATTGTAGTTTAGTAGCTTAGTTCAGCTTAGTTCAGTTGGATAATACATTCTCATTTTTGCAATATATCTTTAACATTTGGATTTAATTTTATATCTTTGAGGTGGATGCTAATTGACTAATGGCATTCCTCATGATATTTTGTGCTAGTATTACTTTCCCCTTTCTCGGTTATACAAATATCAATCAATAGCTTTAGCGTAGACCTTAACCATCTATTAATATGTTTCATTTTCTCCTTGTAGCTTGCTTTCACCAAAATGCTTATAACCTAGGTGTAAGCTGCTGAATAAATTTATCAATAATGACGTGGTTTAGTAAGGACACATTGTGAATACAACCTAATGAATCAATGATAAGGGGCATTTTATTGAGTGAACTTTTTTCTTCTTTGAGATAAAAAGGAGGATTCTGTTGGAAGATGCTCTGTGATATTTGGAATAGGTTCATTGGATGATATGCATTTTCTTTCGGATTTGACAACCTTCATTTTTAAAATTTTGTCCATCTTGTATCAACATATATATGAATTTTCCGTATTGAGTCAAATCCATGTGCAGGCATACGTTCAACAGCTAGAGAGCAGTCGATTAAAATTGACACAACTTGAGCAGGAGCTTCAGCGAGCACGACAGCAGGTTATATTTTCCATCAAATGAACATCCCGATATTCTCATCATCTGTTTTGTAATGATCACTCTCTTTACTTGTATTTCACTATGCTCATTTTTGTCATACCTCTACATAGGGAATTTTCATATCAAGCTCAGGTGATCAGACCCATTCAATGAGTGGAAATGGTATTCCATCTCGCACTCCTAATATTTATCTCTCAAATGTGTTGTTTAGAAGGTTCTAATTCTATAAATGTTAGATTCTGTTTTGTCTAGATCTGCCTTCAATTTGTAAGTGTAAAAGCTACATTCGATCAATTTACTCAAGATTTTCTTCAATTCGCTGCCTGTGTCACGTCCTGAAATGTTTGTTTGAGATGGTCGTTAATGAAGATGAGAAAAACGGGAGGAAGGAAAAAATGATAAAGCATTGTTTCTTCCAAAAAAAAAAAAAAGAAAAGAAAAAGAAAAATGCACGTTCAAGATGTTGTGCCCATTATCTGTTTAAAGAATAGAATCAAGTGTTTGTGATTTTTCCCTCTTATTTGGCCCTCTAACTGATGTATACATTACTATCTTTTTGCAGGGGCCATGGCATTTGATGTAGAATATGCTCGATGGCTTGAGGATCACAACCGTCAATTGAATGAATTGAGATCAGCTGTTAATTCTCATGCAAGTGACCCGGAACTGCGTATTGTTGTCGATGGCATCTTGGTTCATTATGATGAGCTCTTTAGGCTCAAGGGAAATGCAGCAAAGGCTGACGTTTTCCATTTATTATCAGGCATGTGGAAAACACCTGCAGAAAGATGTTTTCTTTGGCTCGGGGGTTTTCGTTCATCCGAGCTCCTAAAGGTGATTAATGCTTTCTCTCTATATATATAAAAAGAAAAGGGCAAAAATGAAGTGCTAGTTTATCCATTTTCAATGCTCTCTTTTAGTGTAAAAATTTACTTCCCCTCAGTTATTACTGTTTATCTTTTCTTGTTGCAAATTGGAAACACCTTTTGTACTTCGTTTGGGAGGGTCCTTTTGTATTCTTTTGGATTATTTCATTCATCAATGAAATTGTTTCACATAAAGAAAAAAAAGGCAAAAAACTGGGATGGTGGTGGCAAAATATGCTAATGTGGACCGTGGTGGTTGGAGAAAAGGGTTTTGTAAGGCCATCTTATGAGAAAAGAATGGGATGATATGGATATGGTCATGTTTTAATATTATTTCAACTATTAAAACCAATATTAGAAAGATAGTACTGTGTTTTTCATCTAAGCATTTCTTTGTGATTGTAAGGAACACTCGATTCTGGTTATTCTATGACTCTCCTTGAGAATGATAATGTTGTTTATGTTTTGTCTTCATCTGAGCATTTCTCTATGTTCTTTATGCTTCAAGAAGGTATTAAGGCTTTCCTCATTAACATGATACTCCGATCAAATGGAATGATAATGTTGCAGCTTCTTGTAAATCAACTGGAGCCTCTAACAGAGCAGCAGTTGGTGGGAATAACAAACTTGCAGCAATCATCGCAGCAGGCAGAAGATGCATTATCACAAGGGATGGAGGCCTTGCAACAGTCCCTGGCAGAGACATTGTCGAGTGGAGCTCTCGGCTCCTCTGGTTCGTCAGGGAACGTTGCAAACTACATGGGTCAAATGGCCATGGCCATGGGTAAACTTGGGACCCTCGAGGGCTTTATACGCCAAGTAAGCCTTTCAACAATAGATATATTATATATGTATGTGTACTCCTTTTTACTTGTTTCCATTTTGGTAAGGTTTGAAACTTTGCTTCCTAGCTACTCCCCTTCCCTCCTATCCTTGAGTCGCTCCACTGCGTGCAGAGTAAGGGCCATTGTTTCATACTAACAGATTCCAAACTTGTTTTCTTTGTTACTAATCCAAGCATAGTTTATCATAGTTTAGTGGATAAGACACTCATTTTTATTCCAAAGGTTGATATGTTGCACACTTTCGAACTGTTGAAATTCAAAAGAAAAAAAATGTTGTTTTTTTACGTGACCACTTCCTTGATTCGTTTAGTCCTTAATGTATCATTTCCAAATGATCTGATTTTTGTTTTTCATATCGATTTTCTGTAATACGGTAATAAAGCAATCTGTTCTTGACCTAAACTGGAAATTATCATATGAAATTTAAGCATCCTGCCCCTTTGTTGAATCTTACAAATTATAGGTAACTGATTCAAGATCCAGAACCATGTTCCATTTTCACATCATTAATGTTTTAGCATTTGAAGAACTCCACGGCTGTGTGATCCCTTTTCTAAGCACACATTTATGTGCTTGTACTTTGCAATGCCCTTTACGATTCCGTCTCGTGCACTTACAGTTTATCGAAATGACTATTTTTGTTCTTCCTGCTCTGCATTATACTTGGGTTCTCAAATTCCAGGCTGACAACTTGCGACAACAGACACTTCAACAAATGCACCGTATATTGACAACTCGTCAGTCTGCTCGTGCTCTTCTTGCCATACATGACTACTTCTCCAGGCTACGAGCTCTTAGTTCGCTCTGGCTTGCTCGTCCGAGAGAGTAA

>ClabZIP36

ATGAAGCGTAGCGCCTCCGAGTTGGCTCTTGAAGAGTTCCTCAAAAAAGCCGCCATCATCTCTCCCGACGACGACGTTATCGACCCCGACGACGAGGACGTTTTCAAAATCGAACAACAACAAATCATCAGAAGCCCAAAAAGAGGCAGAAACTTTCAGGATTCCGCCGATGCCACCTGCTTCTTCGGCGATATTGATTTCAGCTGTTTCCTCGTCAAAAATAATCGGGTCCGTTTTAATCTTTCTTTATAAATTCGTTACTTAATTACAATTTCTAACATCAAATGCATCTTGAGCTACGTTCATATATATTTTTTTTAACCAACAAAGTTTGTTAGGTAACTTTAGTTCCAAAAGATAGGTGCAGTTAAGATGAAACTTAAATTTTGTGATGATGGAATCGCTGCTAATTTTATTGTTGCTGTTTTTTTCGATGGAAAATTGGTAAGTAGGATTGGAATATTTTATTTAGTTTCATGAATTTAAATATTCACGTAAGGTTTTTGTTTAATGCATGAATAATTGAATATTTTAAAGTATAAAATTACAATTTTCTTAGGCTCATGCAGCTTATTTTTTATTTTTTATTTATAAATAAATAAATAAATAAATAAAATTTTAAAATAAATAAAATTGAAAGTTGCAGGAGATAATGGACGCAATTGTTAACTGTGGAGGTGGGCTGGCGGAGGCTCCTTTATGGTCACAGAATCTCACTCCTAAGCATTCAAGCTTTTCAGCCACTATAGATTCCCAGTCTTCTATTGGTATGACCATTATATATTTTTTTTATATATATATAGAATTTTTAATTTCAGCTTTTATATGAATTTTAATTATTGATCATGTTGTGGTTTCTTTTAAAGCATATTATTTCATAAATACGAAATGATGGGTGTTCGTATCTAAAAAATTATTCTTTATTAAGAGGAAATGTAAATTTTGAGTGATAGAATTATTTCAAGTTCAATATTAAAATTTAAGATTATGTTTCTATTAAGATTAAAATAAAAGTGATTTTGCTTAATTATGAATGATACCTTACATTTTAAGAACTTGTTATATGTTTTAGGTGATATTTATGTACGCACGAATAATTTTGTGGGTCAGATATGGTATTTATAATATGATTGAGAACTATGTACCTTTATTTATTTATTTATTATTTGTAAAGATTTTGAGTAGCACGTGCATTTTAAATATCAAAATGAAAAATGTACTTTTTTTAAAAAAAAAATAGTTAAGAAAAAAACATTAGACTCAATAAGTTATTAAAGATTGTGTAAATTAATTATTATCAATGTTAGATTATTGAATGACGTGCATCAAAATTTTAGTTAATTAATCAATTACAATTATTAACTAATTACGTGCTCTTTCTAAGAATAATATTATAACTACATTCAAGTGTCATAAAAATATAATGAATTAATTGTTATACTACATTTATCACATCCACTAAATATTAGACTAGATTAATAAATATTTGTTTATTAGCATGTAATTTATCATATCAATTTAGATATACATCTAACGACTTAAGTATAACTTAACAACGATTTAGACGTATATTCAGATCTTTCATGTTCAAAATTTTAAGTTAGAGAAGTTCAATTGGAAATGATAAAAATATACCGAGAGTGATAGTTCTATTCTTCATAATATTAAAAATAAATAAATAAAATACAAAAACAAAAACTTTGTGCCAAAAAAAGAATGCAAAAGTAAACATAATTTAACTAGCCTATAATATGGGTTAGTGACTAAAAAATGTATAATTCAACGGATCACAATATTATACTAAAAGAAAAAAAAAAACTCAAAAAAGTACAAAGCAAAGTCACCAAACAAAAGAAAAGAAAATCAGAACAAACAAAAAGGAAATATTGAAACTAAAGAACAATGTCACTTTTATTTTTGGGTGAGAGAGAAACTAGGAAGCTCTTTATATATGGAGTGAGCAACAAGCAACTCAAATTTCCTAAAATAGTTTTCCAACAAAACGTCTTTTTTGAACTCTATTTTTTTAGACTTATTCGATTTAGTCTCTATATTTTTAAATATTCAAATTTAATTTTTTTTATATTTAATAAAACCTAACATTAATCTCTATAGTAAAAATAAAAAAATTAGTTTCTATTCCTCTTTTATTAATCTTTCATTCCTAAACCTTTGTAGAATATTCCATTTATTTTTTCTTAAATGAAAGTTATTGTTTGAAAAAAAAAAAAAAGACTAATTTTAAGGTATACAACAAGGACTAAAGTGTAAAAAGTCAATGAAATTACCTAACCAAAAATTGTATTTTGGTTGTGTGTGAGATCAGTAAGGAAAGTTTGGATTGTGTGTAATAAAAGGGTTTGGTAATGGTCAGTGAGCAGCCCAACATCAGCCAGTAATTTGATGGGCAGAGAGCACCAAAGAGGTAACAATAGTGGGTCCTCTGAAGATCAATCTGATGATGAAATTGAAGCTGGCTCTTGTGAACAAAGCACTGACCCTCTTGCTTTGAAGAGAATCAGAAGGTCACCTTTTTTTCCCTTTTCTTTTAATATCATTCATTTTCATTAATTATTATTCATCTGACGATGTCTTCTCCTTGGCTTTCACTTGCAGAATGATCTCTAACAGGGACTCTGCTAGAAGGTCAAGAAGAAGAAAACAAGCTCATTTGGCTGAACTCGAGAACCAGGTATATCATTGGTTATTCAATGTATAAGTAAAGATAATTATCTCTAAATTGACGAAGTTGGTGTTGTTGACCAGCCAATCAGTTTTAGTGTCAGTTTGTGAGTGGAACTAGAATTAACGGCTAAACGAGACTTTAAGAATTTGGAGATATCGTTTGTGTTTGTTGGTCTAATAGGTCTTTTTCTTTTTCTCTCAGGTGAAACAATTGAAAGGAGAAAATGAAACCTTGTTCAACCAACTTTTAGATGCTAGTCAACAATATCGTGATGCCAATACGAACAACCGTGTGCTTAAATCAGACGTAGACGCATTGAGAGCTAAGGTACACACTCCAGTGTTTTCATAAGACTTGTTTGAAACAACTTCCTAAGTGTTTAAAAAAAGTATTCTTTTTAGGTTCAAAATAATATTTTTAAACACTTTGAAAATCATTCCAAACACAGATCTCTTATGGCATGTTTGAGAATAATTGTAAAATTGTTAAAATCACTCGAATAATGTTTTTAATCATTCAAAATCAATTAATAATATGAAAAATGATTTTAATTATTTAAAATCCCTCCGAAACATGCAAGAGATAGAATACTAATTTTTTCTTTTTTTGGTTTGGTCAGGTGAAGTTGGCGGAAGATACACTTGCTAGAGGCTCAATGACCTGTAGCTTGAATCAGCTGCTGCAGAGTCATTTGAGCACACCACAGCCACTCACGGCACTCAGACGAATGTCAAATGTCGCACCGCCGCTCGGTTTACCTGGAGACGAAGTTTCTTATTCCTGCGTGACAATGTCGGGGCAGAACCCGACCGTGGGGCTCCCGAACTCAGATATGCATATGAAAACAGGAATGGGTAGTGAGGCTGTTAGCTGTGTTTCAGGGATTTGGCCTAGAAATTAG

>ClabZIP37

ATGCAGGAGCAAGCCACGAGTTCAGCCGCTGCTAGTTCTCTGCCTTCCAGCAGTGAAAGATCCTCCAGCTCTGCTCTTCATCTCGAAGTTAAAGAAGGTCAATCTTCATCTTCTTTTCCGGAATATGAACAATTTTTCTATTTCGATCCTTCTAAAATCTGATCGTTTCTATTATTTTTTTTCTCTGTTTGGAGCTTGTTATTTGTTCGATATCCATTTGTAATTTAGGGAATTTCTTTTTCTGAGATTTTAAGTGTAATGGGTGTCTGGTGAGTCTGAATGGGGTGAGAAATTTAGGAATGGAGAGCGATGAGGAGATCCGAAGAGTGCCGGAGATAGGCGGTGAATCGGCGGGAACATCCGCTTCCGGGAGGGATACTGGTTCGGTTGCCGGTCCGGACCGGGTTCAAGTTTCTCGGGAGGGTCAAAGGAAAAGAGGGAGAAGTCCGGCTGATAAAGAAAGCAAGAGACTGAAGAGGTGAGGCAAAACGCGTGTCGTTTTGGTACTTTCAAGTCTTCCGAGTTTCAATTTTGTCTCATTTTAAGAGTTTTTAAAATTTTAAATAATTTATAAAAGTTCAAGCACTATTAGATATTTAATTAAAATATTTTATAAACAAACTTCCACAGGTTAAATTATAAAATATTCCTGAACTTTGACTAAGTTACTATGTCCTCTGTTTCGAAATGTTTTATTTTTAGCTAAAGTTTAAAATCTGTAAAAAAGCCTTTGAATTTTATAAATGTTTTGACAAATATATGAAAGTTATCATTTACAACTATATCGAAATTAAGAGATTACCATTTTATTCCCATCATCATATATTGCCGTAAATGATATTATAGAAAAATCAAATTTCAATGTGAAGAGCAGGTAGGAAACGTTTCAAAGGTTAGAGGCATAATTAGACTCTTTATGAAAAGATGTTTGGTATTATATAACTTTTTTTTTTTTTTTTTTTGGATTGAGCTTTTTGATTGTTGAAGCCATTGAGGTTTTGAAGCTATTTATTTTATTTTGAATAAAGATTGCTGAGGAATAGAGTATCGGCACAGCAAGCGAGGGAGAGAAAAAAGGCATATTTGAATGACTTAGAGATAAGGGTGAAGGATTTGGAGAAGAAGAACTCAGAACTTGAAGAAAGGCTTTCCACTTTACAAAATGAGAATCAGATGCTTAGACAAGTACGTTTAATTACTCTTATCATTCCTTCCATTATCATTAATTTATTCCATACTTTTGTTCTTAACTTCAAGTTGGAAAAATGATGGGCTTTTTATGAATAGGCCCAATCACGAATGGGCCAAATATCCAATTTTTTCTTTCCTAACCCGAAATTTTTTTTGATTGCTAATTGTAGATTTTGAAGAACACAACGGCAAGTAGGAGAAGTGGTGAGTGA

>ClabZIP38

ATGGATTCTGCGAGCGGAAATTCCTCTGGTTCCACTAGGCTTCAAAACTCAGGCTCCGAAGAGGATTTGCAGGTTCTGATGGATCAGAGGAAACGGAAGAGAATGCAATCGAATCGCGAATCGGCGAGACGATCTCGGATGAGGAAGCAACAGCATCTGGACGAATTGATGGCGCAAGTGACTCAATTAAGGAAGGATAACGCTCAGATTCTTTCAAATATCAACATTACATCGCAGCTTTATATGAACATCGAGGCCGATAACTCGGTTCTGAGAGCTCAAATGGCGGAATTGACTCAGAGATTGCAATCGCTCGAGGAGATCGCTAATTGCATCAACACCGGCAGCAGCAATGGCGGATTTGGAGAAACGGAGGAGGAAGCGTTTCAGATTCAGACAAATGTGGCCGCCGATAGCTTCATGAACTCGATGAATTTCCTGTATGTCAACCAGCCGATCATGGCCGCTGCTGATATCTTCCATTATTAA

>ClabZIP39

ATGAATTTCAAGGATTTTGGGAATGACCCATCTGCCGGAAACGGCGGAGGAGGCGGAAGACCTCCGGCGAACTATCCATTAGCGCGACAATCATCGATCTACTCGCTGACCTTCGATGAGTTTCAGAGCATGGGGAGTATAGGCAAGGATTTTGGGTCGATGAACATGGATGAGCTGTTGAAGAACATTTGGAGCGCTGAAGAAATGCAAACGATGGCATCTTCTGCTGCTGCTGTTGATAAAGAAGGGGCTGGTAGTGTTGGTCGTAGTGGTGGGTATTTGCAGAGACAGGGCTCCTTGACGCTGCCTCGGACACTCAGCCAGAAGAAGGTGGATGAGGTATGGAAGGACATAATCAATGAACATGCTAGTGCTAAAGATGGGGCTACTGTTGCTTCTAATTTGCAACAGAGGCAGCAAACTCTTGGGGAGATGACACTTGAAGAGTTTTTATTCAGAGCTGGAGTGGTGAGAGAGGATACTCAGGTGACTGCAAACCCCAACAATGGAGGGTTCTTTGGCAATAACACTGGTTTTGGAATTGGTTTTCAGCGGCAGGCTAAAGTTCCTGAGAATAATAATCATATTCCAATTCAATCGTCGAACTTATCGCTGAATGTCAATGGAGTCAGAACACATCAGCCGCAGCCAATTTTTCCAAAGCAGCCTGCTGTGACATATGGATCTCAGTTGGCTTTACCCAGTGATGGTCAGCTGGCTAGTCCAGGAATTAGAGGGGGAATTATGGGGATTGGAGATCAAGGGTTGAATACAAATTTGATGCAAGGCTCTGCACTGCAGGGTGGAAGAATGGGAGTGGTTAATATAGCAGCTGCTCCGTTACCTATTGCAACAGAATCGCCAAGGGACCAACTATCATCTGATGGGATTGGTAAAAGCAATGGTGATACCTCGTCTGTGTCTCCTGTGCCTTACGCGCTTAATGGTGGAATCAGGGGGAGGAGAAGTAACGGAATTGTCGACAAGGTTGTCGAGAGGAGGCAACGGAGAATGATTAAAAACAGAGAGTCCGCTGCAAGATCGCGGGCTCGAAAGCAGGTGATCTGTTGTTCACCCTAACTTTGTTAACTGTTCTCTAAACCATATTACTCGGATTCTTGTTGACTTGCTTCTATGTTGCCCTGTGCACCTGGATTTGTGAAAAATAGTTATTAACAATGTGGAAAACCACCGTTTGATGCATTTGTCCTCCATTACTTAAGCATGCCATACCATATGCTTCATCCATGTGCTGATCTATTCTTTATTAGATGCACTCATAAATGATATATTAAATCCTGGTTCATATTTCTCTTTAGTTCTGGCCGGCCCATTTAGCAAATGTAGTTATGACGTTTCATTCTACATTTCCAAATTGTTCTCCTTGAAACTCGAGAGATATTTTATTGCTGCTAAGTATTATAAATATAGGATTTAAAAGGATTTGATGTCTTAAGGCATCCTAAGAGGAAAAACTGGGCCATATAGTTTTGATACAACTTTAATTGTTGCTGGCTGACCACCATGGGACTGTCTTTTAACTTGTAATTTTAGACTAGTGGCCATCTATCAAAAACACTGAATGGCCAAATGCCTGTGTTGACCAAAAAATGAATCTGCAAGACTCTAACCTTCTGCCCCTAGATTTATTTCTTTGGGCGTGCTGCATTACAAAATTTCACCGAGTCTATATGATGTTTTAAGCTATGATAGAATTTTTTAAACCACATATCATGCTGAAATTCGTAACTCAAGATTGATGTTAGTTTTAGATTGATGGAGATCATTTGTCCATGATTCTTTCTTTGCTCCCACTAGGCCTACACAATGGAATTGGAAGCAGAAGTTGCAAAGCTAAAAGAGGAGAACCAAGAACTCCGGCAAAAACAGGTAAGTGTCTCTAGCTCAAAATAGTGAGTTTGTTAATCAAATGATGGGCATCACCAATTCTCAATCATTACTTTGTGTGGTTGCTGCAGGCAGAAATCATGGAAATGCAGAAGAACCGGGTAAGCTTTGCTTATTGATATTTTCATGTACTAGTATGGCTGTCCATATGCATATTACCATTACTATCTAGGAAAAAACCTTTATTCTTAACTTTTATCACCTATTTTCTATTTCTACGAGTCATTCTTGCTTTAAGGGTAGATCTATACGCAACTAAAAGAAAGTCAAAGTCCTGACAGTTTCCTTTTCCCTTTTATTTCGGAAGTCTTTTTCATCAGCTGTATATTATAATGCCTCAATGGGGATGGAACTTTATGTAGAACATAATATTTTTTATATAGTCATACTTGCTTATTTTGCACCTCGCGTTAAGCTCTTAACATTTTCATCTAGGTCCTTAACTGGTGAGGACTGCTGGTTAATTCATTGTGAAAACATTCTCTCTACCTCTCCTCATCAAACCATTTTTCTATCTTTGACTCCTCATGATAATTGATGAACATTCTTTTTGATACTGCAATCTTCTCCTTGTGAGAAACGTCTGGTTTATATTGTTCTTGATGGCCTTTCGTTATAGGCATTGGAAGTAATGGATAAGCAACAAGGAATAAAGAAGCGATGCTTAAGACGAACGCAGACTGGCCCATGGTGATTTTGGAGGATGTTATCTGATATCAGTCTTAAAAAATAACGTCATAGATGGAGGTGTACATATAATTGTTCTGCTGTAGGTGCCGTGTAGCATATGTCTCAAGGCGTCTTGCTTGCAGAGTATCGTATAACGTATTTGCTTCGAAGTGGATCATCAAGAGTTCAGTAGCTTTTGAGTGGAAGCAAAACACATAG

>ClabZIP40

ATGGCAATGCTGAATATGGCGGAAGATGGAGTTGGGTTTCAGTGTGAGGTACTTGAGAATGATCATGAGTTCACTGCCACTGAAATCGAAGAGCTTCTGTCACTCTTTCTAGCCAACGACGGGCCCCCAAGTCCCGGTTCCGATTCTCAGGGTTCGATGCGAACCTCGGCAACTTGTTCGACCAATGACGACGAGAGGAAGCTGCGGCGGATGATATCAAACCGGGAGTCGGCCCGGCGATCGCGGTGGAGGAAGAAGAGGCATTTGGAAGATCTGACTAACGAGGTGAATCGGTTGATGATCCAGAACCGGGAATTGAAGGAACGGCTCGGCCGGGTTTTGAACCACCGCCATGTGGTTTTGAGAGAAAATGATTGGTTGTGGATGGAGTCCGTGGGCCTTCGGGCCAGACTTTCGGATCTTTGCCGGATCTTGGCCGTCATTCAATAG

>ClabZIP41

ATGCAATCGAATCGTGAATCCGCTCGACGATCGCGGATGAAAAAGCAAAAGCAATTTCAAGATCTAACAAGCGAAGTGAGACGTTTACAGATTGTGAACAGTCGGATTGTAGAAAGTGTCAACAGCAGGGAACAAGCGAGGATTGAAATTGAAACAATGAATAATCTTTTGAGAGTCGAAGCAATGGAAACGACGTACCGACTCAAGGCCTTAGATTTGGTGCTTCAAATCGTCGACAAAGCGAATGCTCTTGCAGTTGGTGTTCGTGATCCTCTGTTGGAACCTTGGCAACTCACCAGCAAAGGCAGCTGCCGCCGGCGGCGGCGGATTACGATACGATTCTTTTTTAATGCAGTTTTATTTTTAACATGTAGAATCTTCTAA

>ClabZIP42

ATGCATACTTTCTTCTCATCGGAAGACCTCTCCGATAACTCCTTCTGGCCGCCACAGCCGGCGGCGTCGTCTTCGCCGCCGTCTCATTCTCCTTTTCGGTCGCCGGATCCTTCCTTGACGATGAACCGAAGCGCCTCTGAGTGGGCGTTCGAGAGGTTTCTCGAGGAAGTTTCGGCGTTGCCTGTGAATAGTTGCCCTTCGACTACTTCCGATCGTGTGCCGGTTTCTCCCGTGGATGTGGCATCTCCTGCTTCTCAGTCTTCAACGTCGAAGCGTGATGAAGGTGATGATGAGATTGTGGAGATCAAGAAGGCGGATTGTGATCATGATCGTTCTCAGCCAATTCCGTCTTCGGATCCGTCGAAAATGGTTCGGAGTAGTTCTGATCGGTACCGTGTGTTTTTGAAGAATCAGCTTGATATGGCTTGCGCTGCTGTGGCTCTGTCGCGGGTGATTGTTTCTCTCTCTCTTTTGAAACTGATTCGTGTACTGTATTTTGATGAATTTGGGATTTGAAATGCTGAACGCTAATTGTGTTTTGAAATGAAATTGAGGAACTTCATTGGTTTCTTCTGGAAGAGAACTTGCTTTAGCCATTTTTTGTGTTCGGGATTGCAGGCTGCTTCTTTGGAGCCCAAAGGTCCAGTTCAGCCAGCCGATCACCGTGGGCAAAGTTCGAACGCATTCCAATTTGGTATGCAAGCTCCGGGCCAAGGTAGCTTCCGCTCATCCAGTCTACGGGTTTTATTATATTCATTATTTATCGTTGTCAAGCTCATCAAGTAGTTATCTAGTTATTGTTCCTATGGCTTTCGTTGGCCGTTTTCCATTTGGAGTGGACAGAAGTATCGAGCAGCCTCTTGTATATGAGAAGATCTAATACGTGAAGTCAAATTTGTGAAAAAAGCATGGAAAATTTCTTAATGTTGTTTTCCTGTTCTCTGTTGTGGGGAACTTCGATATATTGTTTTTGGCGGAGGGCGGTGTTATTTCTTTAGAAGCCAAATCCTTTGAACACTCCGACTCCGAAGTGTTGATTTTGTTTCATCAAGTTGAGTTTCCGTAATCATGAATAATGGTTTGAAGAGTTGCCGGTTGTAGTGCCATGGCAAAATGCATTGCAACCGGATTTTTCTCACTCCACTTTGGTAATTGAATGTCTTGACTCTTTTCGAGTTTTATAATTATCTCTATTTGATGGTGTTCATCTCCTGGTAGGCCAGTTCGATTTTTCTATTGATCTCCTGGCGTGCCTAAATTTTCCGTAATCATCTCGGTCTTATTATTTAAAACCCTTCATTTCTATCTTGTTAGGTCTCTAGCTATTGTTTTGACTCCATTTTGTTTTCCTTTTATTTGATAGGAATCAAAAACCATTCATTGGTATGATGAGTGGAATACATGGGGGGGTTCTATCGGATAACATTACAAAAAACACCTCCATTGATTACAAAAAATACCTCCATTTTTTTGGCTCTGCTTTTGGACAATGATTTTTGTAAGCCTCTTTTGTATTATTTCATTTTTCTCAATTTAAGGTCGACGTCTATTGAAAAAAAAAAAAAGAAGAAAGAAAGAAAAAAAAGCGTGCTCCAGATAATAGTCAATATATTTTCAGGGGATCTCATATTTTTACAAGTTAGATCCCTTTTCATGATGCATCTTGCCCCTTCTAACTCTCTTACCATTTAAATTATGTGCTATTAACTGCAATATCGCTTTTGCTCAACGGTGGCTGTAGGTTCTGATCGTGGCACTTCAACAAAAGAAAGTGAGGTTTCTGGTAGTCCACTTGGGATTCCATCCTTACCTACAATGCCTAAGAAACTAGGGGTACAACCTGCTCAAACAACGAGTGGATCATCCAGAGATGAGTCTGATGATGATGATCTTGAAGGAGACATTGAAAACATTGAGAATATGGATCCTGCTGATGCAAGACGTGCTAGGAGGTAATTTTGGTTTATTGTTCTGTGGACTTTGTTGCTCTTGCTTATGTCAAATTGTTAATCCTCTAAATTATCCAGCTGTTCATATTTCTGTTCATTGAGGACATGACTCAAGCAACGATCATTTGCTTTCTTATGCTCTTCTATTATGTTTTTCACCCGTTATAATGTTTAGAGAAGGCATTGTTGTTACATCTTCATTGATTATCTAGGTTCATCGTGTGCTCAACTTGTTGATCTAACTAGAGTCTCATGTCAAATCCCTTTCACAATCGCCTTGTACATTTGTGTAACAAAAGAAAAAAAAAGGAAGTTAAAGTGTCATATTCCGATCAAATATTTTGAAACATTGAATTCTGTAATTTTTACGAGTAGAGATATGTTAAGCATGTTTCACTTTACTTTGCATTTAAAAACAACTATCTTCTTTTATTGTTGCTACTATTGAAGAGTGTTTTTGTGATCTTTATAGGTTTTTGGGTAAGGAAGATATCTCATCTTCCATCCTGTCCTCTTAATTGTTTAGTGAATGTTCTTATCTTAATGGAAGAGGTTTCGTTTCTAATCAGTAAATAAATAAATTAGAGAGGAGAGAAAAAGAAGGCCCTATGGGAAAAATCAATCCTTCTACCCAATTTGTTGCTCGGTCCTAGCTTTACTCACTTGACATTGCTGGTGGCCTAGGATGTTGTCAAATAGAGAATCAGCAAGACGCTCCAGACGACGAAAACAAGCACACTTGAATGAACTCGAGACACAGGTACTGCAAAATGTTTACCCTTATATGTTCTCGGGTATTTTTCTTTCTTATTAAAACATTTTCTAAACGTGGTTCCTTTTCTAAAGGTTGGTCAACTTAGAGTTGAACACTCTACTTTGTTAAAGCGTCTCACAGATGTAAATCAAAAGTATGATGATGCTGCTGTTGACAATAGAATTTTGAAAGCTGATATCGAGACTTTAAGAGCGAAGGTTAGTCCTTTTTCTTTTTCCCCTCTCTCTCTCCCTTGTGGTAAGGAGTAAGGAAGGAATGGAAAACTTTCAAACAAAAAAAGGAAAATTAACATACAAGACCATAACAAGGACATAAAGGACTTTGACAATCTAATGAAAACAGAAACTAGGCATCTACGCTCTCCCATGCAGAATGATTTAGTTTTTGATGCGTGCCTCTTCTCATCTACCTTGGGTAATTCTTGATCCTTGAAGATTGATTTTCCTCCGGTTCCTTCACACCCTTATTTCTTGCACTCTGCTTTCTTCACGAACTCCTTTCTTTTCTTTAACTTTCCTCCAATCCTTTTTTTTCCTTCATTTCTTCCATTCAAGAGTACCAATTTCACAGTTTTTAGGCAGGAACCTGCTTTTGTCTCAGGGATCCCTTGATTTTGTCAATGTATCTCCAAGGGCTGGCCCTTTGATAGTGGTATGAAATTCCGACCTGGTTTAAGATCAAAGTGTGTGGGGAGCCATACTTAAATAAAAGCAATAACTCTCCTCCATAAAGCTGTCTTTTCCTCATGAAATCTCCTAATCCATTTAGCCGAGAGGGATTTATTTCTCTGTTGGATGCCAATGATGGCAAGACCACCTTCCTCAATTAACGAGAATGACTCTCCCATTTAATATGGTGAGAACCCGCTTTCTCCTTGCTGTCTTTCCATGGGACATTGCAGATCATTCTTTCAATTCTGTTTGAAACCTTGACCGGTATGAGGGTGAGAAGACCACCTTTAGAAATATATGAATGTCCTGATATGCTTTTCCATTATCTTTTCAATAATGGAGCCCAAAAAGTAGAAATCATAATGTTTAAAGAATAAGATTTTGAACTTCAGGAACCAAACACTTGAATAGAGTGGAAGAACCTAACAAAAATGCTGGTGATTCTTCATGATCAAGAGGTATTTATCTCAAAATTTTTTGTTGATTTTTTGTTTTCTTTTGGATAATAGAAACATTTCGTTGATGAATGAAATGAAAAAATAAACCCAACTCTAATAGGTGATTACAATAAAAGTCGCCAATTAGAGCATAAATAAGGTAAGCCGAAATGAGAAAAGGGTGCTTAGATTTGCACTAAAGAAAGTAGTAGAAAACACTAAATCCTTGAAATGATCAAAAGAGGAAAAAGAATCTTCTGAAAAGACAACTATTAAGCTCGTCCAAAGGTTCCAAAAGAAAGACAACATAAGAGCCAAGCAAAGTGTCTTCTTAGTACCAGCAAAAAAAATGACTGACCTAAAGTAAGGCAAGAAGATCAAAACTATTATTGGGGTAAGGAAAAGACCACCCAAAAGCTTCAAGAATAAGTTTCCAAAATCGAGATGTAATTGACCAATGTATAAAGATATGTGCAAGAGATTCAACATTCTGATGACACATATGGCACCAAGAAGGAGAGAGATGCATGAAAGACATACGTTGTTGCAATCTATTAGCAACATTGATGGCTCCAAGACTAAGCTCCCAAAGAAAAAAATTGATTTTCTTTGGGTAACAATCCTTCCAAATAACCGTATACAGATCTTTGAAATGAGGTTCCGCTGTTCTTACTAAATTAACTATAAGAGACTTGACAGTGAAACACATGGAAGAGTCAAGAAGCCAAGACCAAGGATCAGGTGGGTGACGAAGTTTGACTAAAAATAAAAGATGAGATAGAGCAACCCATTTAGTGATTTCCAATTTCGTAGGATTACGGCAAAAACGTGAATTCCAAAAGCCCAAATTTGCGCTCCACACAATAGCCACAGTAGCTTGAGGTTAGAGAGTAATACAAAAAAGACGAGGAAAAGCAGATGAAAAAATACCATAGTTGAGCCGGGGATCTTTGTTTTGTTTTTTTTTTTTTTTTTTTTGGTGCAGAATGACACTTACTTGATTGGTTGATGTGCCAATTCGGTATGCAACAAATCCCTCGACAATGCGATACAAATAATGATTGCACATGAATAACCTGTGTGGGTAGTACAAACGAGATTAGGTTTGCCATTTGGCTCCTCAATTAGAATTATGGGAAAATCGACTTAATTATTTTATTTAGGGGAGACAATTGTGGGGGTGTAGTTGTTGGTCCAGTATATGAAAGATGGTATTCCAACATCAGTAGGCGGTACGTTACACAACCAAGTGCAACATATAATGATTTGGGAAGGAATGTTTCTTATGTTTATGACTGCTTATAATCTGATATCCTATTTTTTTAAAAAATTTGTTTTCGAAAGTATTTCAAATCTCAGTGTACGTAATAAAAGGAACATAAAAATCTGATCACTATATACAAGCCTTGACATCTGGATAAAGGTGAGTCCTCTCAAGATGAGAATGATTATATTCAACAGGAAGATGACCATGTTCAGTATCTCAATTTCTCAGTTGAACATGGAGTAGAATATGAAGCTTCACTGTACACTGAACTGTGCACACCTATGTTCATGATGCAATCTCCGATGACATTTGGTTCGTTTTGACAATTCCTCCAATTTCCAATTTAGAATAGTCACTATGAACATACATCAATCCTCTCATACTCCATAGATGTTTCAAGATTAATATGTGACCTATCAAGTACATAATGAAGGTCAAACCTTAACTGGACAGGATCATCATAATCGTGGGCGTTCTAGGCTTGATCGAAGTGGAACATTTTCCACTAATGAATAAAAATAGCTTTTCTAAAACTAATTTCAAGGGCTCTCATCTAGTTAGTTGGGAGATGGTTGGGCGCCCTGTTAACCAGGGGGTTGGAGATTGGCAATTTAAGGGTTCGTAACAAAGCTCTATTGGCTAAGTGGCTTTGGTAGTTGCCCTTGAGTCAGAATCGTTGTGGAATCAAATCATATTGAGTAAGCATGGCAACCACCCTTTTGATTGAGTGGATAAAGGGGTTAAAGGCACTCATAGAAATCCGTAGGAAGAAATTTTGTTTGAGCTTCCCACTTTTCCCCATCTTGTTCGTTGTATTGTGGGGGATGGTAAGGATACCTTTCCTTGGTTTTATCATCTGTCTTCTTTAAAAAATTGCCCAATCTCTGATTTCTTGGTTTGGTTTGCAAACTTTGTGTCTTTTTCGTTCAGGTTCCGTCGGCATTTGTCCAATAGGAAAACGACAGAGGTTGCCTCTTTTCTTTCCTTACTAAAGGAGAGCTCTTTTAGGGCAGGGAGGAGGGATATTTGTGTTTGGAGCGGTGACCTTGTTGAAGGCTTGTCTTGTAAGTCTTTCTTCCGTTTGTTACTGGATCCCACCCTGGTTGGGGAGTTGGTTTTTGATGTGGTTTGGAGGATCAAGATTCCTTAGAATTTTAGAATTTATGATTCCCCCCACCCAATCAATACCACCAGAGGACAAAACCAGAGGGAAATGATCACATATGTAGTCCGATCCACACGATGGAAGGTCATAGCTCCAAATTTGACAAGGCAAGCATATGTGACAAGGAATTTGTCCAATAAGGAAAGATATTCTAAAATTGTTAAAATCACTTTTGTCATTTTCAAAATCATTTAGAAAATACTTTTAATCATTCAAAATCAATTTTAATAGCATGAAATTGCGTTTCTTGATTTTGAGCGATTAAAAGTGTGTTTCAGAGTGATTTGAAATACGACAAAAGTGATTTTAACCATTTCAAATTCACTCTCAAACATGACTAAGAATACTCAGTCAAGAGCAGTCTAGTCAATAAAAGAAGAGCTCTAAGTAAGTAGATTTAACCAGAAAAATGTCAATTGATGGCAACTTTGTTTCTTGAAGGACAATGTAAGAGTTATGTTGCTGAACCAACTTCTTAACCGAATCATCCTTTTTCTAGGAACATAAGTCACTCACGTTCCATGAGAGGAACATCATGAGTCAAACGTGGCCCCCTCCCGAATAGCCAATGTGATTGATTTATCTTAGTGAATCAGGATATTGGCCATGTCTCGAAGGTTTAAGAGGGAGGTGTGTGGTAGGTGAGATGGTGGGTTGGCTATGAGGTCGGGAGGTAGATGGAAGGGTCTCATCAAGGGAAAAAGGACAGGACCCGACCGTATGGGAAGGGCAGAACAAATGGGTTGGTTTATTTGATGGTTGGTGGGGACTAGGGTCCAAATCCGAGGGTAGAGGAGCTTGTTGATGGGTTTCGTTTGGGTTGTCTCCAAAAATTGCCACGTCAAGGGGATTAGGGGCAAAATCATAGATTTTCGTGGCAGGGCTAGACAAATTGGTTTTAGTGTCGTATCCATGAATGGAAGGGTCTGTTTTGAGGATGATGTGAAGTGTGCTGTGCCCACGTGGAGGGTGGTGGTTCCGTTGTCAAATGTAATAGTGAAGAGAGAAGGCTGAGAGAAAGAGGGAGATGGTAATTTTATGGGCCCAGGGATCTGGTCTGGGAAGGAAAACGAGGGAGAGGGGCTGGCTGTATTGAGAAGGGATCTGTGGGATTGGAGATTCCTTCAAAATAGGATCTTCGGTTGAAATCTGTGGAGTTTCCAAAGAAGAGGGATCAGTAGATAAAACAGAATCTACAATTGGTGATTTACTTTTTCCATTAATGTTCGATACCATAACCCAATTAATTTCGGAGAAACTGGCCGCCGATTGGGAGGGCGAATGTCATCGACACGACACTAGAGCTGAACCATAATTGAAGATTCGACAAGGTCTGGTAACTCCACGGTGGCCGGAATAAAACCCAAGGAGTTTTGTTTGTCTTCACTCTCGCCGATAGTAGGGATAAGCCTCGATCGGTTGGATTCGAGACTGCTAAGAAACCTCCAGAACAATCTCCAATGTATCTGAAGAGTTGGTCATTCCATAGAATTGGGAGAAGATTTGTGATGCCAACCCATCCTCCATATGAAGCAATTGAGTCACATAAGAGGATGTAGTGCTAGTTGCCAAAATCTCAAGTCTGAAATCGCCAATGGTAGTCCAAGATTCCTGACAACATAGTTTGGAAGCAAGGTGGGAGTCATAGACGTGGAGCAGAGCTTTGTTGTCGAGGAAAGGATTAATCAAGCAATTGATGGATGGTGGGGACTGAATTGCCAATTGGATTTCCGGCTAGGGATCATGGAGTTGATGACGCTGAACCACAACCATCGCGACCCAGTCTTGATGAGGTAGTGTGACTGTCGATGCCAAGGTGGCGGGTGAAGCAGTGGTAGGTGCTGTCGACTTTGGTTGCAAAACAAGGGAGACTTTCTGTTGGACCACCTCCCTAAAGGTCTGTTGGTTGCGAACAGATGATGGAGGCTCTCTATTGTAGTTGGAGATCAAGGAGAAGGAGAACCAGCCATTTTTATTTTTCTTTGATAGTATAAAAAGCTTCATTCGTCGGCCTGAGTGCTGCAACAGCACAAGAAGCCATATTTTTGTTACTAAGCTTTTCCAACTATAGTGTGTCGTTTCCAATTTCTTGAAAAACTTGAAGTTGCAAGGGCTAGTCCAAAGGAAATTAAAGGAGGAGGTTAGCCATTCAAGAGCAGACCATGAAAGGGAAATGAAGTAAGTTATTGATCGACCTTGTTCTTTGATGTAAGCCCTTGTAACTCAAGATGAAGCATCAGGGAAAATGGTGAAGATTTTCTGGTCGATGAGAATGGAGCGTGGGGGAGGTTGAACGCCGGAGAAAGGATAGGAAAATTAGGGGAGAGAGGTGGGAGCATTTTTTGTGCCTGTTCTTTCTGTCCGTGTATATAATCAAGAAGACTAAAAGGTAAGCATTAAGAAATACAAAATCTAATATAGTTTTAAAATTATATAAAGACATACTTCGACTACTTGATAAAAACTAACTAATGAATTATCGGAGGAACTATTAACTTGAGTTGAAACATTAAAGGACCACTCCGGAAATTATGAAATCGCAGTCAAAGAACCACTAAGAAAACAGCAAAATAGAAGACAAACTTCAAATGCAAGTATTAATGAACTATTGGGGAAACAGGCGAGGAAAGACTGCTTATTTCTTCATTGTCTTTCCTCAGTCATCAAGGAAGCCTTTCTCGATTGTTCTTCAGTTGAGGAATGGCAGTAAGGAGAAATCTTATGCTTCCTTCTTTTACGAGGCTACATGTGTAATCTCAAATGGTAAGTTCTTTTTGTTTAAAAAGAAAGAAATTAATTTGTTTGACTTCCATCCCTGATTCCCTTCCGAACAACAAAATTCCGCGTTTATTGCAATTGCATCTTGTTTACTTTCAATTGCATTTAGGATGTTTACATTGTATTTGTTTGAACCTGCCAGTTTCAACTACACGTGTATGTATGTGTGCTGTTTGTTTTGTTGCATGGCCTGCGTAGTAATTCAGGATATTGAAGCATGCCAACTATTGTTGTTCCACAACCTTTTTTTAGTTGGTCTCCCCTTTTTGAGACATTTCTAGTTGTTTTTCGTTGTATTCTTTCAATTTTTTTTCTAAATGGAAGGATGTTTATTCTTATTCATTAAAAGAATTAGATTAAAATGTCATTTTGATTCTTATAATTTAAAGTTTGTTCAAATTTAGTCCTTGAACTACGAATAAATCTTAAATTTAGTCTCTCAAAATTAATTTTTACTGAAATTGGTTAAATAATAACAATAATTTTCATGCAAGGAAATACAATATGTGATATGTTTTCAAAATTTACAGTAAAAATGTTAATAGATAACAAAGTTTTTTTTAGAAAAATCAATAATAAGTTAGCCATGGACTAACTAAATGTCATACTTATTGAAAATAAAAGGACTAAAATTGGGCAATTGGAAGTAACAAACTTCAAAGTAGAGGACCAAAATAGTATTTTAGAGAAAAAAAAAAGAAAAAAAAAAACTATTAGTTTATGAAATGAAATCACATTGGCGATGGTGGCTAGGTGGTTGTTTTATTCACATCCGTCATGCTCTCGCTTTATTACACTAGAGAGAAAATATAATGATACCTACCTTTTAGTTTTCTGGGAAATTAATAGAACTGAAGTGGATATGTTTGTCTTGAGTTCTAAAGTCTTTTTGGTTTTTTCTGGAAATCAATAGAATATTTATGAATAGGTTTTGCTTTTTTCAGGTGAAAATGGCTGAAGAAACAGTGAAAAGAGTGACAGGAGTGAATCCACTGCTTGTTGCAATGTCTCAGACCCAAATGCCCTTTGTCAATAACCAAATGCCTATGCAATCCAACACTCAATTCTTCCATCAAAATATGTCTGCATTTGCAAACAGCCCCCCACATCACCATAATTTGGAACCTCCTCCCATTCCACATGTTGGAAGATCACAGAATGACGTGGCAACCAAAATATCTGATATGCCCTCTGTGCATATTGATCACGTGCAGAAGCAGGCCATGCATGGCCCTTTGTCAGCTTGGGATGCAGAACCTCCCCATTCAACTCCAAACCACAAGAAGAATTGA

>ClabZIP43

ATGCTGATGGAACCCAATTTCTCGTCGGCGAAGCCACCGCAGCCGGCGGCGATGGACATCGAACAGATGTCGGAGAATCCTCACCGTGGTTCCCACCACCGCCGCTCCCACTCCGACACTTCCTTCCGCTTCCCAAACCTCGACGAACTTCTCTTCTTCGACCCTTCAGAGCTCGACCTCTCGATGCTCTCTTCCCCTTCTTCTCCACCTCCTGGCGGCACTGCCATGGCAGTTGATTCGTCTAATGCTAAGTTTTCTGATGACGCCGTTCGCCCTAAGCCGGAGCCGATTGCCTCTGGACCATTCGGTGGTCACTTGCGGAGCTTGTCCATGGACTCTGATTTCTTCAAGAATCTGGACCTTGGTGGTGATAGTGGGGAGATCGATTCTTTGGGGAAGAAGACTCCGGCGAGTGAACAGCGGCCAGTTCGTCATCGACATAGCCTGTCCATGGATGGTTCTTCGTCGTCGTTTGAGGCCGATTCAACCCTAGTGATTGATGGAGTGAAGAAAGCAATGGACCCGGAGAGACTTGCGGAGCTTGCCCTAATTGACCCGAAAAGAGCTAAAAGGTCAACATCTACTCGTTTTTCTGTCTTTGATTGAATTTGAACTAGAAAATTTCTATGTTAAGTGTAATTTTAGCTGAATTTGAAATTGATGAACTGAATCATGGGTGGGAATGAATTTTGGAATCTAGGATTCTAGCGAACAGACAATCTGCAGCTCGTTCGAAAGAGAGGAAGATACGGTACACGAATGAGCTGGAGAGAAAGGTTCAGACTCTGCAGTCCGAAGCTACCACTCTGTCTGCGCAGGTGACGATTCTACAGGTATGGATTCAATTGCATTCATAGCGAATTGGAAGTTTTGTTAGACAGAAACCTTATGCTGCTTGTGTTCTTCTTAAATCCATTTGGCTTGGCTATGAAATGTCAAGAAAATAGATCAGTATGGCTCCTCGAGTTTACTTGATTTCTTGCAGAAAAAATTCAGAGGGCTGTATTTCCCTCTGATGCATGCTATGTCTGTTCGAAAGTTGAAGAAAGGGATTGATAAAATGCAATAAAGTTGAGATATCTTCTTGAATTCCTGGGTGAACTCAAGTTGGATCATGTTAAATCATCACTGAACTGAAAGCTTAAGTTGATAATAGTGGCATTTTAGTCTTTCATATTTATTCCTACCACTCACCTCACTCGTAGGTTTGGAAAATTAGATTGGTTTCTTAGGATATTATGTTATGGTAGAAGGAGAAGCTGATTTAAATCTGCAACTTATGAAGGAAGCTTCTTGTACTTGAATGCTTGACTTGCAAGGATTTGAACTCATTACTCCACATTTTGAATTTGAATTTTTTTCCAATATAAAATTTAAAGTAAAGTAGATAGGATTATGTATATTGAATCTTTTATACATTTTCTTAACACTTCCCCCTCATAGATGATAATTTGATTCACAAAGCCCGGCCCCTAATATTATTAATGTCAATTGAGGAGAAAACAATGTTGATAGGATTAGAACACAAGACTTCTTGCTCTCATATGATAAAAATCCACATAAAGCTCACAGAAACAGGAAGAAGAAATTATGGTTTCTTTAATTAACTATTCAATCCTGATACTAGTGCTGTCAATGGCATCCATCATGTGGATGTCGTCTTGTTACGGCTTCGTGCTTCATTTTTAAAACATCAAATCATGCTATCTTCTTTTCTTAGTATCTACGTTTGCCTAATATGCTTTGGTGACTTGGGTTTACCTTATGTGCTATATTGTTATCCACAAAGAAACCTTCAGGTGGTCTATTATTACCTGACCTGTATAGTTTCCCTTTGCTGTTCAATTTGTCTCACGCCCATTTGTATACAAGTTTAAAAATCAGTGGCTTTACTATAAACTCCAGTTATTCAATCTTTGCAACCTCCTGCCTTCCCGTCTTTAATAGATTCCATCACTTGATCCGACTCAATGTTTTCCTTCGACTGTGATTTATTGACCATTTAACACTAAATGTCTTTGACTCTCAATTATATAAGAGGTTTAATGTTGAAAATTTGTTTTCATTTTTTTTTTTTTTTTTTTTTTTTTTTTTTTTTTTTTTTTTTTTTTTTTTTTTTTTTGGTAATCCACATTGGATTTAGAAGCTTTTTCTTTCCCTTGGTAAAATTTCATCTATCAATAAAATTCTTGTTTCTTCTAACAAAGCTCTCATGTGATAAATTGCATGTAATCCAGAGAGATACAAGTGGATTAACTGTGGAAAATAAGGAACTCAAACTTCGGTTACAGGCTATGGAGCAACAAGCACAACTTCGAGATGGTATGTATGTTTCAACCCTTTTTATCCAAGATTTAAAATGTCGATATCAACAGAAAGGTCCTAATTTTATGAAAATTTCAATGGAAATATCGATAAAATGTCGATTTCAATGGATATTTCTGGCAAAGTTATAAAAACAAAAAATTTAAAAATATAAATTTAAATAACATTTTATGATATTTAAACAAGTTAACATGTCTATTATTTATACATTTACATTAGTGAAATTTTGTTGATTATTTTTTATGTTTTGTGGATTTTTCTACGATATAATGGAAATTTCGATTGACCCCTCATGTCGATATCTAATCTATAGAAACATAGAAATATTGATAGAAATATTGACATGTCGACGGAAATTATATACTATATTTTTATCTCTCACCTTTCACTTTTATAAAAGCACTTCTAACATGAGTACAAGCCAGCAAAAGAAAGAAGGAAAATATTTATGTTTTTAGAGTTTACCCTTCGGCTTTTCCTCCTCTTCTGGAATGTGTTGTTCCTTGCATTTTTCTTTGTTTTCTCTTTTCTCTCAGTTAGAGGGTAGGTTCATTTCACATACACAAGGATACACAAGGATTCACATAATTCTTTTTTGTGCTCCACAGCTCTGAGTGAAGCTTTGAAGGAAGAAGTTCAACGGCTTAGAATAGCTGCAGGCCAGGTTGCATCGATCAATGGAAATCTTTTCAACCGACCTCCTCAATATCCTTCATCCCGGCCACCTGTACACCATTTTAGTAGCTCCCATGCCCAGCAGGGTCAACAGCAGCAGCCACCATCCATGTTGGCTACCAACCAGCAGCAGCAATCAGATCCCAAATGGACAAACTCGTCTCAGCTTCTCAGTCGAAGTCCGGATGGCCAAGCAAAGCCTTAG

>ClabZIP44

ATGGCAATGATTTCATCCGCTTTTTCTCATACCAAGGTGTACTAGTTTAACTAATGGGTAATTGTAATGTATGATTTTGGTGTTTTCAGTGTGAATGTAAAATAGTTCTCTAGTATAGCCAGGTAAGTTGCATCAAATTGTATGGTTTGATTGAACCAATCTTGACCGATTATCAATTTGAAGGGAAAAAAATATCCTTCTTTCGTAGCGTTTAGTGCTTTTCAGATGAAGAGAGGGATCTATTTATTATTATTATTATTATTATTATTATTTACTTTTCTTCTGGGTGAAATCAGGATGAAAGAGAGCTGAAAAGACAAAGAAGGAAGCAGTCAAACAGAGAATCTGCTCGTAGATCTCGGTTGCGCAAACAGGTAATTAGGTGTTGTTAAGGCTTATAATCAAATTGGACAATCTTAAACTCATGTAGCCATATTTTTTTTGATTAATTGGTTTTTCCTGCATGTTCCATTTTTTTTTTACTTAGGCTGAATGCGATGAGCTGTCTCAGCGTGCGGAAGCTTTGAAGGAAGAAAATGCGAGTCTTAGATCTGAAGTGGATAGGATTAGGAGTGAGTATGAGCAACTTCTTTCAGAAAATGCCTCTCTCAAGGTGATGCCAAAAACCTGA

>ClabZIP45

ATGAGTGGCAGTGAGATGGAGAAACCACCAAAAGACAAGGAGACGAAGACCCCGCCTCCTACTACTACCACACAGGTTTAATTTAGGCCCGATTTGGAGTTGAGTTTTATTTTATTTGATTTGTTTAAGTGAAAAAATATATATAATTTTTACTGTGTAAACTCATTCTTTGTATTTTTCCTTTAACGACCAAATTCTTCCTAAATAAAAATATGGGCCGAGAGAGTAATTATCAAGATCAAATTAGTATTATGTGTTAAATGGAACCATATCTCATTTCTTTGTCTCTCCTGAACTGTTTTATCAAACAACCTAGAATTTCTAGTACAGAGAGAAACAAATACATATGAGCAAACAGTTCTCGGGTGCAGACCTCAGATTAGTATTGTGTGTTCAATGGGACCTGATTCACTTTATAAAACAACTTAAAAATTTTATATCCAATTATCACTGAAAATCCTAGCTTACAGTTCCTTAGTGTTTGTTTCATTTATTTAAAGTTTGTCCTTGCAGGTTCTGTTTGATAATCTGTTGTTGTTTTACAGGAGCAGACTACAACCACAAGCGCTGGCACAGTTAATCCTGATTGGTCGGGTTTTCAGGTTACACTGGGGCAAAAGCTTTTAGTTTAATCTTGTTTGTTGGGACAGCATTGTGGTTAGATCGTTACATATATACACACATATTTATATTATTGGTTTTCAGGCATATTCTCCTATTCCTCCACATGGTTTCTTGGCATCAAGCCCCCAAGCCCACCCATATATGTGGGGAGTTCAGGTACATGTACCACTGGTTTTTTTGGGTGTTTTATGTCTGCCTTTTGAGCCATATGCACATATTCTGATTGGATTGGGAAATCAGTTTTTATAGCCCTCTTCTTCTTCTTCTTCTTCTTCTTCTTCTTATTATTATTATTATTATTATTATTAGTATTTTAATTAAGGCACTATTAATGTACTTGTGTATCCTAATGTTAAGCCTGTGTCTCTTGAATGAACTTTTCTGGTAACTTTGTGCTATTGCCACATGTGACATGTCTATGCTCAATCATGAGGCTTTTTTGTCATGTGTTCATGGCTAGCATTACTGAGTTCTAATGAATTTTTAATTTTAACAAAATGGTTGCAATGGATGTCTTGATCAGTCAATGATTATAGAGAATTCAATATCCTTCATCAGTAAATTGGCTCTAGCTTCTTTATTTTCCTTTTCCTTCTTTATTTTGTTACCTTCGTAACTTTTCTTAGGATAATTTGCTTAAGTTTTTGTCTTATGTTCCATTTGGTACTATGTCAGGTGTTTACTTCTGTGGTTTAGAAATTAGAAAGCGCCTTCTTTACTTTCTAAACTTTAGTTCACCGCATTGGAATTGGCTGATTTATGGCTTCCAGAAATCTTCTCTTTCCCATTTTTCTTTCCTTTTAGGGAAGGACTGCTTTGTGTATTTATCTAAACGTGGGTTTTTATTTACAGCATATTATGCCACCTTATGGCACGCCTCCACATCCATATGTTGCAATGTATCCACCTGGTGGCATATATGCTCACCCTTCCATTCCCCCGGTATTTCTGAGCATTATGGTTTTATATTTTTTTATTATTATGTTTAAAACTTCTATAATATGCATGTCTGACGGATTTGCTTTATACTGTATGCTATATTCTAATGTGTATAATAGGGATCATATCCTTTTAGCCCCTTTACAATGCCTTCTCCGAATGGAATCACCGAGGCCTCTGTGAGTCTCCTTCTTATTTTATGATAAGGTATGATTGTCTTTAAGCGGATAAAAATGACATGTGAAAACATATATAGGGTAATACAGCAGGAAGTTTGGAGGGAGACGTTAAACCACCCGAAGTGAAGGAAAAGCTGCCAATTAAAAGATCAAAAGGAAGTTTGGGCAGTTTGAACATGATTACCGGGAAGAACAATGAGCTGGGTAAAGCATCTGGAACATCTGCCAATGGAGCATACTCTAAGAGGTTGTTTAACTTTTCTAGAAATTGCCCATGTAGAATAATAATTGGTGTATGCTTTATTTTTTTTTATTAGGAAATGATAGGGATTTTATATAACAAACAAATCAGAACAATGGCCATGTGGTTGCAGGGACAAGTCATCCTCTTCCACAAGGGAGGGGTCAAACTGAAGGTCTTCAATCTAGGTTGATAACAAAAGAAGAGTACTTACGAAAGGACTTATGTAGGATTCTTGAAATCTTCTAGGTGAAGTTAGTCTATGCAGTTGCATCTTTATAAGAAAGAAAAGGAGCAAGATGTTCGCAGGACTATACCTGCAAGGGGGGTGGGAGTGAGAGTAATTGGAAAAAGAAAGCTTAATGCTATGGAAGCATTGAAGTGAACTATTTCTTTGATTATCAGCAACTGGAAAGCGCTTGTCAAGTAGTTTCCTCTATCCAACCCGTAGGTTGCTCCTTTTGCGCTTTTGGTGCTATAAACTTCTTATGATTTCCTTAAAAATCTATTTCTTATGATTTCCCTATGATGGGGTTGAGGAATGGTAAAATTCTCTCATATCTTTGAGGTGAAATTTTTTTTCTTCAAATGTTGGAAATAATTCTAATATAACCAAGTTAATATTTAATGAATAGATTCCTCTTATTTGTCGGATCACGACTTCAATGTTGGAGGAATGAGAGATAAAATTAGACATTGTAGAAAAAGAAAAAAAGAAAAAAAAAATCTATTTCTTTGAAGTTTCATGCCTTTAAAATTATAGAAAATGGCATGACCATTCGAGCCTTTTTTTTTGTGTTTTCTTTTATGGGGGTTGAGGGCTCTGCAGTTGAATAAAACATTTATCATAGTAGTGAGGACGGACTAACATGGGAAATTTTAGATTTTCCATGCAATGAACAAGACCTATATAGATGGTTCGTAAAAAACATTCACTTTAGAATTTTAGTCCTATAAAATTTATTGATGGATCCATTGGAACCTTGGGTGATGTGAATCTGACCTGGTTTTTGTAGTGCTGAGAGTGCCAGCGAAGGTACAAGTGAAGGAAGCGATGCCAATTCTCAAAATGTAAGTAGTGGCCATTGCTTGTCTATTGACATTTTCCTATAGATTCTGTTTCTGTGGGTGAGTCAAACTAATGTTGCATGCCATAAGCTTATCGGGTTCTTTATACATTCTCTTTGTCAGAAGAAATGGACAATAAGTAGTGGGACAGAAAAAATTGACAATTAGGTGGGGGGCCCCCCCCCCCCCCCCCCCCCTCCCTTTCCAAAAAAGAAATGTAATTTCTCTTTCTTACCTCATTGGACGACCTTACTTGAACAGGATCTGTTCTATAGGTTGTACAACTGTTTTTCTTACCTCATTGGTTCTCAGTCTAGTGCTTAAAGTCAAATGATGTTTCCAACTATGAGGAAACCTTGAGAATGAAAGTTGAAGAGTAGTTGAGCGTTGAAATGTTGAACTAGATTGATTGAGGGGAAAAAATGAACTGAGAGAGTTGGAAGTATGTTGATACTGTTTATTTCTTGGATAGTTGTGAGCCAAATTTGTTTGGTAAGGTTTTTTTTCATGTTTTTTTTTTAAGTGCTGTTCCAGTCTTTCTAGATAAAATTGATTTAGCAGTATTTTAATTTTGTAGGAATCACAACCTAAGTTAGGTTCCAGGCCAGACACTTTGGAAGGTTTGGTTCTTTTTTCTTTCGTCTTGACAATATCTCATGCCATGCATGGTAAAAAATACATCTTCCTGTTACAGTTGAAGTATCTCAGAATGGTAACTCAATGCATGGTACTCAAAATGGAGGATCTAATACTCAGGCTATGGCTGCCATCCCATTGGCAACTGCTGGGGCCTCAGGAGTCGTTCCTGGTCCCACGACCAACTTAAATATTGGAATGGACTATTGGGGTGCATCATCCACTATCCCTGCTATCCGTGGAAAGGTTCAATCGACTCCAGTTGCTGGAGGACTAGTTACCGCTGGATCTCGGGATAGCATACAGTCACAGCTTTGGCTGCAGGTAAGCTCTAATATGATTCAAAATCTTGGATTTTGAAGTTGAAAAAAAGAAGTATTATCGAATTTTTATTATCGAATTTTGGATTTAGGATGAGAGAGAGATTAAAAGACAGAGAAGAAAGCAATCAAATAGAGAATCTGCACGACGATCCCGGTTACGCAAGCAGGTATTGAAAAGCTGCTGGAAACTGTTGATCTTGTTGTTAGACCTTGTTTCTCGTGCTTCTCCTATAACCCGCTGGTTCCAAACTCATGTTTTTCAGGCAGAATGTGACGAATTAGCACATCGAGCTGAAGCTTTACAAGAAGAAAATGCCAGTCTTAGGTCTGAAGTGAACCGGATAAGGAGTGACTATGAACAGCTACTTTCAGAGAATGCTTCTCTGAAGGTAATACCACAAATACTGCTTTTCCAATTTCTCAATTTATTCTCCCCTTCAACGATTTGTCTCACCTTGACCATCGTTTCATTATAATTACTTTATGATGTCTACTTTTTTATATATATAATTTACATGGTAGTGTACATGCAAAATGGAGCGGGCTTTTTTTTCTTTTAATAGTCCTTGGCCATCTTTTTGGTAATCAACAAGACGGGGGTTGGATAATTAGACTTATGTTGCAATAATACTGTAATCTATTTCATCATATGTGCAGTTTGAGCAATGAGCGTTGTAAAACATCCTCCCTTTCATTGCAGCAGATATGTGCTGCATAAACTCGCCCCATCTAATAGGCTGGATTTTATTTATATGCAGGAAAGACTCGGGGAAGTTCCGGGAAACGAAGAAGTAAGGGCCGGCAGGAAAGGCCAGCGTTTAAGTAACGAAACCACACAACCTACAGAGTCAGAGGTTGTGCAAGTTGGTAACAAGAATTGA

>ClabZIP46

ATGGAAGAAGTTTGGAAAGACATTAATCTCTCTTCTCTTCACTCTCGTTCTGACCATGACTTCTCCGCCGCAGCCGCTGCTCCCGCCACTCCCATCGGCCTCCACCACCACTCCGCCGCCGCCAACTTCCGTCACATCATCCTCCAAGACTTCCTTTCTTCGTCCACTTCCAAACTTGACTCCTCCTCCGCTGCCACCGCAGCCGCCGTCGCTGCCCCACCACCTACTGTCTTGTCCTTGAATTCCACCCGGGAATTGCATTTCCCGGATAACGCCGTCGCCGCCGCCCACTTCCGCCACCAGGATCCTGCCTCCGTCTCCGCCGCTTTCCACAGCCCCTTTGATCAAGTCCTTGGACCACCGCCCTTTGGAAAGAAAAGGGTCCCTGATTCTGACAGCAATAACTCCGGTGATCGGCGCCAGAAACGGATGATCAAAAATCGCGAATCCGCCGCCCGATCTCGGGCCCGAAAACAGGTCAAATTAAATTCATTAAAAAAACTTATATTAATTTTGAAATAACATTATTTATTATTCTTTTTATTGGGTTCAATCCACAGGCTTACACAAACGAGCTGGAGCTTGAAGTGGCACATTTAATGGAAGAAAATGCAAGACTTAGGAGACAGCAAGAAGAGGTAAATTTATACCATTTTTCTTATTTTTTTCCGAAATTATATAGAATTTTGAATTAATTCCCAAATTTTGATTTTTATATTTGAAATTTAAGTAAATTACCAGACCAACTAGTGATTTTTTAACCATGACATATTTACTCCTTACAAAAAAAAAAAAAAAAAAAACGAGTTGTCTCTCTTTCATGAACTAAGAACACAAGAAATAAGAAACAGAAACGTTAAAAAGAAGATTGCATCTATGCAGCTACGTGCAGCAGCAACAGCGCAAGTCCCGAGAAAGCACAGGCTGCAGCGGACATCGACGGCGCCGTTCTAA

>ClabZIP47

ATGGCTTCTATCCCAAGGCAAACAAGCTCGGGATCTAATGGAGGTTCGCCATCTGCTCTTCCTGATGAGAGGAAGAGGAAGCGGATGCAATCCAATAGGGAATCGGCTCGGAGATCTCGGATGAGAAAGCAGAAGCAATTGGAGGACCTTGCTGGCGAGGTAAGCCGATTACAAATTGCGAATAATCAGCTTGTGCAGAGCATTGGTGCCAAGGAGCAAGCATTTGTACAGGTCGACAACATGAACAACGTTCTCAGGGCTCAAGCTATGGAACTTACTGATCGTCTGCGGTCCTTGAACTCTGTCCTTCATATTGTGGAGGAGGTTAGCGGTCTTGCCATGGATATCCCTGAAATACCCGATCCTCTCTTGAAGCCTTGGGAGCTCTCTCGGCCAGTTCTGCCCGTTGCTGACATGTTCCTGTGTTAG

>ClabZIP48

ATGGGTAGTAGAACAGTGAAGATTGGTGCAGACGATTTAAACAAAACGGTGAACGGGATGCCTAGCTTCGTTTCTTCGATGCCCACAGCCAATAATTCCATGTAACTTTCTTGGCTTGTGTATAGTTTATCTACTTTAATTTGAACTATTGAATAACTATTTTGCGTACGCTGTTTGAACTTAATTTTGCTGTAAACGTACTTGGCTTTTTGATGAGTCTGTCTGTTTTTCTGCTCGTACTTACTTCTTACTTGATAAACTAATCGTGCTTTATTAATTCATTGAGGGGACATAGGACCATAAGACTTTATTAATACCTGCTTTAATTTCAATGGGGCTCTTTGTTTGATTCCATGGGTTGACCATAGCAACGTTGAAGAATTCTATCAGGTCATCATGGGCTACAGAAGACTTGTAGCTTATGCTTTGTTCGATCACTTAAGAGGCTAGGGAAGGGAAACACTAGATTCTTAAGCTCTCATTGCAATGAAAGAACCCAGCTTTTCGTTGTTATATGAAAAGTAAGAAAAAGTTTAAGGATATAACCTCCCTAAGAGGGAGAAATAGAAAACCTCCGGACAGAACCAACCTCCCTTAAGCTCTCAGTCTATGTTTTTTTTTTTTTTTTTTGGCTTCAAATCTCTGTTCTCTAAACATGAACCAATTTAGAGGGAATAAGCTGTCAGGATGATGTAACAGTTCTCCTTGCCACATAACTCCCCCAACTTGGGAAATATAGATGGAATTAATATTGAAACTACAGATGTAAGTTAGAGTAACTTCTTTTATGCATTTGAGTTTAGTGCCAAGGAAGATGTGATTCTTAACATGACTGGCAAACTAAAATGGGATGTATACATTTCTAACGGTAATATTGTTATATGTGAAGATTTCAGAAGTTTTTTTGCAGACAAGCTATCCCTTTGGCTACCTTAGTTGTTTTCTATGTTTTGTTCTTTCTTCCGCAGTTTGCTCCTTTTTGGAGTTCGTATTTTTTGAGCACCAGTCTATTTTCATTTCATTAGTGCAACCTCCTATTGCTGGTTAATAAAAAAGTGTTATATGTGAAATATGAGAGGAATATAAATGCTTCTGGCTGGAGCTTTGTGAACTCTTTTTTCATTGTTGATGCGCTCCAACTCAAATAAGTTCTCTTTTTATTTTGATTTAGTTTGATTACATTACACATTAAAATGTAAATAGCTAAACACATGGTACATGTGTATATGTATGCATGCACATGCTCCTTTTTATACTTGCGATGAGATTTTTTGGGAAACTTTTTATTCACATCACAAAAAATTTTCTCAATTTCAAGGTACATCTGCCTTAAGTTGGTATAGTTATTCTTTGAATATGTGACTTAGAGCATCTTCAACATTTATTATGTCTTAGCATATTCCTTTGTTAAGTTAAATTTTTAGTTGAATTTCAAGTATAACTTAAAGACCGATTTGCTTGGTCAATTCACATTCATGAGTTCAAGAAACATTTGCCCTTCCAGACCCCAGTTATAGATTTGTGGATGCACAATTATGTTTTGATGCATTGCTTAACTTCCACTTTCTTTCTAGGATCTTTTGTTCACCTTTTGCATTATTGTTGGAATTTTCTTTTTCATACTTGATGGTCTTTTTCACTTTGATTTTCAGGGGTGCAGAGGGGAGCTCTATTCCTTCATCTCGGATTTCAGACTTTAGAACACTTGAGCAAACTCTTGGATTTCACATAGAAGATGTAGACCTTACTAGAAGTATGTAACCAAGTTTTATTTTGTTATGGAACTAAGCCTTTTGATTACTTGTCTACTTTCACAGTAGTTTCTGTGATCATCTATTTATCTGATTTCACTCTTGATGTTGCATTATTGAATACACGATCAGTGTATACTGAACTTCTTATGATTTTGACTAAGTTATCTTGATAATTCCTCTGCTATGTTTCTAGCGTGACTAAGAAGTGTTTTAATTAGGTGACCGTTAACAAATATGCCTAAGAACACACGTTCTAATTCTCGTTCCATTACATTCCCAGCTTTTTAGTACTGGCTTTATTTGTTGAACCTTGATCCTGTCATCTCTTTCTATTCCATCTGCAATCTGTCTATTACTTCTCAATTTGACTAGATGTCTAAAGCTTTCTTGTGCAATTATGACTCTCCTACCATGTGATTCTTAAAGTTGCTTAGATTCTTTCCAATTTATAACTAAATAATGTATTTATTTTTGAAAACAAACTAAATGATGTATTTGTAGTATTCATACATTAATAGAGAAGAACAAGAGCTACATTTTCTCTTGGTTCATAAAATCTAAATCTCTGAAAGTCTGACTGCTCTTCTCTTCTATATGCTTATTCTCTTCCTGTCATTCTTCTTCTTCTAATATCTTTTCTTACCAAATATACTTGCTCTAGATCCTTTGTATAATCAGATAAAGTCCAGTAGTTCGACTCTGAACAACATCCAATTTGGTTCTTTAATTAAGGTACTGTTTTACCTGTTCTCATTTTTTTATTAATAGAAAGGTTTATTTGGTTTTGCTTTGCACTTGTATTTGATGGACATTAACATCCCCTGACATAACTATAATGAATTCAGCCACTTGCATCCACTGATGTAAATCTGCCAACTGCTGTCATGGGGTCTCGAACACTGCCATTACAGAAGGAGTCAAATCCAAATTTGGTTTCTACTTCTGGTGGTCCTCGTGAAAATTGGGGAGAGTCTAATATGGCAGATACCAGCACAAGAACTGATACCTCCACAGATGACACGGATGACAAAAGTCAAAGGGTAAAGTCTCTTGGCCTTGAGTATGTTATCAAATTAGTTGTACACGATTCATTTGGGAGATATTGGTTTATTTTGTTTAATAATCAATGTATAGAAGTTTCCTGATATCAATATTATAATTTAAAGTTTAAGTAATTTACGGCACAATTGAATAGAGCAGCTAGACGACAGTACATATATAACATTGTCATCTTGTCCAACGGAATTCTTCACATAGTTTATGATTGAAAACAAAGTTTCTAGACTCATGCCTGCCTCTTTCATTTAATTATGTGAATGCCATCAAACTAGAAAGCCGGTAGCGGCAATTAGTTACTAAAATATATTCGATAATTTTCTGGTTGAATGTTTTCATTATCCCAAATAATAGTGTCTATCTTTCTGTTTAGCATTCTTGGGTTGTAAGCTCATGTTCTTTTATCTTCTGCAGCTTGACAAGGATCAAGGAAACTCACTTGCCGCATATGATTCTAGCAACAAATCAAAAGAAAAAACAGCAGATCAGAAGGTTAGATTTTAAAATAAAATAATTTTACATTGTTAGCTGAACATGTTGATGGACAATTAGCAGCAGCTTGTTCAAGCAACTAATTTGTTGTTTGGCATGCTTTCCAGACCTTACGAAGGCTTGCTCAAAATCGCGAAGCTGCTAGAAAAAGCCGTTTAAGGAAAAAAGTAAGCTTCCATTATCATTGCTCTCTATCTCTTATTCCACTACATATCTAAAATTGTAAACATCCTAGTATTGAACAAGCTGTACCTTGTCTGGAGAATTTGGCTTCAATGATGGTTGCAGCTAGTCCTGCTTTATAACTGATATTGACTTGGTTAGAAATGTTCAGTGAATTTGATCTTATTACACCTTGAATTTCCATAATTATTTCATTGTCACTTTCCGTCCGTAGGCATATGTGCAGCAGCTAGAAAGTAGCAGACTGAAGCTTACTCAACTTGAGCAAGAACTCCAGCGGGCCCGCCAGCAGGTTTGGTTTTCTGAATTTTTGTGCTTGACCATCATAATGGCACATTACCTTTCAATTTACCAGGAAAGTGTTAAGATGAACAATCACTTATATCTTCTATCACAATTTGTGCAGGGTATATTTATTTCAAATTCTGGAGACCAAGCTCATTCAATGAGTGGGAATGGTATTGAAGCTTTTTTCTTTACATGTTATGAGATACCTTCCCCACTAAGCTATTGTGTGCCTTTGTTCACTGTTTACAATTTTTATTGACGATGGCAACATCATGTACAGAAAAGGAAAAAAACCTAAAACTGGTTTCATGCATGTTTTATCTAATTTCCTGTCACATCTTTTCTTTTGGTTTGTAGGGGCCCTAGCATTCGATGTAGAATATTCACGTTGGTTAGAGGAGCATAACAGGCTGATGAATGAGCTTAGAGCTGCAGTAAATTCACATGCTGGTGACACTGAACTTCGTACTATTGTAGATAATGTGACTACACAGTTCGATGATATTTTCAGGTTGAAAGGCATTGCAGCCAAAGCTGATGTGTTCCACATCTTGTCAGGAATGTGGAAGACACCAGCAGAGCGGTGTTTCCTGTGGATTGGTGGCTTCCGTTCATCTGAGATCCTAAAGGTAAGAAACTCTATATATTTGCATTTGAAAATAGAGGAGAAAATGTTTGTAGATTGGTGCATGTACGTCTAAGATTATAAGAACAAAAAGTTAAATGGTTCCCAACTCTGTATTCAAATAAAGTTTGTGACCTCTAATGTGTAAAGTCTAGTTAGTTAAAAATGTGATAGCTATGGGATACACGTAGGTCTTCTGCCCCCTTCACTTTCGCTGCCTTTACTTTTTGGGTATTGACTCATGCCTCCAACTGAAAATTTACATAGAACAGTATGTTGACATGTACAATTGTTCTTAAGTAGTGAGCACTGAGCATTAACAAATCGCAAGAGAAGTTGTAGTGTGTTAGAAGCCTGTGCAGAAGATTTAGGGTGTGGTTTATAACCCTAAGTGTAAGGTAAAATGCATGCTCCGGTTGATCGGTAGAGCCTTTTAAGATCCATAAGTCCAAAACTAATTCAGCATGTCCTTCCGGCCACCAAGTTTAATGGTCCTTTGCAATTTACTCGATTCATCTTGATGATATTTGATGATTCTAGTCAAATAAATACTCTTATCCTTGCTTGACTTTAATCATTAATTTTTAATTTGTGTAGGCTTTAGATGTAATAAGCCTTGGTAAAAAATCAAGCTCTCTCTACCGACCTGTTGTTTTATTACTTGTCTATTTAATTATAGACCTTTCAATGTCATTTTCAACCATTATGGGTTACGAAGTGGTCGTCTATTCAGATGCTTTTAAGGTGATAGGTTAAACTTATTTTGGCCAATTGCCACTTTTCTAGAGCATTGAATATCCCAAGCATTTTCCAATGCCAATTATTAGAGGATTAGGCAGTTTTCCCCTAGATTGTTCTTGGGTTGATGGTACACGTAAGTTGACATGGACACCTAAGGGTATCAAGGAAGAAATTAAATAGAAGTGGTCAGTAAGGCTACTGACTTCTTCAATAAAGAGTTTGCAGTTAGAGTTACCTTCTCGTAATGCCCGCCTACTTTTGCCACCTTTGAGCTCCACTCAGTCCTCCTCAGATGGTGACACCTCTGACTGTAGGCCCATTCCATATTTTTGTCTTTCCAGATTTGGTTGGCGATGTTCGTCTTCAAAGCTATGAAGTTTAGAATAAAGGGTAAGTGTTTTTCTATTGACTTTGATTCTTGTTGAAAAGGTAGGTTCTTTCAAATTTTGGAAAGGCAGCGGGAAAGATTCTGAGTTTTCCATGGAACAGGTGATCAATCCGGATTATTGATGCATTTGAGGCCCTATTATTAACCTCACGAAACCAAAATTTCTTTCAGAAAACCTACTGGCATCAATTGATTCATTTGGGTAACAACAAAAGACTTCGACGTAAAGGGGTAGTTATCTTGAAATCACAAAAGTTCTAAGCCACACAAGACTTCGAATCCTTCATTCGGCAAAGAAGATCCTTTATCAATTTCTCAAGGGAGATTTAAGTGAGTGGTGGTGACTAGATATGGCTTTTTCAATGAATGGAGTGCGATCCTTCACTCTCCAAAACCAGCTTTTCGAGTCTCATTATCATTCCCTCCCTTCTGGAAAAGACCCTTTTAGATGTCCAAATGAGGACTTTGGGGGGGGGGGGGGGGTCAAATTTTGGCAACTTCACATTAAAGCTAGAAAAATTGGAGCACGCAGTAGGTAGTCAGTTTATTGTAGATCCTTCTTGCATGGGTTAGGTTAGATTTTGTGTGAGCAGCACGTAATTGATCAAATAACATTCCTACTATTGAAGGTGAATAATTATCAAAATTTGTTCTTTATACAATCCTCCTATTAAGTAATTCATCGATTAAACATTCCTACCTAATGAGATTTTCAACCTCCTCATATACACCCTCACAGGCCGCCCCTTCATTGATGGCAAGAGAATCATGTGGATGTACCTTATTAGAGCATTTTTTGTGGATCATTTGGATGGAACGGAACAACCGTCTCTTCAACGGGAAAGGAAGCACTTTTGATAGATTTTTTGAACGTCTTATCTTTCTTGCTAAGACTTAGTGCAAATGTACATCCTTTTTATAGCGCATATAATCTATCCTCCCTTTTGAACAATTGGAGAAGTCTCTTGTGTCTCCTTTGGTTTAGGCTAATTGCCCTTCTTTTATAAATTTCATATATCAATGAAATATTTTTCTTATTAAAAAAAGAAAAGAAAAGAGGGGTAAAAAAAACAATCCTCACATTTAAACAAAATCTTCAACTACCATCCTCACTGATGATAATAACAAGACGAGACTTGAAACTACCTACTGCTTGGTCCCTTTATGCAATGTGTTGTCAACACTCACCATCAATCTTTCTAAAACTCATTGGTTTCAGTATTCTGTTTAATCTCAAGCTTCAAACTGTTGATGAAATTTTCTTTCAGTTGTGATGAATCTTTGAAAGGTGCAGTCGATATCTTAGATTGCTGTCAATAGTTTCTCGTTTCTCCATGGAATCACTTCTCCCATTATACTTCTAACTGTCCTGATTTTTCCGATTTCTTTTATAGCAATCTCACTCAAAACTGCAACTATGTTAATGCAGCTTCTTGTAAACCAATTAGAGCCTTTGGCTGAGCAGCAGCTAATGGGCATTTGCAACCTGCAACAATTATCACAACAGGCAGAGGATGCTCTCTCACAAGGAATGGATGCATTGCAGCAATCCTTGGCTGAAACATTGGCCAGTGCCACACCTGCAACGTCGGGATCGTCTGGGAATGTGGCAAATTATATGGGTCAAATGGCCATGGCAATGGGAAAGCTTGGAACTCTTGAAGGCTTCCTACGACAGGTAACCGGTTTTAGATGGAATTTGACAAGCGGAAGAAATTATTTCAAAAAAATATATATATTTGTCATACATAACATTTTCAATATTTATATTTACATTGCTTATATCTTTCATCAACGCTTTTTCTCATAATCTGCAACACACAAGTAAAGTTGAAATATTTATCCATGCCTCACTTTCCCATTTTCCTCCTCTCGTTGGAATGCACAGGCTGATAATCTTCGTCAACAAACCCTTCAACAAATGCATCGCATATTGACAACCCGTCAATCAGCCCGTGCACTTCTTGCAATAAACGACTACTTCTCCCGTCTTAGAGCCCTCAGTTCTCTGTGGCTTGCACGGCCACGAGATTGA

>ClabZIP49

ATGGCTGACCCAATTGAGCTCGTTTCCCCCTCCGATCAAAACCCTAATTCCACTACTTACGCTTCCGAATTTGATTCCCTCCCAATTCCGCCTCTCGACTCTCTGTTTTTCTCCGATCCTAATCACGACGCCCCCGGTGACCCTTTTCTTTATTCCACCGCCTTGGATTTGGGATTCGAAGAAAATGATGATTTCGAGCTCACCTTTGATGACCTTGACGACCTTTACCTCCCCTCTGAGGCTGACGATTTCCTCATCTCCGATAATTTGGATCAGACTACCAATTCGTCGAACTCCCCTCCTGATGTTCCTCTCCTGTCTGATGCTGGTACTCGTCTTTCCAGCCCTGCAGGCTCTCCAGGATCGAGGAGCTCTTCGGTTTCTTGTGAGCAATCTCCCGGTGATTGTAAGTCTCTAAACTATCAATCTTCGAAATTAAGAACAGCGGATAGCGAGTGTTTTTCGACTGGTTCTGGTGGATGGGACTCTAAGCGTTCGAGGATGGTGAATTGTCCTTCCCCGGAGGATGGTGCCGGTGGTAGTGATCATGAATTCTCAGGCGGCCCAGCATCGTCGCAGGGCTCGGGTTCGGGCGTATCTGAAGGAACGAATTACCCATCTTCTAATGCGGAATGTTATGATGTTATTTCCGACCAGAAGATTAAATCAGAGGAAGTGGGGAAAAATTGCATCACGAAGAGGAAGAAAGAACAGGATGAAGGGAATGCGGATTCAAGATCTGCCAAGTATCGAAGGTCATCTGTACCTGCAGAAACTACCAATCCCCAATTGGGTTCCTGTTCTGTAAATGAAGATGATGAGAAGAGGAAAGCGAGGTTGATGAGGAATCGAGAGAGTGCGCAGCTTTCCAGGCAGAGGAAAAAGCATTATGTGGAGGAGTTGGAGGATAAAGTTAGAAACATGCATGCAACCATTGCTGAATTGAATAGTAAGGTATCTTATATGATGGCTGAGAATGCAGGTCTAAGACAGCAGCTGAGTGGTAGTGGTATGTGTCAGCCTCCTCCTCCTCCTCCTGGTATGTATCCTCATCCTTCAATGCCTCCAATGCCTTATACATGGTTGCCATGCGCTCCTTATGTTGTAAAGCCACAAGGGTCTCAAGTCCCTCTGGTTCCAATTCCTAGATTAAAGCCCCAACAACCTGCTCCTGTAGCAAAGGGGAAAAAGAATGAGAGTAAGAAGGCTGAGGGAACAACTAAGAAGGTTGCCAGTGTTAGTTTACTGGGTCTAATGCTCTCTTTTATGCTTTTTGGTGTTCTAGTTCCTTTAGCGAATGTCAGATTTGAAAATGTTGGAGGAGGTCCTGGTAAGTTGTCATTTGTTGGTGATAGGGTATACAATAGTAATCGGAGGAGAGTTTTGAGTGTTGATGGATATTCCAATTTATCAGATGGTGAGAATGTAGGAACTCCTTGTGGGAAATCTGGTACTTCGAACCGCTTACAATGTGAAAGAAATTATAGGAAAGGGCGAGACTTGAAATACAATCAACGAGGAAAGGGATCGCAACGTTTAAATGATTCAGATGAGTCTATTAAGCTTGGAAATGCTAGCGAAGCTCTCGTTGCTTCTTTATACGTTCCAAGGAATGACAAATTAGTGAAGATCGATGGAAACTTGATAATTCATTCTTTCCTAGCTAGTGAGAAAGCTATGGCCTCTCGCAGGGCTTCTGATACCAACAAGGCTAGGGAGACTGGTCTTGCAATTCCTAGAGATCTTAGTCCAGCCCTCACCGTCCGAAACATCAGGGCACTTACTTCTGGTTCAGAGGACCATATCAAGGCAACTGCGGCTGATGGTAAACTCCAACAGTGGTTCCGTGAAGGTCTTGCAGGTAATGCCCAGCACACTAACTTTTTTTAATTATGATTATTATTATTTATAGAATTTTTATACATGGATGATGATATTTGAAAGTTTTATTTGTTGCACGACCTACTCGAATTATTACCTCTTCTATTGGGATTTAGAATTCTAATGGTATCAACTATCAAATGGCTGAAACAAGACATATTGACACTTGGAATATATGCGAGCACTGTAACCTTTCTTTGGTTGACTCATTGACTTGTTGTTTCCCTTGTTAGCCTTAAAATTTGTTATAAAAATCTTGGACTTGAATATTCAAGGGCTTCTTTAATTAATCTGATTGTGACATAATATCCTTCAAAAGGTGTCTTGACCATTTAAACCATATAACATAGTTTCACAGGTGGACTAGGTTTTTAATAAGATACTGTTATATGCATAAGAATAGCTTTTGCACAGAAGTGTGTGATAGGCTGTTTGCACGAATATAATACTTTTTGCCCTTGAAATAGCAACATCTACCTGTTACTTGTTGGAAAACTTGCTTTCCATGTCTACGAAAAAAGGGGTGGAGACATAAATTGAAACTAATGAACTTTTGGGATTCCATGTAGTCGTGATTGTCTTGCTTATATCTGATTGTGTAGGTTATAGATTGATATGATGTATGTCCATTGGGGTCGGGAGGCTGTTGTCATGACTTTCACACCTTATTTATTTTCTTAAAAAGATTTCTGACCCATCACCCAGTTTCTTTCTTTACAGAATCTTGTTGAAACTAGACCTTATCTTTTCCTGATTTATGACCACAGTAGTAGGTGGTAAATTGAATTTGGTGGACTTGTTTACTTAGATGAGGATTTGATCCAATGATTATGAGACGTATATTAGATGAGTGTCAAACTTCAATATCGCTCGAGTCTTCCTTGATTAATGGTCTTGTCAGGTGCGCGTTCGACATTGTCTTGTTGATACTTATGGTTTTCCTTCTCCACCTGTAGGACCGATGTTGAGTTCTGGTTTGTGCACTGAAGTGTTCCAGTTCGATGTTTCGTCAACAGCTCCAGGAGCGATAATTCCAGCATCTTCCATTGCCAATACTTCTAGAGCACATCGTAAGAATGGTACTCATCTTAACAAGGCAAAGAACAGGAGAATCCTGGGTGGTCTTCCAGTTCCCTTAAGTGGATCAAACTTCAACATCACAGAAGAACCTGTTAGAACCCCACACAAAGACAACTTTCCAGGTAACAATAAAACAGCTTCGTCGATGGTAGTTTCTGTGCTCATTGATCCAAGAGAAGCTGGTGACAGTGAAGTTGACGGCGTGATTACGCAAAAGTCTATTTCAAGAATTTTTGTGGTCGTGTTGCTGGACAGTGTCAAGTATGTCACATATTCATGTGTTCTCCCCCGCTCGGGCCCTCATCTTGTGTCTACTTAA

>ClabZIP50

ATGGCTTCTTCCAATATTAAAAGCACCTCCATCATCCAACAAGCAAACCAGCAAGAAGAAGAAAAACAACAACAACAACAACTTCTTCTTCATCATCAACAACAACAACCTCAACTTTTACAAACTCCAAGCTTTGTCATCTCATCAAACTCAAAAAATTCCTCATTATTCAACTTCCAAAACCCCAATTTTCCTGATCATCATCATCATCATCATCAATTACCCTTCTCCATGAATCTCCCTTCCATCCCTTCAAATTTCTTGTAATTTTTTTTATTACTCTTTTTTTATTATTATTAATTAATTTATTTTTATTGGTTATTATTTTGAATTATTTTGTTAAATTTTTAGAAGCAAAGATGGAGGAGGAGATGATTTGGGGGAGTTGGATCAAGCTCTTTTTCTCTATCTTGATGGACAAGAACCCTCTACAACCACTACTCAAGACCAAAGACGTATATATATATATATATTTTTTTTTTTTGTTGGAAATTTTGAATTATTACTCTCAAAATTCCATTTTGGGGTTCTGAGTGAAGATTCTTTCACACTAACAACATGGGATTTTACTGATTTTTTGATTTTTTGAATTGATCTTCTCTGTGGGTTTTATTTGGATTGTACTTCTTTGACCACTTTTTTTTTTTTTTTTTTTTCTATCTCTCTCTCTCTTTATTTTTCTCTTTGTGGGTTTTGAGTGATTCCCTTTTCCTTTCATGCATACACAACAATCTGATCATCACATGTCATAAAAATTCATCATGTACTAAGGGTATGGAGAATATATTTTATTCCTTTTTTCCCCCTTCCATTCTCTCCTCTCTTGGGGTATGAACCAAATATTCATCGCATGCATTCAAATCTCATCTAAAAAAAAATCCTTCTTTTTCTTTTTTCATTTTCAGTTAAATTTATCGGAATCCATCCGCTTAATTTGATAATCACTTGATAGATTTTATGTGCGTTTTTCTTTTGTAGAGAGTTTGGGCATGAGACCTCCGACTTTGAACATCTTCCCGTCGCAGCCGATGCATGTCGACCCGTTACCAATAAAAGTAAATTAAATAATTAGAAAAAAAAAGAAAAAAAAAAAAAAAACTCTATCTTCTCCAACTTGACGATTAGTTATATTATTAAATGGAATAAAAAAAAAGAAGAAGAAGAAGACCATTTTTTTTTTTTTTTTAAATTTTGGGAAAATGAGAAGAATGATGAATTTGGAAGGGAAATACAGCATTGATAAACCATAGTGATTCAAAGATCAAAGCATCTGAGCCGTCCAGAGAGTTGGCCAACCAACGCAGCAGCAATGGAGCCACTGCATCTGGACCACCCCCAACCCAAACTACAACAACCGAACCTCATCCCAAACCTCCCAAAGTAATTTAACCCTCTTTTCTATTTTGCTTTTATAAAATTATTTCTTTCCCATTATTAAATTAAACTCTTTTGTTTTCCATCTTTTATTTTATTTTTATTTTTGTCACAGCGTGAGCCCAACAAAAAAGGACTAACCTCAAGCTCAGAACAAGAAGGACCCAAAACCCCAGACCCCAAGGTCTCAAAATCACTCAATTATATAGTATAGTAAGTTTAATATTATATCAAATACTCTAACCATTTTAGCCGTGTTTCAGACCTTAAGAAGATTGGCTCAAAATCGAGAGGCCGCTAGAAAGAGCAGACTTAGAAAAAAGGTTTCTTTTTTTCTTTTTCTTTTTATATATTATTTATATACATATATTTATAATTATAGGTAATTAATTTTTGGTGTGTTTTAAATGAATTTGTAGGCGTATGTTCAGCAGTTAGAGATGAGTAGGATTAAGCTCACCCAGCTGGAGCAAGAACTACAAAGAGCAAGAACTCAGGTAATACAATATTAATTAAGAATCTAAATTAATTATTTTTCTTCTCTAATTGTGTTTAGTTTTTAATGTACTGATCATGTTATTTCTTTTTAATGTTCATTAATTAGGGCATGTTTCTGGGTGGAGGAGCCGCCATTTTAGGACCTGACCAAGGACTTCCCTCTGGCTTCTATAACCTCAGCTCCGGTGATAATCCACAACTCTCATCATTTGGCCGTTGAGTGAAAGATTCAATTTATCGTAATCTATCAACTTAAACTTTTGTCTAACGATGATTTAACATAACATTAAGCCTACGAGTGAGTCACAATATTAAAAGTATGAATAGTAGAATTATTTACACTAACTTATTATGGTTTATTTTTGTTTAACAGTCCATTTGAATATAACTCGGTTATAATTATAGTGTTGGATCTAATAATTATTTAAGATTTGGTGTTGAAATGTTGTGCTCAAACCCTTATATAACAATATTTTTCCATTTGCAATATTTTAGCGTGTGGAATATAATAATAAATTGTCTTTTCCAAATTTTTAACAAATATTTAATTGTCTCCTGAAAAGAAAAAAAAAAAAAGAAAAAACATCTCCCCTCTCTCTTTGTTGTCTATCTTAACCTATCAACTTGAGGATTTTGAATATATTAGCTAAGTAGTGTAACATTGTAGATGCTGCGGTGTTCGACATTGAGTATGGACGGTGGCAAGAGGAGCACCACCGGCTAATGTGCGAGCTCCGAGCAGCAGTGCAAGAGCAGCTACCGGAGAACGAGTTGCGGTTGTTCGTGGACAGCTGCCTGGCCCATTACGACGAGGTTCTGAACTTGAAGATGATGGTGGCTAAGTCGGACGTGTTCCACCTTGTCTCCGGCATGTGGAAAACGCCGGCGGAGAGATGCTTCATGTGGATGGGAGATTTTAGGCCCTCCGAACTCATTAAGGTACCCTTCACTATTATCATCTGA

>ClabZIP51

ATGGTGGTAACAGAATCAGAAATGATTTCACATGATGAAGTTGAATCGCCATTGCAGTCTGAGCAGCAACTCAAGCACCATGGATTCTCTTCCCTTGGAAGGCAATCCTCCATTTACTCGCTTACACTGGATGAGTTCCAGCATACTCTTTGTGAGAGTGGCAAAAATTTTGGCTCCATGAACATGGATGAGTTCCTCACCAGCATTTGGACGGCGGAGGAAAACCAAGCAATTAACGCCAGCCAATCGGGCACGTCTACCGTTGCTGCCATGGCAGCATTAAGCAATGCTCAGGCTCACTTGCCGGTGAGTGGAGCGTCCATGGAGAAACGCAACATAGAGAAGCAAGCAAGCTTACCCCGCCAAGGTTCGCTTACACTTCCTGCACCCTTGTGTAGGAAAACTGTGGATGAGGTTTGGTCTGAGATACACAAGGGCCAACAGGGGCGGAATCAAAATAGCAACAGTGGTAATGCTAACTCTCAGAATCCGGAATCTGCCACACGCCAACCGACGTTTGGAGAGATGACATTGGAGGATTTTCTGATCAAAGCTGGGGTCGTTCGAGAACCTTGCGCCGGGGCAGGAGTGTCGCAACCGTTGCCACCGCCTCAGCAATATGGTATGTACCAGAATAGCAACCACACCATTGGTGCTGGTTATGTTTCCAGGCCTATTATGGGACTTAATACATCTGCAGCTGGTGGTGGTGCTAGCAATAATCCCAGTGCCGGTGGCATTACGACATACCAACCAGTCCCCCAAGGTGGTTCTACTATTGGAGATACATCAGGATATGTTGGCAATGGTAAAAGAAACAGTGTGTTCTCATCACAGCCTCCACCTGCAGTTTGTTATGGTGGAAGAGTGGTGAATGGTGGTGGAGGTGGTGGCGGGGGAGGAGGATATCCACCGGCTCAACCAATGGGGTTGGCAGCACCTGTAAGTCCAGTATCCCCAGAAGGAATGTGCACAAATCAGGTGGATAGCTCAAATCAATTTGGGTTGGACTTGGGTGGGTTGAGAGGTAGGAAGAGAATAATTGATGGGCCAGTTGAAAAAGTTGTGGAAAGAAGACAGAGAAGGATGATAAAGAATAGAGAGTCTGCTGCAAGGTCTAGAGCTAGAAAACAGGTGCATTTTTCTTTACCTAGTGTCAGCTTTGACAATTCGATAAAATGACAAGAAAATGTAACTAATATGCATTTTCTTTCTTACTTTGAAACAGGCCTACACAGTAGAGTTAGAAGCAGAGTTGAACCAGTTAAGAGAGGAAAATGCTCATCTTAAGCAGGCCTTGGTAAGTATAAAGTACCACATAAACACATTTGATATCTCAGATAGCAATTGCCTACCATGGTGATTCTAACCAGATTTTTTCCTTTTTGTTTTGTAACATTAACAGGCAGAGCTTGAGAGGAAGAGAAAGCAACAGGTACCTTTTCCAAAACAACTAGCTAGATTCTGCTTGATCCCTAACCTCATACAATTTGAAGCAATGGTAGGATAGAATTACATGTCATTTCCTTCTGATATGAAAAGAAATGTAGGATTGATATTTCAAATAATGATTGATTGCTATATCTTCTCATGTGTAAAATGTATGTACATTTACAACTTGTGCATAGCATCTATCAACATACGTGTTTTGCAACTGTAAAGATATCTATCAAGTCAGAACCTCTCTTTCTCAGTTTTTTAATGTCTCTTAAGTTGAAAAATTAAAGTAGAAAACCTTTTCGATTCGTAGAAGGTTTTAGATTTGTTGTTAGATCCTAATCTTGCTATCTGTGTATAAAAATTGTTATTTCCTTGAATTCCTTTCTTTAGAGTTCAAGAACACAATAATGTCGTTCCTTCCTTCAACATCTTTCTAAATCCAAGTGTTTGACCTGATTGTCTCAATAATCATGATATGTGTTTTAAAATCATGGATTTGAGCTGCATCTGTCTGTCCTTCAACAGGTACCTTTTTTAAAATATCTAAGATCTAACTTTGCCAACCCTTCATTTCTGCAGTACTTGGAGGAAACGAAGAATGTTCACACGAAAGCACAAAGAGCCAAGGAAAAGCTTCGTGTTATGAGGAGAACTTTGAGTTGTCCATTGTGA

>ClabZIP52

ATGGCTTCTTCTTCCAAGTGCTCCGACGGAACCACTTGTTCTGGTTTGAGTTCTTCGTCTTCTTCTTCCTCCTCCATGTCCTCTTCTATGGCCAAGGCGGCGGATCAGATGGTCAAGGTTGAGATTGAGGCGGCGGAGGCTCTTGCTGGTTTGGCGGTTTTGGCGGTCAGAGAGACGGGACCTCAACCGTCCGAAACCAAATGGGGGATTAAAGGGAAAGGGAAACGAGCTAGGAAGGAGGTTAAGACCGAGTCGCCGACTTCTGCCTTTGCCGACTCTTTACCTAGTCGCGCGGATCTGAACCTTCGGATTGAGGTTATTCTTTACTTCTTTTTTTTTTTTTTTCTTTTTCCTTTTGGTTTTGCCAGATTTTGATTGCATTTTTGTTGAAGTTCAGAATTAGGGGTGGATTTCTGATTGTGTGTTTTTAGATGGAGGACTGAATTTTGCCCCCGTTAATGGTATAGTAAATTGAGTGGGGTGTTCCTACCTGAGCCATTGTTAATTGAGCGAGGAGCAATTACTCGTCTGATTCTATTTTTGAAAATTTGGATGCCTTCTGAGGCAATACGATGGAATGCTAAGCTATCTGGTTTACATTCACTACGTATTTTTCAAATGTGTTTGCCTGACGGTTCGTTAACTTCACATTTCTATCCTTCTTTGGTGTTTAGATTCTAAGGACTAGGATTCAATTTGGTGATGCTTATCCCAGAAGTATGGAGTAGAATTAAGAATGTGGAGATCTTTGTCTTGGATTCTAAAACAAGTAGTGGGCCATTGCTAGATTAAGTGACACCATTTGCTAACTCGTGAAAGCATATTAAAACTGAAATAGGTTTGGTAATGAAATTTCCTCCAACGGTTGTGGAAACATTTGCCTTTTTTTTTTTAATTTATATTTATCCGCACATCGACTAATCTCGTGGGCAACTTGCTATCCTACAACATTTGAATGTCAAGGAATTCGTAGGATATTAAATCCTTCTGGTAATTATTGGTGACTGGACTAGAAAAGTGGAGCACTTGGCTTGTCTTTTCGACATGTCCATTTGTCTTTCCCAATTAGGTCCCAAGTTCACTGAGATTTATGCTCGTCTTACTACAAGCCTATTTGTTGTTCCAAATTAGGTCCCGGTTTCTGTTATTATGGCTTACACCAAACTGCTAAACGAGTTAAGTTGTATTACTATGCTGCCCATACGAAGTGTTTGATATTCTACAAGTCTTCTTGACAACCAAATATAGTAAAGTCAAGCAATTTTCTCGTGAGAACTGAGCTAGTGTATTCAAGCTAGCTTGAACACTCATGTATATTAAAAGAAAACAAGAGTTACACTACTTATGCAGGCCTTGACAAACTATTTTTGTTTGTAGCAGGAAGATAGAGGGGTGGTAAGACATCAGCCATTAGAAAAAGAATGTACTAGTCAGTCCCACCCTGAGTGGGAAACAACTGGAGAGATGATGAAGGTAGACAAGGAGGCCGAATCATGTAAAGTGAGTCCTGCATGCACCACAAGCTACCAGTTATTTGGCTGCAGGAGATCAAGACGTACTCTAACTGAGGTCATGATTGCTAACTTCTTCCCACACATTTTTACTTTGTTTCAATTTTTCATAAAGCGGCTTTCTTCTTCAGGCTGAAAAGGAAGAAAGGAGGGTACGAAGGATTTTAGCAAACAGAGAGTCAGCCCGGCAGACAATTCGGCGTAGGCAGGTTCATAGAATTTCTTTTCGAGAAGTATTTTTCAGTTTTATAGTTCAGTTGTTCAAATTACGTACCATATATTTAACATGTCTTGGACATCATTGTAATATTTTATCATGCAAATATGTTTATCCAAGATGAAGGTTATTGAAAATCCTTCTTGTTTTCATTCTTTTGTTTGGTGGATTTCCATATCTTACACTAAACCATCTGTGGATGTCTTTGGCTACTTGTTAGTCGCAGTAGAGAGGAGATTTAGGCTCTAAACCAAAAAATTTTAACATTACTCTAAGTCACTCTAAACATGATAATAAAAATGTCCGTCAAAATTAACTCTCTTTTTTATCTTTTTTAACTAGATTGCTATACTGTTGGAAGTTACTTGTTTTTCAACTTGATTGCTTTACTTTTGGAAGTTATTTTTTGTGATTAACCGTACACCATACTCATATTGTCAGTGCAATAACTGGTGTCGGCTTCTTCACAGTCCAACTTTAATCCTCAGTAACAAGTTCAAGGGAGATTAACATCCTTACTTGGTCTAAGGAAAGAAAATACCAGGTGTTTTTATTCTTCGACACCATCAAGAAAGGTTAACAATGTCAGTTAAGTGTGCTATTATGATTTAATTTATTACAAAGAAAGCTTTAACTATAAAACTATTCTGAATCATTGCTTTACAAGTGATCAAAATCTCCATAAATTAATTGGTTGATTGGATATCTTTCTGGGTATTATTCTTGGTGATGAGCTAAATTTTAAGTTAAATCAGTTTTCATCAAAAGTAATTAGAGAGTCCGTGGTACATAGTTGCTTTATTTAAATTACATTTGCATGTAGTAGTTCATTTTGGTTGGTTATTATGCAGGCTCTGTGCGAGGAGTTGACCAGAAAGGCTGCTGATCTAGCATGGGAAAATGAAAATTTAAAGAGGGTAGGCGGTCACGAAAATGATTCTATTTTCTTCTTTGCACCCCCCCCAAAACACCAAAAAAAAAGGACCGATTTATATTCTTTAACTTTCTGTTTGCCATAATCTTTATTTACCTTTCACCTTGTGATCTTCAACAATCAAGGAAAAGGAGTTGGCCCTGAAAGAGTACCAATCTCTGGAGACCACTAACAAGGAACTAAAGGAACAGGTAGGATCGGGTTGCCTTTAAGAAGTGTGTGTGTGGTCATGGTCTAACGGTATCCCCCAACAAATTCACTTGCTCGCTCGTTTGTGTCTAGTTGGCTGAAGCAGTAAAGCCGAAGGTGGAGGAGATCCCAGGAAACAATAGATCATCTCATGTTCAGATGCCTCCTTTACCTACCAACTACCCTCTTTTCTTGTTTAGTCGCCTTCCATATTTCTGGCCATCTGTGGTTCAACCTACAAGTCCCTATCATGAACTACCCAATGTTGTCGTCGTCCCGTCAAGTATTAATTTGCCTGCAAATAGTAATGTTTCTGTGTCTGGCTCTTCTCATGTACAAGAAAACTTTACAAACGCCTGTGGCCCGAGAACACCCTTGTGTATACTACCACCTTGTTCTTGGTTGTTGCCTCATCATGATTTTAGGAACCAACAGAGTCCTCAAATCTGGTTTCCCGCTGGAAATAATCTAGAGGATATTTATTCGAAATCCCAAAACAGTGCTAATACTTCAAAGGTTGTGTGTGCAGAAAGCAGACAGTCTTCTTTGCCTTCAGCTGAAGAAGAAAACGATGCTCCTGACTTGAATGAACCTCCTAATTTAAACGAAGCTTCGAATCCAAAGGATCATGCTCAGAACTCAGTTGGAGTATCTGTAGATGGATTTGATACCAACGCAAGACCTCAAGTTAGAGAAGTACTTTCTCCTGTAAGACTTGAATGTATCGAATCCAGTTCCGCTGTCAAACAAGGTAACCGGAGCGAAGATGATCACGGTCTGTCATCAAGAACTTGTGATGACTTATTTGATTTTGCGGAAAGAAGGCACAAACCAGAGATAGCTCCCTGTAAGAAAACCATAGATGCAATGGCTGCAACTGAGGCAAGGAGGCGGAGAAAAGAACTCACAAAGTTAAAGAATCTTTACGCCCGTCAGTGCCGTATGCATTCCTGA

>ClabZIP53

ATGAGCTCTTCATCAACTCAACTCTGTGCTTCAAGGATGGGCATCTATGAGCCATTTCACCAGATCAACTCATGGGCAAATGCATTCGGTAGTCGACTAGATACGAGCATATCACCTATTATAAAAGTGGATGACTGTGTAGACATTAAAGTGAGAATACTCTATATTGGCTCTTGTTAGAAAATCTGAGTTTCCAAGGTTATCCTCTTGTTGTTGGATTCTGGTAACTTTTGTTTCCTGACTTTGTAGCCCGAGTTTGTTCCTTTTGAATCAATGGATCATCTCGAGAGCAGTCAAGAAATGAACAAGCCAATCGATGATAAGGTGAATAGACTACAAACCAGCTCTCTCTCCTGCAAGCTTCATTTCTTTCCTTTGTTCTTTTCATAAGTTTTGTTTTCTTCTTTTCCTTTTTGCACTAAGTTCTGTGTACATCATCCAATACGTATATGCAGGTACAAAGACGTTTAGCACAAAATCGGGAAGCAGCTCGTAAAAGCCGTATGAGGAAAAAGGTGCTGAAAGGCAATCATTCGTAAAAGTTTTTGCTGAGTATCTGACATATGATTTGAGGACTTATTGAACTTTGTTGCAGGTTTATGTGCAGCAGTTAGAAACTAGCCGTTTGAAGCTGCGACTGTTGGAGCAGGAGCTAGAAAGAACTAAGCAACAGAAGGTAACATGGAAATCATCTTTTCTTATACTGTCTCTATTGTGTATATTTACATGAATTCTTTGTTGCAGGGTACCAGCTGCTTAGTAGATATCAGCCACTTCGGATTCAGCGGATTTGTAAACCCAGGTTTGTGATTCCATGTCTCAACTCGGACGAGCTCACCTCATAACTTGAGTTATCATTAACCGGTTGAGCTTACTTTGTAGCCTTACCCTTCCTTTATTTCGTTATCATTCAAAGTACTTTCTTTCTTCGCCAATCCAACGGCAGAAGGTCTTCATTTCTCTTTAAACACAAACAGAATTCTAACTGAGTTGTTATGTTGTCCTTGAAAGAGAAGAAAGATAATCTTTGAGTTGGTTCCATTCTGAGATTTTTGCTATTCTACTTTCAGGGATTGCTGCATTTGAGATGGAATACAATCACTGGGTTGAAGAGCAACAGAGACAGATCACCGAACTCAGAAAGGCGTTGCAAGTTCATACAACTGATATAGAACTCCAAATTTTAGTGGAGAGCAGCCTAAACCATTACCACAATCTGTTTTGCATGAAAGCCAATGCTGCAAAGGCTGATGTTTTCTATTTAATGTCTGGTATTTGGAGAACATCGGCAGAGCGTTTTTTCCACTGGATAGGAGGATTTCGCCCATCAGAGCTGCTAAACGTAATAACTTACCTCACAGTTAGCTTGATCTTTGACCATTGTTTGCCACTTATTCCATATTATGCATTCATTCTTTTAGGTTATATTACAAGCGTGTTGTGTTTTGTTGAGGTTAATTGTATATTGGTCATACTAGAAGGTATCAGTTATTATTTATATCGGAGCTTGAAAGATAGAAGCTTTCATAAGGGAATTCTTTTCAATTTTTATTCCCAAGTAGAACCTTGAAGAAGTATCAGAAGCGATCCTTTGAAAATCACTACCAAAATATTTAGTCCGATACTTGATGATCAATGGTAGAGAACTTCTTGATCTGACTTTTTAATGAAATTAAACTTTACTATGATATTTTTAAGTTCATATTATTCTTCTCTAGTAAGGTAGACTATAACTAAAATCCCGATATTCGATTACTCGTCACTATTTTGAATGTGCCAACATGTGCACATTCTCCCTTTCTTTTTGCATTATACAGCCAAATAACATATCAGTATCCTAGTTTAGATGGTGAAGGAAACCTTTCATATATGCTGACAGGTTCTGAAGCCATACTTTGAGCCATTAAATGAACAACAAAGAGCCGATATCCACAAGTTGCAACAGTCGTCTCGGCAAGCTGAAGATGCGCTCACACAGGGAATGGAAAAACTGCACCAGAATCTATCCCTCAGCATTGCCAGTGATCCTATAGGAAGCTATATTTCTCAGATGGGTGATGGAATGGAGAAACTAGAAGCACTGGAGAGCTTCGTAAGCCAGGTAAAAGCACATTCTTTCACAATAAATGGAGTTCATTGCTTTCTGTTCCAACTTTGTTCCCACATTTGCTTATTTTATATATTTGTATGATCTTATCCTCTTTTTCATGATAAGAGTAGGAAGATATAGAAAGTCTAAAATTCATTAAGGTGACATTTGAGTGTAGTAAACTTACAAACTGTTAGTATTGGGTGAGTAATGATTCCTTAGATTGCAGTGAATACATCTCAAACTATTTTCTAAATCTTTGGAAGGGGCAATCTGTTTTCTGCGACTCCAGAATTTTCAATACTCAATTGACTCATCATATTTCCTAAAACAACCTTACTTGAACAGGATATGTTCTACATGATGTACTACTCTGTCCTTGAGTTATAAGCAAATTATTTCCTAAAGCAACTCTTCGTAGTATTATGACTTTTCATTCTAGGTGCTGGCTAACTGATTTCATTAGTATTAACTTCTTTTCCTTTTTAAGAAGTCAATACTCTTCTATATCTTCAAAATCATTCACTTCTTGTAGAGGATCAAATCTCTAGAGAAACAGAATGTTTCCCTTCCTCAGAACCCTCCGCGTAGAAAAACAAACTGCTCAAGGAGCAATTGGCTTATTATGCTATTGACGTCTAGATGTCTGTTTCTTTATGAAAACTAGAGTATGATAAGTGTATGACAATAGTTTTTCTTCGATCCCGAACCCTCACTCGTTCAGCCGAGTGTGTGTTACTCTTTTCTTGAACAGGCAGACCATCTCAGGCAACAAACATTGAAGAGAATGTCTCACATCCTAACCACCAAACAGGCAGCACAAGGCTTGCTCGCTTTGGGCGAGTACTTCCACCGTCTCCGCATCCTCAGTTCGCTCTGGGCTACTCGACCTCGTGAACCTGCCTAG

>ClabZIP54

ATGGAGAATTCCAAGGTGTTGTCAAACATGAGAAATATGATTTACTCTGGAAAGCATGCTCTACTTCCTCCTAAGAGTCCACTTCCTAGTGGGTCATCCTCATATGCTGATTATTTCCCTAATCCCATTATTGGGTCAAGAGCAGTGCAGAATCCCAGAGAGGGAAATGTGCACCATCATAGAACATCATCTGAAAGTCTTGTGATGGAGGAACAACCTTCTTGGCTTGATGATCTCCTCAATGAACCCGAAACACCTGTTCAAAGAGGTGGTCATCGACGTTCATCAAGTGACTCCTTTGCTTACTTAGATGCAGGAAATGTTTCAAATGAAAATTATACACAAGATGACTCCCAATGTAAAAATATGTATTTACCTTCCTGGGCATCTCAAGATTTTGATTCCCATCAAGCTTCATTTCATATGAAAGCAAGCTGGATCAAACAGAAAAACAGGACACGGGAATTGCCTCCAACTACATTGACAACTAACCCAGGTGCCCGCTCTTCTGCGAAAAGTAGCATTCTTCTTGAAAGCTCAAGGTCGTTGAGTACTACACCACAGGAAGCAAATGGGTTTTCCTCAACTACTGAAAAGCAGGATTCAGCAGAAACTGGTCTGCCTGATAGGAAGCCATCTGAAAGAATGGATAGTTCTCATGTTAAGCCAGGTCTGGCTGATACAGATAATAAAAGAGCTAAACAGTAAGTTTTGCATTGCTTATTATCTACATTATTTATACGCAAAAACGTCCTCTCCCTGCACAGTCTTGAATTCTGTTTTGCAATCGTGTCTCCCAGTTTGTTTCCAATCTATTTGTTTGTGTTGTACCATCTTTAGCGAGCTTGTTGCTCTAGTTTTCCTTGAGGTTTACAGTCTGCTTTTACACTGGAATAACTTCAATGCTTTTCTTTGCTAAGGAAATGTACCGAAGAAGCCAGGATAGAATTGGAGGGTTTTTTCCCCCCAGAAAAGCATTGGTCTGCAAATTTAAAATTTTCTTGTGGATACTTGACTCTTGAGAATGCTTGTTATTTTGTCCGACCTAGTGATGAACCCCTCTCCTCCCTCACTCGGGCCGTGTGCTCATTCAGTAATTGGGAGAGAATTTTCAATATAAAACTTAATGATATTGGATGGATGTGTGTATGTAACTTAAGAAAAACTAAGTATAACTTCCAGCATTAGAGAGAGAGAGTTGCAGGTTTTCGTTATGTTATGAAGAGATATCTATGTATACGATTTAACCCGACTTGTTATGAATACAGGCAATTTGCTCAACGTTCACGTGTACGGAAACTTCAGTACATTGCAGAGCTGGAAAGGAACGTACAAGCGTTACAAGCAAATGGTTCTGAAGTTTCTGCCGAACTTGAATTTCTCAGTCAGCAAAACTTAATTCTTGGCATGGAGAATAAAGCACTCAAGCAACGATTAGAAAGTTTATCACAGGAGCAGCTTATAAAATACCGTGAGCTTCCATACTCTCAAACTTGAGTTTTGACAGTCCATTCTAATGGTTTCATTTTTTCAAAAACGTTGAATCACTTTTGGATGAAAATTCCTTTCTCCTTTAGTCAAAGTTTCAAACTGAATTTAGAAAGAGAAATGTAATAATTCCTATGTGAAATTATATTCAGTGGAACATGAAGTACTGGAGAGGGAGATTGGAAGACTAAGAATGTTGTACCAACAGCAGCAACAGCCACAGCCACCACCTTCCAGCCTTAAACGCACCAAAAGCCGAGACCTTGAGACGCAATTTGCTAAGCTCTCTTTGAGACAGAAGGATGCACGTTCAGGTTCCGAGTCTGTGGCCGGTCCAGTCCAAATCTAG

>ClabZIP55

ATGAATTTAGATGAATTAGTTTTCAGGAATGTGATGTCTGTGGAAGAAGCAGAGCTGGTACATAACCCTTCCTCTTTATCTCCTGCAGCAGCCACGGCTTCATTGTTTCTCGGGAAAAGAAACGACGACGCCGAACCGCAGCACGATCCAATGGCTGAGGTTTCAGCCCCGGCAGAAGGGATGGATTGGATGCATTACCAACGAGCAATGCTGATTGATTCCAAGTTGCCAGTTTCTCAAGCTGTTTACAATCATGGGAGTGTACCAGATATTGGTGTTTACAATATGCAGGCCATGTCGATAACGACGTCGGCCTTGTCAAACTCAGATTTCCAGGAAGGTAATTGTGGACGGAAACGAAGACAGCTGGATGATATAAAGGAGAAGACTATTGAAAGAAGGCAGAGGAGGATGATCAAGAACCGTGAGTCTGCTGCAAGGTCCAGAGCCAGGAAACAGGCAAGTTCATGA

>ClabZIP56

ATGCCCAATTTAACTCCTTCTCCTCCTCCTCCTCCGCCGCCCTCCGCCACCCACCACCACAACTCTTCTTGGGTCGATGATTTTCTTGACTTCTCCACCGCCCGCCGCGGCCTTCACCGGAGATCCATTAGCGACTCCATTGCTTTCCTTGAAACCCCATTTTCCGATCAATGTCGTAACTCCGCCCTTCTTCATCCTCCTCCTCCTTTTGACCGCCTTGACGACGACCAACTTATGTCCATGTTTACCGATGACATTTCCATCCCCATTCCTGCTTCCCCCTCCAATCCTTCCACTCCTTCCGACCAAAATAGTAACAACGACGACAAAAACCCAACCGAGGACGCCATGTTTCCGCCTCCACCGCCACCACCTCTGCCTCCGCATCAGTTGAAAAATGAACCTGGCGAGGTTGAAATTAGCTCCTCTTGCCAACTGCAGCAGCAATCTCAACCACCGCCGCCTTCCTCTGCCGCTGATAATACCATTGATCCCAAAAGAGTTAAGAGGTATGATATCTCAAATTTCAATCAAGCAATCAATTCCAAGAAAAACACACACACACACACACTGAATTATGAAATTTTTTTTTTTTCTCTCAAAATTCTTGAAATTTCAATCCAATAAACTACTAATTACTTTAATTAACATGAATAATACTCTCCAAGCACATTTGCAAGTAAAATATTTTATTGACATTTAATGTAATTTAAATTAGGTATTATATGTATATAATTGTTCATTGTAACTTTTAAACCACTAAATTACAAGAAAACTCACAATAAATTTATTTTTTTTTCTTACTCACCAATATATGTAGTCAAATTATTTTTGATGAATTAATTTGTATGGCATTTAATAAATAAGTGGACTTGAATAAAACTTGATCATAAAAGAGTCAGACGAAATTCTTACTATTCTGCCTACTGGCTGATATTTGTAGATGTACATTTTCTAATTTTATTTTTAATATGTTGATAGTATTAGTTACATGGCGGTGTACGTGTCAAATTAGTGGTAAACAAATTAAAAGAAAAAACTTTAGCTTAAAAGAGAAAACAAATTGAAGGTCAAGGAGTCCTCCACATATAGGAGGATAAAAATGTAAAGTTTTATGTTGATCACATTTGTGCATGGGTGACCACTACATTATAATTAGTGTGTAATTAACAACATAATGAAATTAGAATTAAAGCATAATAATGATTGGTGGGTGGATCTCTTTTTTGTGTATTAGAATTTTGGCAAATCGACAATCAGCACAAAGGTCGAGAGTCAGAAAATTGCAATATATTTCTGAGCTCGAGAGAAGTGTAACCACATTACAGGTACGGATTTAATTTAATTTAATTTAATTTACTCTCTTTAATTAAATATATATTTTATTTTTATAATCACTAATAATTAGTGTTTATTAATTTTTGTTTTTGGGTTTGTTGTGATGGTAGACTGAAGTATCAGCATTGTCTCCTAGAGTGGCATTTTTAGACCACCAAAGATTGATTCTAAATGTCGATAATAGTGCTCTCAAGCAGAGAATTGCAGCTTTGGCTCAAGATAAGATTTTCAAAGATGGTAATTCCTAATTTTTCAAACCCATTTCTCCCTCTCTTCCTCTCTTAAAACTACTAATTCTTCTTTTTTATTTTTTTATTTTTTTTTAATTTACCACATGACTAACATAATTAGAAATGGATGGCGAAAAGATAGATGTATACTATGATACACAATTTGTACTCCTTAATGCTACTCATATTTTAACATGTCATTAGATGATATATTATTTTGGATAAATTACAAATTTAATTGGTATTCCGTTTATGAGAATTTTATCAAACATAGGAATTATTTATAGAGTTTTAGACTTAATTCTAACATTTTTCCAAACTATAAGAACCAAAATCTACAATTTAATTTATTATTTTATGGGGTTCACATAATTTTTAATGTTTCTTTTAAGACAAGCCATGAATGGAGATGTTTTTTCATAATGTATTTTTCTTAAAGAAAATGGTAATAAATGGTTTTCTTTTTATATTATTATTATTGTAGCTCATCAAGAGGCGTTAAAGAAGGAAATAGAGAGATTGAGGCAAGTTTACCACCAACAAAGTTTGAAGAAAATGAGCGGTAATCAAAATGGAGCTGCGCAACCGCAAGAACCGCCGTCCGGAGAAGGTACTCCGCCGCAAGCGGCTGAGATGGAGAAGGGGCAAGCTGTAAGTTGA

>ClabZIP57

ATGGAGGAGGATCTCCATTTCTCCGAATACGACTTAATAGGGCAGATTGATTGGAACGACTTCTTTGATGAATTCCCGGAGGTGGAGCTCCCTCTTGCCGGGGATTCAACATCGCCCCACGGATCTCCTGATTCCTTTTCTTCCTGGATCAACCACCTGGAAAACGCTTTATTGAATGACGATCAAGACAAGGGGGTTTCCCTTCCCACTCCTACCGATGATTGCTGCGATAGTTTTTTGGCTGACGTCCTCGTCGACACTCATGGAGGAGCCTCTGTAATCGACCTCGATTCCAATGCTTCCGATTGCGGTAACCAGTTTAGCAGTTCCCAGAAGGAGGATGGACATAAGGTTTCTCCCGCGCCTACTGACGATTCCTGTGGTAGTTTTATGGCTGAAGTACTTGTAGATACTCACGGGAGCTCCTCTGGGGTTGATGCTGTAGTTGACGTTCTGTCCAACGCTTCCGATTGCGGTGACGACTCGAACAATTCCCAGAAGGAGAAGATCGACGCAACAAACATCGACGATAGTGTAGGTGAAGATGTCGATGATTCGCTTTCTAAAAAGCGGAGAAGGTATTGCTACTGAATCTTTTATGAGTTGGCATTCTACTATTTTGATCAATTAGGAGGTTTCGCCATGTTAATTTATAGTCTCGTTAAACAATTGTAGTTTCTGCCATGAACATCTTTTTCTAGCTCTTCCATTTGTATCGGTATTGTATCTATTTGGTTTTCTTTTTTGAGATTAAGAATTACTTCCAATAAGTCACCATTTCCCCCAAATATTAATTGAAGTTTGAGGACCTTATATTTCATCTGGGGTTCTTAATTCGAAGTTGTCTTGTATGTTTGAAACACCAAACATACAAGAAGGACGATAGTAAATAATTAAGAAACCGAAGATCATTTTACTTCTGGGTATCAGCTGACATAATATATGAACGCCGTTCGAAGGGATAACTGCAATTTATGAATTCGAATTTTTCAGGCAGCTGAGAAATAGGGATGCCGCAGTAAGATCTAGGGAGAGGAAAAAGATGTACGTAAAGGATCTAGAGATGAAGAGTAAATTTCTTGAAGGTGAATGCCGGAGATTGGGGCGTTTGCTCCAGTGTTACTGTGCTGAGAATCAAGCCTTACGGTTTAGTCTACAGATGGGAGGTGCTTCTGGTGCTTCACTGACCAAGCAGGAGTCTGCTGTGCTCTTGTTGGGTACGGTTTATCTCTTCCTTTGTGCCGTATCATTTTATATATGGCCAAATGAATTTTTGTCTACTGCTGGTAGTTTGCATGTCAACATAAGAGAAATAATTGAAATATTTAAATGGTAAAGTTACAGATATTTTCCGCATCCATGTTGTTATGGACCTTTAATGTCTTTAAAGAGTAAGTAGATGAAGAACATCTAAAGCTCCATTTGTTTTTAAAAACGAGTTTATGGTTTGGAAAATTGGGCTAAAACTGTTCTAGTAGACAACAAACCGTGGTGTTCAGACATAATTTTAAAAGACTAAAAATGAAAAACCAAAGGTCTCCAAAAGAGACCTACGCGATTCTCCTTTTATTTTCTGTGTAGGTATGGTTTTGATGGTTTTAATTAACATATATATACACACACATATGCATACGTGATCCTCTTTAAAAGGACCGCTCATGCATTGAAGAGTAGATTTGAGTATTTAACATTGCTGTCTTTTTATCCGACTACACTTCTCATCAATTGTATTCCTCCAGTCCTGATGAGAAGAAATTATATTTAGAGGCTGATATAATGTGGTGGGTGGTGTTGTGTACTGTTGTTGGGGGTAGCAGAGTGAAAATAAAATTTTTACTAATAACACAAACTTATAGTGAAAGGGTTCAGAGGGTGGCTCCAAACCACCGCAAAAAGTGAAAATTGTAAAGGAAAACGTTTGGGGTGTCAATTTTGAGTAAACTTGGCCATTGACATATTATGGACCTGAGAAAGTATCCATGACTCTGCCTCGCCCTCTGTACATCAGGCTACCTATCTTAGTCTCTCTTGCTAACCTAACATATGCATGACTGTGTCTACTGCCTAGTGTTATATAATAGAATTCATAAATTAAAATGAACTTGTAACTTTAATTCGGGAATTTTCAGTATTAGATTAGAAGATCTTTTAAGTTTTTGTGCTTCTCTATAATGACTACTTCGTCAAACGTTTGCATCAACAATTTATGTTTTCAAGTGTTATATAATTTTGTTCAAGGTAAAACTCATTTTCTCTCTCTCTCACTCCCTATTGGAGTTCCTCGTATCAAATAGGGAAGATTTTGAAAGTTCACATATCTTTACAGTATACATTATTGCCAATGTTGCCTGACCAATCCAACTGCCAAATCAAGTGGGAGAACTTAAAACAACAAGGAAAGGAAAATAATTTTATCATTTATTTTATTTTTATAGTTTTAAATAGGGAGTTATCTGAACTTGAACTTAGACCTCTGAAAAACAGATCAAACTGATCTATGCTCCATCCTATTCCGTCTTGCCTATTTATACCCATTCCGCCCTCATCAAGTTATCATCCTCTCCCATCCATCCACAACACATTACCCAATGACGTGTACCTAGCATGAATCTCACATTACACAACATAATAGATGAAATCCCTATAATCCTGGATGGAGTTCATCCATCCCATCCCACCCCCTCCTACCTCTCCATCCACAATACACTACCTAATAGATGTGTGCCTAAGATGAATCTGACACTTTCTTTCTAGTTTGGAATCTACAACAAAAGTAGAATAATCTGATCTTTTTGAGTTAACTAGTTTAAGTTAATATAGCACTGTCTTCCTTCCCTTTCACTATGAAGGTTTTAGAAGTTGTGATATACATTTCATTTTTCTCTTAAAATTCTGCTTTCGTGTTTGTGTTTGTGTTCTATCCTTCTTCCGCGTTCAATTTTCCTGAGGGACGGCCACTTACCTATTCTTTTAAAAAATAATGATAACTTGATCTGTTGGTTGATTGAGCAATAGTGTAGCTTGATAAGTCAAATGAATGATGTAATAAATATTCGTTGTTGTCCTGCAGAATCCCTGCTGTTGGGTTCCCTGCTTTGGTTAGTGGGCACCGTTTGCCTGTTCACCCTTCCTCAACTCCCACAATCAACTCTGGAACCTGTTCCAAGAGTAACAATGGAGGACGAAGGTCCAGGAAGCGCGCCTCTCAACGAGAATGAAAATAAGGACTCCAGATACTCATACACATCGTTACAAACTAGGAGGTGCAAAGCGGCGAGAACAAGGATGAAACCAAGTATGTTGGATGCGATGCTCGGTCCTAGTTTGGCTCTGATTTCGGTATAA

>ClabZIP58

ATGCAGCCTGGTGAAGTCACAAGTCTCCAATATCTCATCCCCTCCAATTTATCTCCATATGCTACTCACTTTCCCATGGCACAGAACAACTTACCTACAATGCAACTTAACGAATTCTCCAATAACGAATTCTCCAATCCTTTATATAATTTCCAAGGTCCTTCTCAAGTTCATGATTTTAATCGACACCCATGCTTGAGTAGCAATTCAACTTCTGATGAAGCAGATGAGCAACAGCAAAGCCTCATCAACGAGAGGAAGCAGAGAAGGATGATATCTAATAGAGAGTCTGCACGCCGGTCACGTATGCGCAAACAGAAGCATTTGGATGAGCTATGGTCTCAAGTGGTTTGGCTACGGAATGAGAACCATCAGCTTATAGATAAGCTGAACCAAGTTTCAGACTGCCATGACAAGGTTGTTCAAGAAAATGCCCAACTCAAAGAGCAGACTTCTGAACTCCGTCGAATGCTCACAGAAGTACAGGTCAATAGTCATTACCCCAACTTCAGAGAACTTGAAAAAATCCCCCCCAACACACCATCTGAGAGCTGA

>ClabZIP59

ATGGCTTCCTCTAGTGGAACATCTTCGACTTCTTCATCTATGGAAGAAGGGGAATTAGCGGCGTTGATGGAACAGAGGAAGAGGAAGAGGATGATTTCGAATCGGGAATCCGCGAGGAGATCGAGAATGAGAAAGCAAAAGCATTTGGATGATTTAATGGCTATGGTGGGTCAACTCAGAAAGGATAATCAACAAATCGTCGCTAATCTCACCGTCACAACGCAGCACTACGCCGCCGTCGAGGCTGAGAATTCCATTCTCAAAGCTCAGGCCGCCGAGCTCTCTCACCGTTTGCAATCCTTGAATGAGATCGTGGCTTTCTTGAACCCCTCTGATGGGGTTTTGGAAGATGATACCTACGGCTGCGACGGCGCCGGAGGGTTTTTTAGCCCTCTTCAAATGGCTTTCTATATGAGTCACCCTCTTACAGCTTCTGCAGATGTATTTGGAGAGTATTGA
