# Supplementary material for: The bZIP gene family in watermelon: genome-wide identification and expression analysis under cold stress and root-knot nematode infection
Source: PeerJ. 2019 Oct 16;7:e7878. doi: 10.7717/peerj.7878 (PMC6800529; doi:10.7717/peerj.7878)
Supplement: Supplemental Information 8 [file peerj-07-7878-s008.doc]

**Table S6.** Primers used for qRT-PCR in this study.

| Gene | Accession NO. | Forward primer 5' to 3' | Reverse primer 5' to 3' |
| --- | --- | --- | --- |
| *β-actin* | Cla007792 | CCATGTATGTTGCCATCCAG | GGATAGCATGGGGTAGAGCA |
| *ClabZIP45* | Cla008839 | CCGGGAAGAACAATGAGCTG | ATTGGCATCGCTTCCTTCAC |
| *ClabZIP53* | Cla011901 | GCAGCCTAAACCATTACCAC | GGCGAAATCCTCCTATCCAG |
| *ClabZIP6* | Cla015627 | AGCGTTACATCGTTACAGGC | GTGATGGTGCTGCATTGTTT |
| *ClabZIP8* | Cla015873 | GCTCGATCGAAAGAGCGAAA | TTGTAAGGCCCACGGAATCT |
| *ClbZIP59* | Cla016581 | CGCGAGGAGATCGAGAATGA | ACGGTGAGATTAGCGACGAT |
| *ClabZIP47* | Cla017361 | AGAGCATTGGTGCCAAGGAG | GTTCAAGGACCGCAGACGAT |
| *ClabZIP52* | Cla017709 | GTTCCGCTGTCAAACAAGGT | TGGTTTGTGCCTTCTTTCCG |
| *ClabZIP12* | Cla019809 | GCTGAAGAGACTGTCAAGCG | GAACGGCAGCATCTGTTGAT |
| *ClabZIP18* | Cla021184 | AATCCGCTAGGAGGTCAAGG | GGCAGTCAGCGAATCAACTT |
| *ClabZIP20* | Cla021871 | CATTGCTGAGCTTGAACGGA | CTTGCAGGTGAACTTGCTGT |
| *ClabZIP35* | Cla022056 | TGGCAGAGACATTGTCGAGT | TCGAGGGTCCCAAGTTTACC |
| *ClabZIP36* | Cla022235 | TTGGCTGAACTCGAGAACCA | GCTCTCAATGCGTCTACGTC |
| *ClabZIP37* | Cla022315 | GAGATAAGGGTGAAGGATTTG | TTCTCCTACTTGCCGTTGTG |
| *ClabZIP39* | Cla022580 | CTTCTGCTGCTGCTGTTGAT | TTGCTGCCTCTGTTGCAAAT |
| *ClabZIP56* | Cla023348 | ATTGTCTCCTAGAGTGGCATTT | TTTGTTGGTGGTAAACTTGC |
| *ClabZIP57* | Cla023484 | TTACGGTTTAGTCTACAGATGGG | ATTGTGGGAGTTGAGGAAGG |
